# Supplementary material for: Early Emergence Phase of SARS-CoV-2 Delta Variant in Florida, US
Source: Viruses. 2022 Apr 6;14(4):766. doi: 10.3390/v14040766 (PMC9028683; doi:10.3390/v14040766)
Supplement: Supplementary file 1 [file viruses-14-00766-s001.zip › viruses-1641543-figure s1 conv.pdf]

viruses

MDPI

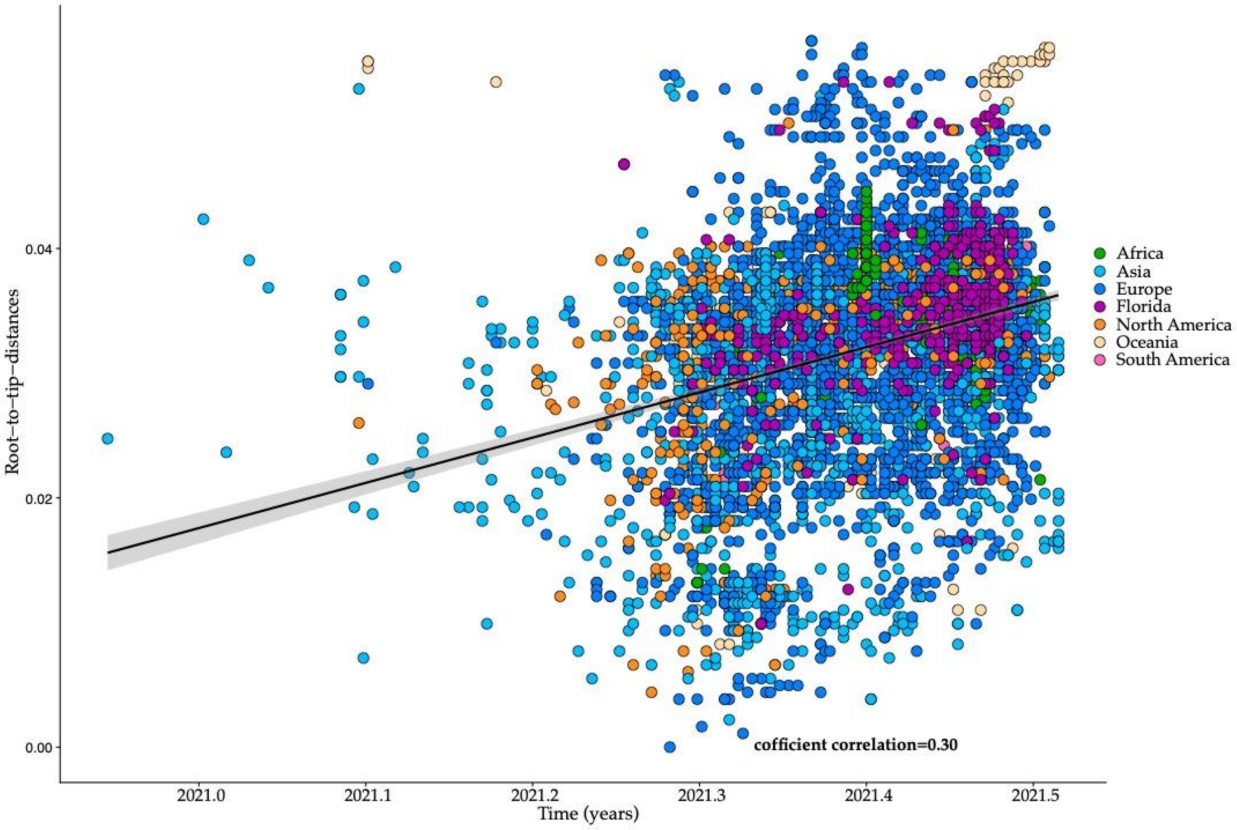

Figure S1. Analysis of temporal structure. Root-to-tip genetic divergence for the whole dataset against time of sampling.
