# Supplementary material for: Early Emergence Phase of SARS-CoV-2 Delta Variant in Florida, US
Source: Viruses. 2022 Apr 6;14(4):766. doi: 10.3390/v14040766 (PMC9028683; doi:10.3390/v14040766)
Supplement: Supplementary file 1 [file viruses-14-00766-s001.zip › viruses-1641543-table s1.pdf]

We gratefully acknowledge the following Authors from the Originating laboratories responsible for obtaining the specimens, as well as the Submitting laboratories where the genome data were generated and shared via GISAID, on which this research is based.

All Submitters of data may be contacted directly via www.gisaid.org

Authors are sorted alphabetically.

Accession ID	Originating Laboratory	Submitting Laboratory	Authors
EPI_ISL_2876027, EPI_ISL_2876032	4Cyte Pathology	NSW Health Pathology - Institute of Clinical Pathology and Medical Research; Westmead Hospital; University of Sydney	CIDM-PH et al.
EPI_ISL_2833513	A. Krumbholz, Labor Dr. Krause und Kollegen MVZ GmbH, Kiel	Charité Universitätsmedizin Berlin, Institut für Virologie	Andi Krumbholz; Barbara Muehlemann; Christian Drosten; Julia Schneider; Julia Tesch; Jörn Beheim-Schwarzbach; Talitha Veith; Terry Jones; Tobias Bleicker; Victor M Corman
EPI_ISL_2721223	A.S.L. CN1	Fondazione del Piemonte per l'Oncologia IRCCS	Antonino Sottile; Giorgio Giardina; Paola Marino; Silvia Brossa
EPI_ISL_2721281, EPI_ISL_2721283	A.S.L. CN2	Fondazione del Piemonte per l'Oncologia IRCCS	Antonino Sottile; Giorgio Giardina; Paola Marino; Silvia Brossa
EPI_ISL_2895117	ABC Algarve	Instituto Nacional de Saude (INSA)	Borges et al
EPI_ISL_2249132	ABC Algarve	Instituto Nacional de Saude (INSA) and Centro de Investigaçao em Biodiversidade e Recursos Geneticos (CIBIO), Universidade do Porto	Borges et al
EPI_ISL_1663516, EPI_ISL_1663522	AIIMS, Patna	Institute of Life Sciences - INSACOG	Ajay Parida; Amol M. Kanampalliwar; Arup Ghosh; Atimukta Jha; INSACOG Consortium; Punit Prasad; Rajeeb Swain; Rupesh Dash; Safal Walia; Shifu Aggarwal; Sunil K. Raghav
EPI_ISL_2231597, EPI_ISL_2231599, EPI_ISL_2231794, EPI_ISL_2373223, EPI_ISL_2774735, EPI_ISL_2774799, EPI_ISL_2774800, EPI_ISL_2774804			
see above	AP SSO	CSIR-Centre for Cellular and Molecular Biology-INSACOG	Amareshwar Vodapalli; Ara Sreenivas; Archana Bharadwaj Siva; B Himasri; Blessy B John; Divya Tej Sowpati; Jandhyala Sai Krishna; Karthik Bharadwaj Tallapaka; Lamuk Zaveri; Onkar Kulkarni; Payel Mukherjee; Priya Nurkurthy; Rakesh K Mishra; Sharath Chandra Thota; Shreekant Verma; Sofia Banu; Sumedha Avadhanula; Tulasi Nagabandi; Valli Nagalakshmi Undamatla; Vidhyadhari Methuku; Viswagithe S L
EPI_ISL_2248966, EPI_ISL_2695437, EPI_ISL_2695438, EPI_ISL_2695451, EPI_ISL_2695454, EPI_ISL_2695496, EPI_ISL_2796236, EPI_ISL_2796263, EPI_ISL_2796281, EPI_ISL_2895142, EPI_ISL_2895143			
see above	ARS Algarve - Laboratorio Laura Ayres	Instituto Nacional de Saude (INSA)	Borges et al
EPI_ISL_2249064	ARS Algarve - Laboratorio Laura Ayres	Instituto Nacional de Saude (INSA) and Institute of Biomedicine (iBiMed), Universidade de Aveiro	Borges et al
EPI_ISL_2716540, EPI_ISL_2861887, EPI_ISL_2861908, EPI_ISL_2861914	ASL Napoli 1 Centro	AMES Centro Polidiagnostico Strumentale S.r.l.	"Giovanni Savarese; Antonella Di Carlo; Antonio Fico; Eloisa Evangelista; Giovanni Savarese; Luigi D'Amore; Luisa Circelli; Maurizio D'Amora; Maurizio D'Amora Antonio Fico"; Monica Ianniello; Nadia Petrillo; Raffaella Ruggiero; Roberto Sirica
EPI_ISL_2873089, EPI_ISL_2873091, EPI_ISL_2873101, EPI_ISL_2873105	ASST MONZA	ASST MONZA	Sergio Maria Ivano Malandrin
EPI_ISL_2604191, EPI_ISL_2638121, EPI_ISL_2638133	AULSS 2 Marca Trevigiana	Istituto Zooprofilattico Sperimentale delle Venezie	Adelaide Milani; Alessia Schivo; Alice Fusaro; Ambra Pastori; Annalisa Salviato; Antonia Ricci; Calogero Terregino; Edoardo Giussani; Elisa Palumbo; Erika Giorgia Quaranta; Isabella Monne; Luca Tassoni
EPI_ISL_2373743, EPI_ISL_2373746, EPI_ISL_2893887	AX BIO OCEAN	CNR Virus des Infections Respiratoires - France SUD	Antonin Bal; Bruno Lina; Gregory Destras; Gwendolyne Burfin; Hadrien Regue; Laurence Josset; Martine Valette; Quentin Semanas
EPI_ISL_2434150, EPI_ISL_2434163, EPI_ISL_2567003, EPI_ISL_2652246, EPI_ISL_2846010, EPI_ISL_2846013, EPI_ISL_2846020, EPI_ISL_2848203			
see above	AZ Klina	AZ Klina	Carl Vael - Lynsey Berckmans
EPI_ISL_1972129	AZ Zeno	AZ SINT-JAN BRUGGE	Jorn Hellemans; Laurien Hoornaert; Marijke Reynders; Patrick Descheemaeker; Thomas Van Landschoot
EPI_ISL_2235305	AZ Zeno Knokke-Blankenberge	AZ SINT-JAN BRUGGE	Jorn Hellemans; Laurien Hoornaert; Marie Madeleine Chabert-Consen; Marijke Reynders; Patrick Descheemaeker; Thomas Van Landschoot
EPI_ISL_2725488, EPI_ISL_2725510, EPI_ISL_2790109	AZDelta	AZ Delta Medical Laboratories in Roeselare, Belgium	Dieter De Smet; Geert Martens; Merijn Vanhee; on behalf of AZ Delta COVID-19 Genomics core (member of Genomic surveillance of SARS-CoV-2 in Belgium network)
EPI_ISL_2833780, EPI_ISL_2833851, EPI_ISL_2833867, EPI_ISL_2833915, EPI_ISL_2833916	Adan Hospital	Kuwait Cancer Control Center	Mona Alateeqi; Shakir Bahzad
EPI_ISL_1562503, EPI_ISL_1690245, EPI_ISL_1690269, EPI_ISL_1736936, EPI_ISL_1995876, EPI_ISL_1997967, EPI_ISL_1999092, EPI_ISL_2000028, EPI_ISL_2000262, EPI_ISL_2039008, EPI_ISL_2039266, EPI_ISL_2041972, EPI_ISL_2042272, EPI_ISL_2042588, EPI_ISL_2090200, EPI_ISL_2090420, EPI_ISL_2090429, EPI_ISL_2146561, EPI_ISL_2147821, EPI_ISL_2148878, EPI_ISL_2149227, EPI_ISL_2150210, EPI_ISL_2150587, EPI_ISL_2158913, EPI_ISL_2159264, EPI_ISL_2180629, EPI_ISL_2180642, EPI_ISL_2181391, EPI_ISL_2201789, EPI_ISL_2203267, EPI_ISL_2368666, EPI_ISL_2370135, EPI_ISL_2370480, EPI_ISL_2421624, EPI_ISL_2421958, EPI_ISL_2422090, EPI_ISL_2489185, EPI_ISL_2489219, EPI_ISL_2489266, EPI_ISL_2489327, EPI_ISL_2489594, EPI_ISL_2489688, EPI_ISL_2489761, EPI_ISL_2527978, EPI_ISL_2528591, EPI_ISL_2528834, EPI_ISL_2528925, EPI_ISL_2600023, EPI_ISL_2686905, EPI_ISL_2687101, EPI_ISL_2687146, EPI_ISL_2687250, EPI_ISL_2687264, EPI_ISL_2687331, EPI_ISL_2687451, EPI_ISL_2687476, EPI_ISL_2687477, EPI_ISL_2687496, EPI_ISL_2687523, EPI_ISL_2687580, EPI_ISL_2784779, EPI_ISL_2784814, EPI_ISL_2784826, EPI_ISL_2784836, EPI_ISL_2784920, EPI_ISL_2784922, EPI_ISL_2784976, EPI_ISL_2784992, EPI_ISL_2785004, EPI_ISL_2785009, EPI_ISL_2785107, EPI_ISL_2785134, EPI_ISL_2785145, EPI_ISL_2785182, EPI_ISL_2785188, EPI_ISL_2785194, EPI_ISL_2785209, EPI_ISL_2785217, EPI_ISL_2785225, EPI_ISL_2785256, EPI_ISL_2785431, EPI_ISL_2785432, EPI_ISL_2785480, EPI_ISL_2832524, EPI_ISL_2832552, EPI_ISL_2832557, EPI_ISL_2875523, EPI_ISL_2875524, EPI_ISL_2875560, EPI_ISL_2875586, EPI_ISL_2875611, EPI_ISL_2875618, EPI_ISL_2875629, EPI_ISL_2875641, EPI_ISL_2875643, EPI_ISL_2875648, EPI_ISL_2875676, EPI_ISL_2875695, EPI_ISL_2875708, EPI_ISL_2875721, EPI_ISL_2875741, EPI_ISL_2875760, EPI_ISL_2875761, EPI_ISL_2875794, EPI_ISL_2875822, EPI_ISL_2875848, EPI_ISL_2875850, EPI_ISL_2875854, EPI_ISL_2875856, EPI_ISL_2875857, EPI_ISL_2875868, EPI_ISL_2875909, EPI_ISL_2875910, EPI_ISL_2875919, EPI_ISL_2875927, EPI_ISL_2875932, EPI_ISL_2875933, EPI_ISL_2875937, EPI_ISL_2875955, EPI_ISL_2875960, EPI_ISL_2875969, EPI_ISL_2875973, EPI_ISL_2875975, EPI_ISL_2875991, EPI_ISL_2875993, EPI_ISL_2876012, EPI_ISL_2877670, EPI_ISL_2877672, EPI_ISL_2877677, EPI_ISL_2877679, EPI_ISL_2877681, EPI_ISL_2877687, EPI_ISL_2877690			
see above	Aegis Sciences Corporation	Centers for Disease Control and Prevention Division of Viral Diseases, Pathogen Discovery	Adrian Paskey; Alec Vest; Benjamin Rambo-Martin; Christopher Gulvick; Clinton R. Paden; Cyndi Clark; Dakota Howard; Darlene Wagner; Dhwani Batra; Dillon Nall; Duncan MacCannell; Ethan Sanders; Holly Houdeshell; Jason Caravas; Kara Moser; Matthew Hardison; Matthew Schmerer; Ola Kvalvaag; Patrick Campbell; Peter W. Cook; Rob Case; Scott Sammons; Shatavia Morrison; Shaun Westlund; Vikramsinha Ghorpade; Yvette Unoarumhi
EPI_ISL_2274979	Aeroporto Internacional de Guarulhos	Instituto Adolfo Lutz, Interdiciplinary Procedures Center, Strategic Laboratory	Caio Vinicius Dias Lopes; Claudia Regina Gonçalves; Claudio Tavares Sacchi; Erica Valessa Ramos Gomes; Karoline Rodrigues Campos; Leonardo Jose Tadeu de Araujo
EPI_ISL_2617090	Africa_CDC - Malawi P6 S2	CERI, Centre for Epidemic Response and Innvoation, Stellenbosch University and KRISP, KZN Research Innovation and Sequencing Platform, UKZN.	Auld A; Chilima B; Chiwaula M; Emmanuel SJ; Giandhari J; Kaba M; Kampira E; Kasambara W; Kim L; Lessells R; Maida A; Mvula B; Mwangomba W; Naidoo Y; Panja L; Pillay S; Tegally H; Wadonda N; Wilkinson E; de Oliveira T
EPI_ISL_2225843, EPI_ISL_2333504, EPI_ISL_2333505, EPI_ISL_2333506, EPI_ISL_2333507, EPI_ISL_2333510, EPI_ISL_2483086, EPI_ISL_2491570, EPI_ISL_2549095, EPI_ISL_2611978, EPI_ISL_2644701, EPI_ISL_2673752, EPI_ISL_2725080, EPI_ISL_2725083, EPI_ISL_2725084, EPI_ISL_2788589, EPI_ISL_2788590, EPI_ISL_2788591, EPI_ISL_2788592, EPI_ISL_2790011, EPI_ISL_2790012, EPI_ISL_2790014, EPI_ISL_2820845, EPI_ISL_2820911, EPI_ISL_2820923, EPI_ISL_2820925			
see above	Akershus University Hospital, Department for Microbiology and Infectious Disease Control	Norwegian Institute of Public Health, Department of Virology	'Kathrine Stene-Johansen; Atiya R Ali; Debech Nadia; Engebretsen Serina Beate; Garcia Llorente Ignacio; Hilde Elshaug; Hilde Vollan; Jon Bråte; Kamilla Heddeland Instefjord; Karoline Bragstad; Kathrine Stene-Johansen; Line Victoria Moen; Marie Paulsen Madsen; Olav Hungnes; Pedersen Benedikte Nevjen; Rasmus Riis Kopperud
EPI_ISL_2617439	Al-Irsyad Hospital	Institute of Tropical Disease, Universitas Airlangga	Aldise M Nastri; Febria Rachmanita; Gatot Soegiarto; Herlin Ferliana; Jezzy R Dewantari; Kazufumi Shimizu; Krisnoadi Rahardjo; Laksmi Wulandari; Maria I Lusida; Resti Yudhawati; Rima R Prasetya; Soetjipto; Yasuko Mori
EPI_ISL_2358230, EPI_ISL_2422123, EPI_ISL_2545629, EPI_ISL_2626920	Algemeen Medisch Labo	Labo Klinische Biologie, UZA	Basil Britto Xavier; Christine Lammens; Herman Goossens; Jasmine Coppens; Marie Le Mercier; Veerle Matheeussen
EPI_ISL_2484898, EPI_ISL_2484899	Amdavad Municipal Corporation, Ahmedabad	Gujarat Biotechnology Research Centre	Chaitanya Joshi; Dalip Singh Rathore; Dinesh Kumar; Hiren Mandalia; Madhvi Joshi; Manish Kumar; Nitin Savaliya; Ramesh Pandit; Sonal Sharma; Twinkle Soni
EPI_ISL_2854052	Ampang Hospital	Institute for Medical Research, Infectious Disease Research Centre, National Institutes of Health, Ministry of Health Malaysia	Anasir MI; Azizan MA; Kamel K; Mohd Zawawi Z; Ramly N; Robert F; Suppiah J; Thayan R
EPI_ISL_1494721, EPI_ISL_1615595, EPI_ISL_1615596, EPI_ISL_1615597, EPI_ISL_1615598, EPI_ISL_1633348, EPI_ISL_1633353, EPI_ISL_1911182, EPI_ISL_1911187, EPI_ISL_1911189, EPI_ISL_1911190, EPI_ISL_1911191, EPI_ISL_1913085, EPI_ISL_2404984, EPI_ISL_2404986, EPI_ISL_2404990, EPI_ISL_2404991, EPI_ISL_2455220, EPI_ISL_2534586, EPI_ISL_2534587, EPI_ISL_2633506, EPI_ISL_2650012, EPI_ISL_2695791, EPI_ISL_2695796, EPI_ISL_2716414, EPI_ISL_2716421			
see above	Area of Virology, Serology and Virology Division (SAViD), New South Wales Health Pathology Randwick	Virology Research Laboratory; Area of Virology, Serology and Virology Division (SAViD), New South Wales Health Pathology Randwick	Au, J.; Bull, R.; Deveson, I.; Foster, C.; Rawlinson, W.; Ruiz Silva, M.; Van Hal, S.
EPI_ISL_2304114, EPI_ISL_2304118	Armed Forces Medical College Pune	National Centre For Cell Science – INSACOG	Ajay Pillai; Dhiraj Paul; INSACOG Consortium team; Manoj Kumar Bhat; Mitali Inamdar; Mohak P Gujare; Shivang P. Bhanushali; Yogesh Shouche
EPI_ISL_2627990	Armies	National Reference Center for Viruses of Respiratory Infections, Institut Pasteur, Paris	Angela Brisebarre; Camille Capel; Christophe Malabat; Corinne Maufrais; Etienne Simon-Lorière; Frédéric Lemoine; Louise Lefrançois; Marine Desroches; Marion Barbet; Maud Vanpeene; Méline Bizard; Sylvie Behillil; Sylvie Van der Werf; Vincent Enouf
EPI_ISL_2137225, EPI_ISL_2427162, EPI_ISL_2484977, EPI_ISL_2485157, EPI_ISL_2657385, EPI_ISL_2657386, EPI_ISL_2657390, EPI_ISL_2657404, EPI_ISL_2657474, EPI_ISL_2657475, EPI_ISL_2657482, EPI_ISL_2657485, EPI_ISL_2657486, EPI_ISL_2657487, EPI_ISL_2657488, EPI_ISL_2657490, EPI_ISL_2657492, EPI_ISL_2657495, EPI_ISL_2657498, EPI_ISL_2757953, EPI_ISL_2757954, EPI_ISL_2757955, EPI_ISL_2757956, EPI_ISL_2757958, EPI_ISL_2757959, EPI_ISL_2757966, EPI_ISL_2757972, EPI_ISL_2757977, EPI_ISL_2757978, EPI_ISL_2757980, EPI_ISL_2757981, EPI_ISL_2757983, EPI_ISL_2757984, EPI_ISL_2757990, EPI_ISL_2757992, EPI_ISL_2758002, EPI_ISL_2758003, EPI_ISL_2758029, EPI_ISL_2758035, EPI_ISL_2758041, EPI_ISL_2758043, EPI_ISL_2758044, EPI_ISL_2758045, EPI_ISL_2758046, EPI_ISL_2758047, EPI_ISL_2758048, EPI_ISL_2758049, EPI_ISL_2758050, EPI_ISL_2758051, EPI_ISL_2758052, EPI_ISL_2758054, EPI_ISL_2758056, EPI_ISL_2758057, EPI_ISL_2758058, EPI_ISL_2758059, EPI_ISL_2758060, EPI_ISL_2758061, EPI_ISL_2758063, EPI_ISL_2758064, EPI_ISL_2758065, EPI_ISL_2758066, EPI_ISL_2758067, EPI_ISL_2758068, EPI_ISL_2758069, EPI_ISL_2758070, EPI_ISL_2758071,			

EPI_ISL_2758073, EPI_ISL_2758074, EPI_ISL_2758075, EPI_ISL_2758076, EPI_ISL_2758077, EPI_ISL_2758078, EPI_ISL_2758080, EPI_ISL_2758083, EPI_ISL_2758085, EPI_ISL_2758087, EPI_ISL_2758088, EPI_ISL_2758090, EPI_ISL_2758091, EPI_ISL_2758092, EPI_ISL_2758094, EPI_ISL_2758095, EPI_ISL_2758096, EPI_ISL_2758097, EPI_ISL_2758098, EPI_ISL_2758099, EPI_ISL_2758101, EPI_ISL_2758102, EPI_ISL_2758103, EPI_ISL_2758104, EPI_ISL_2758106, EPI_ISL_2758107, EPI_ISL_2758108, EPI_ISL_2758113, EPI_ISL_2758114, EPI_ISL_2758118, EPI_ISL_2758119, EPI_ISL_2758122, EPI_ISL_2758123, EPI_ISL_2758127, EPI_ISL_2758128, EPI_ISL_2758141, EPI_ISL_2758148, EPI_ISL_2758163, EPI_ISL_2758167, EPI_ISL_2758174, EPI_ISL_2887417, EPI_ISL_2887420, EPI_ISL_2887429, EPI_ISL_2887436, EPI_ISL_2887439, EPI_ISL_2887448, EPI_ISL_2887456, EPI_ISL_2887457, EPI_ISL_2887464, EPI_ISL_2887466, EPI_ISL_2887473, EPI_ISL_2887477, EPI_ISL_2887484, EPI_ISL_2887485, EPI_ISL_2887486, EPI_ISL_2887487, EPI_ISL_2887494, EPI_ISL_2887527, EPI_ISL_2887528, EPI_ISL_2887572, EPI_ISL_2887573, EPI_ISL_2887604, EPI_ISL_2887610, EPI_ISL_2887616, EPI_ISL_2887619, EPI_ISL_2887621, EPI_ISL_2887625, EPI_ISL_2887626, EPI_ISL_2887629, EPI_ISL_2887636, EPI_ISL_2887649, EPI_ISL_2887653, EPI_ISL_2887661, EPI_ISL_2887662, EPI_ISL_2887665			
see above	Austrian Agency for Health and Food Safety (AGES)	Bergthaler laboratory, CeMM Research Center for Molecular Medicine of the Austrian Academy of Sciences	Andreas Bergthaler; Anna Schedl; Bekir Erguner; Benedikt Agerer; Christoph Bock; Fabian Amman; Jan Laine; Lukas Endler; Maelle Le Moing; Martin Senekowitsch; Matthew Thornton; Michael Schuster; Petr Triska; Thomas Penz
EPI_ISL_2895330, EPI_ISL_2895337, EPI_ISL_2895346	Avelab	Instituto Nacional de Saude (INSA)	Borges et al
EPI_ISL_3031275, EPI_ISL_3031276	Azarian Lab at Burnett School of Biomedical Sciences	Azarian Lab at Burnett School of Biomedical Sciences	Catherine Johnston; Eleonora Cella; Taj Azarian
EPI_ISL_2872658, EPI_ISL_2872660	Azienda Ospedaliero Universitaria di Sassari	AMES Centro Polidiagnostico Strumentale S.r.l.	Anna Puggioni; Bianca Paglietti; Caterina Serra; Claudia Piu; Elena Rimini; Erica Mura; Flavia Angioj; Gabriele Ibba; Giulia Rocca; Laura Firino; Rosalba Govoni; Salvatore Rubino; Sergio Uzzau; Vincenzo Lai
EPI_ISL_2680969, EPI_ISL_2725540	Azienda Sanitaria dell'Alto Adige - Laboratorio Aziendale di Microbiologia e Virologia	Azienda Sanitaria dell'Alto Adige	Irene Bianconi
EPI_ISL_1840787, EPI_ISL_2080266, EPI_ISL_2080267, EPI_ISL_2080268, EPI_ISL_2080270, EPI_ISL_2318924, EPI_ISL_2318925, EPI_ISL_2517124, EPI_ISL_2550649, EPI_ISL_2550690			
see above	Azienda Sanitaria dell'Alto Adige Laboratorio Aziendale di Microbiologia e Virologia	Istituto di Genomica Applicata	Davide Scaglione; Eleonora Paparelli; Elisa Masi; Elisabetta Giacobazzi; Elisabetta Pagani; Gabriele Magris; Irena Jurman; Irene Bianconi; Michele Morgante; Stefanie Wieser; Vera Vendramin
EPI_ISL_2600798	Azienda USL Umbria 2	Istituto Zooprofilattico Sperimentale dell'Abruzzo e Molise "G. Caporale"	Ancora M; Calistri P; Cammà C; Curini V; Delli Compagni E; Di Domenico M; Di Pasquale A; Lorusso A; Mangone I; Marcacci M; Pistoni E; Proietti A; Puglia I; Rinaldi A; Savini G; Scialabba S
EPI_ISL_2728246, EPI_ISL_2728311, EPI_ISL_2728465	BBMP Urban	INSACOG-KA, NIMHANS	Ananthapadmanabha Kotambail; Anita S Desai; Anson Kunjumon George; Chetan G K; Chitra Pattabiraman; Darshan Sreenivas; Ellango Ramasamy; Gautham Arunachal Udupi; Mahesh Kumar.C.S; Sony Sharma; V Ravi
EPI_ISL_2529589, EPI_ISL_2530862, EPI_ISL_2530880, EPI_ISL_2530909, EPI_ISL_2530949, EPI_ISL_2530951, EPI_ISL_2530954, EPI_ISL_2531022, EPI_ISL_2531043, EPI_ISL_2531046, EPI_ISL_2531050, EPI_ISL_2531058, EPI_ISL_2531067, EPI_ISL_2531077, EPI_ISL_2531079, EPI_ISL_2531082, EPI_ISL_2531084, EPI_ISL_2531086, EPI_ISL_2531090, EPI_ISL_2531092, EPI_ISL_2531094, EPI_ISL_2531096, EPI_ISL_2531099, EPI_ISL_2531100, EPI_ISL_2531104, EPI_ISL_2531105, EPI_ISL_2655690, EPI_ISL_2655803, EPI_ISL_2655902, EPI_ISL_2655964, EPI_ISL_2655972, EPI_ISL_2655998, EPI_ISL_2656001, EPI_ISL_2656025, EPI_ISL_2656181, EPI_ISL_2656303, EPI_ISL_2656337, EPI_ISL_2656349, EPI_ISL_2656460, EPI_ISL_2656487, EPI_ISL_2656634, EPI_ISL_2656637, EPI_ISL_2656727, EPI_ISL_2656736, EPI_ISL_2657111, EPI_ISL_2657112, EPI_ISL_2657119, EPI_ISL_2719042, EPI_ISL_2719043, EPI_ISL_2719339, EPI_ISL_2719387, EPI_ISL_2719392, EPI_ISL_2719401, EPI_ISL_2719427, EPI_ISL_2719459, EPI_ISL_2719483, EPI_ISL_2719510, EPI_ISL_2719559, EPI_ISL_2719571, EPI_ISL_2719705, EPI_ISL_2719872, EPI_ISL_2719873, EPI_ISL_2719883, EPI_ISL_2719905, EPI_ISL_2719923, EPI_ISL_2719960, EPI_ISL_2719983, EPI_ISL_2720222, EPI_ISL_2720230, EPI_ISL_2720291, EPI_ISL_2720387, EPI_ISL_2720609, EPI_ISL_2720610, EPI_ISL_2720639, EPI_ISL_2720689, EPI_ISL_2720718, EPI_ISL_2721073, EPI_ISL_2721074, EPI_ISL_2721398, EPI_ISL_2721424, EPI_ISL_2721428, EPI_ISL_2721429, EPI_ISL_2721488, EPI_ISL_2721494, EPI_ISL_2721514, EPI_ISL_2721532, EPI_ISL_2721583, EPI_ISL_2721636, EPI_ISL_2721653, EPI_ISL_2721679, EPI_ISL_2721717, EPI_ISL_2721731, EPI_ISL_2721759			
see above	BCCDC Public Health Laboratory	BCCDC Public Health Laboratory	Ana Pacagnella; Corrinne Ng; Dan Fornika; John Tyson; Kim Macdonald; Kimia Kamelian; Linda Hoang; Loretta Janz; Mel Krajden; Prystajecky Natalie; Robert Azana; Shannon Russell
EPI_ISL_2878555	BIO67-BIOSPHERE	Department of Virology, Henri Mondor University Hospital, Assistance Publique Hôpitaux de Paris, Université Paris-Est Créteil, INSERM U955	Alexandre Soulier; Christophe Rodriguez; Elisabeth Trawinski; Guillaume Gricourt; Jean-Michel Pawlotsky; Melissa N'Debi; Slim Fourati; Vanessa Demontant
EPI_ISL_2894131, EPI_ISL_2894132, EPI_ISL_2894133	BIO86 GENCAY	CHU Poitiers	Agnes BEBY-DEFAUX; Birama N'DIAYE; Caroline MICHAUD; Magali GARCIA; Manon PRAT; Maxime PICHON; Nicolas LEVEQUE; Valentin BON-BARET
EPI_ISL_2229053, EPI_ISL_2878456	BIOMNIS EUROFINS IVRY	Department of Virology, Henri Mondor University Hospital, Assistance Publique Hôpitaux de Paris, Université Paris-Est Créteil, INSERM U955	Alexandre Soulier; Christophe Rodriguez; Elisabeth Trawinski; Guillaume Gricourt; Jean-Michel Pawlotsky; Melissa N'Debi; Slim Fourati; Vanessa Demontant
EPI_ISL_2106975	BPOM Gorontalo	National Institute of Health Research and Development	Arie Ardiansyah Nugraha; Hana Apsari Pawestri; Hartanti Dian Ikawati; Kartika Dewi Puspa; Krisna Pangesti; Nelly Puspandari; Subangkit; Triyani Soekarso; Vivi Setiawaty
EPI_ISL_2288982	BST	Banc de Sang i Teixits	Carlos Hobeich; Francisco Vidal; Irene Corrales; Lorena Ramírez; Maria Glòria Soria; Natália Comes; Nina Borràs; Noemí Gonzalez; Silvia Sauleda
EPI_ISL_2868887, EPI_ISL_2868889, EPI_ISL_2868891, EPI_ISL_2868892, EPI_ISL_2868894, EPI_ISL_2868896, EPI_ISL_2868897, EPI_ISL_2868899, EPI_ISL_2868901			
see above	BTKLPP Kelas I Makassar	National Institute of Health Research and Development	Arie Ardiansyah Nugraha; Hana Apsari Pawestri; Hartanti Dian Ikawati; Kartika Dewi Puspa; Krisna Pangesti; Nelly Puspandari; Subangkit; Triyani Soekarso; Vivi Setiawaty
EPI_ISL_2801892, EPI_ISL_2801893, EPI_ISL_2801894	Banteay Meanchey Regional Laboratory	Virology Unit, Institut Pasteur du Cambodge	Cecile Troupin; Chau Darapheak; Chin Savuth; Erik A Karlsson; Jurre Y Siegers; Kraing Sidonn; Leakhena Pum; Ly Sovann; Veasna Duong; Yi Sengdoeurn
EPI_ISL_2490307, EPI_ISL_2544529	Basurto University Hospital: Clinical Microbiology Laboratory	Biocruces Bizkaia	Ana de la Hoz; Estibaliz Ugalde Zarraga; José Luis Díaz de Tuesta del Arco; Mikel Gallego Rodrigo; Mikel Urrutikoetxea-Gutiérrez; Mª Carmen Nieto Toboso
EPI_ISL_2406459, EPI_ISL_2406460	Battambang Provincial Laboratory	Virology Unit, Institut Pasteur du Cambodge	Cecile Troupin; Chau Darapheak; Chin Savuth; Erik A Karlsson; Jurre Y Siegers; Kraing Sidonn; Leakhena Pum; Ly Sovann; Veasna Duong; Yi Sengdoeurn
EPI_ISL_2801895	Battambang Rapid Response Team	Virology Unit, Institut Pasteur du Cambodge	Cecile Troupin; Chau Darapheak; Chin Savuth; Erik A Karlsson; Jurre Y Siegers; Kraing Sidonn; Leakhena Pum; Ly Sovann; Veasna Duong; Yi Sengdoeurn
EPI_ISL_2260533, EPI_ISL_2260540, EPI_ISL_2260543, EPI_ISL_2262724, EPI_ISL_2262751	Bayerisches Landesamt für Gesundheit und Lebensmittelsicherheit (LGL)	Robert Koch Institute	
EPI_ISL_2529852, EPI_ISL_2529861	Baylor College of Medicine/ GCID	Baylor College of Medicine/ GCID	Charu Agrawal; Donna Muzny; Harsha Doddapaneni; Joseph Pertosino; Paige Farinholt; Pedro A. Piedra; Qinchang Meng; Qing Meng; Richard A. Gibbs; Timothy Farinholt; Vipin Menon; Xiang Qin
EPI_ISL_2333747, EPI_ISL_2878292, EPI_ISL_2878311, EPI_ISL_2878370, EPI_ISL_2878383, EPI_ISL_2878392, EPI_ISL_2878484			
see above	Biogroup Bio Lam-LCD Saint-Denis	Department of Virology, Henri Mondor University Hospital, Assistance Publique Hôpitaux de Paris, Université Paris-Est Créteil, INSERM U955	Alexandre Soulier; Christophe Rodriguez; Elisabeth Trawinski; Guillaume Gricourt; Jean-Michel Pawlotsky; Melissa N'Debi; Slim Fourati; Vanessa Demontant
EPI_ISL_2105673, EPI_ISL_2868422	Biolab Diagnostic Laboratories	Biolab Diagnostic Laboratories	Ahmad Tibi; Amid Abdelnour; Badia Saddedin; Eiad Atwa; Issa Abu-Dayyeh; Lama Hussein; Shaima Ali; Shayma Ali
EPI_ISL_2401509, EPI_ISL_2401510, EPI_ISL_2401513, EPI_ISL_2401516, EPI_ISL_2401517, EPI_ISL_2401526, EPI_ISL_2401615, EPI_ISL_2401625, EPI_ISL_2401633, EPI_ISL_2401651, EPI_ISL_2401678, EPI_ISL_2401687, EPI_ISL_2401709, EPI_ISL_2401720, EPI_ISL_2401729, EPI_ISL_2401749			
see above	BioneXt Lab	Laboratoire national de sante, Microbiology, Microbial Genomics Platform	Anke Wienecke-Baldacchino; Catherine Ragimbeau; Fatu Djabi; Jessica Tapp; Lise Pignon; Raoul Salmon; Tamir Abdelrahman; Thibault Ferrandon
EPI_ISL_2108737, EPI_ISL_2125827, EPI_ISL_2470057, EPI_ISL_2636922, EPI_ISL_2636979, EPI_ISL_2637176, EPI_ISL_2759829, EPI_ISL_2845342			
see above	Bioscientia Labor Wermsdorf	Robert Koch Institute	
EPI_ISL_2761985	Bioscientia MVZ Labor Karlsruhe GmbH	Robert Koch Institute	
EPI_ISL_1970094, EPI_ISL_1970221, EPI_ISL_1970223, EPI_ISL_1970224, EPI_ISL_1970249, EPI_ISL_1970335	Biotechnology Division, NCDC Delhi	NCDC Delhi, Biotechnology Division	Hema Gogia; Hemlata Lall; Kalaiarasan Ponnusamy; Mahesh S Dhar; Manoj K Singh; Meena Datta; Partha Rakshit; Preeti Madan; Priyanka Singh; Radhakrishnan V. S; Robin Marwal; Sandhya Kabra; Sujeet K Singh; Uma Sharma
EPI_ISL_2360256, EPI_ISL_2360257	Boshehr University of Medical Sciences	National Influenza Center	A Nejati; F Ajaminejad; F Ajaminejad and T Mokhtari Azad; J Yavarian; K Sadeghi; N Ghavvami; N Ghavvami and T Mokhtari Azad; NZ Shafiei Jandaghi; V Salimi; Mostafa Salehi-Vaziri
EPI_ISL_2820339, EPI_ISL_2820340, EPI_ISL_2820341, EPI_ISL_2820342, EPI_ISL_2820343, EPI_ISL_2820345, EPI_ISL_2820350, EPI_ISL_2820353, EPI_ISL_2820366, EPI_ISL_2820374, EPI_ISL_2820430, EPI_ISL_2820459, EPI_ISL_2820462, EPI_ISL_2868394, EPI_ISL_2868395, EPI_ISL_2868397, EPI_ISL_2868399, EPI_ISL_2868400, EPI_ISL_2868401, EPI_ISL_2868402, EPI_ISL_2868403, EPI_ISL_2868405, EPI_ISL_2868406, EPI_ISL_2868407, EPI_ISL_2868408, EPI_ISL_2868409, EPI_ISL_2868410, EPI_ISL_2868413			
see above	Botswana Harvard HIV Reference Laboratory	Botswana Harvard HIV Reference Laboratory	Boitumelo Zuze; Botshelo Radibe; Dorcas Maruapula; Godfrey Simoonga; Joseph Makhema; Keoratile Ntshambiwa; Kereng Mphoyakgosi; Legodile Kooepile; Madisa Mine; Modisa Motswaledi; Mosepele Mosepele; Motlalepule L. Pone; Ontlametse T. Bareng; Roger Shapiro; Shahin Lockman; Sikhulile Moyo; Simani Gaseitsiwe; Thongbotho Mphoyakgosi; Wonderful T. Choga
EPI_ISL_2558054, EPI_ISL_2558056, EPI_ISL_2558058, EPI_ISL_2558059, EPI_ISL_2558062, EPI_ISL_2558063	Bureau Of Quarantine (BOQ)	Philippine Genome Center	Alethea R. de Guzman; Anna Ong-Lim; Arianne A. Zamora; Asia Louisa U. Chong; Benedict A. Maralit; Candice Francheska B. Tambaoan; Carlo M. Lapid; Celia Carlos; Devon Ray Pacial; Edsel Maurice Salvaña; El King D. Morado; Elcid Aaron R. Pangilinan; Eva Maria Cutiongco-de la Paz; Francis A. Tablizo; Irish Coleen A. Asin; Jaime C. Montoya; Jan Michael C. Yap; Jo-Hannah S. Llames; John Q. Wong; Joshua Gregor A. Dizon; Juan Antonio R. Magalang; Karol Sophia Agape R. Padilla; Kenneth M. Kim; Kris P. Punayan; Marc Edsel C. Ayes; Maria Rosario Singh-Vergeire and Cynthia P. Saloma; Maria Sofia L. Yangzon; Marissa Alejandria; Razel Nikka M. Hao; Renato Jacinto Q. Mantaring; Rianna Patricia S. Cruz; Sheila Mae M. Araiza
EPI_ISL_2170808, EPI_ISL_2534478	CAP SARRIÀ	Banc de Sang i Teixits	Carlos Hobeich; Francisco Vidal; Irene Corrales; Lorena Ramírez; Maria Glòria Soria; Natália Comes; Nina Borràs; Noemí Gonzalez; Silvia Sauleda
EPI_ISL_2825796, EPI_ISL_2825999	CDPH VBL	California Department of Public Health	CDPH-COVIDNet; UCLA Technology Center for Genomics & Bioinformatics
EPI_ISL_2445707, EPI_ISL_2445709	CEDOC	Instituto Nacional de Saude (INSA)	Borges et al
EPI_ISL_2386977,	CENTOGENE Frankfurt Laboratory: Niederlassung	Robert Koch Institute	

EPI_ISL_2387117, EPI_ISL_2471310, EPI_ISL_2845484, EPI_ISL_2845517	Industriepark Höchst		
EPI_ISL_2893779	CENTRE HOSPITALIER ST JOSEPH ST LUC	CNR Virus des Infections Respiratoires - France SUD	Antonin Bal; Bruno Lina; Gregory Destras; Gwendolyne Burfin; Hadrien Regue; Laurence Josset; Martine Valette; Quentin Semanas
EPI_ISL_2289683	CERBALLIANCE LAURENT DU VAR	CNR Virus des Infections Respiratoires - France SUD	Antonin Bal; Bruno Lina; Gregory Destras; Gwendolyne Burfin; Hadrien Regue; Laurence Josset; Martine Valette; Quentin Semanas
EPI_ISL_2445735, EPI_ISL_2895302	CH Porto - H Sto Antonio	Instituto Nacional de Saude (INSA)	Borges et al
EPI_ISL_2004296, EPI_ISL_2695160	CH Setubal	Instituto Nacional de Saude (INSA)	Borges et al
EPI_ISL_2887746	CH Tourcoing	CHU Lille - Laboratoire de Virologie	AIT YAHYA Emilie; ALIDJINOU Enagnon Kazali; BOCKET Laurence; CREPIN Michel; DEMAY Christophe; ENGELMANN Ilka; GEFFROY Sandrine; GUIGON Aurélie; LAMBERT Valérie; LAZREK Mouna; NOBILLIAUX Florian; PREVOST Brigitte; TCHANTCHOU NJOSSE YANICK; THUILLIER Caroline; TINEZ Claire
EPI_ISL_2464982, EPI_ISL_2465025	CH. ROBERT BALLANGER	Department of Virology, Henri Mondor University Hospital, Assistance Publique Hôpitaux de Paris, Université Paris-Est Créteil, INSERM U955	Alexandre Soulier; Christophe Rodriguez; Elisabeth Trawinski; Guillaume Gricourt; Jean-Michel Pawlotsky; Melissa N'Debi; Slim Fourati; Vanessa Demontant
EPI_ISL_2878377	CH.INTERCOMMUNAL DE CRETEIL	Department of Virology, Henri Mondor University Hospital, Assistance Publique Hôpitaux de Paris, Université Paris-Est Créteil, INSERM U955	Alexandre Soulier; Christophe Rodriguez; Elisabeth Trawinski; Guillaume Gricourt; Jean-Michel Pawlotsky; Melissa N'Debi; Slim Fourati; Vanessa Demontant
EPI_ISL_2248799, EPI_ISL_2248815, EPI_ISL_2248821, EPI_ISL_2248831, EPI_ISL_2248833, EPI_ISL_2248834, EPI_ISL_2362089, EPI_ISL_2362091, EPI_ISL_2362092, EPI_ISL_2362096, EPI_ISL_2362097, EPI_ISL_2362100, EPI_ISL_2362102, EPI_ISL_2362103, EPI_ISL_2362106, EPI_ISL_2362115, EPI_ISL_2362117, EPI_ISL_2362118, EPI_ISL_2376421, EPI_ISL_2536049, EPI_ISL_2536050, EPI_ISL_2536052, EPI_ISL_2536058, EPI_ISL_2536059, EPI_ISL_2536060, EPI_ISL_2536066, EPI_ISL_2536067, EPI_ISL_2536068, EPI_ISL_2536075, EPI_ISL_2694930, EPI_ISL_2694932			
see above	CHLC	Instituto Nacional de Saude (INSA)	Borges et al
EPI_ISL_2536083	CHLN	Instituto Nacional de Saude (INSA)	Borges et al
EPI_ISL_2340136, EPI_ISL_2340137, EPI_ISL_2340138	CHMT	Instituto Nacional de Saude (INSA)	Borges et al
EPI_ISL_2090827	CHOR	UMR PIMIT	Dr Camille Lebarbenchon; Dr David A Wilkinson; Dr Patrick Mavingui; Magali Turpin
EPI_ISL_2695296	CHTMAD	Instituto Nacional de Saude (INSA)	Borges et al
EPI_ISL_2791309	CHU DE DIJON	Department of Virology, Henri Mondor University Hospital, Assistance Publique Hôpitaux de Paris, Université Paris-Est Créteil, INSERM U955	Alexandre Soulier; Christophe Rodriguez; Elisabeth Trawinski; Guillaume Gricourt; Jean-Michel Pawlotsky; Melissa N'Debi; Slim Fourati; Vanessa Demontant
EPI_ISL_2143486, EPI_ISL_2444363	CHU LILLE	CHU Lille - Laboratoire de Virologie	AIT YAHYA Emilie; ALIDJINOU Enagnon Kazali; BOCKET Laurence; CREPIN Michel; DEMAY Christophe; ENGELMANN Ilka; GEFFROY Sandrine; GUIGON Aurélie; LAMBERT Valérie; LAZREK Mouna; NOBILLIAUX Florian; PREVOST Brigitte; THUILLIER Caroline; TINEZ Claire
EPI_ISL_2226348	CHU POITIERS	CHU Poitiers	Agnes BEBY-DEFAUX; Birama N'DIAYE; Caroline MICHAUD; Magali GARCIA; Manon PRAT; Maxime PICHON; Nicolas LEVEQUE; Valentin BON-BARET
EPI_ISL_2441808	CHU de la Réunion - Nord	Laboratoire de virologie, CNR arbovirus Associé, Chu de la Réunion	Etienne Frumence; Marie-Christine Jaffar Bandjee; Nicolas Traversier; Sabrina Petit Genet
EPI_ISL_2603421	CHUGA-IBP-laboratoire de Virologie	IBP-laboratoire de virologie	Alban Caporossi; Anne Signori -Schmuck; Anne-Karen Faure; Aurélie Truffot; Benjamin Nemoz; Hugo Jardin; Julien Andréani; Julien Lupo; Léa Ponderand; Pascal Poignard; Patrice Morand; Raphaële Germi; Sylvie Larrat
EPI_ISL_2695093, EPI_ISL_2695103, EPI_ISL_2695123	CHULN - H Santa Maria	Instituto Nacional de Saude (INSA)	Borges et al
EPI_ISL_2102154, EPI_ISL_2622101, EPI_ISL_2686816, EPI_ISL_2820533, EPI_ISL_2820536, EPI_ISL_2868568, EPI_ISL_2868587, EPI_ISL_2868601, EPI_ISL_2868603, EPI_ISL_2868606, EPI_ISL_2868608, EPI_ISL_2868614			
see above	CHUV	Laboratory of genomics and metagenomics	Claire Bertelli; Damien Jacot; Gilbert Greub; Sébastien Aeby; Trestan Pillonel
EPI_ISL_2017662, EPI_ISL_2017663, EPI_ISL_2637264, EPI_ISL_2637366	CLILAB	Microbiology Department	Aida Gonzalez-Diaz; Carmen Ardanuy; Jordi Camara; Jordi Niubó; Laura Calatayud; M Angeles Domínguez; Miguel Fernandez-Huerta; Sara Marti
EPI_ISL_2629572, EPI_ISL_2676802	CNR Virus des Infections Respiratoires - France SUD	CNR Virus des Infections Respiratoires - France SUD	Antonin Bal; Bruno Lina; Gregory Destras; Gwendolyne Burfin; Hadrien Regue; Laurence Josset; Martine Valette; Quentin Semanas
EPI_ISL_2227272	COVID-19 National Reference Laboratory, Pasteur Institute of Iran	Genetics Research Center, University of Social Welfare and Rehabilitation Sciences	Hossein Najmabadi.; Kimia Kahrizi; Mahsa Tavakoli; Marzieh Mohseni; Mohammad Hassan Pouriayevali; Mostafa Salehi-Vaziri; Tahmineh Jalali; Zohreh Fattahi
EPI_ISL_2227271	COVID-19 National Reference Laboratory, Pasteur Institute of Iran.	Genetics Research Center, University of Social Welfare and Rehabilitation Sciences	Hossein Najmabadi.; Kimia Kahrizi; Mahsa Tavakoli; Marzieh Mohseni; Mohammad Hassan Pouriayevali; Mostafa Salehi-Vaziri; Tahmineh Jalali; Zohreh Fattahi
EPI_ISL_2227204, EPI_ISL_2227268, EPI_ISL_2227269	COVID-19 National Reference Laboratoty, Pasteur Institute of Iran	Genetics Research Center, University of Social Welfare and Rehabilitation Sciences	Hossein Najmabadi.; Kimia Kahrizi; Mahsa Tavakoli; Marzieh Mohseni; Mohammad Hassan Pouriayevali; Mostafa Salehi-Vaziri; Tahmineh Jalali; Zohreh Fattahi
EPI_ISL_2227270	COVID-19 National Reference Laboratoty, Pasteur Institute of Iran.	Genetics Research Center, University of Social Welfare and Rehabilitation Sciences	Hossein Najmabadi.; Kimia Kahrizi; Mahsa Tavakoli; Marzieh Mohseni; Mohammad Hassan Pouriayevali; Mostafa Salehi-Vaziri; Tahmineh Jalali; Zohreh Fattahi
EPI_ISL_2156846, EPI_ISL_2373412, EPI_ISL_2373492, EPI_ISL_2661097, EPI_ISL_2775023, EPI_ISL_2775030, EPI_ISL_2775075, EPI_ISL_2775084, EPI_ISL_2775091, EPI_ISL_2775096, EPI_ISL_2775099, EPI_ISL_2775105, EPI_ISL_2775251			
see above	CSIR-Centre for Cellular and Molecular Biology	CSIR-Centre for Cellular and Molecular Biology-INSACOG	Amareshwar Vodapalli; Ara Sreenivas; Archana Bharadwaj Siva; B Himasri; Blessy B John; Divya Tej Sowpati; Jandhyala Sai Krishna; Karthik Bharadwaj Tallapaka; Lamuk Zaveri; Onkar Kulkarni; Payel Mukherjee; Priya Nurkurthy; Rakesh K Mishra; Sharath Chandra Thota; Shreekant Verma; Sofia Banu; Sumedha Avadhanula; Tulasi Nagabandi; Valli Nagalakshmi Undamatla; Vidhyadhari Methuku; Viswagithe S L
EPI_ISL_2441372	CSIR-National Environmental Engineering Research Institute	CSIR-Centre for Cellular and Molecular Biology-INSACOG	Amareshwar Vodapalli; Ara Sreenivas; Archana Bharadwaj Siva; B Himasri; Divya Tej Sowpati; Karthik Bharadwaj Tallapaka; Krishna Khairnar; Lamuk Zaveri; Onkar Kulkarni; Payel Mukherjee; Priya Nurkurthy; Rakesh K Mishra; Shreekant Verma; Sofia Banu; Sumedha Avadhanula; Tulasi Nagabandi; Valli Nagalakshmi Undamatla; Vidhyadhari Methuku
EPI_ISL_2842815, EPI_ISL_2892494	California Department of Public Health	California Department of Public Health	CDPH IDLB COVIDNet et al
EPI_ISL_2838017	California Department of Public Health Valencia Branch Laboratory (CDPH VBL)	California Department of Public Health	CDPH-COVIDNet
EPI_ISL_2652214, EPI_ISL_2652216, EPI_ISL_2652217, EPI_ISL_2727568, EPI_ISL_2774008, EPI_ISL_2774010	Cantacuzino National Military-Medical Institute, Viral Respiratory Infections Laboratory	Cantacuzino Institute Virology	Carmen Cherciu; Luiza Ustea; Mihaela Lazar; Mihaela Oprea; Nicoleta Paraschiv; Sorin Dinu
EPI_ISL_2365706	Cell-T	1. National Institute of Public Health - National Institute of Hygiene, Warsaw, Poland 2. Biobank Lab, University of Lodz 3. Laboratory of Respiratory Viruses, Teaching and Clinical Center of the Medical University of Lodz	Dominik Strapagiel; Izabela Dróżdż; Jakub Lach; Katarzyna Zacharczuk; Klaudyna Królikowska; Maciej Borowiec; Magdalena Nowakowska; Magdalena Traczyk-Borszyńska; Marcin Słomka; Marta Sobalska-Kwapis; Małgorzata Sadkowska-Todys; Tomasz Płoszaj; Tomasz Wołkowicz
EPI_ISL_2450030, EPI_ISL_2574058, EPI_ISL_2574080, EPI_ISL_2671456	Center for Laboratory Medicine	Center for Laboratory Medicine	Yannick Gerth
EPI_ISL_2887701	Center for Virology, Medical University of Vienna	Bergthaler laboratory, CeMM Research Center for Molecular Medicine of the Austrian Academy of Sciences	Andreas Bergthaler; Anna Schedl; Bekir Erguner; Benedikt Agerer; Christoph Bock; Fabian Amman; Jan Laine; Lukas Endler; Maelle Le Moing; Martin Senekowitsch; Matthew Thornton; Michael Schuster; Petr Triska; Thomas Penz
EPI_ISL_2705575	Centers for Disease Control, R.O.C. (Taiwan)	Centers for Disease Control, R.O.C. (Taiwan)	Ji-Rong Yang; Jung-Jung Mu; Ming-Tsan-Liu; Yu-Chi-Lin
EPI_ISL_1843777, EPI_ISL_1843874, EPI_ISL_2123187, EPI_ISL_2123202, EPI_ISL_2123266	Centogene; Dr. Bauer Laboratoriums GmbH	Robert Koch Institute	
EPI_ISL_2834925, EPI_ISL_2834927	Central Health Laboratory/Airport Health Laboratory	Central Health Laboratory ,Victoria Hospital, Candos,Ministry of Health and Wellness, Mauritius	Bahadoor BS; Jannoo N; Manraj SS; Mathur H; Patoo M; Ramuth M; Sonoo J; Sujeewon C
EPI_ISL_1827727	Centralny Szpital Kliniczny MSWiA w Warszawie	1. Virogenetics Laboratory of Virology, Małopolska Centre of Biotechnology, Jagiellonian University. 2. Intercollegiate Faculty of Biotechnology University of Gdansk and Medical University of Gdansk	Adam Sybilski; Anna Meler; Krystyna Bienkowska-Szewczyk; Krzysztof Pyrc; Lukasz Rabalski; Maciej Kosinski; Michał Hampel; Natalia Mazur-Panasiuk; Sławomir Butkiewicz
EPI_ISL_2002644	Centre Hospitalier Universitaire Clermont-Ferrand	CHU Clermont-Ferrand, service de virologie	Bisseux Maxime; Combes Patricia; Henquell Cécile; Mirand Audrey
EPI_ISL_2671484	Centro de Investigacion Biomedica de Occidente (CIBO)	Unidad de Genomica Avanzada	; Alejandra Garcia-Gasca; Alejandra Hernandez-Teran; Alejandro Sanchez-Flores; Alfredo Herrera-Estrella; Alicia Ocaña-Mondragon; Andreu Comas-Garcia; Angel Gustavo Salas-Lais; Antonio Loza Roman; Bernardo Martinez-Miguel; Blanca Taboada; Brenda Irasema Maldonado-Meza; Bruno Gomez-Gil; Carla Ivon Herrera-Najera; Carlos F. Arias; Celia Boukadida; Celida Duque Molina; Celida Martinez- Rodriguez; Clara Esperanza Santacruz-Tinoco; Concepcion Grajales-Muñiz; Consorcio Mexicano de Vigilancia Genomica (CoViGen-Mex). Authors (in alphabetical order): Julio Elias Alvarado-Yaah; Cristobal Chaidez-Quiroz; Daniel Fregoso-Rueda; Daniel Lira Morales; Eduardo Becerril-Vargas; Fernando Fontove-Herrera; Fidencio Mejia-Nepomuceno;

			Francisco Pulido; Gloria Elena Espinosa-Ayala; Gloria Maria Molina-Salinas; Gloria Vazquez; Hector Esteban Paz-Juarez; Hector Montoya-Fuentes; Helen Haydee Fernanda Ramirez-Plascencia; Irvin Gonzalez-Lopez; Jean Pierre Gonzalez; Jesus Hernandez; Joel Armando Vazquez-Perez.; Jorge Salas-Hernandez; Jose Antonio Enciso-Moreno; Jose Arturo Martinez-Orozco; Jose Esteban Muñoz-Medina; Jose de Jesus Nuñez-Contreras; Juan Bautista Chale-Dzul; Julissa Enciso-Ibarra; Luis Alberto Ochoa-Carrera; Margarita Matias-Florentino; Maria Guadalupe Santiago-Mauricio; Maria Guadalupe de Jesus Mireles-Rivera; Mario Mujica-Sanchez; Marissa Perez-Garcia; Nelly Selem-Mojica; Pavel Isa; Ricardo Ciria Merce; Ricardo Grande; Rosa Maria Gutierrez Rios; Santiago avila-Rios; Selene Zarate; Susana Lopez; Veronica Mata-Haro; Victor Eduardo Garcia-Arias; Victor Hugo Borja-Aburto
EPI_ISL_2671635, EPI_ISL_2671636	Centro de Investigacion Biomedica del Noreste (CIBIN)	Unidad de Genomica Avanzada	; Alejandra Garcia-Gasca; Alejandra Hernandez-Teran; Alejandro Sanchez-Flores; Alfredo Herrera-Estrella; Alicia Ocaña-Mondragon; Andreu Comas-Garcia; Angel Gustavo Salas-Lais; Antonio Loza Roman; Bernardo Martinez-Miguel; Blanca Taboada; Brenda Irasema Maldonado-Meza; Bruno Gomez-Gil; Carla Ivon Herrera-Najera; Carlos F. Arias; Celia Boukadida; Celida Duque Molina; Celida Martinez- Rodriguez; Clara Esperanza Santacruz-Tinoco; Concepcion Grajales-Muñiz; Consorcio Mexicano de Vigilancia Genomica (CoViGen-Mex). Authors (in alphabetical order): Julio Elias Alvarado-Yaah; Cristobal Chaidez-Quiroz; Daniel Fregoso-Rueda; Daniel Lira Morales; Eduardo Becerril-Vargas; Fernando Fontove-Herrera; Fidencio Mejia-Nepomuceno; Francisco Pulido; Gloria Elena Espinosa-Ayala; Gloria Maria Molina-Salinas; Gloria Vazquez; Hector Esteban Paz-Juarez; Hector Montoya-Fuentes; Helen Haydee Fernanda Ramirez-Plascencia; Irvin Gonzalez-Lopez; Jean Pierre Gonzalez; Jesus Hernandez; Joel Armando Vazquez-Perez.; Jorge Salas-Hernandez; Jose Antonio Enciso-Moreno; Jose Arturo Martinez-Orozco; Jose Esteban Muñoz-Medina; Jose de Jesus Nuñez-Contreras; Juan Bautista Chale-Dzul; Julissa Enciso-Ibarra; Luis Alberto Ochoa-Carrera; Margarita Matias-Florentino; Maria Guadalupe Santiago-Mauricio; Maria Guadalupe de Jesus Mireles-Rivera; Mario Mujica-Sanchez; Marissa Perez-Garcia; Nelly Selem-Mojica; Pavel Isa; Ricardo Ciria Merce; Ricardo Grande; Rosa Maria Gutierrez Rios; Santiago avila-Rios; Selene Zarate; Susana Lopez; Veronica Mata-Haro; Victor Eduardo Garcia-Arias; Victor Hugo Borja-Aburto
EPI_ISL_2798753, EPI_ISL_2798754, EPI_ISL_2798755, EPI_ISL_2799000, EPI_ISL_2799099, EPI_ISL_2799114, EPI_ISL_2799137, EPI_ISL_2799152, EPI_ISL_2799171, EPI_ISL_2799172, EPI_ISL_2799198, EPI_ISL_2799200, EPI_ISL_2799206, EPI_ISL_2799215, EPI_ISL_2799230, EPI_ISL_2799236, EPI_ISL_2799240			
see above	Centrālā Laboratorija, SIA	Riga East University Hospital, National Microbiology Reference Laboratory; Eurofins Genomics Europe Sequencing GmbH	Arzu Algulieva; Diāna Dušacka; Dārta Pūpola; Ilva Pole; Jana Osīte; Jevgenijs Bodrenko; Jūlija Čevere; Reinis Vangravs; Reinis Zeltmatis; Sergejs Ņikišins; Stella Lapiņa; Ģirts Šķenders
EPI_ISL_2228858, EPI_ISL_2464723, EPI_ISL_2795744, EPI_ISL_2795874	Cerballiance Paris IDF est Saint-Denis	Department of Virology, Henri Mondor University Hospital, Assistance Publique Hôpitaux de Paris, Université Paris-Est Créteil, INSERM U955	Alexandre Soulier; Christophe Rodriguez; Elisabeth Trawinski; Guillaume Gricourt; Jean-Michel Pawlotsky; Melissa N'Debi; Slim Fourati; Vanessa Demontant
EPI_ISL_2646234, EPI_ISL_2646238, EPI_ISL_2646262, EPI_ISL_2646269, EPI_ISL_2652114, EPI_ISL_2652115	Cerballiance-IDF	Cerba lab	Aude Lessenne; Bénédicte Roquebert; Emmanuel Lecorche; Kader Merah; Laura Verdurme; Patrice Herisson; Sabine Trombert-Paolantoni; Stéphanie Haïm-Boukobza; Thierry Collin
EPI_ISL_2646255, EPI_ISL_2646258, EPI_ISL_2646261, EPI_ISL_2646278	Cerballiance-Paris	Cerba lab	Aude Lessenne; Bénédicte Roquebert; Emmanuel Lecorche; Kader Merah; Laura Verdurme; Patrice Herisson; Sabine Trombert-Paolantoni; Stéphanie Haïm-Boukobza; Thierry Collin
EPI_ISL_2646274, EPI_ISL_2646277	Cerballiance-Provence	Cerba lab	Aude Lessenne; Bénédicte Roquebert; Emmanuel Lecorche; Kader Merah; Laura Verdurme; Patrice Herisson; Sabine Trombert-Paolantoni; Stéphanie Haïm-Boukobza; Thierry Collin
EPI_ISL_2646253	Cerballiance-Pyrénées	Cerba lab	Aude Lessenne; Bénédicte Roquebert; Emmanuel Lecorche; Kader Merah; Laura Verdurme; Patrice Herisson; Sabine Trombert-Paolantoni; Stéphanie Haïm-Boukobza; Thierry Collin
EPI_ISL_2337257	Chiba Prefectural Institute of Public Health	Pathogen Genomics Center, National Institute of Infectious Diseases	Kentaro Itokawa; Makoto Kuroda; Masanori Hashino; Rina Tanaka; Tsuyoshi Sekizuka
EPI_ISL_1970347	Chongqing Yuzhong District Center For Disease Control And Prevention	Chongqing Municipal Center for Disease Control and Prevention	Hua Ling; Jiaqi Li; Mingyue Wang; Rong Rong; Sheng Ye; Shuang Chen; Wenge Tang; Yingbing Zhou; Yun Tang; Yuyue Luo; Zhangping Tan; Zhen Yu
EPI_ISL_2433619, EPI_ISL_2433620, EPI_ISL_2433621, EPI_ISL_2433622, EPI_ISL_2433623, EPI_ISL_2433624, EPI_ISL_2433625, EPI_ISL_2433626, EPI_ISL_2433627, EPI_ISL_2433628, EPI_ISL_2433629, EPI_ISL_2433630, EPI_ISL_2433631, EPI_ISL_2433632, EPI_ISL_2433633, EPI_ISL_2433634, EPI_ISL_2433635, EPI_ISL_2433636, EPI_ISL_2433637, EPI_ISL_2433638, EPI_ISL_2433639, EPI_ISL_2433640, EPI_ISL_2433641, EPI_ISL_2433642, EPI_ISL_2433643, EPI_ISL_2433644, EPI_ISL_2433645			
see above	Chulabhorn Hospital	Division of Genomic Medicine and Innovation support,Department of Medical Sciences, Ministry of Public Health, Thailand	Archawin Rojanawiwat; Jirapha Pakdee; Natthakul Bunneang; Nuanjun Wichukchinda; Penpitcha Thawong; Pilailuk Akkapaiboon Okada; Pundharika Piboonsiri; Surakameth Mahasirimongkol; Waritta Sawaengdee
EPI_ISL_2840598, EPI_ISL_2840599, EPI_ISL_2840600, EPI_ISL_2877030, EPI_ISL_2877031	Chulabhorn Hospital	National Institute of Health, Department of Medical Sciences, Ministry of Public Health, Thailand	; Natchaya Khiadsang; Natchaya Khiadsang; Nuttida Thongpramul; Pakorn Piromtong; Pilailuk Okada; Ratana Tacharoenmuang; Siripaporn Phuygun; Sittiporn Parnmen; Sunthareeya Waicharoen; Thanutsapa Thanadachakul; Warawan Wongboot; sirikanda wimol
EPI_ISL_2450544, EPI_ISL_2450554, EPI_ISL_2450563, EPI_ISL_2450565, EPI_ISL_2523808, EPI_ISL_2523821, EPI_ISL_2523836			
see above	City Hospital No 40	WHO National Influenza Centre Russian Federation	Andrey Komissarov; Artem Fadeev; Daria Danilenko; Dmitry Lioznov; Elena Nabieva; Georgii Bazykin; Kirill Varchenko; Ksenia Safina; Kseniya Komissarova; Maria Pisareva; Maria Timofeeva; Mikhail Bakaev; Nikita Yolshin; Olga Shneider; Oula Mansour; Sergey Scherbak; Tamila Musaeva; Veronika Eder
EPI_ISL_2264591	Clemenshospital - Alexianer Clemenshospital Münster	Robert Koch Institute	
EPI_ISL_2652215	Clinical Emergency County Hospital, Craiova	Cantacuzino Institute Virology	Carmen Cherciu; Luiza Ustea; Mihaela Lazar; Mihaela Oprea; Nicoleta Paraschiv; Sorin Dinu
EPI_ISL_2100243, EPI_ISL_2100244, EPI_ISL_2100245, EPI_ISL_2100246, EPI_ISL_2100247	Clinical Hospital of Infectious and Tropical Diseases “Dr. Victor Babes”	Cantacuzino Institute Virology	Carmen Cherciu; Mihaela Lazar; Mihaela Oprea; Sorin Dinu
EPI_ISL_2835648, EPI_ISL_2835685	Clinical Microbiology Laboratory, Faculty of Medicine, Universitas Indonesia	Faculty of Medicine, Universitas Indonesia	Andi Yasmon; Anis Karuniawati; Ari Fahrial Syam; Badriul Hegar; Beti Ernawati; Budi Wiweko; Fadilah; Fera Ibrahim; Fithriyah; Hana Apsari Pawestri; Khaerunissa Anbar Istiadi; Linda Erlina; Pratiwi Sudarmono; Rafika Indah Paramita; Vivi Setiawaty; Yulia Rosa
EPI_ISL_1833679, EPI_ISL_1993840, EPI_ISL_1993870, EPI_ISL_1993872, EPI_ISL_1993906, EPI_ISL_2367243, EPI_ISL_2408445, EPI_ISL_2466573, EPI_ISL_2610961, EPI_ISL_2610993, EPI_ISL_2611003, EPI_ISL_2652262			
see above	Clinical Virology	Clinical Bacteriology	Adrian Egli; Alfredo Mari; Fanny Wegner; Hans Hirsch; Helena MB Seth-Smith; Julia Bielicki; Karoline Leuzinger; Madlen Stange; Manuel Battegay; Tim Roloff
EPI_ISL_2427389, EPI_ISL_2492438, EPI_ISL_2638046, EPI_ISL_2885681	Clinique Saint-Pierre Ottignies	UCLouvain/IREC/MBLG	Benoit Kabamba Mukadi; Bertrand Bearzatto; Jean Ruelle; Ophélie Simon
EPI_ISL_1935601, EPI_ISL_2427353, EPI_ISL_2492431, EPI_ISL_2492433, EPI_ISL_2492436, EPI_ISL_2626021, EPI_ISL_2626037, EPI_ISL_2778026, EPI_ISL_2778040, EPI_ISL_2778041, EPI_ISL_2778047, EPI_ISL_2885626, EPI_ISL_2885638, EPI_ISL_2885663			
see above	Cliniques universitaires Saint-Luc	UCLouvain/IREC/MBLG	Benoit Kabamba Mukadi; Bertrand Bearzatto; Eleonore Ngyuvula; Jean Ruelle; Ophélie Simon
EPI_ISL_2469771, EPI_ISL_2469774, EPI_ISL_2469779	Corona-Testzentrum ifp Institut für Produktqualität GmbH	Robert Koch Institute	
EPI_ISL_2507326, EPI_ISL_2839783	Curative Labs	Curative Labs	Elias L. Salfati; Eugenia Khorosheva; George Way; J.Cesar Ignacio-Espinoza; Janet Chen; Mikhail Hanewich-Hollatz; Nabjot Sandhu; Sophia Quasem; Vladimir Slepnev; Zhiyi Xie
EPI_ISL_2868918	DINKES Kab Bogor	National Institute of Health Research and Development	Arie Ardiansyah Nugraha; Hana Apsari Pawestri; Hartanti Dian Ikawati; Kartika Dewi Puspa; Krisna Pangesti; Nelly Puspandari; Subangkit; Triyani Soekarso; Vivi Setiawaty
EPI_ISL_2845758	DKMS Life Science Lab GmbH Dresden	Robert Koch Institute	
EPI_ISL_1337507	DOHMH Corona	New York City Public Health Laboratory	Jade Wang; et al.
EPI_ISL_2728324	DSU HASSAN	INSACOG-KA, NIMHANS	Ananthapadmanabha Kotambail; Anita S Desai; Anson Kunjumon George; Chetan G K; Chitra Pattabiraman; Darshan Sreenivas; Ellango Ramasamy; Gautham Arunachal Udupi; Mahesh Kumar.C.S; Sony Sharma; V Ravi
EPI_ISL_2482761, EPI_ISL_2893793	DYOMEDEA-LABORATOIRE DE LA SAUVEGARDE	CNR Virus des Infections Respiratoires - France SUD	Antonin Bal; Bruno Lina; Gregory Destras; Gwendolyne Burfin; Hadrien Regue; Laurence Josset; Martine Valette; Quentin Semanas
EPI_ISL_2319006, EPI_ISL_2319007	Department for Virology, Molecular Biology and Genome Research, R. G. Lugar Center for Public Health Research, National Center for Disease Control and Public Health (NCDC) of Georgia.	Department for Virology, Molecular Biology and Genome Research, R. G. Lugar Center for Public Health Research, National Center for Disease Control and Public Health (NCDC) of Georgia.	Adam Kotorashvili; Amiran Gamkrelidze.; Ana Papkiauri; Ann Machablishvili; Anna Kasradze; Davit Tsaguria; Ekaterine Khmaladze; Ekaterine Zangaladze; Ekaterine Zhghenti; Giorgi Gogoladze; Giorgi Tomashvili; Gvantsa Brachveli; Gvantsa Chanturia; Irma Burjanadze; Ketevan Sidamonidze; Khatuna Zakhashvili; Lela Sabadze; Lela Urushadze; Magda Dgebuadze; Maia Alkhazashvili; Mari Gavashelidze; Mariam Zakalashvili; Marine Murtskhvaladze; Meri Pantsulaia; Nato Kotaria; Nino Berishvili; Paata Imnadze; Roena Sukhiashvili; Tamar Jashiashvili; Tata Imnadze; Tea Tevdoradze
EPI_ISL_2266083	Department für Labormedizin Abteilung III Bereich Molekulare Diagnostik Universitätsklinikum Halle(Saale)	Robert Koch Institute	
EPI_ISL_2723562, EPI_ISL_2723563, EPI_ISL_2723564, EPI_ISL_2834002, EPI_ISL_2834003, EPI_ISL_2834004, EPI_ISL_2840854, EPI_ISL_2840855, EPI_ISL_2840856, EPI_ISL_2840857, EPI_ISL_2840858, EPI_ISL_2840859, EPI_ISL_2840860, EPI_ISL_2863929, EPI_ISL_2863930, EPI_ISL_2863931, EPI_ISL_2863932, EPI_ISL_2863933, EPI_ISL_2863934, EPI_ISL_2863935, EPI_ISL_2893772, EPI_ISL_2893773, EPI_ISL_2893774, EPI_ISL_2893775, EPI_ISL_2893776, EPI_ISL_2893777, EPI_ISL_2893778, EPI_ISL_2896214, EPI_ISL_2896215, EPI_ISL_2896216, EPI_ISL_2896217, EPI_ISL_2896218, EPI_ISL_2896219, EPI_ISL_2896220, EPI_ISL_2896221, EPI_ISL_2896222, EPI_ISL_2896224, EPI_ISL_2896226, EPI_ISL_2896227, EPI_ISL_2896228, EPI_ISL_2896229, EPI_ISL_2896230, EPI_ISL_2896231			
see above	Department of Acute Infectious Diseases Control and Prevention, Yunnan Provincial Center for Disease Control and Prevention	Department of Acute Infectious Diseases Control and Prevention, Yunnan Provincial Center for Disease Control and Prevention	Jienan Zhou; Meiling Zhang; Senquan Jia; Xiaoqing Fu; Zhaosheng Liu
EPI_ISL_2430149, EPI_ISL_2430527, EPI_ISL_2665415, EPI_ISL_2665480, EPI_ISL_2665812, EPI_ISL_2666132, EPI_ISL_2666159, EPI_ISL_2666270, EPI_ISL_2666451, EPI_ISL_2666493, EPI_ISL_2666760, EPI_ISL_2666994, EPI_ISL_2667192, EPI_ISL_2667226, EPI_ISL_2667325, EPI_ISL_2667354, EPI_ISL_2667612, EPI_ISL_2667628, EPI_ISL_2667724, EPI_ISL_2667866, EPI_ISL_2668073, EPI_ISL_2668151, EPI_ISL_2668182, EPI_ISL_2668506, EPI_ISL_2668554, EPI_ISL_2668655, EPI_ISL_2668703, EPI_ISL_2668734, EPI_ISL_2668747, EPI_ISL_2668807, EPI_ISL_2668914, EPI_ISL_2668987, EPI_ISL_2669320, EPI_ISL_2669478, EPI_ISL_2669604, EPI_ISL_2669906, EPI_ISL_2670090, EPI_ISL_2670207, EPI_ISL_2670242, EPI_ISL_2670481, EPI_ISL_2670501, EPI_ISL_2670547, EPI_ISL_2670854, EPI_ISL_2670887, EPI_ISL_2670908, EPI_ISL_2671046, EPI_ISL_2671244, EPI_ISL_2675405, EPI_ISL_2675428, EPI_ISL_2675638, EPI_ISL_2675739, EPI_ISL_2680325, EPI_ISL_2680526, EPI_ISL_2680722, EPI_ISL_2684111, EPI_ISL_2744044, EPI_ISL_2747550, EPI_ISL_2747658, EPI_ISL_2747811, EPI_ISL_2747821, EPI_ISL_2747830, EPI_ISL_2748032, EPI_ISL_2748121, EPI_ISL_2748123, EPI_ISL_2748194, EPI_ISL_2748256, EPI_ISL_2748313, EPI_ISL_2784375, EPI_ISL_2784378, EPI_ISL_2784380, EPI_ISL_2784435, EPI_ISL_2784440, EPI_ISL_2784501, EPI_ISL_2784522, EPI_ISL_2784525, EPI_ISL_2784558, EPI_ISL_2784666, EPI_ISL_2819069, EPI_ISL_2819075, EPI_ISL_2819128, EPI_ISL_2819304, EPI_ISL_2819324, EPI_ISL_2819366, EPI_ISL_2819370, EPI_ISL_2819387, EPI_ISL_2819391, EPI_ISL_2819412, EPI_ISL_2819417, EPI_ISL_2819450, EPI_ISL_2819557, EPI_ISL_2819606, EPI_ISL_2819658, EPI_ISL_2838151, EPI_ISL_2838165, EPI_ISL_2838181, EPI_ISL_2838188, EPI_ISL_2838206, EPI_ISL_2838229, EPI_ISL_2838249, EPI_ISL_2838262, EPI_ISL_2838268, EPI_ISL_2838274, EPI_ISL_2838308, EPI_ISL_2838309, EPI_ISL_2838352, EPI_ISL_2854301, EPI_ISL_2854409, EPI_ISL_2854433, EPI_ISL_2854464, EPI_ISL_2854470, EPI_ISL_2854549, EPI_ISL_2854591, EPI_ISL_2854645, EPI_ISL_2862662, EPI_ISL_2862747, EPI_ISL_2863004, EPI_ISL_2863081, EPI_ISL_2863154, EPI_ISL_2863194, EPI_ISL_2863539, EPI_ISL_2863737			
see above	Department of Bacteria, Parasites and Fungi, Statens Serum Institut, Copenhagen, Denmark	Statens Serum Institut Bioinformatics and Microbial Genomics	Danish Covid-19 Genome Consortium

EPI_ISL_2003343	Department of Clinical Microbiology	GIGA Medical Genomics	Bouchra Boujemla; Cécile Meex; Keith Durkin; Maria Artesi; Marie-Pierre Hayette; Nathalie Renotte; Pierrette Melin; Raphaël Boreux; Sébastien Bontems; Vincent Bours
EPI_ISL_1919364, EPI_ISL_2724975	Department of Medical Microbiology - section Molde, Molde Hospital	Norwegian Institute of Public Health, Department of Virology	Atiya R Ali; Debech Nadia; Engebretsen Serina Beate; Garcia Llorente Ignacio; Hilde Elshaug; Hilde Vollan; Jon Bråte; Kamilla Heddeland Instefjord; Karoline Bragstad; Kathrine Stene-Johansen; Line Victoria Moen; Marie Paulsen Madsen; Olav Hungnes; Pedersen Benedikte Nevjen; Rasmus Riis Kopperud
EPI_ISL_2833646	Department of Microbiology, National Institute for Public Health of Kosova	Charité Universitätsmedizin Berlin, Institut für Virologie	Aferdita Hyseni; Barbara Mühlemann; Blendi Jerliu; Christian Drosten; Donjeta Hajdari; Julia Schneider; Jörn Beheim-Schwarzbach; Nazmi Mehmeti; Pranvera Abazi; Talitha Veith; Terry Jones; Victor M Corman; Xhevat Jakupi; Zana Deva
EPI_ISL_2101020	Department of Microbiology, Tochigi Prefectural Institute of Public Health and Enviromental Science	Genome Analysis Center, Kamma Memorial Hospital	Fuminori Mizukoshi; Hanako Yazawa; Hiroshi Kamma; Jun Ishii; Kaori Watanabe; Michiaki Masuda; Satoko Soma; Takuya Yazawa; Yoshiko Hitomi
EPI_ISL_2551520	Department of Microbiology, Tochigi Prefectural Institute of Public Health and Environmental Science	Genome Analysis Center, Kamma Memorial Hospital	Fuminori Mizukoshi; Hanako Yazawa; Hiroshi Kamma; Jun Ishii; Kaori Watanabe; Michiaki Masuda; Satoko Soma; Takuya Yazawa; Yoshiko Hitomi
EPI_ISL_2637224	Department of Microbiology, Yamanashi Institute for Public Health and Environment	Genome Analysis Center, Yamanashi Central Hospital	Yosuke Hirotsu
EPI_ISL_2722210	Department of Pathology, University of Cambridge	COVID-19 Genomics UK (COG-UK) Consortium	Aminu S. Jahun; Ian Goodfellow; Iliana Georgana; Martin D. Curran; Myra Hosmillo; Rhys Izuagbe; Surendra Parmar; William L. Hamilton; Yasmin Chaudhry
EPI_ISL_2099869, EPI_ISL_2190101, EPI_ISL_2687994, EPI_ISL_2687997	Department of Public Health Bucharest	National Institute of Infectious Diseases-Prof. Dr. Matei Bals Molecular Diagnostics Laboratory	Andreea Tudor; Corina Casangiu; Dan Otelea; Leontina Banica; Marius Surleac; Ovidiu Vlaicu; Petre Milu; Robert Hohan; Simona Paraschiv
EPI_ISL_2434174, EPI_ISL_2434982, EPI_ISL_2438666, EPI_ISL_2757734, EPI_ISL_2757736, EPI_ISL_2757737, EPI_ISL_2757738, EPI_ISL_2757739, EPI_ISL_2757741, EPI_ISL_2757743, EPI_ISL_2757744, EPI_ISL_2757756			
see above	Department of Virology	Department of Virology	Aamer Ikram; Massab Umair; Muhammad Ammar; Muhammad Salman; Nazish Badar; Syed Adnan Haider; Zaira Rehman
EPI_ISL_2608773, EPI_ISL_2608787, EPI_ISL_2608788, EPI_ISL_2608789, EPI_ISL_2608815, EPI_ISL_2608823, EPI_ISL_2608834, EPI_ISL_2608879, EPI_ISL_2609354			
see above	Department of Virology and Immunology, University of Helsinki and Helsinki University Hospital, Huslab Finland	Department of Virology, Faculty of Medicine, University of Helsinki, Helsinki, Finland	Essi Korhonen; Hanna Jarva; Hanna Liimatainen; Hannimari Kallio-Kokko; Harri Kangas; Hussein Alburkat; Jenni Virtanen; Maija Lappalainen; Maija Suvanto; Olli Vapalahti; Pekka Ellonen; Phuoc Truong; Ravi Kant; Sari Hannula; Satu Kurkela; Teemu Smura
EPI_ISL_1868349, EPI_ISL_1869673, EPI_ISL_1871129, EPI_ISL_1872528, EPI_ISL_1885762, EPI_ISL_1890934, EPI_ISL_2024848, EPI_ISL_2024994, EPI_ISL_2026084, EPI_ISL_2026527, EPI_ISL_2026828, EPI_ISL_2027848, EPI_ISL_2172542, EPI_ISL_2172683, EPI_ISL_2175543, EPI_ISL_2176057, EPI_ISL_2178114, EPI_ISL_2296934, EPI_ISL_2299973, EPI_ISL_2300683, EPI_ISL_2300806, EPI_ISL_2300839			
see above	Department of Virus and Microbiological Special Diagnostics, Statens Serum Institut, Copenhagen, Denmark	Aalborg University	Danish Covid-19 Genome Consortium
EPI_ISL_2820852	Dept. of Medical Microbiology, Stavanger University Hospital, Helse Stavanger HF	Norwegian Institute of Public Health, Department of Virology	Atiya R Ali; Debech Nadia; Engebretsen Serina Beate; Garcia Llorente Ignacio; Hilde Elshaug; Hilde Vollan; Jon Bråte; Kamilla Heddeland Instefjord; Karoline Bragstad; Kathrine Stene-Johansen; Line Victoria Moen; Marie Paulsen Madsen; Olav Hungnes; Pedersen Benedikte Nevjen; Rasmus Riis Kopperud
EPI_ISL_1994923, EPI_ISL_2358012, EPI_ISL_2438787, EPI_ISL_2438788, EPI_ISL_2438789, EPI_ISL_2438790, EPI_ISL_2438791, EPI_ISL_2438792, EPI_ISL_2438800, EPI_ISL_2772594, EPI_ISL_2772610, EPI_ISL_2772613, EPI_ISL_2772622, EPI_ISL_2772628, EPI_ISL_2772635, EPI_ISL_2772642, EPI_ISL_2772645, EPI_ISL_2772649, EPI_ISL_2828342, EPI_ISL_2828347, EPI_ISL_2828356, EPI_ISL_2828357, EPI_ISL_2828358, EPI_ISL_2828359, EPI_ISL_2828360, EPI_ISL_2828361, EPI_ISL_2828362, EPI_ISL_2828363, EPI_ISL_2828364, EPI_ISL_2828365, EPI_ISL_2828370, EPI_ISL_2828373, EPI_ISL_2828376, EPI_ISL_2828379, EPI_ISL_2828380, EPI_ISL_2868424, EPI_ISL_2868431, EPI_ISL_2868433, EPI_ISL_2868435, EPI_ISL_2868439, EPI_ISL_2868440, EPI_ISL_2868443, EPI_ISL_2868444, EPI_ISL_2868446, EPI_ISL_2868447, EPI_ISL_2868448, EPI_ISL_2868451, EPI_ISL_2868454, EPI_ISL_2868457, EPI_ISL_2868458, EPI_ISL_2886054, EPI_ISL_2886055, EPI_ISL_2886057, EPI_ISL_2886058, EPI_ISL_2886061, EPI_ISL_2886062, EPI_ISL_2886063, EPI_ISL_2886064, EPI_ISL_2886065, EPI_ISL_2886066, EPI_ISL_2886067, EPI_ISL_2886068, EPI_ISL_2886069, EPI_ISL_2886070, EPI_ISL_2886071, EPI_ISL_2886073, EPI_ISL_2886075, EPI_ISL_2886076, EPI_ISL_2886077, EPI_ISL_2886078, EPI_ISL_2886082, EPI_ISL_2886083, EPI_ISL_2886086			
see above	Dept. of Microbiology and Infection Control, Akershus University Hospital HF	Dept. of Microbiology and Infection Control, Akershus University Hospital HF	Alexander Hesselberg Løvestad; Hege Vangstein Aamot
EPI_ISL_2171267	Dhulikhel Hospital, Kathmandu University Hospital	Molecular and Genomics Research Lab, Dhulikhel Hospital, Kathmandu University Hospital	Dipesh Tamrakar; Meghnath Dhimal; Navin Adhikari; Nishan Katuwal; Pradip Gyanwali; Rajeev Shrestha; Saroj Bhattarai; Surendra Kumar Madhup
EPI_ISL_2314888, EPI_ISL_2636270	Diagnosticum - Labor Neukirchen	Robert Koch Institute	
EPI_ISL_2503955, EPI_ISL_2503966	Diagnostyka	1. Academic Center for Pathomorphological and Genetic-Molecular Diagnostics ltd, Bialystok, Poland 2. National Institute of Public Health - National Institute of Hygiene, Warsaw, Poland	Anetta Sulewska; Jacek Nikliński; Janusz Dzięcioł; Joanna Kiśluk; Katarzyna Zacharczuk; Konrad Raczkowski; Magdalena Nowakowska; Małgorzata Sadkowska-Todys; Piotr Karabowicz; Piotr Majewski; Przemysław Biecek. Joanna Reszeć; Radosław Charkiewicz; Tomasz Wołkowicz
EPI_ISL_2455193	Diagnostyka	1. Tricity SARS-CoV-2 sequencing consortium: University of Gdansk, Medical University of Gdansk, Vaxican Ltd., Invicta Ltd. 2. National Institute of Public Health - National Institute of Hygiene, Warsaw, Poland	Celina Cybulska; Karolina Gackowska; Katarzyna Groth; Katarzyna Zacharczuk; Krystyna Bienkowska Szewczyk; Lukasz Rabalski; Maciej Grzybek; Maciej Kosinski; Magdalena Nowakowska; Marcin Lubocki; Małgorzata Sadkowska-Todys; Tomasz Wołkowicz
EPI_ISL_2503908, EPI_ISL_2503914	Diagnostyka Sp. z o.o.	1. Academic Center for Pathomorphological and Genetic-Molecular Diagnostics ltd, Bialystok, Poland 2. National Institute of Public Health - National Institute of Hygiene, Warsaw, Poland	Anetta Sulewska; Jacek Nikliński; Janusz Dzięcioł; Joanna Kiśluk; Katarzyna Zacharczuk; Konrad Raczkowski; Magdalena Nowakowska; Małgorzata Sadkowska-Todys; Piotr Karabowicz; Piotr Majewski; Przemysław Biecek. Joanna Reszeć; Radosław Charkiewicz; Tomasz Wołkowicz
EPI_ISL_2365789	Diagnostyka Sp. z o.o.	1. National Institute of Public Health - National Institute of Hygiene, Warsaw, Poland 2. Biobank Lab, University of Lodz 3. Laboratory of Respiratory Viruses, Teaching and Clinical Center of the Medical University of Lodz	Dominik Strapagiel; Izabela Dróżdż; Jakub Lach; Katarzyna Zacharczuk; Klaudyna Królikowska; Maciej Borowiec; Magdalena Nowakowska; Magdalena Traczyk-Borszyńska; Marcin Słomka; Marta Sobalska-Kwapis; Małgorzata Sadkowska-Todys; Tomasz Płoszaj; Tomasz Wołkowicz
EPI_ISL_2495292	Diagnostyka Sp. z o.o.	1. National Institute of Public Health - National Institute of Hygiene; 2. Eurofins Genomics Europe Sequencing GmbH	ECDC COVID-19 WGS support team; Eurofins Genomics Europe Sequencing Team; Gierczyński Rafał; Sadkowska-Todys Małgorzata; Wołkowicz Tomasz; Zacharczuk Katarzyna
EPI_ISL_2140365, EPI_ISL_2140366, EPI_ISL_2140367	Diagnostyka Sp. z o.o.	1. Tricity SARS-CoV-2 sequencing consortium: University of Gdansk, Medical University of Gdansk, Vaxican Ltd., Invicta Ltd. 2. National Institute of Public Health - National Institute of Hygiene, Warsaw, Poland	Celina Cybulska; Karolina Gackowska; Katarzyna Groth; Katarzyna Zacharczuk; Krystyna Bienkowska Szewczyk; Lukasz Rabalski; Maciej Grzybek; Maciej Kosinski; Magdalena Nowakowska; Małgorzata Sadkowska-Todys; Tomasz Wołkowicz
EPI_ISL_2423033	Diagnostyka. Laboratoria Medyczne.	1. ViroGenetics - BSL3 Laboratory of Virology, Małopolska Centre of Biotechnology, Jagiellonian University; 2. Diagtron Laboratoria Lukasz Rabalski	Foremny J; Gromowski, T.; Kowalski, M.; Labaj; Maciej Kosinski; Mazur-Panasiuk, N.; Natalia Derewonko; P.P.; Pyrc, K.; Rabalski L.; Rogalska-Kupiec M.; Swadzba J.; Sylwia Januszczak; Szulc, P.; Wydmanski, W.; Zielinska M
EPI_ISL_2821066	Diagnostyka. Laboratoria Medyczne.	1. ViroGenetics - BSL3 Laboratory of Virology, Małopolska Centre of Biotechnology, Jagiellonian University; 2. Diagtron Laboratoria Łukasz Rąbalski	Gromowski, T.; Kowalski, M.; Labaj; Maciej Kosinski; Mazur-Panasiuk, N.; Natalia Derewonko; P.P.; Pyrc, K.; Rabalski L.; Rogalska-Kupiec M.; Swadzba J.; Sylwia Januszczak; Szulc, P.; Wydmanski, W.
EPI_ISL_2319009, EPI_ISL_2423090, EPI_ISL_2423128, EPI_ISL_2423135, EPI_ISL_2423139	Diagnostyka. Laboratoria Medyczne.	1. ViroGenetics - BSL3 Laboratory of Virology, Małopolska Centre of Biotechnology, Jagiellonian University; 2. genXone SA, Research & Development Laboratory	Brylak A; Drweska-Matelska N; Gidlewicz A; Grabowski J; Gromowski, T.; Januszczak S; Kaszuba M; Kowalski, M.; Krych L; Labaj; Mazur-Panasiuk, N.; Nowicki G; P.P.; Pyrc, K.; Sykulski M; Szeszko K; Szulc, P.; Wydmanski W.
EPI_ISL_2694759, EPI_ISL_2694775	Diagnostyka. Laboratoria Medyczne.	1. ViroGenetics - BSL3 Laboratory of Virology, Małopolska Centre of Biotechnology, Jagiellonian University; 2. Diagtron Laboratoria Łukasz Rąbalski	Gromowski, T.; Kowalski, M.; Labaj; Maciej Kosinski; Mazur-Panasiuk, N.; Natalia Derewonko; P.P.; Pyrc, K.; Rabalski L.; Rogalska-Kupiec M.; Swadzba J.; Sylwia Januszczak; Szulc, P.; Wydmanski, W.
EPI_ISL_2597956, EPI_ISL_2597959, EPI_ISL_2599793, EPI_ISL_2599798, EPI_ISL_2812972, EPI_ISL_2820237, EPI_ISL_2820240			
see above	Dianalabs SA	Genesupport	Geraldine Jost; Katia Jaton; Nadia Liassine; Tanguy ARAUD
EPI_ISL_2115134, EPI_ISL_2115136, EPI_ISL_2115145	Dianovis GmbH Greiz	Robert Koch Institute	
EPI_ISL_2379462, EPI_ISL_2379496, EPI_ISL_2379497, EPI_ISL_2379498, EPI_ISL_2379520, EPI_ISL_2379564, EPI_ISL_2379645, EPI_ISL_2379646, EPI_ISL_2379648			
see above	Dr S Raju, Director of Public Heath and Preventive Medicine	inStem NCBS - INSACOG	Uma Ramakrishnan Dasaradhi Palakodeti Aswin SaiNarain
EPI_ISL_2886172, EPI_ISL_2886173	Dr. Gernot Walder GmbH	Dr. Gernot Walder GmbH	Sissy T. Sonnleitner
EPI_ISL_2145682, EPI_ISL_2145703, EPI_ISL_2145782, EPI_ISL_2449950, EPI_ISL_2449966, EPI_ISL_2449987, EPI_ISL_2449989, EPI_ISL_2449990, EPI_ISL_2483493, EPI_ISL_2860463, EPI_ISL_2860467, EPI_ISL_2860528, EPI_ISL_2860529, EPI_ISL_2860564			
see above	Dutch COVID-19 response team	Erasmus Medical Center	Anne van der Linden; Annemiek van der Eijk; Bas Oude Munnink; Corine GeurtsvanKessel; David Nieuwenhuijse; Emmanuelle Munger; Irina Chestakova; Marion Koopmans; Marjan Boter; Reina Sikkema; Richard Molenkamp; on behalf of the Dutch national COVID-19 respo
EPI_ISL_1792906, EPI_ISL_1792907, EPI_ISL_1792922, EPI_ISL_1962940, EPI_ISL_1962944, EPI_ISL_1962964, EPI_ISL_1962965, EPI_ISL_1962966, EPI_ISL_2094352, EPI_ISL_2094353, EPI_ISL_2094354, EPI_ISL_2094386, EPI_ISL_2094387, EPI_ISL_2094388, EPI_ISL_2094389, EPI_ISL_2094390, EPI_ISL_2094391, EPI_ISL_2094392, EPI_ISL_2094393, EPI_ISL_2094394, EPI_ISL_2220505, EPI_ISL_2220507, EPI_ISL_2220513, EPI_ISL_2220515, EPI_ISL_2220518, EPI_ISL_2220520, EPI_ISL_2303934, EPI_ISL_2303935, EPI_ISL_2303973, EPI_ISL_2303974, EPI_ISL_2303975, EPI_ISL_2303976, EPI_ISL_2303977, EPI_ISL_2303980, EPI_ISL_2303981, EPI_ISL_2303982, EPI_ISL_2303983, EPI_ISL_2303984, EPI_ISL_2405621, EPI_ISL_2405646, EPI_ISL_2405815, EPI_ISL_2405912, EPI_ISL_2476259, EPI_ISL_2476260, EPI_ISL_2476261, EPI_ISL_2476262, EPI_ISL_2476263, EPI_ISL_2476311, EPI_ISL_2476313, EPI_ISL_2476314, EPI_ISL_2476316, EPI_ISL_2476317, EPI_ISL_2476321, EPI_ISL_2476322, EPI_ISL_2476325, EPI_ISL_2476327, EPI_ISL_2476329, EPI_ISL_2476331, EPI_ISL_2476332, EPI_ISL_2609791, EPI_ISL_2609827, EPI_ISL_2610086, EPI_ISL_2610198, EPI_ISL_2610257, EPI_ISL_2610314, EPI_ISL_2610329, EPI_ISL_2610342, EPI_ISL_2610435, EPI_ISL_2610461, EPI_ISL_2610494, EPI_ISL_2610564, EPI_ISL_2610596, EPI_ISL_2610865, EPI_ISL_2610895, EPI_ISL_2610914, EPI_ISL_2672306, EPI_ISL_2672558, EPI_ISL_2672582, EPI_ISL_2672616, EPI_ISL_2672651, EPI_ISL_2672683, EPI_ISL_2672798, EPI_ISL_2672845, EPI_ISL_2673072, EPI_ISL_2673107, EPI_ISL_2673108, EPI_ISL_2673115, EPI_ISL_2673124, EPI_ISL_2673143, EPI_ISL_2673153, EPI_ISL_2673162, EPI_ISL_2673183, EPI_ISL_2673193, EPI_ISL_2673202, EPI_ISL_2673341, EPI_ISL_2673369, EPI_ISL_2673395, EPI_ISL_2788157, EPI_ISL_2788159, EPI_ISL_2788166, EPI_ISL_2788168, EPI_ISL_2788170, EPI_ISL_2788176, EPI_ISL_2788190, EPI_ISL_2788214, EPI_ISL_2788241, EPI_ISL_2788242, EPI_ISL_2788253, EPI_ISL_2788267, EPI_ISL_2788291, EPI_ISL_2788295, EPI_ISL_2788296, EPI_ISL_2788306, EPI_ISL_2788315, EPI_ISL_2788340, EPI_ISL_2862893, EPI_ISL_2862903, EPI_ISL_2862905, EPI_ISL_2862916, EPI_ISL_2862920, EPI_ISL_2862935, EPI_ISL_2862937, EPI_ISL_2862941, EPI_ISL_2862961, EPI_ISL_2862968, EPI_ISL_2862985, EPI_ISL_2862987, EPI_ISL_2863007, EPI_ISL_2863012, EPI_ISL_2863122, EPI_ISL_2863244, EPI_ISL_2863264, EPI_ISL_2863344, EPI_ISL_2863369, EPI_ISL_2863371, EPI_ISL_2863385			
see above	Dutch COVID-19 response team	National Institute for Public Health and the Environment (RIVM)	Adam Meijer; AnneMarie van den Brandt; Annelies Kroneman; Bas van der Veer; Chantal Reusken; Dennis Schmitz; Dirk Eggink; Euníce Then; Florian Zwagemaker; Harry Vennema; James Groot; Jeroen Cremer; Karim Hajji; Kim Freriks; Linda van de Nes; Lisa Wijsman; Lynn Aarts; Melissa van Tuil; Robert Kohl; Ryanne Jaarsma; Sanne Bos; Sharon van den Brink; Sjoerd Kuiling; on behalf of the national COVID-19 response team
EPI_ISL_2322968	EHC MORGES	Laboratory of genomics and metagenomics	Claire Bertelli; Damien Jacot; Gilbert Greub; Sébastien Aeby; Trestan Pillonel

EPI_ISL_2614730	EKA Hospital BSD	Eijkman Institute for Molecular Biology, National Research and Innovation Agency	Amin Soebandrio; Edison Johar; Frilasita A Yudhaputri; Hidayat Trimarsanto; Iskandar Adnan; Khin Saw Myint; Lidwina Priliani; Lydia V. Panggalo; Muhammad Rezki Rasyak; Safarina G Malik; Sukma Oktavianthi; Willy Agustine
EPI_ISL_2477869, EPI_ISL_2478515, EPI_ISL_2479023, EPI_ISL_2479127, EPI_ISL_2479472, EPI_ISL_2479857, EPI_ISL_2479874			
see above	Edmonton Provincial Lab	Public Health Agency of Canada (PHAC) National Microbiology Laboratory	Buss; Croxen M; Deo A; Dieu P; E; Ferrato C; Gill K; Khan F; Koleva P; Li V; Lloyd C; Lynch T; Ma R; Murphy S; Pabbaraju K; Shokoples S; Thayer J; Tipples G; Whitehouse M; Wong A; Yu C; Zelyas N
EPI_ISL_2657533	Elling group, Institute of Molecular Biotechnology (IMBA)	Bergthaler laboratory, CeMM Research Center for Molecular Medicine of the Austrian Academy of Sciences	Andreas Bergthaler; Anna Schedl; Bekir Erguner; Benedikt Agerer; Christoph Bock; Fabian Amman; Jan Laine; Lukas Endler; Maelle Le Moing; Martin Senekowitsch; Matthew Thornton; Michael Schuster; Petr Triska; Thomas Penz
EPI_ISL_1845854, EPI_ISL_1846077, EPI_ISL_1846153, EPI_ISL_2110448, EPI_ISL_2110453, EPI_ISL_2110454, EPI_ISL_2110460, EPI_ISL_2110488, EPI_ISL_2110489, EPI_ISL_2110499, EPI_ISL_2110737, EPI_ISL_2123736, EPI_ISL_2123791, EPI_ISL_2123794, EPI_ISL_2123795, EPI_ISL_2123806, EPI_ISL_2259546, EPI_ISL_2314355, EPI_ISL_2316836, EPI_ISL_2316849, EPI_ISL_2316937, EPI_ISL_2317006, EPI_ISL_2471660, EPI_ISL_2471662, EPI_ISL_2471688, EPI_ISL_2471689, EPI_ISL_2472053, EPI_ISL_2634648, EPI_ISL_2634680			
see above	Eurofins LifeCodexx GmbH	Robert Koch Institute	
EPI_ISL_2646231	Exalab	Cerba lab	Aude Lessenne; Bénédicte Roquebert; Emmanuel Lecorche; Kader Merah; Laura Verdurme; Patrice Herisson; Sabine Trombert-Paolantoni; Stéphanie Haïm-Boukobza; Thierry Collin
EPI_ISL_2840294, EPI_ISL_2840296, EPI_ISL_2840298, EPI_ISL_2840299	FL Bur. of Public Health Laboratories-Jacksonville	Centers for Disease Control and Prevention Division of Viral Diseases, Pathogen Discovery	Alison Laufer Halpin; Ben L. Rambo-Martin; Clinton R. Paden; Dakota Howard; Darlene Wagner; Dave Wentworth; Dhwani Batra; Jasmine Padilla; Justin Lee; Katie Dillon; Krista Queen; Kristen Knipe; Kristine Lacek; Mark Burroughs; Matthew Schmerer; Mili Sheth; Peter Cook; Sam Shepard; Sarah Nobles; Shoshona Le; Suxiang Tong; Vivien Dugan; Yvette Unoarumhi
EPI_ISL_2840428	FL Bureau of Public Health Laboratories-Miami	Centers for Disease Control and Prevention Division of Viral Diseases, Pathogen Discovery	Alison Laufer Halpin; Ben L. Rambo-Martin; Clinton R. Paden; Dakota Howard; Darlene Wagner; Dave Wentworth; Dhwani Batra; Jasmine Padilla; Justin Lee; Katie Dillon; Krista Queen; Kristen Knipe; Kristine Lacek; Mark Burroughs; Matthew Schmerer; Mili Sheth; Peter Cook; Sam Shepard; Sarah Nobles; Shoshona Le; Suxiang Tong; Vivien Dugan; Yvette Unoarumhi
EPI_ISL_2840281	FL Bureau of Public Health Laboratories-Tampa	Centers for Disease Control and Prevention Division of Viral Diseases, Pathogen Discovery	Alison Laufer Halpin; Ben L. Rambo-Martin; Clinton R. Paden; Dakota Howard; Darlene Wagner; Dave Wentworth; Dhwani Batra; Jasmine Padilla; Justin Lee; Katie Dillon; Krista Queen; Kristen Knipe; Kristine Lacek; Mark Burroughs; Matthew Schmerer; Mili Sheth; Peter Cook; Sam Shepard; Sarah Nobles; Shoshona Le; Suxiang Tong; Vivien Dugan; Yvette Unoarumhi
EPI_ISL_2341936	FM Medical College, Balasore	Institute of Life Sciences - INSACOG	Ajay Parida; Amol M. Kanampalliwar; Arup Ghosh; Atimukta Jha; INSACOG Consortium; Omprakash Shiriwas; Punit Prasad; Rajeeb Swain; Rupesh Dash; Safal Walia; Sana Fatma; Shifu Aggarwal; Sunil K. Raghav
EPI_ISL_2695316, EPI_ISL_2695317	FMUP	Instituto Nacional de Saude (INSA)	Borges et al
EPI_ISL_2288947, EPI_ISL_2288948, EPI_ISL_2288973	FUNDACIO ALTHAIA-MANRESA	Banc de Sang i Teixits	Carlos Hobeich; Francisco Vidal; Irene Corrales; Lorena Ramírez; Maria Glòria Soria; Natália Comes; Nina Borràs; Noemí Gonzalez; Silvia Sauleda
EPI_ISL_2038893	Federal Budget Health Care Institution "Center of Hygiene and Epidemiology in Tver region"	Group of Genomics and Postgenomic Technologies of Central Research Institute of Epidemiology	Akimkin VG; Kaptelova VV; Kondrasheva LY; Korneenko EV; Melushenkova TG; Nadtoka MI; Saenko SS; Samoilov AE; Smirnova YS; Speranskaya AS; Tivanova EV
EPI_ISL_2466607	Felix-Platter Spital	Clinical Bacteriology	Adrian Egli; Alfredo Mari; Fanny Wegner; Hans Hirsch; Helena MB Seth-Smith; Julia Bielicki; Karoline Leuzinger; Manuel Battegay; Tim Roloff
EPI_ISL_2466841, EPI_ISL_2467518, EPI_ISL_2467519, EPI_ISL_2467871, EPI_ISL_2467935, EPI_ISL_2467936	Fimlab Laboratories	Fimlab Laboratories	Bruno Luukinen; Leena Huhti; Mauri Keinänen; Minna Paloniemi; Sara Lehtinen; Tapio Seiskari
EPI_ISL_2363870, EPI_ISL_2363874, EPI_ISL_2557176, EPI_ISL_2557177, EPI_ISL_2557179, EPI_ISL_2557180, EPI_ISL_2557181, EPI_ISL_2557182, EPI_ISL_2557183, EPI_ISL_2557184, EPI_ISL_2557185, EPI_ISL_2557186, EPI_ISL_2557187, EPI_ISL_2557193, EPI_ISL_2557194, EPI_ISL_2557195, EPI_ISL_2557196, EPI_ISL_2557197, EPI_ISL_2557198, EPI_ISL_2557199, EPI_ISL_2557200, EPI_ISL_2557201, EPI_ISL_2557202, EPI_ISL_2557203, EPI_ISL_2557204, EPI_ISL_2557205, EPI_ISL_2557206, EPI_ISL_2557207, EPI_ISL_2557208, EPI_ISL_2557209, EPI_ISL_2557210, EPI_ISL_2644866, EPI_ISL_2644898, EPI_ISL_2644901, EPI_ISL_2645068, EPI_ISL_2645071, EPI_ISL_2645084, EPI_ISL_2645086, EPI_ISL_2645108, EPI_ISL_2645111, EPI_ISL_2645112, EPI_ISL_2645113, EPI_ISL_2645117, EPI_ISL_2645205, EPI_ISL_2645212, EPI_ISL_2645216, EPI_ISL_2645217, EPI_ISL_2645218			
see above	Fimlab Laboratoriot Oy Tampere	Expert Microbiology, National Institute for Health and Welfare	Carita Savolainen-Kopra; Erika Lindh; Haider al-Hello; Jani Halkilahti; Kirsi Liitsola; Niina Ikonen; Olli Vapalahti; Pekka Ellonen; Phuoc Truong; Päivi Laurila; Ravi Kant; Sari Hannula; Soile Blomqvist; Teemu Smura
EPI_ISL_2558057	First Aide Diagnostic Center	Philippine Genome Center	Alethea R. de Guzman; Anna Ong-Lim; Arianne A. Zamora; Asia Louisa U. Chong; Benedict A. Maralit; Candice Francheska B. Tambaoan; Carlo M. Lapid; Celia Carlos; Devon Ray Pacial; Edsel Maurice Salvaña; El King D. Morado; Elcid Aaron R. Pangilinan; Eva Maria Cutiongco-de la Paz; Francis A. Tablizo; Irish Coleen A. Asin; Jaime C. Montoya; Jan Michael C. Yap; Jo-Hannah S. Llames; John Q. Wong; Joshua Gregor A. Dizon; Juan Antonio R. Magalang; Karol Sophia Agape R. Padilla; Kenneth M. Kim; Kris P. Punayan; Marc Edsel C. Ayes; Maria Rosario Singh-Vergeire and Cynthia P. Saloma; Maria Sofia L. Yangzon; Marissa Alejandria; Razel Nikka M. Hao; Renato Jacinto Q. Mantaring; Rianna Patricia S. Cruz; Sheila Mae M. Araiza
EPI_ISL_2230013, EPI_ISL_2246887, EPI_ISL_2246888, EPI_ISL_2246889, EPI_ISL_2836030, EPI_ISL_2836077, EPI_ISL_2836102, EPI_ISL_2836176, EPI_ISL_2836254, EPI_ISL_2836281, EPI_ISL_2836425, EPI_ISL_2836519, EPI_ISL_2836527, EPI_ISL_2836585, EPI_ISL_2836586, EPI_ISL_2836637, EPI_ISL_2836639, EPI_ISL_2836640, EPI_ISL_2836642, EPI_ISL_2836644, EPI_ISL_2836648, EPI_ISL_2836719, EPI_ISL_2836726, EPI_ISL_2836730, EPI_ISL_2836731, EPI_ISL_2836737, EPI_ISL_2836775, EPI_ISL_2836781, EPI_ISL_2836787, EPI_ISL_2836789, EPI_ISL_2836804, EPI_ISL_2836836, EPI_ISL_2836837, EPI_ISL_2836842, EPI_ISL_2836843			
see above	Florida Bureau of Public Health Laboratories	Florida Bureau of Public Health Laboratories	Jason Blanton; Sarah Schmedes
EPI_ISL_2889854	Fondation Congolaise pour la recherche medicale (FCRM)	Fondation Congolaise pour la Recherche Médicale	Abel Lissom; Batchi-Bouyou Armel Landry; Francine Ntoumi; Jean Claude Djontu; Mfoutou Mapanguy Claujens Chastel; Thirumalaisamy P. Velavan
EPI_ISL_2620951, EPI_ISL_2663244, EPI_ISL_2728608, EPI_ISL_2728614, EPI_ISL_2728618, EPI_ISL_2863954	Fondazione IRCCS Ca' Granda Ospedale Maggiore Policlinico	Fondazione IRCCS Ca' Granda Ospedale Maggiore Policlinico	Ferruccio Ceriotti; Sara Uceda Renteria
EPI_ISL_2125024	Fraunhofer-Institut fur Zelltherapie und Immunologie IZI AG Next-Generation Diagnostics	Robert Koch Institute	
EPI_ISL_2262318	Fraunhofer-Institut für Zelltherapie und Immunologie IZI AG Next-Generation Diagnostics	Robert Koch Institute	
EPI_ISL_1614854, EPI_ISL_1666372, EPI_ISL_1666379, EPI_ISL_1666447, EPI_ISL_1667317, EPI_ISL_1839348, EPI_ISL_1992594, EPI_ISL_1992667, EPI_ISL_1992684, EPI_ISL_1993163, EPI_ISL_2134078, EPI_ISL_2134105, EPI_ISL_2134268, EPI_ISL_2160771, EPI_ISL_2160774, EPI_ISL_2160840, EPI_ISL_2480069, EPI_ISL_2686545, EPI_ISL_2873487, EPI_ISL_2873516, EPI_ISL_2873747			
see above	Fulgent Genetics	Centers for Disease Control and Prevention Division of Viral Diseases, Pathogen Discovery	Adrian Paskey; Becky Tsai; Benafsh Sapra; Benjamin Rambo-Martin; Christopher Gulvick; Clinton R. Paden; Dakota Howard; Darlene Wagner; Dhwani Batra; Doreen Ng; Duncan MacCannell; Harry Gao; James Xie; Jason Caravas; John Gao; Joseph Fierro; Kara Moser; Matthew Schmerer; Mickey Li; Peter W. Cook; Scott Sammons; Shatavia Morrison; Yan Meng; Yvette Unoarumhi
EPI_ISL_2620737	Functional Genomics and Proteomics Laboratory (FGPL), Department of Genetic Engineering and Biotechnology, University of Chittagong	Virology Laboratory, International Centre for Diarrhoeal Disease Research, Bangladesh (ICDDR,B)	Amit Datta; Amlan Bhattacharjee; Dr. Laila Khaleda; Dr. Mohammad Al-Forkan; Dr. Mohammad Enayet Hossain; Dr. Mustafizur Rahman; Dr. Sujat Paul; Inzamamul Ismail Shawon; Md. Abdur Rahman; Md. Jibran Alam; Miftah Mushfique; Mojnu Miah; Rahee Hasan Chowdhury; Rashedul Hasan; Shuva Das
EPI_ISL_2311861	Fundação Ezequiel Dias	Coordenação Geral de Laboratórios de Saúde Pública (CGLAB/DAEVS/SVS/MS)	Vagner Fonseca; et al.
EPI_ISL_2333503, EPI_ISL_2333508, EPI_ISL_2333509, EPI_ISL_2333511, EPI_ISL_2333512, EPI_ISL_2425841, EPI_ISL_2491418, EPI_ISL_2491419, EPI_ISL_2491420, EPI_ISL_2491421, EPI_ISL_2491422, EPI_ISL_2491423, EPI_ISL_2491424, EPI_ISL_2491567, EPI_ISL_2491568, EPI_ISL_2549073, EPI_ISL_2549074, EPI_ISL_2549075, EPI_ISL_2549076, EPI_ISL_2549096, EPI_ISL_2549098, EPI_ISL_2611979, EPI_ISL_2611980, EPI_ISL_2643672, EPI_ISL_2644695, EPI_ISL_2644696, EPI_ISL_2644715, EPI_ISL_2673713, EPI_ISL_2673714, EPI_ISL_2673716, EPI_ISL_2673719, EPI_ISL_2673723, EPI_ISL_2673736, EPI_ISL_2673737, EPI_ISL_2673738, EPI_ISL_2673740, EPI_ISL_2673741, EPI_ISL_2673744, EPI_ISL_2673745, EPI_ISL_2673746, EPI_ISL_2673748, EPI_ISL_2673753, EPI_ISL_2673764, EPI_ISL_2673766, EPI_ISL_2675096, EPI_ISL_2688593, EPI_ISL_2688598, EPI_ISL_2725007, EPI_ISL_2725088, EPI_ISL_2725089, EPI_ISL_2725090, EPI_ISL_2788584, EPI_ISL_2788585, EPI_ISL_2788586, EPI_ISL_2788587, EPI_ISL_2788588, EPI_ISL_2788593, EPI_ISL_2788594, EPI_ISL_2790016, EPI_ISL_2790017, EPI_ISL_2820835, EPI_ISL_2820838, EPI_ISL_2820840, EPI_ISL_2820844, EPI_ISL_2820868, EPI_ISL_2820878			
see above	Furst Medical Laboratory	Norwegian Institute of Public Health, Department of Virology	'Kathrine Stene-Johansen; Atiya R Ali; Debech Nadia; Engebretsen Serina Beate; Garcia Llorente Ignacio; Hilde Elshaug; Hilde Vollan; Jon Bråte; Kamilla Heddeland Instefjord; Karoline Bragstad; Kathrine Stene-Johansen; Line Victoria Moen; Marie Paulsen Madsen; Olav Hungnes; Pedersen Benedikte Nevjen; Rasmus Riis Kopperud
EPI_ISL_2376424	GA Department of Public Health Laboratory	Genomics and Discovery, Respiratory Viruses Branch, Division of Viral Diseases, Centers for Disease Control and Prevention	Adam Retchless; Anna Kelleher; Anna Uehara; Brian Lynch; Clinton R. Paden; Dhwani Batra; Haibin Wang; Han Jia Justin Ng; Jasmine Padilla; Jing Zhang; Justin Lee; Krista Queen; Mark Burroughs; Mili Sheth; Morgan Davis; Peter Cook; Rachel Marine; Sarah Nobles; Suxiang Tong; Tara Coalter; Yan Li; Ying Tao
EPI_ISL_2544823, EPI_ISL_2544835	GMERS Government Medical College (GMC), Gotri	Gujarat Biotechnology Research Centre	Bithika Duttaroy; Chaitanya Joshi; Dinesh Kumar; Janvi Raval; Madhvi Joshi; Nitesh Shah; Nitin Savaliya; Ramesh Pandit; Sonal Sharma; Twinkle Soni; Umang Mishra; Zarna Patel; Zuber Saiyed
EPI_ISL_2001183	GMERS, Government Medical College (GMC), Gotri	Gujarat Biotechnology Research Centre	Bithika Duttaroy; Chaitanya Joshi; Dinesh Kumar; Janvi Raval; Madhvi Joshi; Nitesh Shah; Nitin Savaliya; Ramesh Pandit; Sonal Sharma; Twinkle Soni; Umang Mishra; Zarna Patel; Zuber Saiyed
EPI_ISL_2001140	GMERS, Government Medical College, Gandhinagar	Gujarat Biotechnology Research Centre	Chaitanya Joshi; Dinesh Kumar; Gaurishankar Shrimali; Janvi Raval; Madhvi Joshi; Nitesh Shah; Nitin Savaliya; Ramesh Pandit; Sonal Sharma; Twinkle Soni; Umang Mishra; Zarna Patel; Zuber Saiyed
EPI_ISL_2726782	GenXOne SA	1. National Institute of Public Health - National Institute of Hygiene, Warsaw, Poland 2. Biobank Lab, University of Lodz 3. Laboratory of Respiratory Viruses, Teaching and Clinical Center of the Medical University of Lodz	Dominik Strapagiel; Izabela Dróżdż; Jakub Lach; Katarzyna Zacharczuk; Klaudyna Królikowska; Maciej Borowiec; Magdalena Nowakowska; Magdalena Traczyk-Borszyńska; Marcin Słomka; Marta Sobalska-Kwapis; Małgorzata Sadkowska-Todys; Tomasz Płoszaj; Tomasz Wołkowicz
EPI_ISL_2756159, EPI_ISL_2893002, EPI_ISL_2894958	Genetica Molecular and Subdepartamento de Virologia ISP Chile	Instituto de Salud Publica de Chile	Andres Castillo; Barbara Parra; Constanza Campano; Gisselle Barra; Javier Tognarelli; Jorge Fernandez; Karen Orostica; Loredana Arata; Patricia Bustos; Rodrigo Fasce; Soledad Ulloa
EPI_ISL_1942249, EPI_ISL_2036272	Genome Centre	Genome Centre	A. S. M. Rubayet Ul Alam; Ali Ahsan Setu; Hassan M. Al-Emran; Hassan Md. Al-Emran; Iqbal Kabir Jahid; M. Anwar Hossain; M. Shaminur Rahman; M. Tanvir Islam; Md. Shazid Hasan; Md. Tanvir Islam; Ovinu Kibria Islam; Prosanto Kumar Das; Shovon Lal Sarkar; Tanay Chakrabarty
EPI_ISL_2565983, EPI_ISL_2773945, EPI_ISL_2773946, EPI_ISL_2854210, EPI_ISL_2861718	Genome Information Research Center, Research Institute for Microbial Diseases, Osaka University	Genome Information Research Center, Research Institute for Microbial Diseases, Osaka University	Daisuke Motooka; Hiroya Oki; Kentaro Tanaka; and Shota Nakamura
EPI_ISL_2858872, EPI_ISL_2862010, EPI_ISL_2862016,	Genomic Research Laboratory, Bangabandhu Sheikh Mujib Medical University	Genomic Research Laboratory, BSMMU	Arifa Akram; Laila Anjuman Banu; Md.Saydur Rahman; Md.Sharfuddin Ahmed; Sharadindu Kanti Sinha

EPI_ISL_2862048			
EPI_ISL_2862015	Genomic Research Laboratory, Bangabandhu Sheikh Mujib Medical University (BSMMU) Shahbag, Dhaka_1000	Genomic Research Laboratory, BSMMU	Arifa Akram; Laila Anjuman Banu; Md.Saydur Rahman; Md.Sharfuddin Ahmed; Sharadindu Kanti Sinha
EPI_ISL_2248870, EPI_ISL_2248871, EPI_ISL_2248874, EPI_ISL_2340152	Germano de Sousa	Instituto Nacional de Saude (INSA)	Borges et al
EPI_ISL_2454302	Greek Genome Center, Biomedical Research Foundation of the Academy of Athens (BRFAA)	Greek Genome Center, Biomedical Research Foundation of the Academy of Athens (BRFAA)	Dimitrios Thanos; Emmanouil Athanasiadis; Giannis Vatsellas; Katerina Zoi; Theodoros Loupis
EPI_ISL_2364992	Greek Genome Center, Biomedical Research Foundation of the Academy of Athens (BRFAA)	Greek Genome Center, Biomedical Research Foundation of the Academy of Athens (BRFAA)	Dimitrios Thanos; Emmanouil Athanasiadis; Giannis Vatsellas; Katerina Zoi; Theodoros Loupis
EPI_ISL_2788689, EPI_ISL_2788711, EPI_ISL_2788717, EPI_ISL_2788719	Gunma Prefectural Institute of Public Health and Environmental Sciences	Gunma Prefectural Institute of Public Health and Environmental Sciences	Daisuke Shinoda; Hiroyuki Tsukagoshi; Kentaro Itokawa; Makoto Kuroda; Mariko Saito; Masanori Hashino; Rina Tanaka; Tsuyoshi Sekizuka
EPI_ISL_2695287	H Braga	Instituto Nacional de Saude (INSA)	Borges et al
EPI_ISL_2340227	H Divino Espirito Santo - Ponta Delgada	Instituto Nacional de Saude (INSA)	Borges et al
EPI_ISL_2694948, EPI_ISL_2895078	H Fernando Fonseca	Instituto Nacional de Saude (INSA)	Borges et al
EPI_ISL_2536098	H Forcas Armadas - Polo Lisboa	Instituto Nacional de Saude (INSA)	Borges et al
EPI_ISL_2695075	H Vila Franca Xira	Instituto Nacional de Saude (INSA)	Borges et al
EPI_ISL_2029113, EPI_ISL_2029114	HEGP - Laboratoire de Virologie	HEGP - Laboratoire de Virologie	David Veyer; Hélène Péré; Julien Puech; Maxime Wack; Nicolas Robillard; Sandrine Imbeaud
EPI_ISL_2385214, EPI_ISL_2385226, EPI_ISL_2385230, EPI_ISL_2385242, EPI_ISL_2385266, EPI_ISL_2385267, EPI_ISL_2385279, EPI_ISL_2385280, EPI_ISL_2385284, EPI_ISL_2385288, EPI_ISL_2385292, EPI_ISL_2385308, EPI_ISL_2450606, EPI_ISL_2523633, EPI_ISL_2523639			
see above	HELIX LLC	WHO National Influenza Centre Russian Federation	Andrey Komissarov; Artem Fadeev; Daria Danilenko; Dmitry Lioznov; Elena Nabieva; Georgii Bazykin; Kirill Varchenko; Ksenia Safina; Kseniya Komissarova; Maria Pisareva; Maria Timofeeva; Mikhail Bakaev; Nikita Yolshin; Oula Mansour; Oula Masour; Tamila Musaeva; Veronika Eder
EPI_ISL_4562854	HIV Research Laboratory	Salemi Lab, University of Florida	Cash MN; Lauzardo M; Magalis BR; Mavian C; Riva A; Salemi M; Tagliamonte MS
EPI_ISL_2731451, EPI_ISL_2731452	HLAGYN-Laboratório de Imunologia de Transplantes de Goiás	Laboratory of Respiratory Viruses and Measles, Oswaldo Cruz Institute, FIOCRUZ	Alice Sampaio Rocha; Ana Carolina Mendonca; Anna Carolina Paixao; Elisa Cavalcante Pereira; Fernando Motta; Luciana Appolinario; Marilda Siqueira on behalf of the Fiocruz COVID-19 Genomic Surveillance Network; Paola Resende; Renata Serrano Lopes; Taina Venas
EPI_ISL_1904992, EPI_ISL_1904994, EPI_ISL_1904998	HOPITAL SAINT ANDRE	CNR Virus des Infections Respiratoires - France SUD	Antonin Bal; Bruno Lina; Gregory Destras; Gwendolyne Burfin; Hadrien Regue; Laurence Josset; Martine Valette; Quentin Semanas
EPI_ISL_2134906, EPI_ISL_2134911, EPI_ISL_2134921, EPI_ISL_2361925, EPI_ISL_2361946, EPI_ISL_2626103, EPI_ISL_2626151, EPI_ISL_2626158, EPI_ISL_2710232, EPI_ISL_2710234, EPI_ISL_2710236, EPI_ISL_2812710, EPI_ISL_2812724, EPI_ISL_2812726, EPI_ISL_2812752, EPI_ISL_2812756			
see above	HOSPITAL UNIVERSITARIO SON ESPASES	HOSPITAL UNIVERSITARIO SON ESPASES	Antonio Oliver; Carla López-Causapé; Hospital Universitario Son Espases; Pablo Fraile-Ribot; SeqCovid; Servicio de Microbiología
EPI_ISL_2511336, EPI_ISL_2682098, EPI_ISL_2765983, EPI_ISL_2779674	Health Services Laboratories	Wellcome Sanger Institute for the COVID-19 Genomics UK (COG-UK) Consortium	Cordelia Langford; David K. Jackson; Dominic Kwiatkowski; Ewan Harrison; Health Services Laboratories and Alex Alderton; Ian Johnston; Jeffrey Barrett; John Sillitoe on behalf of the Wellcome Sanger Institute COVID-19 Surveillance Team; Roberto Amato; Sonia Goncalves
EPI_ISL_1512099, EPI_ISL_1576106, EPI_ISL_1735500, EPI_ISL_1907632, EPI_ISL_1923785, EPI_ISL_1923911, EPI_ISL_1923955, EPI_ISL_1942165, EPI_ISL_1990856, EPI_ISL_2009735, EPI_ISL_2010430, EPI_ISL_2010872, EPI_ISL_2098013, EPI_ISL_2144255, EPI_ISL_2145113, EPI_ISL_2159577, EPI_ISL_2159751, EPI_ISL_2160284, EPI_ISL_2160637, EPI_ISL_2247397, EPI_ISL_2247606, EPI_ISL_2247625, EPI_ISL_2247704, EPI_ISL_2248458, EPI_ISL_2269619, EPI_ISL_2269822, EPI_ISL_2270252, EPI_ISL_2270430, EPI_ISL_2270535, EPI_ISL_2270570, EPI_ISL_2270707, EPI_ISL_2270722, EPI_ISL_2320572, EPI_ISL_2320767, EPI_ISL_2321076, EPI_ISL_2367643, EPI_ISL_2440107, EPI_ISL_2440260, EPI_ISL_2440528, EPI_ISL_2598863, EPI_ISL_2599267			
see above	Helix/Illumina	Centers for Disease Control and Prevention Division of Viral Diseases, Pathogen Discovery	Adrian Paskey; Alexandre Bolze; Ary Ascencio; Benjamin Rambo-Martin; Brad Sickler; Charlotte Rivera-Garcia; Christine Tran; Christopher Gulvick; Clinton R. Paden; Dakota Howard; Darlene Wagner; David Becker; Dhwani Batra; Duncan MacCannell; Efren Sandoval; Eileen de Feo; Elizabeth Cirulli; Eric Allen; Geraint Levan; James Lu; Jan Antico; Jason Caravas; Jason Nguyen; Jimmy Ramirez; Jingtao Liu; Kara Moser; Kelly Schiabor Barrett; Kim Gietzen; Magnus Isaksson; Marc Laurent; Matthew Schmerer; Matthew Tolentino; Nicole L. Washington; Peter W. Cook; Phil Febbo; Ryan Cho; Scott Sammons; Shannon Wickline; Shatavia Morrison; Sherry Wang; Simon White; Tyler Cassens; William Lee; Yvette Unoarumhi
EPI_ISL_2530206, EPI_ISL_2600958	Hematopathology Laboratory, ACTREC, TMC	Hematopathology Laboratory, ACTREC, TMC	ACTREC; Hematopathology Laboratory
EPI_ISL_2029931, EPI_ISL_2188352, EPI_ISL_2188418, EPI_ISL_2188419, EPI_ISL_2188450, EPI_ISL_2188452, EPI_ISL_2293281, EPI_ISL_2293284, EPI_ISL_2293285, EPI_ISL_2293303, EPI_ISL_2293304, EPI_ISL_2293305, EPI_ISL_2293306, EPI_ISL_2293307, EPI_ISL_2293308, EPI_ISL_2462965, EPI_ISL_2535475, EPI_ISL_2757663, EPI_ISL_2757664, EPI_ISL_2757665, EPI_ISL_2757666, EPI_ISL_2757667, EPI_ISL_2757668, EPI_ISL_2757669, EPI_ISL_2757670, EPI_ISL_2757671, EPI_ISL_2757672, EPI_ISL_2757673, EPI_ISL_2757674, EPI_ISL_2757675, EPI_ISL_2757676, EPI_ISL_2757677, EPI_ISL_2757678, EPI_ISL_2790981, EPI_ISL_2790982, EPI_ISL_2790983, EPI_ISL_2790984, EPI_ISL_2835879			
see above	Hospital	National Reference Center for Viruses of Respiratory Infections, Institut Pasteur, Paris	Adrien Pain; Amaury Vaysse; Angela Brisebarre; Carole Capel; Christophe Malabat; Corinne Maufrais; Elodie Gleize; Emmanuelle Permal; Etienne Simon-Lorière; Frédéric Lemoine; HéLèNe Roger; JéRôMe Durivault; Laura Djamdjian; Louise Lefrançois; LéA Pilorge; Marion Barbet; Maud Vanpeene; Méline Bizard; Nabil Gastli; Pierre Lechat; Raphael Guiheneuf; Sylvaine Bastian; Sylvie Behillil; Sylvie Van der Werf; Vincent Enouf
EPI_ISL_2727836	Hospital Angkatan Tentera Kota Kinabalu	Institute for Medical Research, Infectious Disease Research Centre, National Institutes of Health, Ministry of Health Malaysia	Azizan MA; Kamel K; Mohd Zawawi Z; Ramly N; Robert F; Suppiah J; Thayan R
EPI_ISL_2727837	Hospital Angkatan Tentera Wilayah Kota Kinabalu	Institute for Medical Research, Infectious Disease Research Centre, National Institutes of Health, Ministry of Health Malaysia	Azizan MA; Kamel K; Mohd Zawawi Z; Ramly N; Robert F; Suppiah J; Thayan R
EPI_ISL_2401863	Hospital Center Emile Mayrisch	Laboratoire national de sante, Microbiology, Microbial Genomics Platform	Anke Wienecke-Baldacchino; Catherine Ragimbeau; Cynthia Oxacelay; Fatu Djabi; Jessica Tapp; Lise Pignon; Raoul Salmon; Tamir Abdelrahman
EPI_ISL_1917450, EPI_ISL_2401928	Hospital Center Luxembourg	Laboratoire national de sante, Microbiology, Microbial Genomics Platform	Anke Wienecke-Baldacchino; Catherine Ragimbeau; Fatu Djabi; Jean-Hugues Francois; Jessica Tapp; Lise Pignon; Michel Kohnen; Raoul Salmon; Tamir Abdelrahman
EPI_ISL_2680929	Hospital Fátima S.A. de C.V.	Laboratorio nacional-LANIIA-CIAD	; Alejandra García-Gasca; Alejandra Hernández-Terán; Alejandro Sánchez-Flores; Alfredo Herrera-Estrella; Alicia Ocaña-Mondragón; Andreu Comas-García; Angel Gustavo Salas-Lais; Antonio Loza Román; Bernardo Martínez-Miguel; Blanca Taboada; Brenda Irasema Maldonado-Meza; Bruno Gómez-Gil; Carla Ivón Herrera-Najera; Carlos F. Arias; Celia Boukadida; Clara Esperanza Santacruz-Tinoco; Concepción Grajales-Muñiz; Consorcio Mexicano de Vigilancia Genómica (CoViGen-Mex). Authors (in alphabetical order): Julio Elias Alvarado-Yaah; Cristóbal Cháidez-Quiróz; Célida Duque Molina; Célida Martínez- Rodríguez; Daniel Fregoso-Rueda; Daniel Lira Morales; Eduardo Becerril-Vargas; Fernando Fontove-Herrera; Fidencio Mejía-Nepomuceno; Francisco Pulido; Gloria Elena Espinosa-Ayala; Gloria María Molina-Salinas; Gloria Vazquez; Hector Esteban Paz-Juárez; Hector Montoya-Fuentes; Helen Haydee Fernanda Ramírez-Plascencia; Irvin González-López; Jean Pierre González; Jesús Hernández; Joel Armando Vázquez-Pérez.; Jorge Salas-Hernández; José Antonio Enciso-Moreno; José Arturo Martínez-Orozco; José Esteban Muñoz-Medina; José de Jesús Nuñez-Contreras; Juan Bautista Chale-Dzul; Julissa Enciso-Ibarra; Luis Alberto Ochoa-Carrera; Margarita Matías-Florentino; Mario Mújica-Sánchez; Marissa Perez-Garcia; María Guadalupe Santiago-Mauricio; María Guadalupe de Jesús Mireles-Rivera; Nelly Sélem-Mojica; Pavel Isa; Ricardo Ciria Merce; Ricardo Grande; Rosa María Gutiérrez Rios; Santiago Ávila-Ríos; Selene Zárate; Susana Lopez; Verónica Mata-Haro; Victor Eduardo García-Arias; Víctor Hugo Borja-Aburto
EPI_ISL_2402478, EPI_ISL_2675298, EPI_ISL_2782376	Hospital General Universitario Gregorio Marañón	Hospital General Universitario Gregorio Marañón	Cristina Rodriguez-Grande; Darío García de Viedma; Julia Suárez; Laura Pérez-Lago; Marta Herranz Martin; Patricia Muñoz; Pedro Sola Campoy; Pilar Catalán; Sergio Buenestado Serrano; Victor Manuel de la Cueva
EPI_ISL_2233382, EPI_ISL_2550733	Hospital Labuan	Institute for Medical Research, Infectious Disease Research Centre, National Institutes of Health, Ministry of Health Malaysia	Azizan MA; Kamel K; Mohd Zawawi Z; Ramly N; Robert F; Suppiah J; Thayan R
EPI_ISL_2680937, EPI_ISL_2894553	Hospital Margarita Maza de Juárez	Microbial Genomics Laboratory	; Alejandra García-Gasca; Alejandra Hernández-Terán; Alejandro Sánchez-Flores; Alfredo Herrera-Estrella; Alicia Ocaña-Mondragón; Andreu Comas-García; Angel Gustavo Salas-Lais; Antonio Loza Román; Bernardo Martínez-Miguel; Blanca Taboada; Brenda Irasema Maldonado-Meza; Bruno Gómez-Gil; Carla Ivón Herrera-Najera; Carlos F. Arias; Celia Boukadida; Clara Esperanza Santacruz-Tinoco; Concepción Grajales-Muñiz; Consorcio Mexicano de Vigilancia Genómica (CoViGen-Mex). Authors (in alphabetical order): Julio Elias Alvarado-Yaah; Cristóbal Cháidez-Quiróz; Célida Duque Molina; Célida Martínez- Rodríguez; Daniel Fregoso-Rueda; Daniel Lira Morales; Eduardo Becerril-Vargas; Fernando Fontove-Herrera; Fidencio Mejía-Nepomuceno; Francisco Pulido; Gloria Elena Espinosa-Ayala; Gloria María Molina-Salinas; Gloria Vazquez; Hector Esteban Paz-Juárez; Hector Montoya-Fuentes; Helen Haydee Fernanda Ramírez-Plascencia; Irvin González-López; Jean Pierre González; Jesús Hernández; Joel Armando Vázquez-Pérez.; Jorge Salas-Hernández; José Antonio Enciso-Moreno; José Arturo Martínez-Orozco; José Esteban Muñoz-Medina; José de Jesús Nuñez-Contreras; Juan Bautista Chale-Dzul; Julissa Enciso-Ibarra; Luis Alberto Ochoa-Carrera; Margarita Matías-Florentino; Mario Mújica-Sánchez; Marissa Perez-Garcia; María Guadalupe Santiago-Mauricio; María Guadalupe de Jesús Mireles-Rivera; Nelly Sélem-Mojica; Pavel Isa; Ricardo Ciria Merce; Ricardo Grande; Rosa María Gutiérrez Rios; Santiago Ávila-Ríos; Selene Zárate; Susana Lopez; Verónica Mata-Haro; Victor Eduardo García-Arias; Víctor Hugo Borja-Aburto
EPI_ISL_1972570, EPI_ISL_2001050, EPI_ISL_2249246, EPI_ISL_2249247, EPI_ISL_2249249, EPI_ISL_2249252, EPI_ISL_2249253, EPI_ISL_2533924, EPI_ISL_2533926, EPI_ISL_2533927, EPI_ISL_2894563			
see above	Hospital Sharp	Microbial Genomics Laboratory	; Alejandra Garcia-Gasca; Alejandra García-Gasca; Alejandra Hernández-Terán; Alejandro Sánchez-Flores; Alfredo Herrera-Estrella; Alicia Ocaña-Mondragón; Andreu Comas-García; Angel Gustavo Salas-Lais; Antonio Loza Román; Bernardo Martínez-Miguel; Blanca Taboada; Brenda Irasema Maldonado-Meza; Bruno Gomez-Gil; Bruno Gómez-Gil; Carla Ivón Herrera-Najera; Carlos F. Arias; Celia Boukadida; Clara Esperanza Santacruz-Tinoco; Concepción Grajales-Muñiz; Consorcio Mexicano de Vigilancia Genómica (CoViGen-Mex). Authors (in alphabetical order): Julio Elias Alvarado-Yaah; Cristóbal Cháidez-Quiróz; Célida Duque Molina; Célida Martínez- Rodríguez; Daniel Fregoso-Rueda; Daniel Lira Morales; Eduardo Becerril-Vargas; Fernando Fontove-Herrera; Fidencio Mejía-Nepomuceno; Francisco Pulido; Gloria Elena Espinosa-Ayala; Gloria María Molina-Salinas; Gloria Vazquez; Hector Esteban Paz-Juárez; Hector Montoya-Fuentes; Helen Haydee Fernanda Ramírez-Plascencia; Irvin González-López; Jean Pierre González; Jesús Hernández; Joel Armando Vázquez-Pérez.; Jorge Salas-Hernández; José Antonio Enciso-Moreno; José Arturo Martínez-Orozco; José Esteban Muñoz-Medina; José de Jesús Nuñez-Contreras; Juan Bautista Chale-Dzul; Julissa Enciso-Ibarra; Luis Alberto Ochoa-Carrera; Margarita Matías-Florentino; Mario Mújica-Sánchez; Marissa Perez-Garcia; María Guadalupe Santiago-Mauricio; María Guadalupe de Jesús Mireles-Rivera; Nelly Sélem-Mojica; Pavel Isa; Ricardo Ciria Merce; Ricardo Grande; Rosa María Gutiérrez Rios; Santiago Ávila-Ríos; Selene Zárate; Susana Lopez; Verónica Mata-Haro; Victor Eduardo García-Arias; Víctor Hugo Borja-Aburto
EPI_ISL_2549576	Hospital Tuanku Fauziah	Institute for Medical Research, Infectious Disease Research Centre, National Institutes of Health, Ministry of Health Malaysia	Azizan MA; Kamel K; Mohd Zawawi Z; Ramly N; Robert F; Suppiah J; Thayan R
EPI_ISL_2628166	Hospital Universitari Arnau de Vilanova	Hospital Universitari Vall d'Hebron - Vall d'Hebron Institut de Recerca	Alejandra González-Sánchez; Andrés Antón; Ariadna Rando; Carla Castillo; Cristina Andrés; Damir Garcia-Cehic; Josep Quer; Juliana Esperalba; Maria Carmen Martin; Maria Gema Codina; Maria Piñana; Tomàs Pumarola
EPI_ISL_2352009, EPI_ISL_2352011, EPI_ISL_2516514, EPI_ISL_2516552, EPI_ISL_2516570, EPI_ISL_2516575, EPI_ISL_2637281, EPI_ISL_2637285, EPI_ISL_2637289, EPI_ISL_2637291, EPI_ISL_2637302, EPI_ISL_2637307, EPI_ISL_2637310, EPI_ISL_2637312, EPI_ISL_2637332, EPI_ISL_2637367, EPI_ISL_2637374, EPI_ISL_2637384, EPI_ISL_2861742, EPI_ISL_2861759, EPI_ISL_2861782			
see above	Hospital Universitari Bellvitge	Microbiology Department	Aida Gonzalez-Diaz; Carmen Ardanuy; Jordi Camara; Jordi Niubó; Laura Calatayud; M Angeles Domínguez; Miguel Fernandez-Huerta; Sara Marti
EPI_ISL_2657357, EPI_ISL_2820248, EPI_ISL_2824956, EPI_ISL_2824958,	Hospital Universitari Joan XXIII	Hospital Universitari Joan XXIII	Carla Martín; Clara Benavent; Cristina Gutiérrez; Ester Picó; Gemma Recio; Margarida Terrón; Natalia Bastón

EPI_ISL_2824960			
EPI_ISL_1908856, EPI_ISL_2047772, EPI_ISL_2047773, EPI_ISL_2284917, EPI_ISL_2284919, EPI_ISL_2391814, EPI_ISL_2391843, EPI_ISL_2391913, EPI_ISL_2458125, EPI_ISL_2493132, EPI_ISL_2493136, EPI_ISL_2628127, EPI_ISL_2628155, EPI_ISL_2628158, EPI_ISL_2712846, EPI_ISL_2828129, EPI_ISL_2828137, EPI_ISL_2828143, EPI_ISL_2828174, EPI_ISL_2885272, EPI_ISL_2885284, EPI_ISL_2885292, EPI_ISL_2885327, EPI_ISL_2885348, EPI_ISL_2885349			
see above	Hospital Universitari Vall d'Hebron - Vall d'Hebron Institut de Recerca	Hospital Universitari Vall d'Hebron - Vall d'Hebron Institut de Recerca	Alejandra González-Sánchez; Andrés Antón; Ariadna Rando; Carla Castillo; Cristina Andrés; Damir Garcia-Cehic; Josep Quer; Juliana Esperalba; Karen García; Maria Carmen Martin; Maria Gema Codina; Maria Piñana; Rodrigo Vásquez; Tomàs Pumarola
EPI_ISL_2775929, EPI_ISL_2775937, EPI_ISL_2775967, EPI_ISL_2775984	Hospital Universitari i Politècnic La Fe de València	SeqCOVID-SPAIN consortium/IBV(CSIC)	Ana Gil Brusola; Eva González Barberá; José Luis López Hontangas and SeqCOVID-SPAIN consortium; María Dolores Gómez Ruiz; Salvador Giner Almaraz
EPI_ISL_1941875, EPI_ISL_2091014	Hospital Universitario Cruces	Biocruces Bizkaia	Ana Belen de la Hoz; Mikel Gallego
EPI_ISL_2788595	Hospital of Southern Norway - Kristiansand, Department of Medical Microbiology	Norwegian Institute of Public Health, Department of Virology	Atiya R Ali; Debech Nadia; Engebretsen Serina Beate; Garcia Llorente Ignacio; Hilde Elshaug; Hilde Vollan; Jon Bråte; Kamilla Heddeland Instefjord; Karoline Bragstad; Kathrine Stene-Johansen; Line Victoria Moen; Marie Paulsen Madsen; Olav Hungnes; Pedersen Benedikte Nevjen; Rasmus Riis Kopperud
EPI_ISL_2860648, EPI_ISL_2860764	Hyogo Prefectural Institute of Public Health science	Pathogen Genomics Center, National Institute of Infectious Diseases	Denshi Takai; Etsuko Saito; Kenichi Ogita; Kentaro Itokawa; Kyoko Suzuki; Makoto Kuroda; Masanori Hashino; Masatsugu Chikahira; Miki Ogi; Rina Tanaka; Tomohiro Oshibe; Tsuyoshi Sekizuka; Yumi Akiyama
EPI_ISL_2790911, EPI_ISL_2790947, EPI_ISL_2790953, EPI_ISL_2790954	Hôpital	National Reference Center for Viruses of Respiratory Infections, Institut Pasteur, Paris	Angela Brisebarre; BéNéDicte Roquebert; Camille Capel; Christophe Malabat; Corinne Maufrais; Emmanuelle Permal; Etienne Simon-Lorière; Frédéric Lemoine; Louise Lefrançois; Marion Barbet; Maud Vanpeene; Méline Bizard; Nabil Gastli; Sylvie Behillil; Sylvie Van der Werf; Vincent Enouf
EPI_ISL_2228723	Hôpital Avicenne	Department of Virology, Henri Mondor University Hospital, Assistance Publique Hôpitaux de Paris, Université Paris-Est Créteil, INSERM U955	Alexandre Soulier; Christophe Rodriguez; Elisabeth Trawinski; Guillaume Gricourt; Jean-Michel Pawlotsky; Melissa N'Debi; Slim Fourati; Vanessa Demontant
EPI_ISL_2878324	Hôpital Cochin	Department of Virology, Henri Mondor University Hospital, Assistance Publique Hôpitaux de Paris, Université Paris-Est Créteil, INSERM U955	Alexandre Soulier; Christophe Rodriguez; Elisabeth Trawinski; Guillaume Gricourt; Jean-Michel Pawlotsky; Melissa N'Debi; Slim Fourati; Vanessa Demontant
EPI_ISL_2400536	Hôpitaux Robert Schuman	Laboratoire national de sante, Microbiology, Microbial Genomics Platform	Alain Hakim; Anke Wienecke-Baldacchino; Catherine Ragimbeau; Fatu Djabi; Jessica Tapp; Lise Pignon; Nawfal Faik; Raoul Salmon; Tamir Abdelrahman
EPI_ISL_2384050	IA State Hygienic Laboratory	Centers for Disease Control and Prevention Division of Viral Diseases, Pathogen Discovery	Alison Laufer Halpin; Ben L. Rambo-Martin; Clinton R. Paden; Dakota Howard; Darlene Wagner; Dave Wentworth; Dhwani Batra; Jasmine Padilla; Justin Lee; Katie Dillon; Krista Queen; Kristen Knipe; Kristine Lacek; Mark Burroughs; Matthew Schmerer; Mili Sheth; Peter Cook; Sam Shepard; Sarah Nobles; Shoshona Le; Suxiang Tong; Vivien Dugan; Yvette Unoarumhi
EPI_ISL_2189642, EPI_ISL_2189698, EPI_ISL_2189765, EPI_ISL_2189773	ICMR-National Institute of Virology	NCDC Delhi, Biotechnology Division	A Walimbe; A.Awhale; A.Titkare; B.Apoorva; G.Divekar; H.Kengale; Hema Gogia; Hemlata Lall; K P.Shinde; K.Iyengar; K.Korabu; Kalaiarasan Ponnusamy; M L Choudhary; M.Das; Mahesh S Dhar; Manoj K Singh; Meena Datta; Partha Rakshit; Preeti Madan; Priyanka Singh; R.Verma; Radhakrishnan V. S; Robin Marwal; S Jadhav; S.Bhorekar; S.Jadhav; S.Shelkande; Sandhya Kabra; Sujeet K Singh; T.Sanjeevi; U.Saha; Uma Sharma; V Malik; V.Autude; V.Vipat; Z. Sayyed
EPI_ISL_1704605, EPI_ISL_1704620, EPI_ISL_1704627, EPI_ISL_1704628, EPI_ISL_1704634, EPI_ISL_1841276, EPI_ISL_1841293, EPI_ISL_1841366, EPI_ISL_1928417, EPI_ISL_1928541, EPI_ISL_2546136, EPI_ISL_2546156, EPI_ISL_2546550, EPI_ISL_2546567, EPI_ISL_2546721, EPI_ISL_2546854, EPI_ISL_2547110, EPI_ISL_2547124, EPI_ISL_2547162, EPI_ISL_2547185, EPI_ISL_2547219, EPI_ISL_2547226			
see above	ICMR-National Institute of Virology - INSACOG	NIV Influenza	Dr. Varsha Potdar; Dr. Varsha Potdar and NIC Team
EPI_ISL_1825677	IHU Méditerranée Infection	IHU Méditerranée Infection	Bernard La Scola et al.
EPI_ISL_2801430, EPI_ISL_2801431, EPI_ISL_2801432	IHiT Pracownia Wirusologii Klinicznej	National Institute of Public Health - National Institute of Hygiene	Gierczyński Rafał; Sadkowska-Todys Małgorzata; Wołkowicz Tomasz
EPI_ISL_2758180	ILV Kärnten	Bergthaler laboratory, CeMM Research Center for Molecular Medicine of the Austrian Academy of Sciences	Andreas Bergthaler; Anna Schedl; Bekir Erguner; Benedikt Agerer; Christoph Bock; Fabian Amman; Jan Laine; Lukas Endler; Maelle Le Moing; Martin Senekowitsch; Matthew Thornton; Michael Schuster; Petr Triska; Thomas Penz
EPI_ISL_2420245	IN State Department of Health Laboratory Services	IN State Department of Health Laboratory Services	Brian Pope; Cassandra Campion; Jamie Yeadon; Kyle Brownlee; Lixia Liu; Mark Glazier; Melissa Hindenlang
EPI_ISL_2000617, EPI_ISL_2000619, EPI_ISL_2000620, EPI_ISL_2000621, EPI_ISL_2000625, EPI_ISL_2000626	INMI Lazzaro Spallanzani IRCCS	INMI Lazzaro Spallanzani IRCCS	A Di Caro; B Bartolini; CEM Gruber; E Giombini; F Messina; F Santini; G Bonfiglio; M Rueca; MR Capobianchi; O Butera
EPI_ISL_2878571, EPI_ISL_2878573, EPI_ISL_2878574, EPI_ISL_2878576, EPI_ISL_2878578, EPI_ISL_2878579, EPI_ISL_2878887, EPI_ISL_2879138, EPI_ISL_2880930, EPI_ISL_2881419			
see above	INSACOG Surveillance	INSACOG at CSIR Institute of Genomics and Integrative Biology	INSACOG
EPI_ISL_2503826	INSACOG-ARUNACHAL PRADESH	National Institute of Biomedical Genomics – INSACOG	Arindam Maitra; Lobsang Jampa; Mika Umpo; Nidhan Kumar Biswas; Saumitra Das; Sreedhar Chinnaswamy; Yompe Kamki
EPI_ISL_2877823, EPI_ISL_2877826	INSACOG-Assam	National Institute of Biomedical Genomics – INSACOG	Ajanta Sharma; Arindam Maitra; Kailash Chamuah; Lahari Saikia; Nidhan Kumar Biswas; Saumitra Das; Sreedhar Chinnaswamy
EPI_ISL_2547448	INSACOG-MEGHALAYA	National Institute of Biomedical Genomics – INSACOG	Arindam Maitra; E Marak; E Shadap; Nidhan Kumar Biswas; R. Lyngdoh; Saumitra Das; Sreedhar Chinnaswamy
EPI_ISL_2521601, EPI_ISL_2521605, EPI_ISL_2521606	INSACOG-Mizoram	National Institute of Biomedical Genomics – INSACOG	Arindam Maitra; Gracy Laldinmawii; N Senthil Kumar; Nidhan Kumar Biswas; Saumitra Das; Sreedhar Chinnaswamy; Swagnik Roy
EPI_ISL_2675337	INSACOG-NAGALAND	National Institute of Biomedical Genomics – INSACOG	Arindam Maitra; Chenole Keppen; Nidhan Kumar Biswas; Saumitra Das; Sreedhar Chinnaswamy; Vinotsole Khamo; Wetetsho Kapfo
EPI_ISL_2878033	INSACOG-Tripura	National Institute of Biomedical Genomics – INSACOG	Apurba Sarkar; Arindam Maitra; Nidhan Kumar Biswas; Saikat Majumder; Saumitra Das; Sreedhar Chinnaswamy; Tapan Majumdar
EPI_ISL_1589867, EPI_ISL_1589872, EPI_ISL_2503246, EPI_ISL_2503538, EPI_ISL_2503615, EPI_ISL_2503641, EPI_ISL_2503739, EPI_ISL_2548465, EPI_ISL_2548631, EPI_ISL_2548641, EPI_ISL_2548684, EPI_ISL_2548904, EPI_ISL_2549015, EPI_ISL_2675940, EPI_ISL_2675971, EPI_ISL_2676126, EPI_ISL_2676176, EPI_ISL_2676229, EPI_ISL_2676273, EPI_ISL_2676309, EPI_ISL_2676321, EPI_ISL_2676341, EPI_ISL_2676343, EPI_ISL_2676347, EPI_ISL_2860944, EPI_ISL_2860959			
see above	INSACOG-WB	National Institute of Biomedical Genomics – INSACOG	Ajay Chakraborti; Arindam Maitra; Bhaswati Bandyopadhyay; Nidhan Kumar Biswas; Saumitra Das; Sreedhar Chinnaswamy; Tamal Ghosh
EPI_ISL_2820952	INSIDE DIAGNÓSTICOS	Instituto Butantan	Antonio Jorge Martins; Claudia Renata dos Santos Barros; David Schlesinger; Debora Botequio Moretti; Dimas Tadeu Covas; Elaine Cristina Marqueze; Elaine Vieira Santos; Evandra Strazza Rodrigues; Heidge Fukumasu; Jayme Augusto de Souza-Neto; José Salvatore Leister Patané; Luiz Alcantara; Luiz Lehmann Coutinho; Maria Carolina Elias; Maurício Lacerda Nogueira; Rafael dos Santos Bezerra; Raul Machado Neto; Rejane Maria Tommasini Grotto; Ricardo Haddad; Sandra Coccuzzo Sampaio Vessoni; Simone Kashima; Svetoslav Nanev Slavov; Vincent Louis Viala
EPI_ISL_2895665, EPI_ISL_2895666, EPI_ISL_2895667, EPI_ISL_2895669, EPI_ISL_2895670, EPI_ISL_2895671, EPI_ISL_2895673, EPI_ISL_2895674			
see above	INSPI-CRN DE INFLUENZA Y OTROS VIRUS RESPIRATORIOS	NIC-INSPI	Alfredo Bruno; Domenica de Mora.; Jimmy Garcés; Johanna Laines; Lizbeth Patiño; Manuel Gonzalez; Maritza Olmedo; Michelle Páez
EPI_ISL_2577027, EPI_ISL_2716755, EPI_ISL_2716756, EPI_ISL_2716763, EPI_ISL_2716768, EPI_ISL_2716772, EPI_ISL_2716776, EPI_ISL_2716778, EPI_ISL_2774164, EPI_ISL_2774212, EPI_ISL_2774239			
see above	IRCCS San Gallicano Dermatological Institute	IRCCS Regina Elena National Cancer Institute	Aldo Morrone; Alice Massacci; Antonio Federico; Eleonora Sperandio; Fabrizio Ensoli; Francesca De Nicola; Frauke Goeman; Fulvia Fraticelli; Fulvia Pimpinelli; Gennaro Ciliberto; Giovanna D'agosto; Giovanni Blandino; Giulia Orlandi; Ilaria Cavallo; Ludovica Ciuffreda; Martina Diano; Matteo Pallocca; Maurizio Fanciulli; Sabrina Strano; Sara Donzelli; Sara Petrolo; Valentina Ricca
EPI_ISL_2886166	IVRCRD Puskesmas Cebongan	Institute for Vector and Reservoir Control, Research and Development (IVRCRD)- National Institute of Health Research and Development (NIHRD), Indonesia	Aprilia Safitri; Arief Mulyono; Arif Suryo Prasetyo; Aryo Ardanto; Ayu Pradipta Pratiwi; Dimas Bagus Wicaksono Putro; Dwi Susilo; Esti Rahardianingtyas; Evi sulistyorini; Hana Apsari Pawestri; Joko Waluyo; Kusumaningtyas Sekar Negari; Lulus Susanti; Marjianto; Mega Tyas Prihatin; Muhidin; Mujiyanto; Nur Hidayati; Restu Choirul Saban; Ristiyanto; Sidiq Setyo Nugroho; Siti Alfiah; Sri Wahyuni Handayani; Subangkit; Tika Fiona Sari; Triwibowo Ambar Garjito; Vivi Setiawaty; Wimbi Kartika Ratnasari; Yusnita Mirna Anggraeni
EPI_ISL_2886160, EPI_ISL_2886161	IVRCRD Puskesmas Muntilan 1	Institute for Vector and Reservoir Control, Research and Development (IVRCRD)- National Institute of Health Research and Development (NIHRD), Indonesia	Aprilia Safitri; Arief Mulyono; Arif Suryo Prasetyo; Aryo Ardanto; Ayu Pradipta Pratiwi; Dimas Bagus Wicaksono Putro; Dwi Susilo; Esti Rahardianingtyas; Evi sulistyorini; Hana Apsari Pawestri; Joko Waluyo; Kusumaningtyas Sekar Negari; Lulus Susanti; Marjianto; Mega Tyas Prihatin; Muhidin; Mujiyanto; Nur Hidayati; Restu Choirul Saban; Ristiyanto; Sidiq Setyo Nugroho; Siti Alfiah; Sri Wahyuni Handayani; Subangkit; Tika Fiona Sari; Triwibowo Ambar Garjito; Vivi Setiawaty; Wimbi Kartika Ratnasari; Yusnita Mirna Anggraeni
EPI_ISL_2886164	IVRCRD Puskesmas Welahan 1	Institute for Vector and Reservoir Control, Research and Development (IVRCRD)- National Institute of Health Research and Development (NIHRD), Indonesia	Aprilia Safitri; Arief Mulyono; Arif Suryo Prasetyo; Aryo Ardanto; Ayu Pradipta Pratiwi; Dimas Bagus Wicaksono Putro; Dwi Susilo; Esti Rahardianingtyas; Evi sulistyorini; Hana Apsari Pawestri; Joko Waluyo; Kusumaningtyas Sekar Negari; Lulus Susanti; Marjianto; Mega Tyas Prihatin; Muhidin; Mujiyanto; Nur Hidayati; Restu Choirul Saban; Ristiyanto; Sidiq Setyo Nugroho; Siti Alfiah; Sri Wahyuni Handayani; Subangkit; Tika Fiona Sari; Triwibowo Ambar Garjito; Vivi Setiawaty; Wimbi Kartika Ratnasari; Yusnita Mirna Anggraeni
EPI_ISL_2886157, EPI_ISL_2886159	IVRCRD RSPAW	Institute for Vector and Reservoir Control, Research and Development (IVRCRD)- National Institute of Health Research and Development (NIHRD), Indonesia	Aprilia Safitri; Arief Mulyono; Arif Suryo Prasetyo; Aryo Ardanto; Ayu Pradipta Pratiwi; Dimas Bagus Wicaksono Putro; Dwi Susilo; Esti Rahardianingtyas; Evi sulistyorini; Hana Apsari Pawestri; Joko Waluyo; Kusumaningtyas Sekar Negari; Lulus Susanti; Marjianto; Mega Tyas Prihatin; Muhidin; Mujiyanto; Nur Hidayati; Restu Choirul Saban; Ristiyanto; Sidiq Setyo Nugroho; Siti Alfiah; Sri Wahyuni Handayani; Subangkit; Tika Fiona Sari; Triwibowo Ambar Garjito; Vivi Setiawaty; Wimbi Kartika Ratnasari; Yusnita Mirna Anggraeni
EPI_ISL_2812801, EPI_ISL_2812804	Ifo Regina Elena	National Institute for Infectious Diseases (INMI) L. Spallanzani I.R.C.C.S	A Di Caro; B Bartolini; CEM Gruber; E Giombini; F Messina; F Santini; G Bonfiglio; M Rueca; MR Capobianchi; O Butera
EPI_ISL_2885452	Illinois Department of Public Health - Springfield Lab	Illinois Department of Public Health - Springfield Lab	Bryan Sim; Gordon McCall
EPI_ISL_2533925	Imágenes Diagnósticas de Mazatlán	Microbial Genomics Laboratory	; Alejandra García-Gasca; Alejandra Hernández-Terán; Alejandro Sánchez-Flores; Alfredo Herrera-Estrella; Alicia Ocaña-Mondragón; Andreu Comas-García; Angel Gustavo Salas-Lais; Antonio Loza Román; Bernardo Martínez-Miguel; Blanca Taboada; Brenda Irasema Maldonado-Meza; Bruno Gómez-Gil; Carla Ivón Herrera-Najera; Carlos F. Arias; Celia Boukadida; Clara Esperanza Santacruz-Tinoco; Concepción Grajales-Muñiz; Consorcio Mexicano de Vigilancia Genómica (CoViGen-Mex). Authors (in alphabetical order): Julio Elias Alvarado-Yaah; Cristóbal Cháidez-Quiróz; Célida Duque Molina; Célida Martínez- Rodríguez; Daniel Fregoso-Rueda; Daniel Lira Morales; Eduardo Becerril-Vargas; Fernando Fontove-Herrera; Fidencio Mejía-Nepomuceno; Francisco Pulido; Gloria Elena Espinosa-Ayala; Gloria María Molina-Salinas; Gloria Vazquez; Hector Esteban Paz-Juárez; Hector Montoya-Fuentes; Helen Haydee Fernanda Ramírez-Plascencia; Irvin González-López; Jean Pierre González; Jesús Hernández; Joel Armando Vázquez-Pérez.; Jorge Salas-Hernández; José Antonio Enciso-Moreno; José Arturo Martínez-Orozco; José Esteban Muñoz-Medina; José de Jesús Nuñez-Contreras; Juan Bautista Chale-Dzul; Julissa Enciso-Ibarra; Luis Alberto Ochoa-Carrera; Margarita Matías-Florentino; Mario Mújica-Sánchez; Marissa Perez-Garcia; María Guadalupe Santiago-Mauricio; María Guadalupe de Jesús Mireles-Rivera; Nelly Sélem-Mojica; Pavel Isa; Ricardo Ciria Merce; Ricardo Grande; Rosa María Gutiérrez Rios; Santiago Ávila-Ríos; Selene Zárate; Susana Lopez; Verónica Mata-Haro; Victor Eduardo García-Arias; Víctor Hugo Borja-Aburto
EPI_ISL_2603612,	InDRE	Instituto Nacional de Medicina Genomica	Cedro-Tanda A; Garcia-Cardenas FJ; Gisela Barrera-Badillo; Gonzalez-Barrera D; Gonzalez-Woge MA; Herrera-Montalvo LA.; Hidalgo-Miranda A; Irma Lopez-Martinez; Jose Ernesto Ramirez González; Mendoza-Vargas A; Munguia-Garza P; Rangel-DeLeon

EPI_ISL_2603635			D; Reyes-Grajeda JP
EPI_ISL_2570805, EPI_ISL_2570806	Indrapura Field Hospital Surabaya	Institute of Tropical Disease, Universitas Airlangga	Aldise M Nastri; Febria Rachmanita; Gatot Soegiarto; Herlin Ferliana; Jezzy R Dewantari; Kazufumi Shimizu; Krisnoadi Rahardjo; Laksmi Wulandari; Maria I Lusida; Resti Yudhawati; Rima R Prasetya; Soetjipto; Yasuko Mori
EPI_ISL_2660359	Infectious Disease Program, Broad Institute of Harvard and MIT	Infectious Disease Program, Broad Institute of Harvard and MIT	A.E.; Adams, G.; Anahtar, M.; B.L.; B.W.; Bauer, M.; Birren; Branda, J.; Carter, A.; Cerrato, C.; Chaluvadi, S.; Chapman; Cusick, C.; D.J.; DeRuff, K.; Flowers, K.; Gallagher, G.; Gladden-Young, A.; Gnirke, A.; Harris, J.; J.E.; K.J.; LaRocque, R.; Lagerborg, K.; Lemieux; Lin; Loreth, C.; MacInnis; Neumann, A.; Normandin, E.; P.C.; Park; Pierce, V.; Reilly, S.; Rosenberg, E.; Rudy, M.; Ryan, E.; S.B.; Sabeti; Shaw, B.; Siddle; Slater, D.; Smole, S.; Tomkins-Tinch, C.; Turbett, S.
EPI_ISL_2816227, EPI_ISL_2816229, EPI_ISL_2816240, EPI_ISL_2816241	Infectious Diseases Hospital No 2	WHO National Influenza Centre Russian Federation	Andrey Komissarov; Artem Fadeev; Daria Danilenko; Dmitry Lioznov; Elena Nabieva; Georgii Bazykin; Kirill Varchenko; Ksenia Safina; Kseniya Komissarova; Maria Pisareva; Mikhail Bakaev; Nikita Yolshin; Oula Mansour; Tamila Musaeva; Veronika Eder
EPI_ISL_2087219, EPI_ISL_2087232, EPI_ISL_2087519, EPI_ISL_2087703, EPI_ISL_2876082	Infinity Biologix	Centers for Disease Control and Prevention Division of Viral Diseases, Pathogen Discovery	Adrian Paskey; Benjamin Rambo-Martin; Chirayu Goswami; Christian Bixby; Christopher Gulvick; Clinton R. Paden; Dakota Howard; Darlene Wagner; Dhwani Batra; Duncan MacCannell; Jason Caravas; Jonathan Schultz; Kara Moser; Matthew Schmerer; Peter W. Cook; Robin Grimwood; Russ Hager; Scott Sammons; Shatavia Morrison; Yihe Wang; Yvette Unoarumhi
EPI_ISL_2820841, EPI_ISL_2820850, EPI_ISL_2820872, EPI_ISL_2820873	Innlandet Hospital Trust, Division Lillehammer, Department for Medical Microbiology	Norwegian Institute of Public Health, Department of Virology	Atiya R Ali; Debech Nadia; Engebretsen Serina Beate; Garcia Llorente Ignacio; Hilde Elshaug; Hilde Vollan; Jon Bråte; Kamilla Heddeland Instefjord; Karoline Bragstad; Kathrine Stene-Johansen; Line Victoria Moen; Marie Paulsen Madsen; Olav Hungnes; Pedersen Benedikte Nevjen; Rasmus Riis Kopperud
EPI_ISL_2466400	Inst. for Med. Virology, University Hospital Frankfurt, Goethe University Frankfurt	Inst. for Med. Virology, University Hospital Frankfurt, Goethe University Frankfurt	Goetsch, U.; Gottschalk; H.F.; Pallas, C.; R. and Ciesek, S.; Rabenau; Toptan, T.; Widera, M.; Wilhelm, A.
EPI_ISL_2110643	Institut Pasteur du Maroc	Functional Genomics platform/CNRST	Abdelkrim Meziane Bellefquih; Alaoui Sanaa-amine; Chouati Taha; EL FAHIME Elmostafa; Elannaz Hicham; Ennibi Khalid; Hemlali Mouhsine; MERABET MOUAD; Maaroufi Abderrahmane; Melloul Marouane; NOURLIL Jalal; Touil Nadia; Youbi Mohammed
EPI_ISL_2774076	Institute for Developing Science and Health Initiatives (ideSHi)	IEDCR-ideSHi-icddr,b	Firdausi Qadri; Hassan Afrad; Manjur Hossain Khan; Sadia Rahman; Tahmina Shirin
EPI_ISL_2758188	Institute for Laboratory Diagnostics and Microbiology, Klinikum Klagenfurt am Wörthersee	Bergthaler laboratory, CeMM Research Center for Molecular Medicine of the Austrian Academy of Sciences	Andreas Bergthaler; Anna Schedl; Bekir Erguner; Benedikt Agerer; Christoph Bock; Fabian Amman; Jan Laine; Lukas Endler; Maelle Le Moing; Martin Senekowitsch; Matthew Thornton; Michael Schuster; Petr Triska; Thomas Penz
EPI_ISL_2379433, EPI_ISL_2379443, EPI_ISL_2379650, EPI_ISL_2429136, EPI_ISL_2429137, EPI_ISL_2429138, EPI_ISL_2429141, EPI_ISL_2819024, EPI_ISL_2819571, EPI_ISL_2819919			
see above	Institute for Medical Research, Infectious Disease Research Centre, National Institutes of Health, Ministry of Health Malaysia	Institute for Medical Research, Infectious Disease Research Centre, National Institutes of Health, Ministry of Health Malaysia	Azizan MA; Kamel K; Mohd Zawawi Z; Ramly N; Robert F; Suppiah J; Thayan R
EPI_ISL_2678096, EPI_ISL_2678097	Institute for Urban Disease Control and Prevention	COVID-19 Network Investigations (CONI) Alliance	Amornmas Kongklieng; Angkana Huang; Anthony R. Jones; Arporn Wangwiwatsin; Bhakbhoom Panthan; Chonticha Klungtong; Duangkamon Loesbanluechai; Ekawat Pasomsub; Elizabeth Batty; Insee Sensorn; Janjira Thaipadungpanit; Kamolthip Atsawawaranunt; Khajohn Joonlasak; Kingkan Rakmanee; Krittikorn Kumpornsin; Namfon Kotanan; Prayuth Kaewmalang; Pukkaporn Parnwijitkul; Stefan Fernandez; Thanat Chookajorn; Theerarat Kochakarn; Treewat Watthanachockchai; Vichan Pawun; Wasun Chantratita; Wudtichai Manasatienkij
EPI_ISL_2180455, EPI_ISL_2284885, EPI_ISL_2284886, EPI_ISL_2284887, EPI_ISL_2284888, EPI_ISL_2284889, EPI_ISL_2284890, EPI_ISL_2284891, EPI_ISL_2284892, EPI_ISL_2304461, EPI_ISL_2304462, EPI_ISL_2304463, EPI_ISL_2304465, EPI_ISL_2399441, EPI_ISL_2399444, EPI_ISL_2399445, EPI_ISL_2399446, EPI_ISL_2399448, EPI_ISL_2399449, EPI_ISL_2399452, EPI_ISL_2399453, EPI_ISL_2399454, EPI_ISL_2399455, EPI_ISL_2399456, EPI_ISL_2434139, EPI_ISL_2434140, EPI_ISL_2434141, EPI_ISL_2434142, EPI_ISL_2434143, EPI_ISL_2600366, EPI_ISL_2600367, EPI_ISL_2600372, EPI_ISL_2774038, EPI_ISL_2774039, EPI_ISL_2774040, EPI_ISL_2774042, EPI_ISL_2774043, EPI_ISL_2774044, EPI_ISL_2774045, EPI_ISL_2774046, EPI_ISL_2774048, EPI_ISL_2774049, EPI_ISL_2774050, EPI_ISL_2774051, EPI_ISL_2774052, EPI_ISL_2774053, EPI_ISL_2774054, EPI_ISL_2774055, EPI_ISL_2774056, EPI_ISL_2774057, EPI_ISL_2774058, EPI_ISL_2774059, EPI_ISL_2774060, EPI_ISL_2774061, EPI_ISL_2774062, EPI_ISL_2774063, EPI_ISL_2774064, EPI_ISL_2774065, EPI_ISL_2774066, EPI_ISL_2774067, EPI_ISL_2774068, EPI_ISL_2774069, EPI_ISL_2774070, EPI_ISL_2774071, EPI_ISL_2774072, EPI_ISL_2774073, EPI_ISL_2774074, EPI_ISL_2774075			
see above	Institute of Epidemiology, Disease Control and Research (IEDCR)	IEDCR-ideSHi-icddr,b	Firdausi Qadri; Hassan Afrad; Manjur Hossain Khan; Sadia Rahman; Tahmina Shirin
EPI_ISL_1915436, EPI_ISL_1915437, EPI_ISL_1915439, EPI_ISL_1938476, EPI_ISL_1938477	Institute of Epidemiology, Disease Control and Research (IEDCR)	Institute for Developing Science and Health Initiatives (ideSHi)	Fidausi Qadri; Hassan Afrad; Sadia Rahman; Tahmina Shirin
EPI_ISL_2492325, EPI_ISL_2644451, EPI_ISL_2886575, EPI_ISL_2886903	Institute of Microbiology and Immunology, Faculty of Medicine, University of Ljubljana	Institute of Microbiology and Immunology, Faculty of Medicine, University of Ljubljana	Alen Suljič; Andraž Celar; Dominika Šturm; Doroteja Vlaj; Mario Poljak; Matic Brvar; Miša Korva; Patricija Pozvek; Samo Zakotnik; Tatjana Avšič – Županc; Tomaž Mark Zorec; Špela Pleh
EPI_ISL_2802010, EPI_ISL_2894423, EPI_ISL_2894428, EPI_ISL_2894436, EPI_ISL_2894439, EPI_ISL_2894448, EPI_ISL_2894456, EPI_ISL_2894457, EPI_ISL_2894459, EPI_ISL_2894463, EPI_ISL_2894465, EPI_ISL_2894466, EPI_ISL_2894475, EPI_ISL_2894477, EPI_ISL_2894484, EPI_ISL_2894486			
see above	Institute of Virology, Biomedical Research Center of the Slovak Academy of Sciences, Bratislava	Faculty of Natural Sciences, Comenius University, Bratislava	Boris Klempa; Brona Brejova; Jozef Nosek; Juraj Kopacek; Kristina Borsova; Lubomira Lukacikova; Martina Lickova; Martina Nebohacova; Monika Slavikova; Sabina Fumacova Havlikova; Tomas Vinar; Viktoria Cabanova; Viktoria Hodorova
EPI_ISL_1805024	Institute of Virology, Medical Center, University of Freiburg, Freiburg, Germany	Institute of Virology, Clinial Virus Genomics, Medical Center, University of Freiburg, Freiburg, Germany	Hajo Grundmann; Jonas Fuchs; Lena Jaki; Lisa Kern; Marcus Panning; Sandra Reuter
EPI_ISL_2777222, EPI_ISL_2777224, EPI_ISL_2777225, EPI_ISL_2777226, EPI_ISL_2777229, EPI_ISL_2777230, EPI_ISL_2777231, EPI_ISL_2777232, EPI_ISL_2777233			
see above	Instituto Nacional de Cancerología	Centro de Investigación en Enfermedades Infecciosas (CIENI), Instituto Nacional de Enfermedades Respiratorias (INER)	Alejandra Garcia-Gasca; Alejandra Hernández-Terán; Alejandro Sanchez-Flores; Alfredo Herrera-Estrella; Alicia Ocaña-Mondragón; Andreu Comas-García; Angel Gustavo Salas-Lais; Antonio Loza Román; Bernardo Martínez-Miguel; Blanca Taboada; Brenda Irasema Maldonado-Meza; Bruno Gomez-Gil; Carla Ivón Herrera-Najera; Carlos F. Arias; Celia Boukadida; Clara Esperanza Santacruz-Tinoco; Concepción Grajales-Muñiz; Consorcio Mexicano de Vigilancia Genómica (CoViGen-Mex). Authors (in alphabetical order): Julio Elias Alvarado-Yaah; Cristóbal Cháidez-Quiróz; Célida Duque Molina; Célida Martínez- Rodríguez; Daniel Fregoso-Rueda; Daniel Lira Morales; Eduardo Becerril-Vargas; Fernando Fontove-Herrera; Fidencio Mejía-Nepomuceno; Francisco Pulido; Gloria Elena Espinosa-Ayala; Gloria María Molina-Salinas; Gloria Vazquez; Hector Esteban Paz-Juárez; Hector Montoya-Fuentes; Helen Haydee Fernanda Ramírez-Plascencia; Irvin González-López; Jean Pierre González; Joel Armando Vázquez-Pérez.; Jorge Salas-Hernández; José Antonio Enciso-Moreno; José Arturo Martínez-Orozco; José Esteban Muñoz-Medina; José de Jesús Nuñez-Contreras; Juan Bautista Chale-Dzul; Julissa Enciso-Ibarra; Kathia Elizabeth Tapia-Diaz; Luis Alberto Ochoa-Carrera; Margarita Matías-Florentino; Mario Mújica-Sánchez; Marissa Pérez-García; María Guadalupe Santiago-Mauricio; María Guadalupe de Jesús Mireles-Rivera; Nelly Sélem-Mojica; Pavel Isa; Ricardo Ciria Merce; Ricardo Grande; Rosa María Gutierrez Rios; Santiago Ávila-Ríos; Selene Zárate; Susana Lopez; Victor Eduardo Garcia-Arias; Víctor Hugo Borja-Aburto
EPI_ISL_2777214, EPI_ISL_2777216	Instituto Nacional de Enfermedades Respiratorias (INER)	Centro de Investigación en Enfermedades Infecciosas (CIENI), Instituto Nacional de Enfermedades Respiratorias (INER)	Alejandra Garcia-Gasca; Alejandra Hernández-Terán; Alejandro Sanchez-Flores; Alfredo Herrera-Estrella; Alicia Ocaña-Mondragón; Andreu Comas-García; Angel Gustavo Salas-Lais; Antonio Loza Román; Bernardo Martínez-Miguel; Blanca Taboada; Brenda Irasema Maldonado-Meza; Bruno Gomez-Gil; Carla Ivón Herrera-Najera; Carlos F. Arias; Celia Boukadida; Clara Esperanza Santacruz-Tinoco; Concepción Grajales-Muñiz; Consorcio Mexicano de Vigilancia Genómica (CoViGen-Mex). Authors (in alphabetical order): Julio Elias Alvarado-Yaah; Cristóbal Cháidez-Quiróz; Célida Duque Molina; Célida Martínez- Rodríguez; Daniel Fregoso-Rueda; Daniel Lira Morales; Eduardo Becerril-Vargas; Fernando Fontove-Herrera; Fidencio Mejía-Nepomuceno; Francisco Pulido; Gloria Elena Espinosa-Ayala; Gloria María Molina-Salinas; Gloria Vazquez; Hector Esteban Paz-Juárez; Hector Montoya-Fuentes; Helen Haydee Fernanda Ramírez-Plascencia; Irvin González-López; Jean Pierre González; Joel Armando Vázquez-Pérez.; Jorge Salas-Hernández; José Antonio Enciso-Moreno; José Arturo Martínez-Orozco; José Esteban Muñoz-Medina; José de Jesús Nuñez-Contreras; Juan Bautista Chale-Dzul; Julissa Enciso-Ibarra; Kathia Elizabeth Tapia-Diaz; Luis Alberto Ochoa-Carrera; Margarita Matías-Florentino; Mario Mújica-Sánchez; Marissa Pérez-García; María Guadalupe Santiago-Mauricio; María Guadalupe de Jesús Mireles-Rivera; Nelly Sélem-Mojica; Pavel Isa; Ricardo Ciria Merce; Ricardo Grande; Rosa María Gutierrez Rios; Santiago Ávila-Ríos; Selene Zárate; Susana Lopez; Victor Eduardo Garcia-Arias; Víctor Hugo Borja-Aburto
EPI_ISL_2617181, EPI_ISL_2617182, EPI_ISL_2841652, EPI_ISL_2841653, EPI_ISL_2841654	Instituto Nacional de Investigação em Saúde	CERI, Centre for Epidemic Response and Innvoation, Stellenbosch University and KRISP, KZN Research Innovation and Sequencing Platform, UKZN.	Afonso P; David K; Emmanuel SJ; Freitas RH; Giandhari J; Inglês L; Lutucuta S; Miranda J; Morais J; Mufinda M; Naidoo Y; Neto Z; Paulo A Carralero RR Paixão JP; Pereira A; Pillay S; Tegally H; Wilkinson E; de Oliveira T
EPI_ISL_2778017	Instituto Nacional de Medicina Genomica	Centro de Investigación en Enfermedades Infecciosas (CIENI), Instituto Nacional de Enfermedades Respiratorias (INER)	Avila-Rios S; Boukadida C; Cedro-Tanda A; Herrera-Montalvo LA; Hidalgo-Miranda A; Matias-Florentino M; Mendoza-Vargas A; Perez-Garcia M; Reyes-Grajeda JP; Reyes-Teran G
EPI_ISL_2692836, EPI_ISL_2692840, EPI_ISL_2692845, EPI_ISL_2894251, EPI_ISL_2894252, EPI_ISL_2894256, EPI_ISL_2894257, EPI_ISL_2894258, EPI_ISL_2894260, EPI_ISL_2894262, EPI_ISL_2894263, EPI_ISL_2894270, EPI_ISL_2894276, EPI_ISL_2894277, EPI_ISL_2894281, EPI_ISL_2894283, EPI_ISL_2894288, EPI_ISL_2894289, EPI_ISL_2894308, EPI_ISL_2894310, EPI_ISL_2894317, EPI_ISL_2894318, EPI_ISL_2894328, EPI_ISL_2894401			
see above	Instituto Nacional de Medicina Genomica	Instituto Nacional de Medicina Genomica	Abraham Campos-Romero; Cedro-Tanda A; Cisneros- Villanueva M; Escobar-Arrazola; Gonzalez-Barrera D; Herrera-Montalvo LA.; Hidalgo-Miranda A; Luna-Ruiz Marco; M; Mendoza-Vargas A; Moreno-Camacho José Luis; Munguia-Garza P; Ramirez-Vega O; Rangel-DeLeon D; Reyes-Grajeda JP; Rodriguez-Gallegos Jorge
EPI_ISL_2340461	Instituto Nacional de Saude (INSA)	Instituto Nacional de Saude (INSA) and BioSystems & Integrative Sciences Institute (BioISI) Genomics Unit, FCUL	Borges et al
EPI_ISL_2290665, EPI_ISL_2290666	Invicta	1. National Institute of Public Health - National Institute of Hygiene, Warsaw, Poland 2. Biobank Lab, University of Lodz 3. Laboratory of Respiratory Viruses, Teaching and Clinical Center of the Medical University of Lodz	Dominik Strapagiel; Izabela Dróżdż; Jakub Lach; Katarzyna Zacharczuk; Klaudyna Królikowska; Maciej Borowiec; Magdalena Nowakowska; Magdalena Traczyk-Borszyńska; Marcin Słomka; Marta Sobalska-Kwapis; Małgorzata Sadkowska-Todys; Tomasz Płoszaj; Tomasz Wołkowicz
EPI_ISL_2815329, EPI_ISL_2815330	Ipoh Public Health Laboratory (MKAI), Ministry of Health Malaysia	Institute for Medical Research, Infectious Disease Research Centre, National Institutes of Health, Ministry of Health Malaysia	Azizan MA; Kamel K; Mohd Zawawi Z; Ramly N; Robert F; Suppiah J; Thayan R
EPI_ISL_2645119	Islab, Pohjois-Savon aluelaboratorio	Expert Microbiology, National Institute for Health and Welfare	Carita Savolainen-Kopra; Erika Lindh; Haider al-Hello; Jani Halkilahti; Kirsi Liitsola; Niina Ikonen; Olli Vapalahti; Pekka Ellonen; Phuoc Truong; Päivi Laurila; Ravi Kant; Sari Hannula; Soile Blomqvist; Teemu Smura
EPI_ISL_2183026, EPI_ISL_2183029, EPI_ISL_2183057, EPI_ISL_2183059, EPI_ISL_2183060, EPI_ISL_2183112, EPI_ISL_2183114, EPI_ISL_2183116, EPI_ISL_2183420, EPI_ISL_2183422, EPI_ISL_2183424, EPI_ISL_2183427, EPI_ISL_2183431, EPI_ISL_2183613, EPI_ISL_2183691, EPI_ISL_2183692, EPI_ISL_2183694, EPI_ISL_2183702, EPI_ISL_2183704, EPI_ISL_2183721, EPI_ISL_2183727, EPI_ISL_2183749, EPI_ISL_2183779, EPI_ISL_2183805, EPI_ISL_2636189, EPI_ISL_2636241, EPI_ISL_2636298, EPI_ISL_2636326, EPI_ISL_2636333, EPI_ISL_2636353, EPI_ISL_2636354, EPI_ISL_2636356, EPI_ISL_2636358, EPI_ISL_2636361, EPI_ISL_2636363, EPI_ISL_2636365, EPI_ISL_2636367, EPI_ISL_2636368, EPI_ISL_2636371, EPI_ISL_2636373, EPI_ISL_2636377, EPI_ISL_2636385, EPI_ISL_2636387, EPI_ISL_2636396, EPI_ISL_2636399			
see above	Israel Central Virology laboratory	Israel National Consortium for SARS-CoV-2 sequencing	Dana Bar-Ilan; Efrat Dahan Bucris; Efrat Glick-Saar; Ella Mendelson; Gideon Rechavi; Michal Mandelboim; Miranda Geva; Neta Zuckerman; Netanel Abu; Omri Nayshool; Oran Erster; Orna Mor
EPI_ISL_2844483	Istituto Zooprofilattico Sperimentale del Mezzogiorno	TIGEM	Antonio Grimaldi Patrizia Annunziata Francesco Panariello Biancamaria Pierri Claudia Tiberio Teresa Giuliano Valentina Bouche Chiara Colantuono Maria Concetta Cuomo Denise Di Concilio Lucio Di Filippo Anna Manfredi Marcello Salvi Antonio Limone Luigi Atripaldi Pellegrino Cerino Andrea Ballabio Davide Cacchiarelli
EPI_ISL_2641798,	Istituto Zooprofilattico Sperimentale del Mezzogiorno	Telethon Institute of Genetics and Medicine (TIGEM)	Antonio Grimaldi Patrizia Annunziata Francesco Panariello Biancamaria Pierri Claudia Tiberio Teresa Giuliano Valentina Bouche Chiara Colantuono Maria Concetta Cuomo Denise Di Concilio Lucio Di Filippo Anna Manfredi Marcello Salvi Antonio Limone

EPI_ISL_2641928, EPI_ISL_2641992, EPI_ISL_2641993			Luigi Atripaldi Pellegrino Cerino Andrea Ballabio Davide Cacchiarelli
EPI_ISL_2725330	Istituto Zooprofilattico Sperimentale del Mezzogiorno - Azienda Ospedaliera "Pugliese Ciaccio" di Catanzaro	Telethon Institute of Genetics and Medicine (TIGEM)	Antonio Grimaldi Patrizia Annunziata Francesco Panariello Biancamaria Pierri Claudia Tiberio Teresa Giuliano Valentina Bouche Chiara Colantuono Maria Concetta Cuomo Denise Di Concilio Lucio Di Filippo Anna Manfredi Pasquale Minchella Marcello Salvi Antonio Limone Luigi Atripaldi Pellegrino Cerino Andrea Ballabio Davide Cacchiarelli
EPI_ISL_2171266	Janamaitri Hospital	Molecular and Genomics Research Lab, Dhulikhel Hospital, Kathmandu University Hospital	Dipesh Tamrakar; Meghnath Dhimal; Navin Adhikari; Nishan Katuwal; Pradip Gyanwali; Rajeev Shrestha; Saroj Bhattarai; Surendra Kumar Madhup
EPI_ISL_1753676, EPI_ISL_1922480, EPI_ISL_2422370	Jessa	Jessa	Berden et al. on behalf of the Jessa_cmdLab; Cruys et al. on behalf of the Jessa_cmdLab
EPI_ISL_2248930	Joaquim Chaves Saude	Instituto Nacional de Saude (INSA)	Borges et al
EPI_ISL_2550714, EPI_ISL_2550731	Johor Bahru Public Health Laboratory	Institute for Medical Research, Infectious Disease Research Centre, National Institutes of Health, Ministry of Health Malaysia	Azizan MA; Kamel K; Mohd Zawawi Z; Ramly N; Robert F; Suppiah J; Thayan R
EPI_ISL_2603038, EPI_ISL_2603039, EPI_ISL_2603040, EPI_ISL_2603051	KEMRI-Wellcome Trust Research Programme,Kilifi	KEMRI-Wellcome Trust Research Programme,Kilifi	; Githinji G.; Matoke D.; Mburu M.W.; Mohamed K.S.; Onyango C.; de Laurent Z.
EPI_ISL_2425254, EPI_ISL_2425255, EPI_ISL_2425256, EPI_ISL_2425257, EPI_ISL_2425258, EPI_ISL_2425316, EPI_ISL_2425317, EPI_ISL_2425318, EPI_ISL_2425319, EPI_ISL_2425320, EPI_ISL_2425321, EPI_ISL_2425322, EPI_ISL_2726311, EPI_ISL_2726502, EPI_ISL_2726503, EPI_ISL_2726504, EPI_ISL_2726516, EPI_ISL_2726520, EPI_ISL_2726522, EPI_ISL_2832259, EPI_ISL_2832634, EPI_ISL_2833228, EPI_ISL_2833229, EPI_ISL_2833350, EPI_ISL_2833355, EPI_ISL_2833431, EPI_ISL_2834778, EPI_ISL_2864474, EPI_ISL_2864485, EPI_ISL_2864489, EPI_ISL_2864691, EPI_ISL_2864762, EPI_ISL_2877534, EPI_ISL_2878165, EPI_ISL_2878182, EPI_ISL_2878185, EPI_ISL_2886256, EPI_ISL_2886360, EPI_ISL_2886382			
see above	KU Leuven, Rega Institute, Clinical and Epidemiological Virology	KU Leuven, Rega Institute, Clinical and Epidemiological Virology	Bert Vanmechelen; Joan Marti-Carerras; Piet Maes; Tony Wawina-Bokalanga
EPI_ISL_2608421	Kampong Cham Rapid Response Team	Virology Unit, Institut Pasteur du Cambodge	Cecile Troupin; Chau Darapheak; Chin Savuth; Erik A Karlsson; Jurre Y Siegers; Kraing Sidonn; Leakhena Pum; Ly Sovann; Veasna Duong; Yi Sengdoeurn
EPI_ISL_2608444	Kampot Rapid Response Team	Virology Unit, Institut Pasteur du Cambodge	Cecile Troupin; Chau Darapheak; Chin Savuth; Erik A Karlsson; Jurre Y Siegers; Kraing Sidonn; Leakhena Pum; Ly Sovann; Veasna Duong; Yi Sengdoeurn
EPI_ISL_2544687, EPI_ISL_2544688	Kanagawa Prefectural Institute of Public Health	Kanagawa Prefectural Institute of Public Health	Itsumi Toyokura; Junichi Sakuragi; Kentaro Itokawa; Makiko Kondo; Makoto Kuroda; Masanori Hashino; Rieko Suzuki; Rina Tanaka; Sumi Watanabe; Takako Sano; Takayuki Hishiki; Tomohiko Takasaki; Tsuyoshi Sekizuka
EPI_ISL_2893690, EPI_ISL_2894546	Kansas Health and Environmental Lab	Kansas Health and Environmental Lab	Ben Olsen; Jonathan Barnell; Katherine Wiggins; Mike Grose; and Phil Adam
EPI_ISL_1791065, EPI_ISL_1791066	Kantonsspital Baden AG	Institute of Medical Virology, University of Zurich	Alexandra Trkola; Annette Audigé; Cyril Shah; Gabriela Ziltener; Guido Bloemberg; Jon Huder; Jürg Böni; Kevin Steiner; Maria Grünberg; Maryam Zaheri; Michael Huber; Riccarda Capaul; Stefan Schmutz; Verena Kufner
EPI_ISL_2854783, EPI_ISL_2854784, EPI_ISL_2854796	Kantor Kesehatan Pelabuhan Kelas I Medan	National Institute of Health Research and Development	Arie Ardiansyah Nugraha; Hana Apsari Pawestri; Hartanti Dian Ikawati; Kartika Dewi Puspa; Krisna Pangesti; Nelly Puspandari; Subangkit; Triyani Soekarso; Vivi Setiawaty
EPI_ISL_2233091, EPI_ISL_2233092, EPI_ISL_2233093, EPI_ISL_2233096, EPI_ISL_2233097, EPI_ISL_2233098, EPI_ISL_2233099, EPI_ISL_2233100, EPI_ISL_2233101, EPI_ISL_2233102, EPI_ISL_2233103, EPI_ISL_2233104, EPI_ISL_2233105			
see above	Kantor Kesehatan Pelabuhan Kelas II Cilacap	National Institute of Health Research and Development	Arie Ardiansyah Nugraha; Hana Apsari Pawestri; Hartanti Dian Ikawati; Kartika Dewi Puspa; Krisna Pangesti; Nelly Puspandari; Subangkit; Triyani Soekarso; Vivi Setiawaty
EPI_ISL_2854757, EPI_ISL_2854758, EPI_ISL_2854759	Kantor Kesehatan Pelabuhan Kelas III Bengkulu	National Institute of Health Research and Development	Arie Ardiansyah Nugraha; Hana Apsari Pawestri; Hartanti Dian Ikawati; Kartika Dewi Puspa; Krisna Pangesti; Nelly Puspandari; Subangkit; Triyani Soekarso; Vivi Setiawaty
EPI_ISL_2832805, EPI_ISL_2840080	Karolinska University Hospital Solna	Karolinska University Hospital	Annelie Bjerkner; Isak Sylvin; Jan Albert; Karolina Ininbergs; Lina Guerra Blomqvist; Lynda Eneh; Martin Ekman; Martina Wahlund; Robert Dyrdak; Sandra Broddesson; Tanja Normark; Tobias Allander; Valtteri Wirta; Zhibing Yun
EPI_ISL_2648221, EPI_ISL_2648223, EPI_ISL_2648224, EPI_ISL_2648226, EPI_ISL_2648235, EPI_ISL_2648236, EPI_ISL_2648237, EPI_ISL_2820296, EPI_ISL_2820300, EPI_ISL_2820303, EPI_ISL_2820304, EPI_ISL_2820307, EPI_ISL_2820308, EPI_ISL_2820312, EPI_ISL_2820321, EPI_ISL_2820322, EPI_ISL_2820325, EPI_ISL_2820326, EPI_ISL_2820330, EPI_ISL_2820331, EPI_ISL_2820333, EPI_ISL_2820386, EPI_ISL_2820392, EPI_ISL_2820400			
see above	Kasane Primary Hospital Laboratory	Botswana Harvard HIV Reference Laboratory	Agnes Karutwaeng; Boitumelo Zuze; Botshelo Radibe; Dorcas Maruapula; Joseph Makhema; Keoratile Ntshambiwa; Legodile Kooepile; Madisa Mine; Mosepele Mosepele; Ontlametse T. Bareng; Roger Shapiro; Rose Munyere; Shahin Lockman; Sikhulile Moyo; Simani Gaseitsiwe; Thongbotho Mphoyakgosi; Wonderful T. Choga
EPI_ISL_2433313, EPI_ISL_2433572, EPI_ISL_2433573, EPI_ISL_2433574, EPI_ISL_2433575, EPI_ISL_2433576, EPI_ISL_2433577, EPI_ISL_2433578, EPI_ISL_2433579, EPI_ISL_2433580, EPI_ISL_2433581, EPI_ISL_2433582, EPI_ISL_2433583, EPI_ISL_2433584, EPI_ISL_2433585, EPI_ISL_2433586, EPI_ISL_2433587, EPI_ISL_2433588, EPI_ISL_2433589, EPI_ISL_2433590, EPI_ISL_2433591, EPI_ISL_2433592, EPI_ISL_2433593, EPI_ISL_2433594, EPI_ISL_2433595, EPI_ISL_2433596, EPI_ISL_2433597, EPI_ISL_2433598, EPI_ISL_2433599, EPI_ISL_2433600, EPI_ISL_2433601, EPI_ISL_2433602, EPI_ISL_2433603, EPI_ISL_2433604, EPI_ISL_2433605, EPI_ISL_2433606, EPI_ISL_2433607, EPI_ISL_2433608, EPI_ISL_2433609, EPI_ISL_2433610, EPI_ISL_2433611, EPI_ISL_2433612, EPI_ISL_2433613, EPI_ISL_2433614, EPI_ISL_2433615, EPI_ISL_2433616, EPI_ISL_2433617			
see above	Kasemrad international hospital	Division of Genomic Medicine and Innovation support,Department of Medical Sciences, Ministry of Public Health, Thailand	Archawin Rojanawiwat; Jirapha Pakdee; Natthakul Bunneang; Nuanjun Wichukchinda; Penpitcha Thawong; Pilailuk Akkapaiboon Okada; Pundharika Piboonsiri; Surakameth Mahasirimongkol; Waritta Sawaengdee
EPI_ISL_2840605	Kasemrad international hospital ratthanatibeth	National Institute of Health, Department of Medical Sciences, Ministry of Public Health, Thailand	; Natchaya Khiadsang; Nuttida Thongpramul; Pakorn Piromtong; Pilailuk Okada; Ratana Tacharoenmuang; Siripaporn Phuygun; Sittiporn Parnmen; Sunthareeya Waicharoen; Thanutsapa Thanadachakul; Warawan Wongboot; sirikanda wimol
EPI_ISL_2693549	Klinika za infektivne bolesti "Dr. Fran Mihaljević"	Hrvatski zavod za javno zdravstvo	Irena Tabain; Ivana Ferenčak
EPI_ISL_2758190	Klinikum Wels-Grieskirchen	Bergthaler laboratory, CeMM Research Center for Molecular Medicine of the Austrian Academy of Sciences	Andreas Bergthaler; Anna Schedl; Bekir Erguner; Benedikt Agerer; Christoph Bock; Fabian Amman; Jan Laine; Lukas Endler; Maelle Le Moing; Martin Senekowitsch; Matthew Thornton; Michael Schuster; Petr Triska; Thomas Penz
EPI_ISL_2397179, EPI_ISL_2397200	Klinisch Laboratorium ZNA	Klinisch Laboratorium ZNA	Verstrepen et al.
EPI_ISL_2815331	Kuala Lumpur General Hospital	Institute for Medical Research, Infectious Disease Research Centre, National Institutes of Health, Ministry of Health Malaysia	Azizan MA; Kamel K; Mohd Zawawi Z; Ramly N; Robert F; Suppiah J; Thayan R
EPI_ISL_2854187	Kuala Terengganu District Health Office	Institute for Medical Research, Infectious Disease Research Centre, National Institutes of Health, Ministry of Health Malaysia	Anasir MI; Azizan MA; Kamel K; Mohd Zawawi Z; Ramly N; Robert F; Suppiah J; Thayan R
EPI_ISL_1941773	LA SOURCE	Laboratory of genomics and metagenomics	Claire Bertelli; Damien Jacot; Gilbert Greub; Sebastien Aeby; Trestan Pillonel
EPI_ISL_2854729	LAB RS Mitra Kemayoran	National Institute of Health Research and Development	; Arie Ardiansyah Nugraha; Hana Apsari Pawestri; Hartanti Dian Ikawati; Kartika Dewi Puspa; Krisna Pangesti; Nelly Puspandari; Subangkit; Triyani Soekarso; Vivi Setiawaty
EPI_ISL_2629561	LABM DU PRE - LABOMAINE	CNR Virus des Infections Respiratoires - France SUD	Antonin Bal; Bruno Lina; Gregory Destras; Gwendolyne Burfin; Hadrien Regue; Laurence Josset; Martine Valette; Quentin Semanas
EPI_ISL_2228675	LABM Unilabs EYLAU Corbeil	Department of Virology, Henri Mondor University Hospital, Assistance Publique Hôpitaux de Paris, Université Paris-Est Créteil, INSERM U955	Alexandre Soulier; Christophe Rodriguez; Elisabeth Trawinski; Guillaume Gricourt; Jean-Michel Pawlotsky; Melissa N'Debi; Slim Fourati; Vanessa Demontant
EPI_ISL_2629445	LABORATOIRE BIOESTEREL	CNR Virus des Infections Respiratoires - France SUD	Antonin Bal; Bruno Lina; Gregory Destras; Gwendolyne Burfin; Hadrien Regue; Laurence Josset; Martine Valette; Quentin Semanas
EPI_ISL_2567490, EPI_ISL_2567491, EPI_ISL_2567492, EPI_ISL_2567494, EPI_ISL_2567495, EPI_ISL_2676548	LABORATOIRE FORTE BIO ST PAUL LES DAX	CNR Virus des Infections Respiratoires - France SUD	Antonin Bal; Bruno Lina; Gregory Destras; Gwendolyne Burfin; Hadrien Regue; Laurence Josset; Martine Valette; Quentin Semanas
EPI_ISL_2893787	LABORATOIRE UNILIANS DECINES	CNR Virus des Infections Respiratoires - France SUD	Antonin Bal; Bruno Lina; Gregory Destras; Gwendolyne Burfin; Hadrien Regue; Laurence Josset; Martine Valette; Quentin Semanas
EPI_ISL_2676692, EPI_ISL_2893832	LABORATOIRE d'ANALYSES de BIOLOGIE MEDICALES	CNR Virus des Infections Respiratoires - France SUD	Antonin Bal; Bruno Lina; Gregory Destras; Gwendolyne Burfin; Hadrien Regue; Laurence Josset; Martine Valette; Quentin Semanas
EPI_ISL_2893866	LABORATOIRE- BIOESTERL	CNR Virus des Infections Respiratoires - France SUD	Antonin Bal; Bruno Lina; Gregory Destras; Gwendolyne Burfin; Hadrien Regue; Laurence Josset; Martine Valette; Quentin Semanas
EPI_ISL_2801427	LABORATORIUM BADAŃ KLINICZNYCH WSSE w OPOLU	National Institute of Public Health - National Institute of Hygiene	Gierczyńki Rafał; Sadkowska-Todys Małgorzata; Wołkowicz Tomasz
EPI_ISL_2536122, EPI_ISL_2536124, EPI_ISL_2536127, EPI_ISL_2536129	LAC UCoimbra	Instituto Nacional de Saude (INSA)	Borges et al
EPI_ISL_1904967, EPI_ISL_1904969, EPI_ISL_1904971, EPI_ISL_1904972, EPI_ISL_1904973	LAM ORIADE ABBAYE ST MARTIN D'HERES	CNR Virus des Infections Respiratoires - France SUD	Antonin Bal; Bruno Lina; Gregory Destras; Gwendolyne Burfin; Hadrien Regue; Laurence Josset; Martine Valette; Quentin Semanas
EPI_ISL_2841774	LBM ALPHABIO, Marseille	LBM ALPHABIO, Marseille	Vincent GARCIA
EPI_ISL_2289248, EPI_ISL_2289249, EPI_ISL_2482846, EPI_ISL_2676752	LBM UNIBIO VALENTIN	CNR Virus des Infections Respiratoires - France SUD	Antonin Bal; Bruno Lina; Gregory Destras; Gwendolyne Burfin; Hadrien Regue; Laurence Josset; Martine Valette; Quentin Semanas
EPI_ISL_2533818	LESP Baja California Sur	Instituto de Diagnostico y Referencia Epidemiologicos (INDRE)	Abril Rodriguez-Maldonado; Ariadna Medina-Benitez; Claudia Wong-Arambula; Ernesto Ramirez-Gonzalez.; Gisela Barrera-Badillo; Irma Lopez-Martinez; Joaquin Quiroz-Mercado; Lucia Hernandez-Rivas; Natividad Cruz-Ortiz; Sergio Rangel-Guerrero;

			Tatiana Nunez-Garcia; Vanessa Rivero-Arredondo
EPI_ISL_2835024	LESP Campeche	Instituto de Diagnostico y Referencia Epidemiologicos (INDRE)	Abril Rodriguez-Maldonado; Ariadna Medina-Benitez; Claudia Wong-Arambula; Ernesto Ramirez-Gonzalez.; Gisela Barrera-Badillo; Irma Lopez-Martinez; Joaquin Quiroz-Mercado; Lucia Hernandez-Rivas; Natividad Cruz-Ortiz; Sergio Rangel-Guerrero; Tatiana Nunez-Garcia; Vanessa Rivero-Arredondo
EPI_ISL_2246838, EPI_ISL_2443048, EPI_ISL_2533817, EPI_ISL_2674901, EPI_ISL_2674902, EPI_ISL_2674904, EPI_ISL_2835009, EPI_ISL_2835012, EPI_ISL_2835027, EPI_ISL_2835032, EPI_ISL_2835043			
see above	LESP Ciudad de Mexico	Instituto de Diagnostico y Referencia Epidemiologicos (INDRE)	Abril Rodriguez-Maldonado; Ariadna Medina-Benitez; Claudia Wong-Arambula; Ernesto Ramirez-Gonzalez.; Gisela Barrera-Badillo; Irma Lopez-Martinez; Joaquin Quiroz-Mercado; Lucia Hernandez-Rivas; Maribel Gonzalez-Villa; Natividad Cruz-Ortiz; Sergio Rangel-Guerrero; Tatiana Nunez-Garcia; Vanessa Rivero-Arredondo
EPI_ISL_2246827	LESP Ciudad de Mexico /LANS	Instituto de Diagnostico y Referencia Epidemiologicos (INDRE)	Abril Rodriguez-Maldonado; Ariadna Medina-Benitez; Claudia Wong-Arambula; Ernesto Ramirez-Gonzalez.; Gisela Barrera-Badillo; Irma Lopez-Martinez; Joaquin Quiroz-Mercado; Lucia Hernandez-Rivas; Natividad Cruz-Ortiz; Sergio Rangel-Guerrero; Tatiana Nunez-Garcia; Vanessa Rivero-Arredondo
EPI_ISL_2533821	LESP Ciudad de México /DIAGNOMOL	Instituto de Diagnostico y Referencia Epidemiologicos (INDRE)	Abril Rodriguez-Maldonado; Ariadna Medina-Benitez; Claudia Wong-Arambula; Ernesto Ramirez-Gonzalez.; Gisela Barrera-Badillo; Irma Lopez-Martinez; Joaquin Quiroz-Mercado; Lucia Hernandez-Rivas; Natividad Cruz-Ortiz; Sergio Rangel-Guerrero; Tatiana Nunez-Garcia; Vanessa Rivero-Arredondo
EPI_ISL_2835040	LESP Estado de Mexico	Instituto de Diagnostico y Referencia Epidemiologicos (INDRE)	Abril Rodriguez-Maldonado; Ariadna Medina-Benitez; Claudia Wong-Arambula; Ernesto Ramirez-Gonzalez.; Gisela Barrera-Badillo; Irma Lopez-Martinez; Joaquin Quiroz-Mercado; Lucia Hernandez-Rivas; Maribel Gonzalez-Villa; Natividad Cruz-Ortiz; Sergio Rangel-Guerrero; Tatiana Nunez-Garcia; Vanessa Rivero-Arredondo
EPI_ISL_2094505	LESP Jalisco	Instituto de Diagnostico y Referencia Epidemiologicos (INDRE)	Abril Rodriguez-Maldonado; Ariadna Medina-Benitez; Claudia Wong-Arambula; Ernesto Ramirez-Gonzalez.; Gisela Barrera-Badillo; Irma Lopez-Martinez; Joaquin Quiroz-Mercado; Lucia Hernandez-Rivas; Natividad Cruz-Ortiz; Sergio Rangel-Guerrero; Tatiana Nunez-Garcia; Vanessa Rivero-Arredondo
EPI_ISL_2246829, EPI_ISL_2246830, EPI_ISL_2246831, EPI_ISL_2246832, EPI_ISL_2246833, EPI_ISL_2443050	LESP Michoacan	Instituto de Diagnostico y Referencia Epidemiologicos (INDRE)	Abril Rodriguez-Maldonado; Ariadna Medina-Benitez; Claudia Wong-Arambula; Ernesto Ramirez-Gonzalez.; Gisela Barrera-Badillo; Irma Lopez-Martinez; Joaquin Quiroz-Mercado; Lucia Hernandez-Rivas; Natividad Cruz-Ortiz; Sergio Rangel-Guerrero; Tatiana Nunez-Garcia; Vanessa Rivero-Arredondo
EPI_ISL_2342999	LESP Michoacan/LANS	Instituto de Diagnostico y Referencia Epidemiologicos (INDRE)	Abril Rodriguez-Maldonado; Ariadna Medina-Benitez; Claudia Wong-Arambula; Ernesto Ramirez-Gonzalez.; Gisela Barrera-Badillo; Irma Lopez-Martinez; Joaquin Quiroz-Mercado; Lucia Hernandez-Rivas; Natividad Cruz-Ortiz; Sergio Rangel-Guerrero; Tatiana Nunez-Garcia; Vanessa Rivero-Arredondo
EPI_ISL_2835008	LESP Morelos	Instituto de Diagnostico y Referencia Epidemiologicos (INDRE)	Abril Rodriguez-Maldonado; Ariadna Medina-Benitez; Claudia Wong-Arambula; Ernesto Ramirez-Gonzalez.; Gisela Barrera-Badillo; Irma Lopez-Martinez; Joaquin Quiroz-Mercado; Lucia Hernandez-Rivas; Natividad Cruz-Ortiz; Sergio Rangel-Guerrero; Tatiana Nunez-Garcia; Vanessa Rivero-Arredondo
EPI_ISL_2736785	LESP Puebla	Instituto de Diagnostico y Referencia Epidemiologicos (INDRE)	Abril Rodriguez-Maldonado; Ariadna Medina-Benitez; Claudia Wong-Arambula; Ernesto Ramirez-Gonzalez.; Gisela Barrera-Badillo; Irma Lopez-Martinez; Joaquin Quiroz-Mercado; Lucia Hernandez-Rivas; Natividad Cruz-Ortiz; Sergio Rangel-Guerrero; Tatiana Nunez-Garcia; Vanessa Rivero-Arredondo
EPI_ISL_2835014	LESP Queretaro	Instituto de Diagnostico y Referencia Epidemiologicos (INDRE)	Abril Rodriguez-Maldonado; Ariadna Medina-Benitez; Claudia Wong-Arambula; Ernesto Ramirez-Gonzalez.; Gisela Barrera-Badillo; Irma Lopez-Martinez; Joaquin Quiroz-Mercado; Lucia Hernandez-Rivas; Natividad Cruz-Ortiz; Sergio Rangel-Guerrero; Tatiana Nunez-Garcia; Vanessa Rivero-Arredondo
EPI_ISL_2443049, EPI_ISL_2533820	LESP Quintana Roo	Instituto de Diagnostico y Referencia Epidemiologicos (INDRE)	Abril Rodriguez-Maldonado; Ariadna Medina-Benitez; Claudia Wong-Arambula; Ernesto Ramirez-Gonzalez.; Gisela Barrera-Badillo; Irma Lopez-Martinez; Joaquin Quiroz-Mercado; Lucia Hernandez-Rivas; Natividad Cruz-Ortiz; Sergio Rangel-Guerrero; Tatiana Nunez-Garcia; Vanessa Rivero-Arredondo
EPI_ISL_2246834, EPI_ISL_2246835, EPI_ISL_2246836, EPI_ISL_2246837, EPI_ISL_2736781, EPI_ISL_2736783, EPI_ISL_2835020			
see above	LESP Sinaloa	Instituto de Diagnostico y Referencia Epidemiologicos (INDRE)	Abril Rodriguez-Maldonado; Ariadna Medina-Benitez; Claudia Wong-Arambula; Ernesto Ramirez-Gonzalez.; Gisela Barrera-Badillo; Irma Lopez-Martinez; Joaquin Quiroz-Mercado; Lucia Hernandez-Rivas; Natividad Cruz-Ortiz; Sergio Rangel-Guerrero; Tatiana Nunez-Garcia; Vanessa Rivero-Arredondo
EPI_ISL_2533819	LESP Yucatan	Instituto de Diagnostico y Referencia Epidemiologicos (INDRE)	Abril Rodriguez-Maldonado; Ariadna Medina-Benitez; Claudia Wong-Arambula; Ernesto Ramirez-Gonzalez.; Gisela Barrera-Badillo; Irma Lopez-Martinez; Joaquin Quiroz-Mercado; Lucia Hernandez-Rivas; Natividad Cruz-Ortiz; Sergio Rangel-Guerrero; Tatiana Nunez-Garcia; Vanessa Rivero-Arredondo
EPI_ISL_2545652, EPI_ISL_2861683	LHUB-ULB	Labo Klinische Biologie, UZA	Basil Britto Xavier; Christine Lammens; Herman Goossens; Ines Verbesselt; Jasmine Coppens; Kathleen Holemans; Marie Le Mercier; Veerle Matheeussen
EPI_ISL_2695350, EPI_ISL_2695354, EPI_ISL_2695361	Lab Dra Celeste Formosinho	Instituto Nacional de Saude (INSA)	Borges et al
EPI_ISL_2617534	Lab Genomik Solidaritas Indonesia	National Institute of Health Research and Development	Arie Ardiansyah Nugraha; Hana Apsari Pawestri; Hartanti Dian Ikawati; Kartika Dewi Puspa; Krisna Pangesti; Meutia Kumaheri; Nelly Puspandari; Subangkit; Triyani Soekarso; Vivi Setiawaty
EPI_ISL_2695396	Lab La Salete Robles - VN Famalicao	Instituto Nacional de Saude (INSA)	Borges et al
EPI_ISL_1969245	Lab RSUP DR Mohammad Hoesin Palembang	National Institute of Health Research and Development	Arie Ardiansyah Nugraha; Hana Apsari Pawestri; Hartanti Dian Ikawati; Kartika Dewi Puspa; Krisna Pangesti; Nelly Puspandari; Subangkit; Triyani Soekarso; Vivi Setiawaty
EPI_ISL_1827216, EPI_ISL_2192420, EPI_ISL_2604978, EPI_ISL_2605118, EPI_ISL_2605502, EPI_ISL_2605553, EPI_ISL_2861441, EPI_ISL_2861490, EPI_ISL_2861502, EPI_ISL_2861533, EPI_ISL_2861553, EPI_ISL_2861560			
see above	Lab voor klinische biologie	Lab voor klinische biologie	Bruno Verhasselt; Hannelore Hamerlinck; Marija Janevska
EPI_ISL_2725164	Lab. Microbiologia e Virologia Cotugno A.O. dei Colli - Istituto Zooprofilattico Sperimentale del Mezzogiorno	Telethon Institute of Genetics and Medicine (TIGEM)	Antonio Grimaldi Patrizia Annunziata Francesco Panariello Biancamaria Pierri Claudia Tiberio Teresa Giuliano Valentina Bouche Chiara Colantuono Maria Concetta Cuomo Denise Di Concilio Lucio Di Filippo Anna Manfredi Marcello Salvi Antonio Limone Luigi Atripaldi Pellegrino Cerino Andrea Ballabio Davide Cacchiarelli
EPI_ISL_2121121	LabKom - Labor Augsburg MVZ GmbH	Robert Koch Institute	
EPI_ISL_2260688	LabKom - Labor Mainz MVZ GmbH	Robert Koch Institute	
EPI_ISL_2536107, EPI_ISL_2695378, EPI_ISL_2695383, EPI_ISL_2895261	Labeto - CAB - Leiria	Instituto Nacional de Saude (INSA)	Borges et al
EPI_ISL_2854726	Labkesda Kota Tangerang	National Institute of Health Research and Development	; Arie Ardiansyah Nugraha; Hana Apsari Pawestri; Hartanti Dian Ikawati; Kartika Dewi Puspa; Krisna Pangesti; Nelly Puspandari; Subangkit; Triyani Soekarso; Vivi Setiawaty
EPI_ISL_2233094	Labkesda Prov Kal Tim	National Institute of Health Research and Development	Arie Ardiansyah Nugraha; Hana Apsari Pawestri; Hartanti Dian Ikawati; Kartika Dewi Puspa; Krisna Pangesti; Nelly Puspandari; Subangkit; Triyani Soekarso; Vivi Setiawaty
EPI_ISL_1915156, EPI_ISL_2029687, EPI_ISL_2029713, EPI_ISL_2029727, EPI_ISL_2029740, EPI_ISL_2029751, EPI_ISL_2029877, EPI_ISL_2029916, EPI_ISL_2462964, EPI_ISL_2464181, EPI_ISL_2531945, EPI_ISL_2627997, EPI_ISL_2709277, EPI_ISL_2790915, EPI_ISL_2790980, EPI_ISL_2790990, EPI_ISL_2835716, EPI_ISL_2835722, EPI_ISL_2835839			
see above	Labo Analyses Med	National Reference Center for Viruses of Respiratory Infections, Institut Pasteur, Paris	Angela Brisebarre; Arditi Cocco; BéNéDicte Roquebert; Camille Capel; Caroline Devaux; Christophe Malabat; Corinne Maufrais; Damien Mornico; Elodie Etienne; Emmanuelle Permal; Eric Grandsire; Etienne Simon-Lorière; Fabienne Artur; Frédéric Lemoine; Hugues Leroy; Jean Boyer; Judith Zerah; Louise Lefrançois; Marion Barbet; Maud Vanpeene; Méline Bizard; Nicolas Capron; Nouredine Sadeg; Pierre Lechat; Pierre Netzer; Sylvie Behillil; Sylvie Van der Werf; Victoire Baillet; Vincent Enouf
EPI_ISL_2709283	Labo Analyses med	National Reference Center for Viruses of Respiratory Infections, Institut Pasteur, Paris	Angela Brisebarre; Camille Capel; Christophe Malabat; Corinne Maufrais; Etienne Simon-Lorière; Frédéric Lemoine; Louise Lefrançois; Marion Barbet; Maud Vanpeene; Méline Bizard; OphéLie Said-Delattre; Sylvie Behillil; Sylvie Van der Werf; Vincent Enouf
EPI_ISL_2348577, EPI_ISL_2348578, EPI_ISL_2348579, EPI_ISL_2348581, EPI_ISL_2348587, EPI_ISL_2348591	Labo Luc Olivier	GIGA Medical Genomics	Bouchra Boujemla; Cécile Meex; Keith Durkin; Maria Artesi; Marie-Pierre Hayette; Nathalie Renotte; Pierrette Melin; Raphaël Boreux; Sébastien Bontems; Vincent Bours
EPI_ISL_2448636	Laboffice	CHU Poitiers	Agnes BEBY-DEFAUX; Birama N'DIAYE; Caroline MICHAUD; Magali GARCIA; Manon PRAT; Maxime PICHON; Nicolas LEVEQUE; Valentin BON-BARET
EPI_ISL_1843504, EPI_ISL_2109111, EPI_ISL_2125917, EPI_ISL_2470015	Labor 28 MVZ GmbH	Robert Koch Institute	
EPI_ISL_2389915, EPI_ISL_2678387, EPI_ISL_2678417, EPI_ISL_2678421, EPI_ISL_2678435, EPI_ISL_2845807	Labor Becker & Kollegen (Standort München)	Robert Koch Institute	
EPI_ISL_2339438	Labor Berlin Charité Vivantes GmbH / Institut für Virologie	Charité Universitätsmedizin Berlin, Institut für Virologie/Labor Berlin	Barbara Mühlemann; Christian Drosten; Christine Stephan; Peter Menzel; Rolf Schwarzer; Terry Jones; Victor M Corman
EPI_ISL_1904299	Labor Doz DDr Stefan Mustafa	AGES IMED Vienna	Alexander Indra; Elisabeth Walter; Johanna Schmitt; Sara Meschini; Stefan Mustafa; Theodhora Ziu
EPI_ISL_1566762, EPI_ISL_2845224, EPI_ISL_2845226, EPI_ISL_2845231	Labor Dr. Heidrich & Kollegen MVZ GmbH Hamburg	Robert Koch Institute	
EPI_ISL_2125017	Labor Dr. Wisplinghoff - Koln	Robert Koch Institute	
EPI_ISL_2845269	Labor Dr. Wisplinghoff - Köln	Robert Koch Institute	
EPI_ISL_1848757	Labor MÃ¶nchengladbach MVZ Dr. Stein + Kollegen GbR	Robert Koch Institute	
EPI_ISL_2111142, EPI_ISL_2762279	Laborarztpraxis Dres. med. Walther Weindel & Kollegen	Robert Koch Institute	
EPI_ISL_2464430	Laboratoire Ana-L	Department of Virology, Henri Mondor University Hospital, Assistance Publique Hôpitaux de Paris, Université Paris-Est Créteil, INSERM U955	Alexandre Soulier; Christophe Rodriguez; Elisabeth Trawinski; Guillaume Gricourt; Jean-Michel Pawlotsky; Melissa N'Debi; Slim Fourati; Vanessa Demontant

EPI_ISL_2893527, EPI_ISL_2893532	Laboratoire Hôpital Sainte Musse	Laboratoire Hôpital Sainte Musse	Lionel Chollet
EPI_ISL_2401121, EPI_ISL_2401139, EPI_ISL_2401152	Laboratoire national de sante, Microbiology, Virology	Laboratoire national de sante, Microbiology, Microbial Genomics Platform	Anke Wienecke-Baldacchino; Catherine Ragimbeau; Fatu Djabi; Jessica Tapp; Lise Pignon; Raoul Salmon; Tamir Abdelrahman; Trung Nguyen Nguyen
EPI_ISL_1917963, EPI_ISL_2400942, EPI_ISL_2400960	Laboratoires Reunis	Laboratoire national de sante, Microbiology, Microbial Genomics Platform	Anke Wienecke-Baldacchino; Bernard Weber; Catherine Ragimbeau; Fatu Djabi; Jessica Tapp; Lise Pignon; Raoul Salmon; Tamir Abdelrahman
EPI_ISL_2400820, EPI_ISL_2400821, EPI_ISL_2400824, EPI_ISL_2400827, EPI_ISL_2400828, EPI_ISL_2400829, EPI_ISL_2400830			
see above	Laboratoires d'analyses medicales - Ketterthill	Laboratoire national de sante, Microbiology, Microbial Genomics Platform	Anke Wienecke-Baldacchino; Caroline Scheiber; Catherine Ragimbeau; Fatu Djabi; Jessica Tapp; Lise Pignon; Raoul Salmon; Serge Vedy; Tamir Abdelrahman
EPI_ISL_2036263, EPI_ISL_2036266, EPI_ISL_2036268, EPI_ISL_2308278, EPI_ISL_2421279, EPI_ISL_2421280, EPI_ISL_2421281, EPI_ISL_2421282, EPI_ISL_2537480			
see above	Laboratori de Referencia de Catalunya	Laboratori de Referencia de Catalunya	Bellosillo B.; Canal M.; Hernandez JJ.; Padilla E.; Ramirez A.; Vilas A.
EPI_ISL_2686054, EPI_ISL_2686055, EPI_ISL_2686072	Laboratorio Analisi Osp. Città di Castello - Azienda USL Umbria1	Istituto Zooprofilattico Sperimentale dell'Abruzzo e Molise "G. Caporale"	Ancora M; Calistri P; Cammà C; Curini V; Delli Compagni E; Di Domenico M; Di Pasquale A; Lorusso A; Malagigi V; Mangone I; Marcacci M; Puglia I; Rinaldi A; Savini G; Scialabba S; Taccani P
EPI_ISL_2340135	Laboratorio Aziendale di Microbiologia e Virologia, Azienda Sanitaria dell'Alto Adige	Laboratorio Aziendale di Microbiologia e Virologia, Azienda Sanitaria dell'Alto Adige	Anne Picard; Chiara Cantaloni; Claudia Volpato; Elisa Masi; Elisabetta Giacobazzi; Elisabetta Pagani; Irene Bianconi; Stefanie Wieser
EPI_ISL_2671488, EPI_ISL_2671539, EPI_ISL_2671563, EPI_ISL_2671649	Laboratorio Central de Epidemiologia (LCE)	Unidad de Genomica Avanzada	; Alejandra Garcia-Gasca; Alejandra Hernandez-Teran; Alejandro Sanchez-Flores; Alfredo Herrera-Estrella; Alicia Ocaña-Mondragon; Andreu Comas-Garcia; Angel Gustavo Salas-Lais; Antonio Loza Roman; Bernardo Martinez-Miguel; Blanca Taboada; Brenda Irasema Maldonado-Meza; Bruno Gomez-Gil; Carla Ivon Herrera-Najera; Carlos F. Arias; Celia Boukadida; Celida Duque Molina; Celida Martinez- Rodriguez; Clara Esperanza Santacruz-Tinoco; Concepcion Grajales-Muñiz; Consorcio Mexicano de Vigilancia Genomica (CoViGen-Mex). Authors (in alphabetical order): Julio Elias Alvarado-Yaah; Cristobal Chaidez-Quiroz; Daniel Fregoso-Rueda; Daniel Lira Morales; Eduardo Becerril-Vargas; Fernando Fontove-Herrera; Fidencio Mejia-Nepomuceno; Francisco Pulido; Gloria Elena Espinosa-Ayala; Gloria Maria Molina-Salinas; Gloria Vazquez; Hector Esteban Paz-Juarez; Hector Montoya-Fuentes; Helen Haydee Fernanda Ramirez-Plascencia; Irvin Gonzalez-Lopez; Jean Pierre Gonzalez; Jesus Hernandez; Joel Armando Vazquez-Perez.; Jorge Salas-Hernandez; Jose Antonio Enciso-Moreno; Jose Arturo Martinez-Orozco; Jose Esteban Muñoz-Medina; Jose de Jesus Nuñez-Contreras; Juan Bautista Chale-Dzul; Julissa Enciso-Ibarra; Luis Alberto Ochoa-Carrera; Margarita Matias-Florentino; Maria Guadalupe Santiago-Mauricio; Maria Guadalupe de Jesus Mireles-Rivera; Mario Mujica-Sanchez; Marissa Perez-Garcia; Nelly Selem-Mojica; Pavel Isa; Ricardo Ciria Merce; Ricardo Grande; Rosa Maria Gutierrez Rios; Santiago avila-Rios; Selene Zarate; Susana Lopez; Veronica Mata-Haro; Victor Eduardo Garcia-Arias; Victor Hugo Borja-Aburto
EPI_ISL_2681059, EPI_ISL_2681064, EPI_ISL_2681065, EPI_ISL_2681113, EPI_ISL_2681155, EPI_ISL_2681156, EPI_ISL_2681181, EPI_ISL_2681184, EPI_ISL_2681188, EPI_ISL_2681189, EPI_ISL_2681193, EPI_ISL_2681194, EPI_ISL_2681196, EPI_ISL_2681199, EPI_ISL_2681205, EPI_ISL_2681214, EPI_ISL_2681229, EPI_ISL_2681231, EPI_ISL_2681251, EPI_ISL_2801772, EPI_ISL_2801780, EPI_ISL_2801789, EPI_ISL_2801809, EPI_ISL_2801810, EPI_ISL_2801811, EPI_ISL_2801814, EPI_ISL_2801815, EPI_ISL_2801816, EPI_ISL_2801823, EPI_ISL_2801829, EPI_ISL_2801881			
see above	Laboratorio Central de Epidemiología (LCE)	Instituto de Biotecnología de la UNAM	; Alejandra García-Gasca; Alejandra Hernández-Terán; Alejandro Sánchez-Flores; Alfredo Herrera-Estrella; Alicia Ocaña-Mondragón; Andreu Comas-García; Angel Gustavo Salas-Lais; Antonio Loza Román; Bernardo Martínez-Miguel; Blanca Taboada; Brenda Irasema Maldonado-Meza; Bruno Gómez-Gil; Carla Ivón Herrera-Najera; Carlos F. Arias; Celia Boukadida; Clara Esperanza Santacruz-Tinoco; Concepción Grajales-Muñiz; Consorcio Mexicano de Vigilancia Genómica (CoViGen-Mex). Authors (in alphabetical order): Julio Elias Alvarado-Yaah; Cristóbal Cháidez-Quiróz; Célida Duque Molina; Célida Martínez- Rodríguez; Daniel Fregoso-Rueda; Daniel Lira Morales; Eduardo Becerril-Vargas; Fernando Fontove-Herrera; Fidencio Mejía-Nepomuceno; Francisco Pulido; Gloria Elena Espinosa-Ayala; Gloria María Molina-Salinas; Gloria Vazquez; Hector Esteban Paz-Juárez; Hector Montoya-Fuentes; Helen Haydee Fernanda Ramírez-Plascencia; Irvin González-López; Jean Pierre González; Jesús Hernández; Joel Armando Vázquez-Pérez.; Jorge Salas-Hernández; José Antonio Enciso-Moreno; José Arturo Martínez-Orozco; José Esteban Muñoz-Medina; José de Jesús Nuñez-Contreras; Juan Bautista Chale-Dzul; Julissa Enciso-Ibarra; Luis Alberto Ochoa-Carrera; Margarita Matías-Florentino; Mario Mújica-Sánchez; Marissa Perez-Garcia; María Guadalupe Santiago-Mauricio; María Guadalupe de Jesús Mireles-Rivera; Nelly Sélem-Mojica; Pavel Isa; Ricardo Ciria Merce; Ricardo Grande; Rosa María Gutiérrez Rios; Santiago Ávila-Ríos; Selene Zárate; Susana Lopez; Verónica Mata-Haro; Victor Eduardo García-Arias; Víctor Hugo Borja-Aburto
EPI_ISL_2645412, EPI_ISL_2645413, EPI_ISL_2645414, EPI_ISL_2645415, EPI_ISL_2645416	Laboratorio Central de Saude Publica do Estado Maranhao (LACEN-MA)	Laboratory of Respiratory Viruses and Measles, Oswaldo Cruz Institute, FIOCRUZ	Alex Pauvolid-Corrêa; Alice Sampaio Rocha; Ana Beatriz Machado Lima; Ana Carolina Mendonca; Anna Carolina Paixao; Elisa Cavalcante Pereira; Fernando Motta; Lidio Gonçalves Lima Neto; Luciana Appolinario; Marilda Siqueira on behalf of the Fiocruz COVID-19 Genomic Surveillance Network; Mia Ferreira de Araujo; Paola Resende; Renata Serrano Lopes; Taina Venas
EPI_ISL_2645417, EPI_ISL_2645418	Laboratorio Central de Saude Publica do Estado do Maranhao (LACEN-MA)	Laboratory of Respiratory Viruses and Measles, Oswaldo Cruz Institute, FIOCRUZ	Alex Pauvolid-Corrêa; Alice Sampaio Rocha; Ana Beatriz Machado Lima; Ana Carolina Mendonca; Anna Carolina Paixao; Elisa Cavalcante Pereira; Fernando Motta; Lidio Gonçalves Lima Neto; Luciana Appolinario; Marilda Siqueira on behalf of the Fiocruz COVID-19 Genomic Surveillance Network; Mia Ferreira de Araujo; Paola Resende; Renata Serrano Lopes; Taina Venas
EPI_ISL_2466268	Laboratorio Central de Saude Publica do Estado do Rio de Janeiro (LACEN-RJ)	Laboratory of Respiratory Viruses and Measles, Oswaldo Cruz Institute, FIOCRUZ	Alice Sampaio Rocha; Ana Carolina Mendonca; Andrea Cony Cavalcanti; Anna Carolina Paixao; Elisa Cavalcante Pereira; Fernando Motta; Luciana Appolinario; Marilda Siqueira on behalf of the Fiocruz COVID-19 Genomic Surveillance Network; Paola Resende; Renata Serrano Lopes; Taina Venas
EPI_ISL_2155359, EPI_ISL_2155363, EPI_ISL_2155365, EPI_ISL_2155367, EPI_ISL_2155368, EPI_ISL_2240824, EPI_ISL_2361493, EPI_ISL_2536207			
see above	Laboratorio HUB -Azienda Ospedaliero Universitaria - AOU – Cagliari	Laboratorio SPOKE Biologia Molecolare -Azienda Ospedaliero Universitaria - AOU – Cagliari	Alessandra Scano; Ferdinando Coghe; Germano Orrù; Miriam Loddo; Riccardo Cappai; Sara Fais; Valentina Medda
EPI_ISL_1970733	Laboratorio analisi - Policlinico San Pietro	Laboratory of Clinical Microbiology, Virology and Bioemergencies, ASST Fatebenefratelli Sacco - Sacco Hospital	Alberto Rizzo; Alessandro Mancon; Fiorenza Bracchitta; Luca Rizzuto; Maria Rita Gismondo; Valeria Micheli
EPI_ISL_2501860	Laboratorio analisi, IRCCS Policlinico San Donato	Laboratory of Clinical Microbiology, Virology and Bioemergencies, ASST Fatebenefratelli Sacco - Sacco Hospital	Alberto Rizzo; Alessandro Mancon; Fiorenza Bracchitta; Maria Rita Gismondo; Valeria Micheli
EPI_ISL_2562268, EPI_ISL_2652249	Laboratorio de Referencial Nacional de Virus Respiratorios	Laboratorio de Referencial Nacional de Virus Respiratorios	Carlos Padilla Rojas; Henri Bailon Calderon; Iris Silva Molina; Joseph Huayra Niquen; Lely Solari Zerpa; Luis Barcena Flores; Marco Galarza Perez; Nancy Rojas Serrano; Omar Caceres Rey; Orson Mestanza Millones; Priscila Lope Pari; Sandra Morales Ruiz; Steve Acedo Lazo; Veronica Hurtado Vela
EPI_ISL_2544220, EPI_ISL_2544231, EPI_ISL_2708969	Laboratorio di Microbiologia	Laboratorio di Microbiologia	Martinetti Lucchini Gladys; Valeria Spina
EPI_ISL_2650443	Laboratorio di Riferimento Regionale della Sicilia Occidentale per l'Emergenza COVID-19	Laboratorio di Riferimento Regionale della Sicilia Occidentale per l'Emergenza COVID-19	Carmelo Massimo Maida; Claudio Costantino; Daniela Di Naro; Fabio Tramuto; Francesco Vitale; Giorgio Graziano; Giulia Randazzo; Vincenzo Restivo; Walter Mazzucco
EPI_ISL_2680919, EPI_ISL_2680921, EPI_ISL_2680926, EPI_ISL_2894549, EPI_ISL_2894550	Laboratorios Delia Barraza	Laboratorio nacional-LANIIA-CIAD	; Alejandra García-Gasca; Alejandra Hernández-Terán; Alejandro Sánchez-Flores; Alfredo Herrera-Estrella; Alicia Ocaña-Mondragón; Andreu Comas-García; Angel Gustavo Salas-Lais; Antonio Loza Román; Bernardo Martínez-Miguel; Blanca Taboada; Brenda Irasema Maldonado-Meza; Bruno Gómez-Gil; Carla Ivón Herrera-Najera; Carlos F. Arias; Celia Boukadida; Clara Esperanza Santacruz-Tinoco; Claudia Soto Félix; Concepción Grajales-Muñiz; Consorcio Mexicano de Vigilancia Genómica (CoViGen-Mex). Authors (in alphabetical order): Julio Elias Alvarado-Yaah; Cristóbal Cháidez-Quiróz; Célida Duque Molina; Célida Martínez- Rodríguez; Daniel Fregoso-Rueda; Daniel Lira Morales; Eduardo Becerril-Vargas; Fernando Fontove-Herrera; Fidencio Mejía-Nepomuceno; Francisco Pulido; Gloria Elena Espinosa-Ayala; Gloria María Molina-Salinas; Gloria Vazquez; Hector Esteban Paz-Juárez; Hector Montoya-Fuentes; Helen Haydee Fernanda Ramírez-Plascencia; Irvin González-López; Jean Pierre González; Jesús Hernández; Joel Armando Vázquez-Pérez. Laboratorios Delia Barraza: María Delia Barraza Sámano; Jorge Salas-Hernández; José Antonio Enciso-Moreno; José Arturo Martínez-Orozco; José Esteban Muñoz-Medina; José de Jesús Nuñez-Contreras; Juan Bautista Chale-Dzul; Julissa Enciso-Ibarra; Luis Alberto Ochoa-Carrera; Margarita Matías-Florentino; Mario Mújica-Sánchez; Marissa Perez-Garcia; María Guadalupe Santiago-Mauricio; María Guadalupe de Jesús Mireles-Rivera; Melissa García Angulo.; Nelly Sélem-Mojica; Pavel Isa; Ricardo Ciria Merce; Ricardo Grande; Rosa María Gutiérrez Rios; Santiago Ávila-Ríos; Selene Zárate; Susana Lopez; Verónica Mata-Haro; Victor Eduardo García-Arias; Víctor Hugo Borja-Aburto
EPI_ISL_2801429	Laboratorium Diagnostyczne Centrum Medycznego Luxmed	National Institute of Public Health - National Institute of Hygiene	Gierczyńki Rafał; Sadkowska-Todys Małgorzata; Wołkowicz Tomasz
EPI_ISL_2726789	Laboratorium Diagnostyczno-Analityczne Ośrodek Profilaktyki i Epidemiologii Nowotworów im. A. Pieńkowskiej	1. National Institute of Public Health - National Institute of Hygiene, Warsaw, Poland 2. Biobank Lab, University of Lodz 3. Laboratory of Respiratory Viruses, Teaching and Clinical Center of the Medical University of Lodz	Dominik Strapagiel; Izabela Dróżdż; Jakub Lach; Katarzyna Zacharczuk; Klaudyna Królikowska; Maciej Borowiec; Magdalena Nowakowska; Magdalena Traczyk-Borszyńska; Marcin Słomka; Marta Sobalska-Kwapis; Małgorzata Sadkowska-Todys; Tomasz Płoszaj; Tomasz Wołkowicz
EPI_ISL_1611370, EPI_ISL_1683986, EPI_ISL_1684717, EPI_ISL_1799336, EPI_ISL_1801624, EPI_ISL_1926283, EPI_ISL_1927603, EPI_ISL_1928600, EPI_ISL_1929448, EPI_ISL_2045440, EPI_ISL_2045852, EPI_ISL_2045959, EPI_ISL_2183147, EPI_ISL_2306709, EPI_ISL_2306819, EPI_ISL_2306894, EPI_ISL_2307000, EPI_ISL_2307070, EPI_ISL_2398661, EPI_ISL_2398900, EPI_ISL_2399078, EPI_ISL_2399106, EPI_ISL_2399205, EPI_ISL_2399232, EPI_ISL_2480235, EPI_ISL_2480242, EPI_ISL_2480295, EPI_ISL_2480300, EPI_ISL_2480369, EPI_ISL_2480438, EPI_ISL_2480454, EPI_ISL_2480537, EPI_ISL_2480545, EPI_ISL_2480549, EPI_ISL_2480550, EPI_ISL_2480708, EPI_ISL_2482114, EPI_ISL_2482162, EPI_ISL_2482278, EPI_ISL_2611146, EPI_ISL_2611164, EPI_ISL_2611178, EPI_ISL_2611181, EPI_ISL_2611197, EPI_ISL_2611204, EPI_ISL_2611205, EPI_ISL_2611214, EPI_ISL_2611224, EPI_ISL_2611228, EPI_ISL_2611291, EPI_ISL_2611299, EPI_ISL_2611333, EPI_ISL_2611337, EPI_ISL_2611354, EPI_ISL_2611362, EPI_ISL_2611370, EPI_ISL_2611381, EPI_ISL_2611421, EPI_ISL_2611425, EPI_ISL_2611426, EPI_ISL_2872167, EPI_ISL_2872199, EPI_ISL_2872211, EPI_ISL_2872214, EPI_ISL_2872226, EPI_ISL_2872249, EPI_ISL_2872252, EPI_ISL_2872261, EPI_ISL_2872271, EPI_ISL_2872277, EPI_ISL_2872280, EPI_ISL_2872282, EPI_ISL_2872291, EPI_ISL_2872294, EPI_ISL_2872297, EPI_ISL_2872319, EPI_ISL_2872345, EPI_ISL_2872364, EPI_ISL_2872373, EPI_ISL_2872401, EPI_ISL_2872403, EPI_ISL_2872426, EPI_ISL_2872434, EPI_ISL_2872437, EPI_ISL_2872440, EPI_ISL_2872454, EPI_ISL_2872457, EPI_ISL_2872458, EPI_ISL_2872526, EPI_ISL_2872528, EPI_ISL_2872529, EPI_ISL_2872554, EPI_ISL_2872562, EPI_ISL_2872563, EPI_ISL_2872564, EPI_ISL_2872567, EPI_ISL_2872568, EPI_ISL_2872569, EPI_ISL_2872570, EPI_ISL_2872571, EPI_ISL_2872572, EPI_ISL_2872573, EPI_ISL_2872583, EPI_ISL_2872591, EPI_ISL_2872611, EPI_ISL_2872613, EPI_ISL_2872697, EPI_ISL_2872699, EPI_ISL_2872702, EPI_ISL_2872704, EPI_ISL_2872784, EPI_ISL_2872861, EPI_ISL_2873032, EPI_ISL_2873072			
see above	Laboratory Corporation of America	Centers for Disease Control and Prevention Division of Viral Diseases, Pathogen Discovery	Adrian Paskey; Amanda Douglas; Amanda Suchanek; Andrea Throop; Ayla Burns; Benjamin Rambo-Martin; Bobbi Croy; Brian Krueger; Brian Norvell; Christopher Gulvick; Christos Petropoulos; Clinton R. Paden; Craig Lukasik; Dakota Howard; Darlene Wagner; Debbie Boles; Dhwani Batra; Duncan MacCannell; Eyad Almasri; Goran Stevovic; Howard Engler; Hrushikesh Deshmukh; Jake Humphrey; Jana Schroth; Jason Caravas; Joe Voshell; John Pruitt; Jonathan Meltzer; Jonathan Williams; Kara Moser; Kimberly Wagner; Lax Iyer; Lisa Pfefferle; Lyndon Tilson; Manoj Jain; Marcia Eisenberg; Mary Ann Cristobal; Mary Williamson; Matthew Robinson; Matthew Schmerer; Michael Levandoski; Mike Sapeta; Mindy Nye; Minoo Agarwal; Mohan Kolli; Nuthawin Charoensri; Oren Cohen; Peter W. Cook; Prashant Gupta; Qian Zeng; Rama Ghatti; Scott Parker; Scott Ryan; Scott Sammons; Shatavia Morrison; Stanley Letovsky; Steven Ragan; Suresh Babu Selvaraju; Susan Countryman; Susan Hicks; Suzanne Dale; Thomas Urban; Tim Kuphal; Tricia Zwiefelhofer; Vincent Drouillon; Yvette Unoarumhi
EPI_ISL_1716736	Laboratory of Immunohematology, Division of Hematology	Greek Genome Center, Biomedical Research Foundation of the Academy of Athens (BRFAA)	Athanasia Mouzaki; Dimitrios Thanos; Emmanouil Athanasiadis; Ioannis Vatsellas; Katerina Zoi; Theodoros Loupis
EPI_ISL_2611031	Labormedizinisches Zentrum Dr Risch	Clinical Bacteriology	Adrian Egli; Alfredo Mari; Fanny Wegner; Hans Hirsch; Helena MB Seth-Smith; Julia Bielicki; Karoline Leuzinger; Lorenz Risch; Manuel Battegay; Martin Risch; Nadia Wohlwend; Tim Roloff
EPI_ISL_2677318	Labortorio Central de Saude Publica do Estado do Rio de Janeiro (LACEN/RJ)	Laboratory of Respiratory Viruses and Measles, Oswaldo Cruz Institute, FIOCRUZ	Alex Pauvolid-Corrêa; Alice Sampaio Rocha; Ana Beatriz Machado Lima; Ana Carolina Mendonca; Andrea Cony Cavalcanti; Anna Carolina Paixao; Elisa Cavalcante Pereira; Fernando Motta; Luciana Appolinario; Marilda Siqueira on behalf of the Fiocruz COVID-19 Genomic Surveillance Network; Mia Ferreira de Araujo; Paola Resende; Renata Serrano Lopes; Taina Venas
EPI_ISL_2549741, EPI_ISL_2757931, EPI_ISL_2812651, EPI_ISL_2812971, EPI_ISL_2854048	Labuan Hospital	Institute for Medical Research, Infectious Disease Research Centre, National Institutes of Health, Ministry of Health Malaysia	Anasir MI; Azizan MA; Kamel K; Mohd Zawawi Z; Ramly N; Robert F; Suppiah J; Thayan R
EPI_ISL_2760153, EPI_ISL_2760176, EPI_ISL_2761649	Landesgesundheitsamt Baden-Württemberg	Robert Koch Institute	
EPI_ISL_2876037	Laverty Pathology	NSW Health Pathology - Institute of Clinical Pathology and Medical Research; Westmead Hospital; University of Sydney	CIDM-PH et al.

EPI_ISL_2443545, EPI_ISL_2863898, EPI_ISL_2863899	Lboratorio Central de Saude Publica do Estado do Parana (LACEN/PR)	Laboratory of Respiratory Viruses and Measles, Oswaldo Cruz Institute, FIOCRUZ	Agatha Cristinne Prudencio Soares; Alice Sampaio Rocha; Ana Carolina Mendonca; Anna Carolina Paixao; Elisa Cavalcante Pereira; Fernando Motta; Ighor Leonardo Arantes Gomes; Irina Riediger; Luciana Appolinario; Marilda Siqueira on behalf of the Fiocruz COVID-19 Genomic Surveillance Network; Paola Resende; Renata Serrano Lopes; Taina Venas
EPI_ISL_2726890, EPI_ISL_2726902, EPI_ISL_2726908, EPI_ISL_2726927, EPI_ISL_2757892	Lifebrain Covid Labor GmbH	Dept. of Laboratory Medicine	Anna Gschaider; Claudia Weber; Fabian Konig; Harald Esterbauer; Oswald Wagner; Petra Jurkowitsch; Robert Strassl; Sabina Plumer
EPI_ISL_1740600, EPI_ISL_1912394, EPI_ISL_2091885, EPI_ISL_2199493, EPI_ISL_2199674, EPI_ISL_2236171, EPI_ISL_2317926, EPI_ISL_2318182, EPI_ISL_2347592, EPI_ISL_2347947, EPI_ISL_2348170, EPI_ISL_2348275, EPI_ISL_2353281, EPI_ISL_2353636, EPI_ISL_2353729, EPI_ISL_2366308, EPI_ISL_2393585, EPI_ISL_2394111, EPI_ISL_2396034, EPI_ISL_2435533, EPI_ISL_2435620, EPI_ISL_2435902, EPI_ISL_2436040, EPI_ISL_2437787, EPI_ISL_2446668, EPI_ISL_2447199, EPI_ISL_2468602, EPI_ISL_2468758, EPI_ISL_2487769, EPI_ISL_2511936, EPI_ISL_2513758, EPI_ISL_2513884, EPI_ISL_2514305, EPI_ISL_2515752, EPI_ISL_2537860, EPI_ISL_2538118, EPI_ISL_2539030, EPI_ISL_2553836, EPI_ISL_2569651, EPI_ISL_2569918, EPI_ISL_2570089, EPI_ISL_2606832, EPI_ISL_2606977, EPI_ISL_2639449, EPI_ISL_2639662, EPI_ISL_2640821, EPI_ISL_2664651, EPI_ISL_2701398, EPI_ISL_2703466, EPI_ISL_2703688, EPI_ISL_2704345, EPI_ISL_2718080, EPI_ISL_2718495, EPI_ISL_2729441, EPI_ISL_2780366, EPI_ISL_2781804, EPI_ISL_2807659, EPI_ISL_2831557, EPI_ISL_2846626, EPI_ISL_2847615, EPI_ISL_2848064, EPI_ISL_2853339			
see above	Lighthouse Lab in Alderley Park	Wellcome Sanger Institute for the COVID-19 Genomics UK (COG-UK) Consortium	Cordelia Langford; David K. Jackson; Dominic Kwiatkowski; Ewan Harrison; Ian Johnston; Jacquelyn Wynn; Jeffrey Barrett; John Sillitoe on behalf of the Wellcome Sanger Institute COVID-19 Surveillance Team; Mairead Hyland; Roberto Amato; Sonia Goncalves; The Lighthouse Lab in Alderley Park and Alex Alderton
EPI_ISL_1635379, EPI_ISL_1635400, EPI_ISL_1673100	Lighthouse Lab in Cambridge	Wellcome Sanger Institute for the COVID-19 Genomics UK (COG-UK) Consortium	Cordelia Langford; David K. Jackson; Dominic Kwiatkowski; Ewan Harrison; Ian Johnston; Jeffrey Barrett; John Sillitoe on behalf of the Wellcome Sanger Institute COVID-19 Surveillance Team; Rob Howes; Roberto Amato; Sonia Goncalves; The Lighthouse Lab in Cambridge and Alex Alderton
EPI_ISL_1635330, EPI_ISL_1741623, EPI_ISL_1741651, EPI_ISL_1741838, EPI_ISL_1806892, EPI_ISL_1829541, EPI_ISL_1830056, EPI_ISL_1830493, EPI_ISL_1831174, EPI_ISL_1912695, EPI_ISL_1912732, EPI_ISL_1939724, EPI_ISL_1939726, EPI_ISL_1939759, EPI_ISL_1939812, EPI_ISL_1987194, EPI_ISL_1987292, EPI_ISL_2021347, EPI_ISL_2022593, EPI_ISL_2022634, EPI_ISL_2022726, EPI_ISL_2022753, EPI_ISL_2092698, EPI_ISL_2092725, EPI_ISL_2092754, EPI_ISL_2120846, EPI_ISL_2122710, EPI_ISL_2122724, EPI_ISL_2122728, EPI_ISL_2122813, EPI_ISL_2122872, EPI_ISL_2152769, EPI_ISL_2152770, EPI_ISL_2152826, EPI_ISL_2152945, EPI_ISL_2153012, EPI_ISL_2198880, EPI_ISL_2199065, EPI_ISL_2199772, EPI_ISL_2199914, EPI_ISL_2199941, EPI_ISL_2199973, EPI_ISL_2235533, EPI_ISL_2235602, EPI_ISL_2236046, EPI_ISL_2236057, EPI_ISL_2236574, EPI_ISL_2236617, EPI_ISL_2237177, EPI_ISL_2237447, EPI_ISL_2237558, EPI_ISL_2237635, EPI_ISL_2237798, EPI_ISL_2238557, EPI_ISL_2275410, EPI_ISL_2275529, EPI_ISL_2275728, EPI_ISL_2316882, EPI_ISL_2317053, EPI_ISL_2317089, EPI_ISL_2318111, EPI_ISL_2318861, EPI_ISL_2318891, EPI_ISL_2353903, EPI_ISL_2353927, EPI_ISL_2353982, EPI_ISL_2354066, EPI_ISL_2354183, EPI_ISL_2354229, EPI_ISL_2354251, EPI_ISL_2354424, EPI_ISL_2395555, EPI_ISL_2395933, EPI_ISL_2396589, EPI_ISL_2407797, EPI_ISL_2407834, EPI_ISL_2407991, EPI_ISL_2436273, EPI_ISL_2438364, EPI_ISL_2438383, EPI_ISL_2438405, EPI_ISL_2438479, EPI_ISL_2447184, EPI_ISL_2457698, EPI_ISL_2457855, EPI_ISL_2469212, EPI_ISL_2487409, EPI_ISL_2488201, EPI_ISL_2488548, EPI_ISL_2513301, EPI_ISL_2514806, EPI_ISL_2542016, EPI_ISL_2543427, EPI_ISL_2552179, EPI_ISL_2553165, EPI_ISL_2553295, EPI_ISL_2553595, EPI_ISL_2554928, EPI_ISL_2554942, EPI_ISL_2554970, EPI_ISL_2555313, EPI_ISL_2555445, EPI_ISL_2555477, EPI_ISL_2568078, EPI_ISL_2607438, EPI_ISL_2607620, EPI_ISL_2608271, EPI_ISL_2639961, EPI_ISL_2639994, EPI_ISL_2641060, EPI_ISL_2641542, EPI_ISL_2651657, EPI_ISL_2651718, EPI_ISL_2682045, EPI_ISL_2682769, EPI_ISL_2683409, EPI_ISL_2683425, EPI_ISL_2701356, EPI_ISL_2701545, EPI_ISL_2718357, EPI_ISL_2729087, EPI_ISL_2730481, EPI_ISL_2730746, EPI_ISL_2730952, EPI_ISL_2731037, EPI_ISL_2731265, EPI_ISL_2807332, EPI_ISL_2807339, EPI_ISL_2807757, EPI_ISL_2808893, EPI_ISL_2808924, EPI_ISL_2814286, EPI_ISL_2815060, EPI_ISL_2815316, EPI_ISL_2846975, EPI_ISL_2850154, EPI_ISL_2850977, EPI_ISL_2851406, EPI_ISL_2852018, EPI_ISL_2852711, EPI_ISL_2852746, EPI_ISL_2852768, EPI_ISL_2853047, EPI_ISL_2866753, EPI_ISL_2866904, EPI_ISL_2868180			
see above	Lighthouse Lab in Glasgow	Wellcome Sanger Institute for the COVID-19 Genomics UK (COG-UK) Consortium	Anna Dominiczak and Alex Alderton; Carol Clugston; Cordelia Langford; David Gray; David K. Jackson; Dominic Kwiatkowski; Ewan Harrison; Harper VanSteenhouse; Ian Johnston; Jeffrey Barrett; John Sillitoe on behalf of the Wellcome Sanger Institute COVID-19 Surveillance Team; Roberto Amato; Sonia Goncalves; Yumi Kasai
EPI_ISL_1806899, EPI_ISL_1806900, EPI_ISL_1806901, EPI_ISL_1806902, EPI_ISL_1806903, EPI_ISL_1829280, EPI_ISL_1921531, EPI_ISL_1985465, EPI_ISL_1986101, EPI_ISL_2022513, EPI_ISL_2022526, EPI_ISL_2022579, EPI_ISL_2120630, EPI_ISL_2121509, EPI_ISL_2138079, EPI_ISL_2152660, EPI_ISL_2198679, EPI_ISL_2236648, EPI_ISL_2236708, EPI_ISL_2275042, EPI_ISL_2316846, EPI_ISL_2347151, EPI_ISL_2353024, EPI_ISL_2366269, EPI_ISL_2392623, EPI_ISL_2437783, EPI_ISL_2437794, EPI_ISL_2447124, EPI_ISL_2468416, EPI_ISL_2514892, EPI_ISL_2570290, EPI_ISL_2606707, EPI_ISL_2663822, EPI_ISL_2663980, EPI_ISL_2664174, EPI_ISL_2664249, EPI_ISL_2664309, EPI_ISL_2664412, EPI_ISL_2700280, EPI_ISL_2700550, EPI_ISL_2703721, EPI_ISL_2729913, EPI_ISL_2730406, EPI_ISL_2766895, EPI_ISL_2767422, EPI_ISL_2779905, EPI_ISL_2782067, EPI_ISL_2809414, EPI_ISL_2813208, EPI_ISL_2813843, EPI_ISL_2814025, EPI_ISL_2829177, EPI_ISL_2829939, EPI_ISL_2847738, EPI_ISL_2848384, EPI_ISL_2848935			
see above	Lighthouse Lab in Milton Keynes	Wellcome Sanger Institute for the COVID-19 Genomics UK (COG-UK) Consortium	Cordelia Langford; David K. Jackson; Dominic Kwiatkowski; Ewan Harrison; Ian Johnston; Jeffrey Barrett; John Sillitoe on behalf of the Wellcome Sanger Institute COVID-19 Surveillance Team; Roberto Amato; Sonia Goncalves; The Lighthouse Lab in Milton Keynes and Alex Alderton
EPI_ISL_2485422, EPI_ISL_2515896, EPI_ISL_2568930, EPI_ISL_2607022, EPI_ISL_2638459, EPI_ISL_2651497, EPI_ISL_2664830, EPI_ISL_2767580, EPI_ISL_2804003, EPI_ISL_2808309, EPI_ISL_2813568, EPI_ISL_2828866, EPI_ISL_2830722, EPI_ISL_2831905, EPI_ISL_2849733, EPI_ISL_2849817			
see above	Lighthouse Laboratory Plymouth	Wellcome Sanger Institute for the COVID-19 Genomics UK (COG-UK) Consortium	Cordelia Langford; David K. Jackson; Dominic Kwiatkowski; Ewan Harrison; Ian Johnston; Jeffrey Barrett; John Sillitoe on behalf of the Wellcome Sanger Institute COVID-19 Surveillance Team; Lighthouse Laboratory Plymouth and Alex Alderton; Roberto Amato; Sonia Goncalves
EPI_ISL_1727471, EPI_ISL_2470670, EPI_ISL_2470684, EPI_ISL_2632723, EPI_ISL_2845055	Limbach - MVZ Humangenetik Ulm	Robert Koch Institute	
EPI_ISL_2495622, EPI_ISL_2495623, EPI_ISL_2495624, EPI_ISL_2495625, EPI_ISL_2495626, EPI_ISL_2495627, EPI_ISL_2495633, EPI_ISL_2495635, EPI_ISL_2495636, EPI_ISL_2495648, EPI_ISL_2495653, EPI_ISL_2495655, EPI_ISL_2495660, EPI_ISL_2495661, EPI_ISL_2495664			
see above	MB-Cadham Provincial laboratory	National Microbiology Laboratory (NML)	Anna Majer; Anneliese Landgraff; CanCOGeN's metadata curation team; Darian Hole; David Alexander; Elsie Grudeski; Gary Van Domselaar; Grace Seo; Jared Bullard; Jennifer Tanner; Kerry Dust; Kirsten Biggar; Madison Chapel; Morag Graham; Natalie Knox; Nathalie Bastien; Paul Van Caeseele; Philip Mabon; Public Health Agency of Canada CanCOGeN team; Rhiannon Huzarewich; Russell Mandes; Shari Tyson; Timothy Booth; Yan Li
EPI_ISL_2689109	MD PHL	MD PHL	Maryland Department of Health Laboratories Administration
EPI_ISL_2471954, EPI_ISL_2471969, EPI_ISL_2635834, EPI_ISL_2845985	MDI Limbach Berlin GmbH; MVZ Labor Berlin	Robert Koch Institute	
EPI_ISL_2894130	MEDILAB	CHU Poitiers	Agnes BEBY-DEFAUX; Birama N'DIAYE; Caroline MICHAUD; Magali GARCIA; Manon PRAT; Maxime PICHON; Nicolas LEVEQUE; Valentin BON-BARET
EPI_ISL_2272597, EPI_ISL_2272600, EPI_ISL_2272602	MEPHI, Aix Marseille University	MEPHI, Aix Marseille University	Anthony LEVASSEUR
EPI_ISL_1663548, EPI_ISL_1663549	MGM Medical College, Jamshedpur	Institute of Life Sciences - INSACOG	Ajay Parida; Amol M. Kanampalliwar; Arup Ghosh; Atimukta Jha; INSACOG Consortium; Punit Prasad; Rajeeb Swain; Rupesh Dash; Safal Walia; Shifu Aggarwal; Sunil K. Raghav
EPI_ISL_2016024, EPI_ISL_2373878, EPI_ISL_2373880, EPI_ISL_2373881, EPI_ISL_2626465, EPI_ISL_2676784	MIRIALIS CLUSES BECHET	CNR Virus des Infections Respiratoires - France SUD	Antonin Bal; Bruno Lina; Gregory Destras; Gwendolyne Burfin; Hadrien Regue; Laurence Josset; Martine Valette; Quentin Semanas
EPI_ISL_2689637	MLD Szpital Św. Elżbiety	National Institute of Public Health - National Institute of Hygiene	Gierczyński Rafał; Sadkowska-Todys Małgorzata; Wołkowicz Tomasz; Zacharczuk Katarzyna
EPI_ISL_2615399, EPI_ISL_2759093, EPI_ISL_2763624, EPI_ISL_2833731, EPI_ISL_2833734, EPI_ISL_2833738, EPI_ISL_2833739			
see above	MOH - Jaber Al-Ahmad Hospital (Innovation Research Laboratory)	MOH - Jaber Al-Ahmad Hospital (Innovation Research Laboratory)	Mohammad Alghounaim; Salman Al-Sabah
EPI_ISL_1970565, EPI_ISL_1970566, EPI_ISL_1970567, EPI_ISL_1970568	MRC/UVRI & LSHTM Uganda Research Unit	MRC/UVRI & LSHTM Uganda Research Unit	Dan Lule Bugembe; Isaac Sseeewanyana; Matthew Cotten; My V.T. Phan; Patrick Semanda; Pontiano Kaleebu; Susan Nabadda
EPI_ISL_2690479, EPI_ISL_2690480, EPI_ISL_2690481, EPI_ISL_2690482, EPI_ISL_2690483, EPI_ISL_2690484, EPI_ISL_2690485, EPI_ISL_2690486, EPI_ISL_2690487, EPI_ISL_2690488, EPI_ISL_2690489, EPI_ISL_2690490, EPI_ISL_2690491, EPI_ISL_2690492, EPI_ISL_2690493, EPI_ISL_2690494, EPI_ISL_2690495, EPI_ISL_2690496, EPI_ISL_2690497, EPI_ISL_2690498, EPI_ISL_2690499, EPI_ISL_2690500, EPI_ISL_2690502, EPI_ISL_2690503, EPI_ISL_2690504, EPI_ISL_2690505, EPI_ISL_2690506, EPI_ISL_2690507, EPI_ISL_2690508, EPI_ISL_2690509, EPI_ISL_2690510, EPI_ISL_2690511, EPI_ISL_2690512, EPI_ISL_2690513			
see above	MRC/UVRI & LSHTM Uganda Research Unit, Central Public Health Laboratories, Rakai Health Sciences Program	MRC/UVRI & LSHTM Uganda Research Unit, Central Public Health Laboratories	Dan Lule Bugembe; Hellen Nansumba; Isaac Sseeewanyana; Matthew Cotten; My V.T. Phan; Patrick Semanda; Pontiano Kaleebu; Susan Nabadda
EPI_ISL_2313256, EPI_ISL_2469893	MVZ Dr. Eberhard & Partner Dortmund	Robert Koch Institute	
EPI_ISL_2111238	MVZ Labor Dr. Fenner und Kollegen (Standort Hamburg)	Robert Koch Institute	
EPI_ISL_1847444, EPI_ISL_1852081, EPI_ISL_2264588, EPI_ISL_2388139, EPI_ISL_2388159, EPI_ISL_2471905, EPI_ISL_2845215			
see above	MVZ Labor Dr. Limbach & Kollegen GbR	Robert Koch Institute	
EPI_ISL_2261471	MVZ Labor Dr. Quade & Kollegen GmbH	Robert Koch Institute	
EPI_ISL_2115263	MVZ Labor Krone GbR	Robert Koch Institute	
EPI_ISL_1850317, EPI_ISL_2314690, EPI_ISL_2316449, EPI_ISL_2845978	MVZ Medizinisches Labor Hannover GmbH	Robert Koch Institute	
EPI_ISL_2471593	MVZ für Laboratoriumsmedizin und Mikrobiologie Koblenz-Mittelrhein (Labor Koblenz)	Robert Koch Institute	
EPI_ISL_2450977, EPI_ISL_2647143, EPI_ISL_2647413, EPI_ISL_2647679, EPI_ISL_2647870, EPI_ISL_2786039, EPI_ISL_2786072, EPI_ISL_2786395, EPI_ISL_2875234, EPI_ISL_2875278			
see above	Mako Medical	Centers for Disease Control and Prevention Division of Viral Diseases, Pathogen Discovery	Adrian Paskey; Benjamin Rambo-Martin; Christopher Gulvick; Clinton R. Paden; Dakota Howard; Darlene Wagner; Dhwani Batra; Duncan MacCannell; Jason Caravas; Kara Moser; Lauren Moon; Matthew Schmerer; Matthew Tugwell; Peter W. Cook; Scott Sammons; Shatavia Morrison; Yvette Unoarumhi
EPI_ISL_2360253	Maku Molecular Laboratory	National Influenza Center	A Nejati; F Ajaminejad and T Mokhtari Azad; J Yavarian; K Sadeghi; N Ghavvami; NZ Shafiei Jandaghi; V Salimi
EPI_ISL_2501834	Maryland Genomics, Institute for Genome Sciences, University of Maryland School of Medicine	Maryland Genomics, Institute for Genome Sciences, University of Maryland School of Medicine	Claire M; Fraser; Hazen; Holly; Humphrys; Ivette; Jacques; Jonathan; Kranthi; Lim; Lisa D; Luke J; Mike; Ott; Ravel; Roussey; Sadzewicz; Sandra; Santana-Cruz; Tallon; Tracy; Vavikolanu
EPI_ISL_2008369	Massachusetts State Public Health Laboratory	Massachusetts State Public Health Laboratory	Andrew Lang; Glen Gallagher; Sandra Smole; Timelia Fink
EPI_ISL_2006677, EPI_ISL_2095274,	Max von Pettenkofer Institute, Virology, National Reference Center for Retroviruses, LMU Munich	Laboratory for Functional Genome Analysis; Dept. Genomics; Gene Center of the LMU Munich	Alexander Graf; Helmut Blum; Max Muenchhoff; Oliver Keppler; Stefan Krebs

EPI_ISL_2095280, EPI_ISL_2450280			
EPI_ISL_2389360	Med. Labor Prof. Schenk Dr. Ansorge & Kollegen	Robert Koch Institute	
EPI_ISL_2360242	Medica	Institute of Medical Virology	Alexandra Trkola; Annette Audigé; Cyril Shah; Gabriela Ziltener; Guido Bloemberg; Jon Huder; Jürg Böni; Kevin Steiner; Maria Grünberg; Maryam Zaheri; Michael Huber; Riccarda Capaul; Stefan Schmutz; Verena Kufner
EPI_ISL_2491569, EPI_ISL_2644691, EPI_ISL_2644706, EPI_ISL_2673739, EPI_ISL_2788597, EPI_ISL_2820869, EPI_ISL_2820870, EPI_ISL_2820871, EPI_ISL_2820888, EPI_ISL_2820913, EPI_ISL_2820917, EPI_ISL_2820919, EPI_ISL_2820933			
see above	Medical Microbiology Unit, Department for Laboratory Medicine, Drammen Hospital, Vestre Viken Health Trust,	Norwegian Institute of Public Health, Department of Virology	Atiya R Ali; Debech Nadia; Engebretsen Serina Beate; Garcia Llorente Ignacio; Hilde Elshaug; Hilde Vollan; Jon Bråte; Kamilla Heddeland Instefjord; Karoline Bragstad; Kathrine Stene-Johansen; Line Victoria Moen; Marie Paulsen Madsen; Olav Hungnes; Pedersen Benedikte Nevjen; Rasmus Riis Kopperud
EPI_ISL_2471761, EPI_ISL_2471770	Medizinisch-Diagnostisches Labor Kempten allgäulab	Robert Koch Institute	
EPI_ISL_2116115, EPI_ISL_2129020	Medizinische Laboratorien Dusseldorf	Robert Koch Institute	
EPI_ISL_2389094, EPI_ISL_2389096	Medizinische Laboratorien Düsseldorf	Robert Koch Institute	
EPI_ISL_2318999, EPI_ISL_2348499	Megalab, Molecular and Cytogenetics Diagnostics	Department for Virology, Molecular Biology and Genome Research, R. G. Lugar Center for Public Health Research, National Center for Disease Control and Public Health (NCDC) of Georgia.	Adam Kotorashvili; Amiran Gamkrelidze.; Ana Papkiauri; Ann Machablishvili; Anna Kasradze; Davit Tsaguria; Ekaterine Khmaladze; Ekaterine Zangaladze; Ekaterine Zhghenti; Giorgi Gogoladze; Giorgi Tomashvili; Gvantsa Brachveli; Gvantsa Chanturia; Irma Burjanadze; Ketevan Sidamonidze; Khatuna Zakhashvili; Lela Sabadze; Lela Urushadze; Magda Dgebuadze; Maia Alkhazashvili; Mari Gavashelidze; Mariam Zakalashvili; Marine Murtskhvaladze; Meri Pantsulaia; Nato Kotaria; Nino Berishvili; Nino Chikhovani; Paata Imnadze; Roena Sukhiashvili; Tamar Jashiashvili; Tata Imnadze; Tea Tevdoradze
EPI_ISL_2815327, EPI_ISL_2815328	Melaka Hospital	Institute for Medical Research, Infectious Disease Research Centre, National Institutes of Health, Ministry of Health Malaysia	Azizan MA; Kamel K; Mohd Zawawi Z; Ramly N; Robert F; Suppiah J; Thayan R
EPI_ISL_2372772, EPI_ISL_2650247	Microbiologia CATLAB	Can Ruti SARS-CoV-2 Sequencing Hub (HUGTiP/IrsiCaixa/IGTP)	Alba Sánchez; Anna Not; Antoni E Bordoy; Bonaventura Clotet; Cristina Casañ; Cristina Esteban; Francesc Catala-Moll; Gemma Clara; Ignacio Blanco; Marc Noguera-Julian; Maria Casadellà; Mariona Parera; Mercedes Guerrero; Montserrat Giménez; Pere-Joan Cardona; Pilar Armengol; Roger Pared; Roger Paredes; Verónica Saludes; and Elisa Martró on behalf of the Can Ruti SARS-CoV-2 Sequencing Hub
EPI_ISL_1913110, EPI_ISL_2405344, EPI_ISL_2482436, EPI_ISL_2482455, EPI_ISL_2598392	Microbiological Diagnostic Unit - Public Health Laboratory (MDU-PHL)	MDU-PHL	M.L.; N.L.; Sait; Seemann T.; Sherry
EPI_ISL_2281283, EPI_ISL_2281368, EPI_ISL_2281401, EPI_ISL_2448056, EPI_ISL_2650197, EPI_ISL_2650310, EPI_ISL_2671366, EPI_ISL_2671422, EPI_ISL_2681567, EPI_ISL_2812461, EPI_ISL_2812518, EPI_ISL_2832152, EPI_ISL_2832227			
see above	Microbiology Department, Laboratori Clínic Metropolitana Nord. Hospital Universitari Germans Trias i Pujol	Can Ruti SARS-CoV-2 Sequencing Hub (HUGTiP/IrsiCaixa/IGTP)	Alba Snchez; Alba Sánchez; Alexia París; Anna Not; Antoni E Bordoy; Bonaventura Clotet; Cristina Casa; Cristina Casañ; Cristina Esteban; David Panisello; Francesc Catala-Moll; Gemma Clara; Ignacio Blanco; Laia Soler; Lauro Sumoy; Marc Noguera-Julian; Maria Casadell; Maria Casadellà; Mariona Parera; Mercedes Guerrero; Montserrat Gimnez; Montserrat Giménez; Pere-Joan Cardona; Pilar Armengol; Roger Paredes; Vernica Saludes; Verónica Saludes; and Elisa Martr on behalf of the Can Ruti SARS-CoV-2 Sequencing Hub.; and Elisa Martró on behalf of the Can Ruti SARS-CoV-2 Sequencing Hub; and Elisa Martró on behalf of the Can Ruti SARS-CoV-2 Sequencing Hub.
EPI_ISL_1999791	Microbiology Department, Laboratori Clínic Metropolitana Nord. Hospital Universitari Germans Trias i Pujol.	Can Ruti SARS-CoV-2 Sequencing Hub (HUGTiP/IrsiCaixa/IGTP)	Alba Sánchez; Anna Not; Antoni E Bordoy; Bonaventura Clotet; Cristina Casañ; Cristina Esteban; Francesc Catala-Moll; Gemma Clara; Ignacio Blanco; Marc Noguera-Julian; Maria Casadellà; Mariona Parera; Mercedes Guerrero; Montserrat Giménez; Pere-Joan Cardona; Pilar Armengol; Roger Paredes; Verónica Saludes; and Elisa Martró on behalf of the Can Ruti SARS-CoV-2 Sequencing Hub.
EPI_ISL_2861321, EPI_ISL_2861322, EPI_ISL_2861323	Microbiology Department, University Hospital Donostia	Microbiology Department, University Hospital Donostia	Cilla G.; Gomez M; Marimon JM; Martin-Peñaranda T; Montes M; Piñeiro L; Sorarrain A
EPI_ISL_2086160, EPI_ISL_2501369, EPI_ISL_2501371, EPI_ISL_2501372, EPI_ISL_2501373, EPI_ISL_2501374, EPI_ISL_2861296, EPI_ISL_2861305, EPI_ISL_2861306, EPI_ISL_2861314, EPI_ISL_2864141			
see above	Microbiology Department. Complexo Hospitalario Universitario de Vigo	Microbiology Department. Complexo Hospitalario Universitario de Vigo	Alfaya N; Alonso I; Alvarez M; Cabrera JJ; Carballo R; Cores O; Cortizo S; Davina C; Martinez L; Mediero G; Perez S; Potel C; Regueiro B; Rey S; Vasallo FJ; del-Campo V
EPI_ISL_2854066	Microbiology and Virology Laboratory, "Policlinico Riuniti, Azienda Ospedaliero Universitaria, Foggia"	Microbiology and Virology Laboratory, "Policlinico Riuniti, Azienda Ospedaliero Universitaria, Foggia"	Annamaria D'Aprile; Daniela Pisanelli; Donatella Cedola; Fabio Arena; Loris Micelli; Maria Pia Patrizio; Maria Rosaria Lipsi; Maurizio Margaglione and Rosella De Nittis.; Settimia Altamura; Tiziana Rollo; Valeria Delli Carri
EPI_ISL_2362674, EPI_ISL_2362680, EPI_ISL_2362684, EPI_ISL_2362685	Microbiology and Virology Unit, Florence Careggi University Hospital	Microbiology and Virology Unit, Florence Careggi University Hospital	Alberto Antonelli; Emanuele Gori; Fabio Morecchiato; Gian Maria Rossolini; Ilaria Baccani; Marco Coppi; Nicla Giovacchini; Noemi Aiezza; Vincenzo Di Pilato
EPI_ISL_2232671, EPI_ISL_2403370, EPI_ISL_2716318, EPI_ISL_2716319	Ministry of Health Turkey	Ministry of Health Turkey	Fatma Bayrakdar; Gulay Korukluoglu; Suleyman Yalcin; Yasemin Cosgun
EPI_ISL_2776430	Ministry of Health, Jaber Al-Ahmad Hospital	Virology Unit, Department of Microbiology, Faculty of Medicine, Kuwait University	Anfal Al-Adwani; Ebaa Al-Awadhi; Hussain Safar; Nada Madi
EPI_ISL_2776369	Ministry of Health, Jaber Al-Ahmad Hospital	Virology Unit, Department of Microbiology, Faulty of Medicine, Kuwait University	Anfal Al-Adwani; Ebaa Al-Awadhi; Hussain Safar; Nada Madi
EPI_ISL_2843009, EPI_ISL_2843089, EPI_ISL_2843091, EPI_ISL_2843092, EPI_ISL_2843095, EPI_ISL_2843096, EPI_ISL_2843103, EPI_ISL_2843106, EPI_ISL_2843108, EPI_ISL_2843111, EPI_ISL_2843112, EPI_ISL_2843114, EPI_ISL_2843119, EPI_ISL_2843122, EPI_ISL_2843125, EPI_ISL_2843128, EPI_ISL_2843129, EPI_ISL_2843141, EPI_ISL_2843143, EPI_ISL_2843145, EPI_ISL_2843148, EPI_ISL_2843150, EPI_ISL_2843154, EPI_ISL_2843161, EPI_ISL_2843163, EPI_ISL_2843165, EPI_ISL_2843173, EPI_ISL_2843174, EPI_ISL_2843199, EPI_ISL_2843204, EPI_ISL_2843228, EPI_ISL_2843241			
see above	Ministry of Public Health / Hamad Medical Corporation	Biomedical Research Center (BRC), Qatar University / Qatar Genome Project (QGP)	Asmaa A. Al-Thani. MOPH and HMC: Abdullatif Al-Khal; BRC: Fatiha M. Benslimane; Chadi Saad; Dana Al-Batesh; Dina Elgakhlab QGP: Fatima H. Al-Kuwari; Einas A. E. Al-Kuwari; Hadi M. Yassine; Hamad E. Al-Romaihi; Hamda Alromaihi; Heba A. Al-Khatib; Mashael A. Al-Bader; Mohammed Al-Thani; Muna A. S. Al-Maslamani; Oal Al-Jamal; Peter V. Coyle; Reham A. El-Kahlout. QBB: Tasneem Al-Hamad; Roberto Bertollini; Salih Al-Marri
EPI_ISL_2693671	Mitra Keluarga Hospital Waru	Institute of Tropical Disease, Universitas Airlangga	Aldise M Nastri; Febria Rachmanita; Gatot Soegiarto; Herlin Ferliana; Jezzy R Dewantari; Kazufumi Shimizu; Krisnoadi Rahardjo; Laksmi Wulandari; Maria I Lusida; Resti Yudhawati; Rima R Prasetya; Soetjipto; Yasuko Mori
EPI_ISL_2724009	Molecular Biology Laboratory, Infectious Diseases Department, Instituto Nacional de Ciencias Medicas y Nutricion Salvador Zubiran	Instituto Nacional de Ciencias Medicas y Nutricion Infectious Diseases	Arturo Galindo; Eric Ochoa; Fernando Ledesma Barrientos; Guillermo Ruiz-Palacios y Santos; Patricia Leal; Pilar Ramos Cervantes; Violeta Ibarra Gonzalez
EPI_ISL_2626269, EPI_ISL_2626341, EPI_ISL_2626393	Molecular diagnostic laboratory of Federal Budget Institution of Science "Central Research Institute of Epidemiology" of The Federal Service on Customers' Rights Protection and Human Well-being Surveillance	Group of Genomics and Postgenomic Technologies of Central Research Institute of Epidemiology	Akimkin V.G.; Kaptelova V.V.; Kondrasheva L.Y.; Korneenko E.V.; Nadtoka M.I.; Saenko S.S.; Samoilov A.E.; Sinitsyn S.O.; Smirnova Y.S.; Speranskaya A.S.; Tivanova E.V.
EPI_ISL_2688325, EPI_ISL_2688357, EPI_ISL_2688365, EPI_ISL_2774088	Multiplex DX	Multiplex DX	Diana Drobna; Jakub Kovac; Pavol Cekan; Silvia Rybecka; Veronika Mancikova
EPI_ISL_2816255, EPI_ISL_2816263, EPI_ISL_2816264, EPI_ISL_2816266, EPI_ISL_2816290, EPI_ISL_2816306, EPI_ISL_2816309, EPI_ISL_2816326, EPI_ISL_2816406			
see above	N.F. Gamaleya Research Center for Epidemiology and Microbiology	WHO National Influenza Centre Russian Federation	Alexander Gintsburg; Alexey Shchetinin; Alina Odintsova; Andrei Botikov; Andrei Pochtovyi; Andrei Siniavin; Andrey Komissarov; Anna Kovyrshina; Artem Fadeev; Artem Tkachuk; Daria Danilenko; Denis Kleymenov; Denis Logunov; Dmitry Lioznov; Dmitry Shcheblyakov; Elena Mazunina; Elena Nabieva; Elena Shidlovskaya; Elizaveta Divisenko; Evgeniia Bykonia; Georgii Bazykin; Inna Dolzhikova; Kirill Varchenko; Ksenia Safina; Kseniya Komissarova; Liubov Popova; Ludmila Vasilchenko; Maria Nikiforova; Maria Pisareva; Mikhail Bakaev; Nadezhda Kuznetsova; Nikita Yolshin; Oula Mansour; Tamila Musaeva; Veronika Eder; Vladimir Gushchin
EPI_ISL_2405118	N.H.L Municipal Medical College, Ahmedabad	Gujarat Biotechnology Research Centre	Chaitanya Joshi; Dinesh Kumar; Janvi Raval; Jayshri Pethani; Madhvi Joshi; Nitesh Shah; Nitin Savaliya; Ramesh Pandit; Sonal Sharma; Twinkle Soni; Umang Mishra; Zarna Patel; Zuber Saiyed
EPI_ISL_2550605	NB-Hôpital Georges L. Dumont	National Microbiology Laboratory (NML)	Anna Majer; Anneliese Landgraff; CanCOGeN's metadata curation team; Darian Hole; Elsie Grudeski; Gary Van Domselaar; Grace Seo; Guillaume Desnoyers; Jennifer Tanner; Kirsten Biggar; Madison Chapel; Morag Graham; Natalie Knox; Nathalie Bastien; Philip Mabon; Public Health Agency of Canada CanCOGeN team; Rhiannon Huzarewich; Richard Garceau; Russell Mandes; Shari Tyson; Timothy Booth; Yan Li
EPI_ISL_1663376	NCCS, Pune	Institute of Life Sciences - INSACOG	Ajay Parida; Amol M. Kanampalliwar; Arup Ghosh; Atimukta Jha; INSACOG Consortium; Punit Prasad; Rajeeb Swain; Rupesh Dash; Safal Walia; Shifu Aggarwal; Sunil K. Raghav
EPI_ISL_2161784, EPI_ISL_2484633, EPI_ISL_2484635, EPI_ISL_2484638, EPI_ISL_2484639, EPI_ISL_2484641, EPI_ISL_2484643, EPI_ISL_2484644			
see above	NL-Dr. Leonard A. Miller Centre for Health Services	National Microbiology Laboratory (NML)	Adel Malek; Anna Majer; Anneliese Landgraff; CanCOGeN's metadata curation team; Darian Hole; Elsie Grudeski; Gary Van Domselaar; George Zahariadis; Grace Seo; Jennifer Tanner; Kerri Smith; Kirsten Biggar; Laura Gilbert; Madison Chapel; Morag Graham; Natalie Knox; Nathalie Bastien; Philip Mabon; Public Health Agency of Canada CanCOGeN team; Rhiannon Huzarewich; Robert Needle; Russell Mandes; Shari Tyson; Timothy Booth; Yan Li; Yang Yu
EPI_ISL_1482556	NORTHWELL HEALTH LABORATORIES	Wadsworth Center, New York State Department of Health	Alexis Russell; Catharine Prussing; Daryl M. Lamson; Erasmus Schneider; Erica Lasek-Nesselquist; John Kelly; Jonathan Plitnick; Kirsten St. George; Matthew Shudt; Melissa A Leisner; Navjot Singh
EPI_ISL_2162072	NS-QEII Health Sciences Centre	National Microbiology Laboratory (NML)	Anna Majer; Anneliese Landgraff; CanCOGeN's metadata curation team; Dan Gaston; Darian Hole; Elsie Grudeski; Gary Van Domselaar; Grace Seo; Janice Pettipas; Jason LeBlanc; Jennifer Tanner; Kirsten Biggar; Madison Chapel; Morag Graham; Natalie Knox; Nathalie Bastien; Philip Mabon; Public Health Agency of Canada CanCOGeN team; Rhiannon Huzarewich; Russell Mandes; Shari Tyson; Timothy Booth; Todd Hatchette; Yan Li
EPI_ISL_1678079	NYC Department of Health and Mental Hygiene	Centers for Disease Control and Prevention Division of Viral Diseases, Pathogen Discovery	Alison Laufer Halpin; Ben L. Rambo-Martin; Clinton R. Paden; Dakota Howard; Darlene Wagner; Dave Wentworth; Dhwani Batra; Jasmine Padilla; Justin Lee; Katie Dillon; Krista Queen; Kristen Knipe; Kristine Lacek; Mark Burroughs; Matthew Schmerer; Mili Sheth; Peter Cook; Sam Shepard; Sarah Nobles; Shoshona Le; Suxiang Tong; Vivien Dugan; Yvette Unoarumhi
EPI_ISL_2448957	NZOZ Medyczne Laboratorium Diagnostyczne	1. National Institute of Public Health - National Institute of Hygiene, Warsaw, Poland 2. Biobank Lab, University of Lodz 3. Laboratory of Respiratory Viruses, Teaching and Clinical Center of the Medical University of Lodz	Dominik Strapagiel; Izabela Dróżdż; Jakub Lach; Katarzyna Zacharczuk; Klaudyna Królikowska; Maciej Borowiec; Magdalena Nowakowska; Magdalena Traczyk-Borszyńska; Marcin Słomka; Marta Sobalska-Kwapis; Małgorzata Sadkowska-Todys; Tomasz Płoszaj; Tomasz Wołkowicz
EPI_ISL_2644777	Nacionalinis maisto ir veterinarijos rizikos vertinimo institutas	Institute of Biotechnology, Life Sciences Center, Vilnius University	Albertas Timinskas; Alma Gedvilaite; Danguole Ziogiene; Emilija Vasiliunaite; Milda Norkiene
EPI_ISL_2614819, EPI_ISL_2615232, EPI_ISL_2694580, EPI_ISL_2694581	Nacionalinis maisto ir veterinarijos rizikos vertinimo institutas	National Public Health Surveillance Laboratory	Ana Steponkiene; Danas Baksa; Jelena Razmuk; Lukas Vasionis; Lukas Zemaitis; Migle Gabrielaite; Svajune Muralyte

EPI_ISL_2798960	Nacionālais medicīnas serviss - laboratorija, SIA	Riga East University Hospital, National Microbiology Reference Laboratory; Eurofins Genomics Europe Sequencing GmbH	Arzu Algulieva; Diāna Dušacka; Dārta Pūpola; Ilva Pole; Inita Balta; Jevgenijs Bodrenko; Jūlija Čevere; Natalja Mikena; Reinis Vangravs; Reinis Zeltmatis; Sergejs Ņikišins; Ģirts Šķenders
EPI_ISL_2673831, EPI_ISL_2673842	Nastavni zavod za javno zdravstvo Splitsko- Dalmatinske Županije	Hrvatski zavod za javno zdravstvo	Irena Tabain; Ivana Ferenčak
EPI_ISL_2379203, EPI_ISL_2772526, EPI_ISL_2772527, EPI_ISL_2772529, EPI_ISL_2772530, EPI_ISL_2772531	National Center of Infectious and Parasitic Diseases	National Center of Infectious and Parasitic Diseases	Alexiev et al
EPI_ISL_1544014, EPI_ISL_2304085, EPI_ISL_2332708, EPI_ISL_2332739, EPI_ISL_2332740	National Centre For Cell Science	National Centre For Cell Science – INSACOG	Ajay Pillai; Dhiraj Paul; INSACOG Consortium team; Manoj Kumar Bhat; Mitali Inamdar; Mohak P Gujare; Shivang P. Bhanushali; Sonal Manik Chavan; Yogesh Shouche
EPI_ISL_2233595	National Centre for Disease Control	CDFD -INSACOG	Ashwin Dalal; Asmita Gupta; Divya Vashisht; Murali Bashyam; Pratyusha Bala; Vinay Donipadi
EPI_ISL_2460868, EPI_ISL_2461385, EPI_ISL_2461393, EPI_ISL_2461408, EPI_ISL_2461448, EPI_ISL_2461623, EPI_ISL_2461995			
see above	National Centre for Disease Control (NCDC) Biotechnology Division, Delhi	NCDC Delhi, Biotechnology Division INSACOG	Hema Gogia; Hemlata Lall; Kalaiarasan Ponnusamy; Mahesh S Dhar; Manoj K Singh; Meena Datta; Partha Rakshit; Preeti Madan; Priyanka Singh; Radhakrishnan V. S; Robin Marwal; Sandhya Kabra; Sujeet K Singh; Uma Sharma
EPI_ISL_2493032, EPI_ISL_2493068, EPI_ISL_2493069	National HIV Reference Laboratory, Ministry of Health, Public Health Institute of Malawi	CERI, Centre for Epidemic Response and Innvoation, Stellenbosch University and KRISP, KZN Research Innovation and Sequencing Platform, UKZN.	Auld A; Chilima B; Chiwaula M; Emmanuel SJ; Giandhari J; Kaba M; Kampira E; Kasambara W; Kim L; Lessells R; Maida A; Mvula B; Mwangomba W; Naidoo Y; Panja L; Pillay S; Tegally H; Wadonda N; Wilkinson E; de Oliveira T
EPI_ISL_2379748	National Heart Institute	Institute for Medical Research, Infectious Disease Research Centre, National Institutes of Health, Ministry of Health Malaysia	Azizan MA; Kamel K; Mohd Zawawi Z; Ramly N; Robert F; Suppiah J; Thayan R
EPI_ISL_2434158	National Hospital for Tropical Diseases	Oxford University Clinical Research Unit, Hanoi	H.Rogier van Doorn on behalf of the OUCRU COVID-19 research group; Le Van Duyet; Nguyen Thi Hong Thuong; Nguyen Thi Kim Chi; Nguyen Thi Tam; Nguyen Thu Trang; Pham Ngoc Thach; Phan Manh Cuong; Thomas Kesteman; Van Dinh Trang
EPI_ISL_2455221, EPI_ISL_2455222, EPI_ISL_2455223, EPI_ISL_2455224, EPI_ISL_2455233, EPI_ISL_2455234, EPI_ISL_2455235, EPI_ISL_2455238, EPI_ISL_2455239, EPI_ISL_2455240, EPI_ISL_2455398, EPI_ISL_2455399, EPI_ISL_2455415, EPI_ISL_2455416, EPI_ISL_2455451, EPI_ISL_2455479, EPI_ISL_2455480, EPI_ISL_2455481, EPI_ISL_2455482, EPI_ISL_2455483, EPI_ISL_2455484, EPI_ISL_2455485, EPI_ISL_2455486, EPI_ISL_2455487, EPI_ISL_2455488, EPI_ISL_2455489, EPI_ISL_2455490, EPI_ISL_2455491, EPI_ISL_2455492, EPI_ISL_2455494, EPI_ISL_2455495, EPI_ISL_2455496, EPI_ISL_2455497, EPI_ISL_2455554, EPI_ISL_2455559, EPI_ISL_2455560, EPI_ISL_2455561, EPI_ISL_2455583, EPI_ISL_2455585, EPI_ISL_2455587, EPI_ISL_2455677, EPI_ISL_2455895, EPI_ISL_2456004, EPI_ISL_2456330, EPI_ISL_2457173, EPI_ISL_2458057, EPI_ISL_2458058, EPI_ISL_2458059, EPI_ISL_2458060, EPI_ISL_2458061, EPI_ISL_2458062			
see above	National Hospital for Tropical Diseases	Oxford University Clinical Research Unit, Hanoi, Vietnam	H.Rogier van Doorn on behalf of the OUCRU COVID-19 research group; Le Van Duyet; Nguyen Thi Hong Thuong; Nguyen Thi Kim Chi; Nguyen Thi Tam; Nguyen Thu Trang; Pham Ngoc Thach; Phan Manh Cuong; Thomas Kesteman; Van Dinh Trang
EPI_ISL_2373108, EPI_ISL_2373109, EPI_ISL_2373110, EPI_ISL_2373111, EPI_ISL_2479934, EPI_ISL_2479935, EPI_ISL_2479936, EPI_ISL_2479937, EPI_ISL_2479938, EPI_ISL_2479939, EPI_ISL_2479940, EPI_ISL_2479941, EPI_ISL_2479942, EPI_ISL_2479943, EPI_ISL_2479944, EPI_ISL_2479945, EPI_ISL_2479946, EPI_ISL_2479947			
see above	National Influenza Center, National Institute of Hygiene and Epidemiology (NIHE)	National Influenza Center, National Institute of Hygiene and Epidemiology (NIHE)	Dang Duc Anh; Hoang Vu Mai Phuong; Le Quynh Mai; Le Thi Thanh; Nguyen Co Thach; Nguyen Le Khanh Hang; Nguyen Phuong Anh; Nguyen Thi Kieu Anh; Nguyen Thi Quyen; Nguyen Vu Son; Pham Thi Hien; Taichiro Takemura; Tran Thi Hien; Tran Thu Huong; Ung Thi Hong Trang; Vuong Duc Cuong; Vũ Hiếu
EPI_ISL_2285862	National Influenza Centre	National Influenza Centre	; Benjamiin B. Lindsey; Benjamin H. Foulkes; Dennis Laryea; Ernest Asiedu; Franklin Asiedu-Bekoe; Gordon Awandare; Ivy A. Asante; Joseph Oliver-Commey; Joyce Ngoi; Linda Boatemaa; Lorreta Kwasah; Mathew D. Parker; Michael Marks; Mildred Adusei-Poku; Sharon Hsu; Thushan I de Silva; William K. Ampofo
EPI_ISL_2099853, EPI_ISL_2308249, EPI_ISL_2566447, EPI_ISL_2566459, EPI_ISL_2566460, EPI_ISL_2687995, EPI_ISL_2687996			
see above	National Institute of Infectious Diseases-Prof. Dr. Matei Bals Molecular Diagnostics Laboratory	National Institute of Infectious Diseases-Prof. Dr. Matei Bals Molecular Diagnostics Laboratory	Andreea Tudor; Corina Casangiu; Dan Otelea; Leontina Banica; Marius Surleac; Ovidiu Vlaicu; Petre Milu; Robert Hohan; Simona Paraschiv
EPI_ISL_2433938, EPI_ISL_2796525, EPI_ISL_2796528	National Institute of Laboratory Medicine and Referral Center	Genomic Research Lab, BCSIR	Abu Sayeed Mohammad Mahmud; Arifa Akram; Asish Kumar Ghosh; Barna Goswami; Eshrar Osman; Iffat Jahan; Mahmuda Yeasmin; Md. Ahasan Habib; Md. Kamrul Islam; Md. Maruf Ahmed Molla; Md. Murshed Hasan Sarkar; Md. Salim Khan; Mohammad Mohi Uddin; Mohammad Samir Uzzaman; Shahina Akter; Tanjina Akhter Banu; Tasnim Nafisa
EPI_ISL_2015692	National Institute of Public Health - National Institute of Hygiene	1. National Institute of Public Health - National Institute of Hygiene, Warsaw, Poland 2. Biobank Lab, University of Lodz 3. Laboratory of Respiratory Viruses, Teaching and Clinical Center of the Medical University of Lodz	Dominik Strapagiel; Izabela Dróżdż; Jakub Lach; Katarzyna Zacharczuk; Klaudyna Królikowska; Maciej Borowiec; Magdalena Nowakowska; Magdalena Traczyk-Borszyńska; Marcin Słomka; Marta Sobalska-Kwapis; Małgorzata Sadkowska-Todys; Tomasz Płoszaj; Tomasz Wołkowicz
EPI_ISL_2789321	National Laboratory for Health, Environment and Food, OMM, Koper	NLZOH (National Laboratory for Health, Environment and Food) / CISLD (Clinical Institute of Special Laboratory Diagnostics), University Children's Hospital, University Medical Center Ljubljana	Aleksander Kocuvan; Aleksander Mahnic; Alenka Štorman; Ana Grom; Barbara Jenko Bizjan; Gašper Strugar; Kaja Tominc; Katarina Kozmos; Maja Rupnik; Marko Pokorn; Maruša Debeljak; Maša Jarčič; Mitja Rak / Jernej Kovač; Nika Gobec; Robert Šket; Sandra Janezic; Tadej Battelino; Tina Cvetković; Tine Tesovnik; Tjaša Žohar Čretnik
EPI_ISL_2789325, EPI_ISL_2789326	National Laboratory for Health, Environment and Food, OMM, Kranj	NLZOH (National Laboratory for Health, Environment and Food) / CISLD (Clinical Institute of Special Laboratory Diagnostics), University Children's Hospital, University Medical Center Ljubljana	Aleksander Kocuvan; Aleksander Mahnic; Alenka Štorman; Ana Grom; Barbara Jenko Bizjan; Kaja Tominc; Katarina Kozmos; Maja Rupnik; Marjana Petrevčič / Jernej Kovač; Marko Pokorn; Maruša Debeljak; Mateja Ravnik; Maša Jarčič; Monika Korošec; Nika Gobec; Robert Šket; Sandra Janezic; Tadej Battelino; Tine Tesovnik; Tjaša Žohar Čretnik
EPI_ISL_2789324	National Laboratory for Health, Environment and Food, OMM, Maribor	NLZOH (National Laboratory for Health, Environment and Food) / CISLD (Clinical Institute of Special Laboratory Diagnostics), University Children's Hospital, University Medical Center Ljubljana	Aleksander Kocuvan; Aleksander Mahnic; Alenka Štorman; Ana Grom; Andrej Golle / Jernej Kovač; Barbara Jenko Bizjan; Kaja Tominc; Katarina Kozmos; Maja Rupnik; Marko Pokorn; Maruša Debeljak; Maša Jarčič; Mojca Cimerman; Nika Gobec; Nika Volmajer; Robert Šket; Sandra Janezic; Tadej Battelino; Tine Tesovnik; Tjaša Žohar Čretnik
EPI_ISL_2756211, EPI_ISL_2895632	National Platform bis UMONS/Jolimont	National Platform bis UMONS/Jolimont	Eric Tarantino; Florian Juszczak; François Dufrasne; Gautier Detry; Guillaume Bayon-Vicente; Ruddy Wattiez
EPI_ISL_1543981, EPI_ISL_1620158, EPI_ISL_1620159, EPI_ISL_1634428, EPI_ISL_1652117, EPI_ISL_1652118, EPI_ISL_1652119, EPI_ISL_1652120, EPI_ISL_1652122, EPI_ISL_1652532, EPI_ISL_1704830, EPI_ISL_1704831, EPI_ISL_1704832, EPI_ISL_1704833, EPI_ISL_1704836, EPI_ISL_1704839, EPI_ISL_1704840, EPI_ISL_1704841, EPI_ISL_1704842, EPI_ISL_1719885, EPI_ISL_1719886, EPI_ISL_1719887, EPI_ISL_1719888, EPI_ISL_1719889, EPI_ISL_1719890, EPI_ISL_1719891, EPI_ISL_1719892, EPI_ISL_1719893, EPI_ISL_1719894, EPI_ISL_1719895, EPI_ISL_1719896, EPI_ISL_1719897, EPI_ISL_1719898, EPI_ISL_1719899, EPI_ISL_1719900, EPI_ISL_1752677, EPI_ISL_1816935, EPI_ISL_1816944, EPI_ISL_1816945, EPI_ISL_1816947, EPI_ISL_1816948, EPI_ISL_1816949, EPI_ISL_1816950, EPI_ISL_1816951, EPI_ISL_1816952, EPI_ISL_1816953, EPI_ISL_1816957, EPI_ISL_1816960, EPI_ISL_1816961, EPI_ISL_1816966, EPI_ISL_1816970, EPI_ISL_1816973, EPI_ISL_1897601, EPI_ISL_1897603, EPI_ISL_1897604, EPI_ISL_1897607, EPI_ISL_1897610, EPI_ISL_1897611, EPI_ISL_1897613, EPI_ISL_1897614, EPI_ISL_1897616, EPI_ISL_1897617, EPI_ISL_1897618, EPI_ISL_1897619, EPI_ISL_1914639, EPI_ISL_1914641, EPI_ISL_1914642, EPI_ISL_1914644, EPI_ISL_2349712, EPI_ISL_2349715, EPI_ISL_2349717, EPI_ISL_2349720, EPI_ISL_2349721, EPI_ISL_2349725, EPI_ISL_2349726, EPI_ISL_2349728, EPI_ISL_2349730, EPI_ISL_2349734, EPI_ISL_2349735, EPI_ISL_2349738, EPI_ISL_2349739, EPI_ISL_2349740, EPI_ISL_2349741, EPI_ISL_2349743, EPI_ISL_2349745, EPI_ISL_2349750, EPI_ISL_2349753, EPI_ISL_2349754, EPI_ISL_2349755, EPI_ISL_2349791, EPI_ISL_2349806, EPI_ISL_2349809, EPI_ISL_2349825, EPI_ISL_2349836, EPI_ISL_2349874, EPI_ISL_2508621, EPI_ISL_2508648, EPI_ISL_2508650, EPI_ISL_2508663, EPI_ISL_2508691, EPI_ISL_2508717, EPI_ISL_2508780, EPI_ISL_2508810, EPI_ISL_2508812, EPI_ISL_2508814, EPI_ISL_2508827, EPI_ISL_2508861, EPI_ISL_2508902, EPI_ISL_2508922, EPI_ISL_2508926, EPI_ISL_2508964, EPI_ISL_2508982, EPI_ISL_2508994, EPI_ISL_2508999, EPI_ISL_2509001, EPI_ISL_2509009, EPI_ISL_2509056, EPI_ISL_2509065, EPI_ISL_2509071, EPI_ISL_2621906, EPI_ISL_2621929, EPI_ISL_2621937, EPI_ISL_2621952, EPI_ISL_2621957, EPI_ISL_2621959, EPI_ISL_2627541, EPI_ISL_2627546, EPI_ISL_2710087, EPI_ISL_2710090, EPI_ISL_2710091, EPI_ISL_2710101, EPI_ISL_2710104, EPI_ISL_2710120, EPI_ISL_2710128, EPI_ISL_2710153, EPI_ISL_2710160, EPI_ISL_2710169, EPI_ISL_2790298, EPI_ISL_2790301, EPI_ISL_2790350, EPI_ISL_2790357, EPI_ISL_2790360, EPI_ISL_2790375, EPI_ISL_2820264, EPI_ISL_2820269, EPI_ISL_2820275, EPI_ISL_2820276, EPI_ISL_2820279, EPI_ISL_2887782, EPI_ISL_2887783, EPI_ISL_2887788, EPI_ISL_2887790, EPI_ISL_2887793			
see above	National Public Health Laboratory, National Centre for Infectious Diseases	National Public Health Laboratory, National Centre for Infectious Diseases	Grace Jie Yin Ngan; Katherine Ching; Lin Cui; Raymond Tzer Pin Lin; Royce Ang; Tze Minn Mak; Zhenyang Zhou
EPI_ISL_1921165, EPI_ISL_1921288, EPI_ISL_1921739, EPI_ISL_1921764, EPI_ISL_1921765, EPI_ISL_1921768	National Public Health Organization	Institute of Applied Biosciences, Centre for Research and Technology Hellas	Anastasia Chatzidimitriou et al.
EPI_ISL_1731198, EPI_ISL_1731330, EPI_ISL_1785273, EPI_ISL_1890977, EPI_ISL_1890990, EPI_ISL_1904652, EPI_ISL_1904670, EPI_ISL_1904703, EPI_ISL_1904829, EPI_ISL_1960669, EPI_ISL_1960691, EPI_ISL_1960795, EPI_ISL_1960834, EPI_ISL_1960854, EPI_ISL_1972735, EPI_ISL_1972833, EPI_ISL_2029353, EPI_ISL_2029354, EPI_ISL_2087953, EPI_ISL_2088054, EPI_ISL_2088086, EPI_ISL_2131856, EPI_ISL_2131879, EPI_ISL_2131897, EPI_ISL_2131943, EPI_ISL_2131950, EPI_ISL_2132155, EPI_ISL_2132165, EPI_ISL_2132169, EPI_ISL_2132188, EPI_ISL_2132215, EPI_ISL_2158539, EPI_ISL_2193835, EPI_ISL_2193895, EPI_ISL_2193909, EPI_ISL_2193937, EPI_ISL_2240844, EPI_ISL_2240882, EPI_ISL_2240899, EPI_ISL_2240912, EPI_ISL_2241055, EPI_ISL_2241056, EPI_ISL_2241078, EPI_ISL_2241082, EPI_ISL_2241134, EPI_ISL_2241253, EPI_ISL_2241268, EPI_ISL_2246823, EPI_ISL_2363042, EPI_ISL_2363072, EPI_ISL_2363194, EPI_ISL_2363199, EPI_ISL_2363211, EPI_ISL_2363288, EPI_ISL_2363291, EPI_ISL_2363297, EPI_ISL_2363307, EPI_ISL_2363322, EPI_ISL_2363324, EPI_ISL_2363328, EPI_ISL_2363379, EPI_ISL_2363380, EPI_ISL_2399958, EPI_ISL_2399961, EPI_ISL_2399962, EPI_ISL_2399963, EPI_ISL_2399964, EPI_ISL_2399974, EPI_ISL_2399978, EPI_ISL_2399979, EPI_ISL_2399991, EPI_ISL_2491119, EPI_ISL_2491120, EPI_ISL_2491121, EPI_ISL_2491123, EPI_ISL_2491124, EPI_ISL_2491127, EPI_ISL_2533639, EPI_ISL_2533690, EPI_ISL_2601108, EPI_ISL_2601114, EPI_ISL_2601120, EPI_ISL_2601126, EPI_ISL_2601130, EPI_ISL_2601146, EPI_ISL_2601147, EPI_ISL_2601207, EPI_ISL_2601214, EPI_ISL_2601392, EPI_ISL_2601402, EPI_ISL_2612906, EPI_ISL_2612909, EPI_ISL_2612910, EPI_ISL_2612911, EPI_ISL_2612912, EPI_ISL_2612913, EPI_ISL_2612915, EPI_ISL_2612922, EPI_ISL_2627615, EPI_ISL_2710640, EPI_ISL_2710652, EPI_ISL_2710707, EPI_ISL_2710708, EPI_ISL_2710709, EPI_ISL_2710711, EPI_ISL_2710713, EPI_ISL_2789505, EPI_ISL_2789507, EPI_ISL_2789538, EPI_ISL_2789541, EPI_ISL_2789543, EPI_ISL_2789549, EPI_ISL_2789560, EPI_ISL_2789561, EPI_ISL_2789587, EPI_ISL_2789595, EPI_ISL_2789613, EPI_ISL_2789619, EPI_ISL_2789620, EPI_ISL_2789621, EPI_ISL_2789623, EPI_ISL_2789634, EPI_ISL_2862354, EPI_ISL_2862358, EPI_ISL_2862371, EPI_ISL_2862381, EPI_ISL_2862399, EPI_ISL_2862406, EPI_ISL_2862422, EPI_ISL_2893038, EPI_ISL_2893057, EPI_ISL_2893072, EPI_ISL_2893075, EPI_ISL_2893086, EPI_ISL_2893089, EPI_ISL_2893090, EPI_ISL_2893093, EPI_ISL_2893099, EPI_ISL_2893102, EPI_ISL_2893106, EPI_ISL_2893127, EPI_ISL_2893133, EPI_ISL_2893134, EPI_ISL_2893149, EPI_ISL_2893156, EPI_ISL_2893164, EPI_ISL_2893173, EPI_ISL_2893189, EPI_ISL_2893190, EPI_ISL_2893202			
see above	National Virus Reference Laboratory	National Virus Reference Laboratory	Calum Walsh; Charlene Bennet; Charlene Bennett; Cillian F De Gascun; Fiona Crispie; Gabriel Gonzalez; Jonathan Dean; Matthew McCabe; Michael Carr; Paul Cotter; Zoe Yandle
EPI_ISL_1315070, EPI_ISL_1756027, EPI_ISL_1756032, EPI_ISL_2107443, EPI_ISL_2107444, EPI_ISL_2107446, EPI_ISL_2107447, EPI_ISL_2107448, EPI_ISL_2107451, EPI_ISL_2107454, EPI_ISL_2107455, EPI_ISL_2107456, EPI_ISL_2107458, EPI_ISL_2107499, EPI_ISL_2107500, EPI_ISL_2107501, EPI_ISL_2107506, EPI_ISL_2107529, EPI_ISL_2274250, EPI_ISL_2402461, EPI_ISL_2695799, EPI_ISL_2696274, EPI_ISL_2696276, EPI_ISL_2696277, EPI_ISL_2696278, EPI_ISL_2696280, EPI_ISL_2811797, EPI_ISL_2811798, EPI_ISL_2811802, EPI_ISL_2811803, EPI_ISL_2811809, EPI_ISL_2811812, EPI_ISL_2811816, EPI_ISL_2811818, EPI_ISL_2811819, EPI_ISL_2827957, EPI_ISL_2827958, EPI_ISL_2827959, EPI_ISL_2827960			
see above	New South Wales Health Pathology Royal Prince Alfred Hospital	Microbiology RPAH	Au, J.; Bull, R.; Deveson, I.; Foster, C.; Rawlinson, W.; Ruiz Silva, M.; Van Hal, S.
EPI_ISL_2644845, EPI_ISL_2644846, EPI_ISL_2644847, EPI_ISL_2644848, EPI_ISL_2645004, EPI_ISL_2645005, EPI_ISL_2645006, EPI_ISL_2645007, EPI_ISL_2645008, EPI_ISL_2645139, EPI_ISL_2645140, EPI_ISL_2645141, EPI_ISL_2645142, EPI_ISL_2645143			
see above	NordLab Kokkola	Expert Microbiology, National Institute for Health and Welfare	Carita Savolainen-Kopra; Erika Lindh; Haider al-Hello; Jani Halkilahti; Kirsi Liitsola; Niina Ikonen; Olli Vapalahti; Pekka Ellonen; Phuoc Truong; Päivi Laurila; Ravi Kant; Sari Hannula; Soile Blomqvist; Teemu Smura
EPI_ISL_2644935, EPI_ISL_2644943, EPI_ISL_2645040, EPI_ISL_2645042, EPI_ISL_2645043, EPI_ISL_2645047, EPI_ISL_2645051, EPI_ISL_2645053, EPI_ISL_2645055, EPI_ISL_2645057, EPI_ISL_2645060, EPI_ISL_2645061, EPI_ISL_2645062, EPI_ISL_2645065, EPI_ISL_2645066, EPI_ISL_2645146, EPI_ISL_2645149, EPI_ISL_2645150, EPI_ISL_2645151, EPI_ISL_2645153, EPI_ISL_2645155, EPI_ISL_2645156, EPI_ISL_2645157, EPI_ISL_2645158, EPI_ISL_2645163, EPI_ISL_2645165, EPI_ISL_2645166, EPI_ISL_2645167, EPI_ISL_2645168, EPI_ISL_2645170			
see above	NordLab Oulu	Expert Microbiology, National Institute for Health and Welfare	Carita Savolainen-Kopra; Erika Lindh; Haider al-Hello; Jani Halkilahti; Kirsi Liitsola; Niina Ikonen; Olli Vapalahti; Pekka Ellonen; Phuoc Truong; Päivi Laurila; Ravi Kant; Sari Hannula; Soile Blomqvist; Teemu Smura
EPI_ISL_2725101	Nordland Hospital - Bodo, Laboratory Department, Molecular Biology Unit	Norwegian Institute of Public Health, Department of Virology	Atiya R Ali; Debech Nadia; Engebretsen Serina Beate; Garcia Llorente Ignacio; Hilde Elshaug; Hilde Vollan; Jon Bråte; Kamilla Heddeland Instefjord; Karoline Bragstad; Kathrine Stene-Johansen; Line Victoria Moen; Marie Paulsen Madsen; Olav Hungnes; Pedersen Benedikte Nevjen; Rasmus Riis Kopperud
EPI_ISL_2518713, EPI_ISL_2518876	Northumbria University / South Tees Hospitals NHS Foundation Trust / North Cumbria Integrated Care NHS Foundation Trust / North Tees and Hartlepool NHS Foundation Trust / Newcastle Hospitals NHS Foundation Trust	COVID-19 Genomics UK (COG-UK) Consortium	Andrew Nelson; Brendan Payne; Clive Graham; Darren L Smith; Debra Padgett; Edward Barton; Emma Swindells; Garren Scott; Gary Black; Gary Eltringham; Giles S Holt; Greg R Young; Jane Greenaway; Jennifer Collins; John Allan; Joshua Loh; Lynn Dover; Matthew Bashton; Mohammad A Tariq; Paul Baker; Sarah Essex; Steve Liggett; Wen C Yew; Yusri Taha
EPI_ISL_2788596	Norwegian Institute of Public Health, Department of Virology	Oslo Helse	Atiya R Ali; Debech Nadia; Engebretsen Serina Beate; Garcia Llorente Ignacio; Hilde Elshaug; Hilde Vollan; Jon Bråte; Kamilla Heddeland Instefjord; Karoline Bragstad; Kathrine Stene-Johansen; Line Victoria Moen; Marie Paulsen Madsen; Olav Hungnes;

			Pedersen Benedikte Nevjen; Rasmus Riis Kopperud
EPI_ISL_2811720, EPI_ISL_2811721, EPI_ISL_2811722, EPI_ISL_2811723, EPI_ISL_2811724, EPI_ISL_2811725, EPI_ISL_2811726, EPI_ISL_2811728, EPI_ISL_2828472, EPI_ISL_2828477, EPI_ISL_2828479, EPI_ISL_2828480, EPI_ISL_2828481, EPI_ISL_2828482, EPI_ISL_2828483, EPI_ISL_2828484, EPI_ISL_2828485, EPI_ISL_2828486, EPI_ISL_2828487, EPI_ISL_2828488, EPI_ISL_2828489, EPI_ISL_2828490, EPI_ISL_2828491, EPI_ISL_2828492, EPI_ISL_2828493, EPI_ISL_2828494, EPI_ISL_2828495, EPI_ISL_2828497, EPI_ISL_2828499, EPI_ISL_2828500, EPI_ISL_2828505, EPI_ISL_2828506, EPI_ISL_2828507, EPI_ISL_2828508, EPI_ISL_2828509, EPI_ISL_2828510, EPI_ISL_2828511, EPI_ISL_2828512, EPI_ISL_2828513, EPI_ISL_2828514, EPI_ISL_2828515, EPI_ISL_2863942			
see above	Nucleic Acid Testing, National Reference Laboratory	GIGA Medical Genomics	Bouchra Boujemla; Esperence Umumararungu; Jacob Souopgui; Keith Durkin; Léon Mutesa; Maria Artesi; Marie-Pierre Hayette; Nathalie Renotte; Patrick Tuyisenge; Reuben Sindayiheba; Robert Rutayisire; Sabin Nsanzimana; Swaibu Gatare; Sébastien Bontems; Vincent Bours; Yvan Butera
EPI_ISL_2080768, EPI_ISL_2462074	OHSU Lab Services Molecular Microbiology Lab	Oregon SARS-CoV-2 Genome Sequencing Center	Alec J. Hirsch; Andrew C. Adey; Benjamin N. Bimber; Brendan L. O'Connell; Brian J. O'Roak; Cierra LaBlanc; Daniel N. Streblow; Donna Hansel; Guang Fan; Kayla Carter; Ruth V. Nichols; Sally Grindstaff; Sonia Acharya; William B. Messer; Xuan Qin
EPI_ISL_1904862, EPI_ISL_1904863, EPI_ISL_1904868, EPI_ISL_1904869, EPI_ISL_1904870, EPI_ISL_2017485, EPI_ISL_2017486, EPI_ISL_2017487, EPI_ISL_2017488, EPI_ISL_2017489, EPI_ISL_2017490			
see above	OLVZ Aalst	OLVZ Aalst	Anne Vankeerberghen
EPI_ISL_2893809	ORIAPOLE	CNR Virus des Infections Respiratoires - France SUD	Antonin Bal; Bruno Lina; Gregory Destras; Gwendolyne Burfin; Hadrien Regue; Laurence Josset; Martine Valette; Quentin Semanas
EPI_ISL_2757812, EPI_ISL_2840692, EPI_ISL_2840693, EPI_ISL_2840710, EPI_ISL_2840712, EPI_ISL_2840713, EPI_ISL_2840734			
see above	OSPEDALE CIVILE TERAMO - CENTRO TRASFUSIONALE	Istituto Zooprofilattico Sperimentale dell'Abruzzo e Molise "G. Caporale"	Ancora M; Calistri P; Cammà C; Caporale M; Curini V; Delli Compagni E; Di Domenico M; Di Lollo Valeria; Di Pasquale A; Lorusso A; Mangone I; Marcacci M; Puglia I; Rinaldi A; Savini G; Scialabba S
EPI_ISL_2372267	OUCRU	OUCRU	Guy Thwaites; Huynh Trung Trieu; Lam Minh Yen; Le Manh Hung; Le Nguyen Truc Nhu; Le Thi Thu Huong; Le Van Tan; Nghiem My Ngoc; Ngo Ngoc Quang Minh; Nguyen Thanh Dung; Nguyen Thanh Phong; Nguyen Thanh Truong; Nguyen Thi Han Ny; Nguyen Thi thu Hong; Nguyen To Anh; Nguyen Tri Dung; Nguyen Van Vinh Chau; Tran Nguyen Hoang Tu; Tran Tan Thanh
EPI_ISL_2406461, EPI_ISL_2801899, EPI_ISL_2801903	Oddar Meanchey Rapid Response Team	Virology Unit, Institut Pasteur du Cambodge	Cecile Troupin; Chau Darapheak; Chin Savuth; Erik A Karlsson; Jurre Y Siegers; Kraing Sidonn; Leakhena Pum; Ly Sovann; Veasna Duong; Yi Sengdoeurn
EPI_ISL_2346118	Oregon State Public Health Laboratory	Oregon State Public Health Laboratory	Eugene Yeboah; John Fontana and Shane Sevey; Laura Tsaknaridis; Rafia Razzaque; Vanda Makris
EPI_ISL_2707957	Originating lab: Wales Specialist Virology Centre Sequencing lab: Pathogen Genomics Unit	Public Health Wales Microbiology Cardiff Wales Specialist Virology Centre	Alec Birchley; Alexander Adams; Amy Gaskin; Angela Marchbank; Bree Gatica-Wilcox; Catherine Moore; Jason Coombes; Joanne Watkins; Joel Southgate; Johnathan Evans; Laura Gifford; Lauren Gilbert; Lee Graham; Malorie Perry; Matthew Bull; Nicole Pacchiarini; Sally Corden; Sara Kumziene-Summerhayes; Sara Rey; Sarah Taylor; Simon Cottrell; Sophie Jones; Tom Connor
EPI_ISL_2760840, EPI_ISL_2760842, EPI_ISL_2760844, EPI_ISL_2768840, EPI_ISL_2768841, EPI_ISL_2768842, EPI_ISL_2768843			
see above	Osaka Institute of Public Health, Morinomiya Center	Pathogen Genomics Center, National Institute of Infectious Diseases	Kentaro Itokawa; Makoto Kuroda; Masanori Hashino; Rina Tanaka; Tsuyoshi Sekizuka
EPI_ISL_2876423	Osaka Prefecture	Clinical Laboratory Medicine, Kyoto University Graduate School of Medicine	Masaki Yamamoto; Miki Nagao; Yasufumi Matsumura
EPI_ISL_2612011	Ospedale Cardinale G. Panico	Istituto Zooprofilattico Sperimentale della Puglia e della Basilicata	Bianco A.; Bruno A. R.; Capozzi L.; Del Sambro L.; Difato L.; Parisi A.; Simone D.
EPI_ISL_2602420	Ospedale San Giovanni Evangelista	INMI Lazzaro Spallanzani IRCCS	CEM Gruber; D Cerini; D Di Fusco; E Giombini; E Sperandio; F Santini; G Bonfiglio; G Orlandi; O Butera
EPI_ISL_2888017	Ospedale Sandro Pertini ASL Roma2	Department of General Diagnostics; Department of Virology; Istituto Zooprofilattico Sperimentale del Lazio e della Toscana (IZSLT)	Alessia Franco; Antonella Cersini; Antonio Battisti.; Elena L. Diaconu; Fabiola Feltrin; Giuseppe Manna; Patricia Alba; Raffaela Conti; Teresa Scicluna; Virginia Carfora
EPI_ISL_1919757, EPI_ISL_2264766, EPI_ISL_2612004, EPI_ISL_2757694, EPI_ISL_2757720	Ospedale Santa Caterina Novella	Istituto Zooprofilattico Sperimentale della Puglia e della Basilicata	Bianco A.; Bruno A.; Bruno A. R.; Capozzi L.; Del Sambro L.; Difato L.; Parisi A.; Simone D.
EPI_ISL_2466287, EPI_ISL_2612005	Ospedale Vito Fazzi	Istituto Zooprofilattico Sperimentale della Puglia e della Basilicata	Bianco A.; Bruno A. R.; Capozzi L.; Del Sambro L.; Difato L.; Lobreglio G.; Parisi A.; Simone D.
EPI_ISL_2602847	Ospedale di Genzano - ASL RM 6	INMI Lazzaro Spallanzani IRCCS	B Bartolini; E Conti; E Sperandio; F Messina; F Santini; G Bonfiglio; G Orlandi; G Tramini; O Butera
EPI_ISL_2724999, EPI_ISL_2725002	Ostfold Hospital Trust - Kalnes, Centre for Laboratory Medicine, Section for gene technology and infection serology	Norwegian Institute of Public Health, Department of Virology	Atiya R Ali; Debech Nadia; Engebretsen Serina Beate; Garcia Llorente Ignacio; Hilde Elshaug; Hilde Vollan; Jon Bråte; Kamilla Heddeland Instefjord; Karoline Bragstad; Kathrine Stene-Johansen; Line Victoria Moen; Marie Paulsen Madsen; Olav Hungnes; Pedersen Benedikte Nevjen; Rasmus Riis Kopperud
EPI_ISL_2624539	Oxford Viromics, NDM, University of Oxford; Oxford University Hospitals; Basingstoke and North Hampshire Hospital	COVID-19 Genomics UK (COG-UK) Consortium	Alex Mobbs; Amy Trebes; Anita Justice; Catrin Moore; Christophe Fraser; David Bonsall; David Buck; Emma Wise; George Macintyre; Jessica Lynch; John Todd; Mariateresa de Cesare; Matilde Mori; Monique Andersson; Nathan Moore; Nick Cortes; Robert Shaw; Stephen Kidd; Tanya Golubchik; Timothy Peto
EPI_ISL_2660900	PASTEUR MOLECULAR LABORATORY - INSACOG MEGHALAYA	CSIR-Centre for Cellular and Molecular Biology-INSACOG	Amareshwar Vodapalli; Ara Sreenivas; Archana Bharadwaj Siva; B Himasri; Divya Tej Sowpati; E Marak; E Shadap; Karthik Bharadwaj Tallapaka; Lamuk Zaveri; Onkar Kulkarni; Payel Mukherjee; Priya Nurkurthy; R. Lyngdoh; Rakesh K Mishra; Shreekant Verma; Sofia Banu; Sumedha Avadhanula; Tulasi Nagabandi; Valli Nagalakshmi Undamatla; Vidhyadhari Methuku
EPI_ISL_1824607	PKC Jagakarsa	National Institute of Health Research and Development	Arie Ardiansyah Nugraha; Hana Apsari Pawestri; Hartanti Dian Ikawati; Kartika Dewi Puspa; Krisna Pangesti; Nelly Puspandari; Subangkit; Vivi Setiawaty
EPI_ISL_1824604	PRVKP FK UI	National Institute of Health Research and Development	Arie Ardiansyah Nugraha; Hana Apsari Pawestri; Hartanti Dian Ikawati; Kartika Dewi Puspa; Krisna Pangesti; Nelly Puspandari; Subangkit; Vivi Setiawaty
EPI_ISL_1470937, EPI_ISL_2106292	Pandemic Response Lab - NYC	Pandemic Response Lab, R&D	Cybill del Castillo; Dylan Law; Haiping Hao; Henry Lee; Jon Laurent; Katharine Nelson; Melissa Hopkins; Michael Hammerling; Pradeep Bugga; Shinyoung Clair Kang; Sol Rey; William Ward
EPI_ISL_2832128	Parc Taulí Hospital Universitari	Can Ruti SARS-CoV-2 Sequencing Hub (HUGTiP/IrsiCaixa/IGTP)	Alba Sánchez; Alexia París; Anna Not; Antoni E Bordoy; Bonaventura Clotet; Cristina Casañ; David Panisello; Francesc Catala-Moll; Gemma Clara; Ignacio Blanco; Laia Soler; Lauro Sumoy; Marc Noguera-Julian; Maria Casadellà; Mariona Parera; Mercedes Guerrero; Montserrat Giménez; Pere-Joan Cardona; Pilar Armengol; Roger Paredes; Verónica Saludes; and Elisa Martró on behalf of the Can Ruti SARS-CoV-2 Sequencing Hub
EPI_ISL_2330490, EPI_ISL_2335415, EPI_ISL_2337324, EPI_ISL_2337429, EPI_ISL_2760454, EPI_ISL_2768338, EPI_ISL_2768359, EPI_ISL_2768366, EPI_ISL_2768372, EPI_ISL_2770688, EPI_ISL_2770696, EPI_ISL_2770698, EPI_ISL_2772121			
see above	Pathogen Genomics Center, National Institute of Infectious Diseases	Pathogen Genomics Center, National Institute of Infectious Diseases	Kentaro Itokawa; Makoto Kuroda; Masanori Hashino; Rina Tanaka; Tsuyoshi Sekizuka
EPI_ISL_2894148	Pathology West - NSW Health Pathology	NSW Health Pathology - Institute of Clinical Pathology and Medical Research; Westmead Hospital; University of Sydney	CIDM-PH et al.
EPI_ISL_2811984	Pekan Tajau Health Clinic	Institute for Medical Research, Infectious Disease Research Centre, National Institutes of Health, Ministry of Health Malaysia	Azizan MA; Kamel K; Mohd Zawawi Z; Ramly N; Robert F; Suppiah J; Thayan R
EPI_ISL_2558052	Philippine Red Cross - Clark Molecular Laboratory	Philippine Genome Center	Alethea R. de Guzman; Anna Ong-Lim; Arianne A. Zamora; Asia Louisa U. Chong; Benedict A. Maralit; Candice Francheska B. Tambaoan; Carlo M. Lapid; Celia Carlos; Devon Ray Pacial; Edsel Maurice Salvaña; El King D. Morado; Elcid Aaron R. Pangilinan; Eva Maria Cutiongco-de la Paz; Francis A. Tablizo; Irish Coleen A. Asin; Jaime C. Montoya; Jan Michael C. Yap; Jo-Hannah S. Llames; John Q. Wong; Joshua Gregor A. Dizon; Juan Antonio R. Magalang; Karol Sophia Agape R. Padilla; Kenneth M. Kim; Kris P. Punayan; Marc Edsel C. Ayes; Maria Rosario Singh-Vergeire and Cynthia P. Saloma; Maria Sofia L. Yangzon; Marissa Alejandria; Razel Nikka M. Hao; Renato Jacinto Q. Mantaring; Rianna Patricia S. Cruz; Sheila Mae M. Araiza
EPI_ISL_2013035	Plateforme Covid IDF, site Broussais	HEGP - Laboratoire de Virologie	David Veyer; Hélène Péré; Julien Puech; Maxime Wack; Nabil Gastli; Nicolas Robillard; Sandrine Imbeaud
EPI_ISL_2346306, EPI_ISL_2397207, EPI_ISL_2521971	Plateforme de testing Namuroise	Plateforme de testing Namuroise	Degosserie Jonathan; Demars Aurore; Denis Olivier; Lesly Nyinkeu Kemamen; Maschietto Céline; Mullier François; Nicolas Gilliard; Nobis Chloé; Otto Gaetan
EPI_ISL_1745199, EPI_ISL_2047647, EPI_ISL_2047650, EPI_ISL_2047652, EPI_ISL_2047653, EPI_ISL_2047654, EPI_ISL_2047656, EPI_ISL_2131297, EPI_ISL_2131303, EPI_ISL_2131345, EPI_ISL_2246063, EPI_ISL_2246064, EPI_ISL_2246115, EPI_ISL_2272671, EPI_ISL_2272672, EPI_ISL_2272678, EPI_ISL_2272690, EPI_ISL_2272775, EPI_ISL_2292722, EPI_ISL_2545603, EPI_ISL_2545612, EPI_ISL_2545635, EPI_ISL_2626861, EPI_ISL_2626890, EPI_ISL_2628111, EPI_ISL_2714807, EPI_ISL_2861583, EPI_ISL_2861585, EPI_ISL_2861660, EPI_ISL_2861662			
see above	Platform BIS UZA/UAntwerpen	Labo Klinische Biologie, UZA	Basil Britto Xavier; Christine Lammens; Herman Goossens; Ines Verbesselt; Jasmine Coppens; Kathleen Holemans; Marie Le Mercier; Veerle Matheeussen
EPI_ISL_2550758, EPI_ISL_2550759	Port Dickson Hospital	Institute for Medical Research, Infectious Disease Research Centre, National Institutes of Health, Ministry of Health Malaysia	Azizan MA; Kamel K; Mohd Zawawi Z; Ramly N; Robert F; Suppiah J; Thayan R
EPI_ISL_2657216	Pracownia Biologii Molekularnej Laboratorium Centralne SPZOZ	1. Tricity SARS-CoV-2 sequencing consortium: University of Gdansk, Medical University of Gdansk, Vaxican Ltd., Invicta Ltd. 2. National Institute of Public Health - National Institute of Hygiene, Warsaw, Poland	Celina Cybulska; Karolina Gackowska; Katarzyna Groth; Katarzyna Zacharczuk; Krystyna Bienkowska Szewczyk; Lukasz Rabalski; Maciej Grzybek; Maciej Kosinski; Magdalena Nowakowska; Marcin Lubocki; Małgorzata Sadkowska-Todys; Tomasz Wołkowicz
EPI_ISL_2264831, EPI_ISL_2466280, EPI_ISL_2466281, EPI_ISL_2466284, EPI_ISL_2532472, EPI_ISL_2611988, EPI_ISL_2611990, EPI_ISL_2611991, EPI_ISL_2611995, EPI_ISL_2757685, EPI_ISL_2820736, EPI_ISL_2820738			
see above	Presidio di Brindisi Di Summa - Perrino	Istituto Zooprofilattico Sperimentale della Puglia e della Basilicata	Bianco A.; Capozzi L.; Del Sambro L.; Difato L.; Giannico A.; Manzari C.; Parisi A.; Pennuzzi G.; Pesole G.; Ridolfi D.; Santoro A.; Simone D.
EPI_ISL_1789673	Pro-Vitam Diagnostics and Research Laboratory	Pro-Vitam Diagnostics and Research Laboratory	Istvan Horvath; Kinga Rakosi; Monika Korodi; Szilard N. Fejer; Zsuzsanna Jenei
EPI_ISL_2543667	Provincial Public Health Reference Laboratory (PPHRL) P&SHD	Provincial Public Health Reference Laboratory (PPHRL) P&SHD	ANDLEEB HANIF
EPI_ISL_2341869, EPI_ISL_2341881, EPI_ISL_2341890, EPI_ISL_2341898, EPI_ISL_2341907	Pt. Jawahar Lal Nehru Memorial Medical College, Raipur	Institute of Life Sciences - INSACOG	Ajay Parida; Amol M. Kanampalliwar; Arup Ghosh; Atimukta Jha; INSACOG Consortium; Omprakash Shiriwas; Punit Prasad; Rajeeb Swain; Rupesh Dash; Safal Walia; Sana Fatma; Shifu Aggarwal; Sunil K. Raghav
EPI_ISL_2709998	Public Health Authority of the Slovak Republic	Laboratory of Genomics and Bioinformatics, Comenius University Science Park	Anna Gičová; Diana Rusňáková; Jakub Styk; Jaroslav Budiš; Miroslav Böhmer; Tatiana Sedláčková; Tomáš Szemes
EPI_ISL_2699510	Public Health Laboratory, Minnesota Department of Health	University of Minnesota Genomics Center	Corbin Dirkx; Daryl M. Gohl; Jaquelyn Kuriger-Laber; John Garbe; and Sean Wang
EPI_ISL_2433928	Public Health Virology-Forensic and Scientific Services	Public Health Virology-Forensic and Scientific Services	Alyssa T. Pyke
EPI_ISL_2801898	Pursat Rapid Response Team	Virology Unit, Institut Pasteur du Cambodge	Cecile Troupin; Chau Darapheak; Chin Savuth; Erik A Karlsson; Jurre Y Siegers; Kraing Sidonn; Leakhena Pum; Ly Sovann; Veasna Duong; Yi Sengdoeurn
EPI_ISL_2854780	Puskesmas kec Menteng	National Institute of Health Research and Development	Arie Ardiansyah Nugraha; Hana Apsari Pawestri; Hartanti Dian Ikawati; Kartika Dewi Puspa; Krisna Pangesti; Nelly Puspandari; Subangkit; Triyani Soekarso; Vivi Setiawaty

EPI_ISL_2623685, EPI_ISL_2624033, EPI_ISL_2624291	Quadram Institute Bioscience	COVID-19 Genomics UK (COG-UK) Consortium	Alexander J Trotter; Alison E. Mather; Alp Aydin; Ana P. Tedim; Anastasia Kolyva; Andrew Bell; Andrew J. Page; Claire Stuart; Dave J. Baker; Gemma L. Kay; John Wain; Justin O'Grady; Leonardo de Oliveira Martins; Lizzie Meadows; Maria Diaz; Mark Webber; Muhammed Yasir; Nabil-Fareed Alikhan; Ngozi Elumogo; Nicholas M. Thomson; Rachael Stanley; Rachel Gilroy; Reenesh Prakash; Samir Dervisevic; Samuel Bloomfield; Steven Rudder; Thanh Le-Viet
EPI_ISL_1938308, EPI_ISL_2274249, EPI_ISL_2385968, EPI_ISL_2385973, EPI_ISL_2507084, EPI_ISL_2507085, EPI_ISL_2507090, EPI_ISL_2507092, EPI_ISL_2507093, EPI_ISL_2507094, EPI_ISL_2507095, EPI_ISL_2507096, EPI_ISL_2603819, EPI_ISL_2603820, EPI_ISL_2603821, EPI_ISL_2661518			
see above	Queensland Health Forensic and Scientific Services	Queensland Health Forensic and Scientific Services	Son Nguyen
EPI_ISL_1648249, EPI_ISL_1753105, EPI_ISL_1798096, EPI_ISL_1924930, EPI_ISL_1989866, EPI_ISL_2133309, EPI_ISL_2133456, EPI_ISL_2143008, EPI_ISL_2143023, EPI_ISL_2143449, EPI_ISL_2204104, EPI_ISL_2247111, EPI_ISL_2268814, EPI_ISL_2268940, EPI_ISL_2268970, EPI_ISL_2269027, EPI_ISL_2397470, EPI_ISL_2440841, EPI_ISL_2529080, EPI_ISL_2529083, EPI_ISL_2529283, EPI_ISL_2652362, EPI_ISL_2868958, EPI_ISL_2869256, EPI_ISL_2869260, EPI_ISL_2869263, EPI_ISL_2869298, EPI_ISL_2869300, EPI_ISL_2869306, EPI_ISL_2869325, EPI_ISL_2869330, EPI_ISL_2869376, EPI_ISL_2869402, EPI_ISL_2869441, EPI_ISL_2869462, EPI_ISL_2869481, EPI_ISL_2869493, EPI_ISL_2869530, EPI_ISL_2869552, EPI_ISL_2869563, EPI_ISL_2869574, EPI_ISL_2869579, EPI_ISL_2869591, EPI_ISL_2869597, EPI_ISL_2869607, EPI_ISL_2869627, EPI_ISL_2869632, EPI_ISL_2870330, EPI_ISL_2870527, EPI_ISL_2870548, EPI_ISL_2870598, EPI_ISL_2870663, EPI_ISL_2870684, EPI_ISL_2870730, EPI_ISL_2870733, EPI_ISL_2870842, EPI_ISL_2870909, EPI_ISL_2870915, EPI_ISL_2870982, EPI_ISL_2871026, EPI_ISL_2871075, EPI_ISL_2871379, EPI_ISL_2871515, EPI_ISL_2871620, EPI_ISL_2871980, EPI_ISL_2871992, EPI_ISL_2872103, EPI_ISL_2873875, EPI_ISL_2873902, EPI_ISL_2873907, EPI_ISL_2873925, EPI_ISL_2873950, EPI_ISL_2873951, EPI_ISL_2873955, EPI_ISL_2873965, EPI_ISL_2873995, EPI_ISL_2873996, EPI_ISL_2874010, EPI_ISL_2874030, EPI_ISL_2874031, EPI_ISL_2874039, EPI_ISL_2874045, EPI_ISL_2874063, EPI_ISL_2874067, EPI_ISL_2874070, EPI_ISL_2874088, EPI_ISL_2874100, EPI_ISL_2874115, EPI_ISL_2874125, EPI_ISL_2874129, EPI_ISL_2874139, EPI_ISL_2874166, EPI_ISL_2874168, EPI_ISL_2874186, EPI_ISL_2874190, EPI_ISL_2874193, EPI_ISL_2874212, EPI_ISL_2874216, EPI_ISL_2874220, EPI_ISL_2874222, EPI_ISL_2874228, EPI_ISL_2874240, EPI_ISL_2874261, EPI_ISL_2874264, EPI_ISL_2874266, EPI_ISL_2874320, EPI_ISL_2874341, EPI_ISL_2874368, EPI_ISL_2874386, EPI_ISL_2874432, EPI_ISL_2874445, EPI_ISL_2874574, EPI_ISL_2874587, EPI_ISL_2874619, EPI_ISL_2874661, EPI_ISL_2874702, EPI_ISL_2874704, EPI_ISL_2874705, EPI_ISL_2874710, EPI_ISL_2874712, EPI_ISL_2874715, EPI_ISL_2874725, EPI_ISL_2874726, EPI_ISL_2874730, EPI_ISL_2874733, EPI_ISL_2874770, EPI_ISL_2874775, EPI_ISL_2874781, EPI_ISL_2874797, EPI_ISL_2874799, EPI_ISL_2874804, EPI_ISL_2874821, EPI_ISL_2874824, EPI_ISL_2874845, EPI_ISL_2874864, EPI_ISL_2874873, EPI_ISL_2874891, EPI_ISL_2874901, EPI_ISL_2874906, EPI_ISL_2874913, EPI_ISL_2874914, EPI_ISL_2874933, EPI_ISL_2874959, EPI_ISL_2874961, EPI_ISL_2874981, EPI_ISL_2874989, EPI_ISL_2874990, EPI_ISL_2874994, EPI_ISL_2875015, EPI_ISL_2875026, EPI_ISL_2875051, EPI_ISL_2875054, EPI_ISL_2875073, EPI_ISL_2875094, EPI_ISL_2875100, EPI_ISL_2875113, EPI_ISL_2875120, EPI_ISL_2875126, EPI_ISL_2875128, EPI_ISL_2875132, EPI_ISL_2875136, EPI_ISL_2875138, EPI_ISL_2875140, EPI_ISL_2875164			
see above	Quest Diagnostics Incorporated	Centers for Disease Control and Prevention Division of Viral Diseases, Pathogen Discovery	A. Gerasimova; A. Perez; Adrian Paskey; B. Anderson; Benjamin Rambo-Martin; Christopher Gulvick; Clinton R. Paden; Dakota Howard; Darlene Wagner; Dhwani Batra; Duncan MacCannell; F. Lacbawan; I. A. Shlyakhter; Jason Caravas; K.E. Livingston; Kara Moser; L.E. Bernstein; M. Hua; Matthew Schmerer; P. Tanpaiboon; Peter W. Cook; R. M. Kagan; R. Owen; R. V. Rolando; S. H. Rosenthal; Scott Sammons; Shatavia Morrison; Y. Liu; Yvette Unoarumhi
EPI_ISL_2531894, EPI_ISL_2531897, EPI_ISL_2531901	RIIP	National Reference Center for Viruses of Respiratory Infections, Institut Pasteur, Paris	Angela Brisebarre; Camille Capel; Christophe Malabat; Corinne Maufrais; Etienne Simon-Lorière; Frédéric Lemoine; Louise Lefrançois; Marion Barbet; Maud Vanpeene; Méline Bizard; StéPhanie Guyomard-Rabenirina; Sylvie Behillil; Sylvie Van der Werf; Vincent Enouf
EPI_ISL_2784685	RM3	National Institute for Infectious Diseases (INMI) L. Spallanzani I.R.C.C.S	A Di Caro; B Bartolini; CEM Gruber; E Giombini; F Messina; F Santini; G Bonfiglio; M Rueca; MR Capobianchi; O Butera
EPI_ISL_2868902, EPI_ISL_2868910	RS Cinta Kasih Tzu Chi	National Institute of Health Research and Development	Arie Ardiansyah Nugraha; Hana Apsari Pawestri; Hartanti Dian Ikawati; Kartika Dewi Puspa; Krisna Pangesti; Nelly Puspandari; Subangkit; Triyani Soekarso; Vivi Setiawaty
EPI_ISL_2868915	RS Gading Pluit	National Institute of Health Research and Development	Arie Ardiansyah Nugraha; Hana Apsari Pawestri; Hartanti Dian Ikawati; Kartika Dewi Puspa; Krisna Pangesti; Nelly Puspandari; Subangkit; Triyani Soekarso; Vivi Setiawaty
EPI_ISL_2614728	RS Grha Kedoya	Eijkman Institute for Molecular Biology, National Research and Innovation Agency	Amin Soebandrio; Edison Johar; Frilasita A Yudhaputri; Hidayat Trimarsanto; Iskandar Adnan; Khin Saw Myint; Lidwina Priliani; Lydia V. Panggalo; Muhammad Rezki Rasyak; Safarina G Malik; Sukma Oktavianthi; Willy Agustine
EPI_ISL_2614736	RS Hermina Depok	Eijkman Institute for Molecular Biology, National Research and Innovation Agency	Amin Soebandrio; Edison Johar; Frilasita A Yudhaputri; Hidayat Trimarsanto; Iskandar Adnan; Khin Saw Myint; Lidwina Priliani; Lydia V. Panggalo; Muhammad Rezki Rasyak; Safarina G Malik; Sukma Oktavianthi; Willy Agustine
EPI_ISL_2854724	RS Hermina Depok	National Institute of Health Research and Development	; Arie Ardiansyah Nugraha; Hana Apsari Pawestri; Hartanti Dian Ikawati; Kartika Dewi Puspa; Krisna Pangesti; Nelly Puspandari; Subangkit; Triyani Soekarso; Vivi Setiawaty
EPI_ISL_2617518	RS Hermina Kemayoran	National Institute of Health Research and Development	Arie Ardiansyah Nugraha; Hana Apsari Pawestri; Hartanti Dian Ikawati; Kartika Dewi Puspa; Krisna Pangesti; Nelly Puspandari; Subangkit; Triyani Soekarso; Vivi Setiawaty
EPI_ISL_2617523	RS IMC Bintaro	National Institute of Health Research and Development	Arie Ardiansyah Nugraha; Hana Apsari Pawestri; Hartanti Dian Ikawati; Kartika Dewi Puspa; Krisna Pangesti; Nelly Puspandari; Subangkit; Triyani Soekarso; Vivi Setiawaty
EPI_ISL_2854748	RS Ichsan Medical Centre Bintaro Tangsel	National Institute of Health Research and Development	; Arie Ardiansyah Nugraha; Hana Apsari Pawestri; Hartanti Dian Ikawati; Kartika Dewi Puspa; Krisna Pangesti; Nelly Puspandari; Subangkit; Triyani Soekarso; Vivi Setiawaty
EPI_ISL_2854749, EPI_ISL_2868905	RS Kartika Pulomas	National Institute of Health Research and Development	Arie Ardiansyah Nugraha; Hana Apsari Pawestri; Hartanti Dian Ikawati; Kartika Dewi Puspa; Krisna Pangesti; Nelly Puspandari; Subangkit; Triyani Soekarso; Vivi Setiawaty
EPI_ISL_2709390, EPI_ISL_2709395	RS OMNI Pulomas	Eijkman Institute for Molecular Biology, National Research and Innovation Agency	Amin Soebandrio; Edison Johar; Frilasita A Yudhaputri; Hidayat Trimarsanto; Iskandar Adnan; Khin Saw Myint; Lidwina Priliani; Lydia V. Panggalo; Muhammad Rezki Rasyak; Safarina G Malik; Sukma Oktavianthi; Willy Agustine
EPI_ISL_2614725, EPI_ISL_2614726, EPI_ISL_2614727, EPI_ISL_2614731, EPI_ISL_2709366, EPI_ISL_2709367, EPI_ISL_2709368			
see above	RS Pelni	Eijkman Institute for Molecular Biology, National Research and Innovation Agency	Amin Soebandrio; Edison Johar; Frilasita A Yudhaputri; Hidayat Trimarsanto; Iskandar Adnan; Khin Saw Myint; Lidwina Priliani; Lydia V. Panggalo; Muhammad Rezki Rasyak; Safarina G Malik; Sukma Oktavianthi; Willy Agustine
EPI_ISL_2709394	RS Permata Cibubur	Eijkman Institute for Molecular Biology, National Research and Innovation Agency	Amin Soebandrio; Edison Johar; Frilasita A Yudhaputri; Hidayat Trimarsanto; Iskandar Adnan; Khin Saw Myint; Lidwina Priliani; Lydia V. Panggalo; Muhammad Rezki Rasyak; Safarina G Malik; Sukma Oktavianthi; Willy Agustine
EPI_ISL_2614735, EPI_ISL_2614737, EPI_ISL_2614738, EPI_ISL_2709374, EPI_ISL_2709376, EPI_ISL_2709391, EPI_ISL_2811836			
see above	RS Pondok Indah Pondok Indah	Eijkman Institute for Molecular Biology, National Research and Innovation Agency	Amin Soebandrio; Edison Johar; Frilasita A Yudhaputri; Hidayat Trimarsanto; Iskandar Adnan; Khin Saw Myint; Lidwina Priliani; Lydia V. Panggalo; Muhammad Rezki Rasyak; Safarina G Malik; Sukma Oktavianthi; Willy Agustine
EPI_ISL_2896198	RS Pusat Otak Nasional Prof dr. Mahar Mardjono	National Institute of Health Research and Development	Arie Ardiansyah Nugraha; Hana Apsari Pawestri; Hartanti Dian Ikawati; Kartika Dewi Puspa; Krisna Pangesti; Nelly Puspandari; Subangkit; Triyani Soekarso; Vivi Setiawaty
EPI_ISL_2709362, EPI_ISL_2709363, EPI_ISL_2709365, EPI_ISL_2709371, EPI_ISL_2709372, EPI_ISL_2709379, EPI_ISL_2709380			
see above	RS Pusat Pertamina	Eijkman Institute for Molecular Biology, National Research and Innovation Agency	Amin Soebandrio; Edison Johar; Frilasita A Yudhaputri; Hidayat Trimarsanto; Iskandar Adnan; Khin Saw Myint; Lidwina Priliani; Lydia V. Panggalo; Muhammad Rezki Rasyak; Safarina G Malik; Sukma Oktavianthi; Willy Agustine
EPI_ISL_2709392	RS Sentra Medika Cibinong	Eijkman Institute for Molecular Biology, National Research and Innovation Agency	Amin Soebandrio; Edison Johar; Frilasita A Yudhaputri; Hidayat Trimarsanto; Iskandar Adnan; Khin Saw Myint; Lidwina Priliani; Lydia V. Panggalo; Muhammad Rezki Rasyak; Safarina G Malik; Sukma Oktavianthi; Willy Agustine
EPI_ISL_2614732	RS Sumber Waras	Eijkman Institute for Molecular Biology, National Research and Innovation Agency	Amin Soebandrio; Edison Johar; Frilasita A Yudhaputri; Hidayat Trimarsanto; Iskandar Adnan; Khin Saw Myint; Lidwina Priliani; Lydia V. Panggalo; Muhammad Rezki Rasyak; Safarina G Malik; Sukma Oktavianthi; Willy Agustine
EPI_ISL_2262263	RS Umum Kartika Pulomas	National Institute of Health Research and Development	Arie Ardiansyah Nugraha; Hana Apsari Pawestri; Hartanti Dian Ikawati; Kartika Dewi Puspa; Krisna Pangesti; Nelly Puspandari; Subangkit; Triyani Soekarso; Vivi Setiawaty
EPI_ISL_2617513, EPI_ISL_2617516, EPI_ISL_2617517	RS Umum Medistra	National Institute of Health Research and Development	Arie Ardiansyah Nugraha; Hana Apsari Pawestri; Hartanti Dian Ikawati; Kartika Dewi Puspa; Krisna Pangesti; Nelly Puspandari; Subangkit; Triyani Soekarso; Vivi Setiawaty
EPI_ISL_2709388	RSCM	Eijkman Institute for Molecular Biology, National Research and Innovation Agency	Amin Soebandrio; Edison Johar; Frilasita A Yudhaputri; Hidayat Trimarsanto; Iskandar Adnan; Khin Saw Myint; Lidwina Priliani; Lydia V. Panggalo; Muhammad Rezki Rasyak; Safarina G Malik; Sukma Oktavianthi; Willy Agustine
EPI_ISL_2631455	RSK Paru Karawang	Indonesian Institute of Sciences (LIPI)/National Agency of Research and Innovation (BRIN) and National Institute of Health Research and Development (NIHRD)	Anggia Prasetyoputri; Eko Wahyu Putro; Herjuno A. Nugroho; Idris; Indriawati; Isa Nuryana; Listiana Oktavia; Puspita Lisdiyanti; Sri Swasthikawati; Subangkit; Syam B. Iryanto; Vivi Setiawaty; Yayuk Sri Rahayu; Yuliawati
EPI_ISL_2262283, EPI_ISL_2262285, EPI_ISL_2262288, EPI_ISL_2262289, EPI_ISL_2262291	RSPI Prof. Dr. Sulianti Saroso	National Institute of Health Research and Development	Arie Ardiansyah Nugraha; Hana Apsari Pawestri; Hartanti Dian Ikawati; Kartika Dewi Puspa; Krisna Pangesti; Nelly Puspandari; Subangkit; Triyani Soekarso; Vivi Setiawaty
EPI_ISL_2868879, EPI_ISL_2868886	RSPI Sulianti Saroso	National Institute of Health Research and Development	Arie Ardiansyah Nugraha; Hana Apsari Pawestri; Hartanti Dian Ikawati; Kartika Dewi Puspa; Krisna Pangesti; Nelly Puspandari; Subangkit; Triyani Soekarso; Vivi Setiawaty
EPI_ISL_2709393, EPI_ISL_2709398, EPI_ISL_2709399	RSU Adhyaksa	Eijkman Institute for Molecular Biology, National Research and Innovation Agency	Amin Soebandrio; Edison Johar; Frilasita A Yudhaputri; Hidayat Trimarsanto; Iskandar Adnan; Khin Saw Myint; Lidwina Priliani; Lydia V. Panggalo; Muhammad Rezki Rasyak; Safarina G Malik; Sukma Oktavianthi; Willy Agustine
EPI_ISL_2709396, EPI_ISL_2709397	RSU HGA Depok	Eijkman Institute for Molecular Biology, National Research and Innovation Agency	Amin Soebandrio; Edison Johar; Frilasita A Yudhaputri; Hidayat Trimarsanto; Iskandar Adnan; Khin Saw Myint; Lidwina Priliani; Lydia V. Panggalo; Muhammad Rezki Rasyak; Safarina G Malik; Sukma Oktavianthi; Willy Agustine
EPI_ISL_2854741, EPI_ISL_2854742, EPI_ISL_2854743, EPI_ISL_2854744	RSU Kartika Pulomas	National Institute of Health Research and Development	; Arie Ardiansyah Nugraha; Hana Apsari Pawestri; Hartanti Dian Ikawati; Kartika Dewi Puspa; Krisna Pangesti; Nelly Puspandari; Subangkit; Triyani Soekarso; Vivi Setiawaty
EPI_ISL_2854777	RSU Permata Depok	National Institute of Health Research and Development	Arie Ardiansyah Nugraha; Hana Apsari Pawestri; Hartanti Dian Ikawati; Kartika Dewi Puspa; Krisna Pangesti; Nelly Puspandari; Subangkit; Triyani Soekarso; Vivi Setiawaty
EPI_ISL_2233089	RSUD Abdul Moeis	National Institute of Health Research and Development	Arie Ardiansyah Nugraha; Hana Apsari Pawestri; Hartanti Dian Ikawati; Kartika Dewi Puspa; Krisna Pangesti; Nelly Puspandari; Subangkit; Triyani Soekarso; Vivi Setiawaty
EPI_ISL_2854747	RSUD Ciawi	National Institute of Health Research and Development	; Arie Ardiansyah Nugraha; Hana Apsari Pawestri; Hartanti Dian Ikawati; Kartika Dewi Puspa; Krisna Pangesti; Nelly Puspandari; Subangkit; Triyani Soekarso; Vivi Setiawaty
EPI_ISL_2709400	RSUD Cileungsi	Eijkman Institute for Molecular Biology, National Research and Innovation Agency	Amin Soebandrio; Edison Johar; Frilasita A Yudhaputri; Hidayat Trimarsanto; Iskandar Adnan; Khin Saw Myint; Lidwina Priliani; Lydia V. Panggalo; Muhammad Rezki Rasyak; Safarina G Malik; Sukma Oktavianthi; Willy Agustine
EPI_ISL_2502615	RSUD dr Loekmono Hadi	Genetics Working Group (Pokja Genetik) Faculty of Medicine, Public Health and Nursing Universitas Gadjah Mada (FK-KMK UGM); Disease Investigation Center Wates Ministry of Agriculture Indonesia; Department of Microbiology FK-KMK UGM; Laboratorium Diagnostik Yayasan Tahija World Mosquito Program (WMP) Yogyakarta Center for Tropical Medicine FK-KMK UGM; Integrated Research Center FK-KMK UGM; Department of Computer Science and Electronics FMIPA UGM; RSUP Dr. Sardjito	Afiahayati; Dwi AA Nugrahaningsih; Dwi Indaryati; Dyah A Puspitarani; Edwin W. Daniwijaya; Eggi Arguni; Endah Supriyati; Gunadi; Hendra Wibawa; Indarto Sulistiyono; Kristy Iskandar; Ludhang P. Rizki; Marcellus; Mohamad S. Hakim; Nungki Anggorowati; Pramesti G Dewi; Siswanto; Susan Simanjaya; Titik Nuryastuti; Tri Wibawa
EPI_ISL_2502687, EPI_ISL_2502764, EPI_ISL_2506041, EPI_ISL_2534079, EPI_ISL_2534379, EPI_ISL_2534457, EPI_ISL_2534489, EPI_ISL_2534490, EPI_ISL_2534518			
see above	RSUD dr. LOEKMONO HADI	Genetics Working Group (Pokja Genetik) Faculty of Medicine, Public Health and Nursing Universitas Gadjah Mada (FK-KMK UGM); Disease	Afiahayati; Alvina A Setiawan; Cita S Amalia; Dwi AA Nugrahaningsih; Dwi Indaryati; Dyah A Puspitarani; Edwin W. Daniwijaya; Eggi Arguni; Endah Supriyati; Gunadi; Hana F Hanifin; Hendra Wibawa; Indarto Sulistiyono; Irene Tania; Khanza a Vujira; Kristy Iskandar; Ludhang P. Rizki; Marcellus; Mohamad S. Hakim; Nungki Anggorowati; Pramesti G Dewi; Siswanto; Susan Simanjaya; Titik Nuryastuti; Tri Wibawa

		Investigation Center Wates Ministry of Agriculture Indonesia; Department of Microbiology FK-KMK UGM; Laboratorium Diagnostik Yayasan Tahija World Mosquito Program (WMP) Yogyakarta Center for Tropical Medicine FK-KMK UGM; Integrated Research Center FK-KMK UGM; Department of Computer Science and Electronics FMIPA UGM; RSUP Dr. Sardjito	
EPI_ISL_2617504	RSUPP Persahabatan Jakarta	National Institute of Health Research and Development	Arie Ardiansyah Nugraha; Hana Apsari Pawestri; Hartanti Dian Ikawati; Kartika Dewi Puspa; Krisna Pangesti; Nelly Puspandari; Subangkit; Triyani Soekarso; Vivi Setiawaty
EPI_ISL_2678103, EPI_ISL_2678104, EPI_ISL_2678105, EPI_ISL_2802834, EPI_ISL_2802835, EPI_ISL_2802841, EPI_ISL_2802846, EPI_ISL_2802853, EPI_ISL_2802854, EPI_ISL_2802855			
see above	Ramathibodi Hospital	COVID-19 Network Investigations (CONI) Alliance	Angkana Huang; Anthony R. Jones; Arporn Wangwiwatsin; Bhakbhoom Panthan; Chonticha Klungtong; Duangkamon Loesbanluechai; Ekawat Pasomsub; Elizabeth Batty; Insee Sensorn; Janjira Thaipadungpanit; Khajohn Joonlasak; Kingkan Rakmanee; Krittikorn Kumpornsin; Namfon Kotanan; Stefan Fernandez; Thanat Chookajorn; Theerarat Kochakarn; Treewat Watthanachockchai; Wasun Chantratita; Wudtichai Manasatienkij
EPI_ISL_1564356, EPI_ISL_1652717, EPI_ISL_1857699	Randox Laboratories	Wellcome Sanger Institute for the COVID-19 Genomics UK (COG-UK) Consortium	Cordelia Langford; David K. Jackson; Dominic Kwiatkowski; Ewan Harrison; Ian Johnston; Jeffrey Barrett; John Sillitoe on behalf of the Wellcome Sanger Institute COVID-19 Surveillance Team; Randox Laboratories and Alex Alderton; Roberto Amato; Sonia Goncalves
EPI_ISL_2433618	Regional Medical Science Center 2 Phitsanulok	Division of Genomic Medicine and Innovation support,Department of Medical Sciences, Ministry of Public Health, Thailand	Archawin Rojanawiwat; Jirapha Pakdee; Natthakul Bunneang; Nuanjun Wichukchinda; Penpitcha Thawong; Pilailuk Akkapaiboon Okada; Pundharika Piboonsiri; Surakameth Mahasirimongkol; Waritta Sawaengdee
EPI_ISL_2877032, EPI_ISL_2877033	Regional Medical Sciences Center 1/1 Chiangrai	National Institute of Health, Department of Medical Sciences, Ministry of Public Health, Thailand	; Natchaya Khiadsang; Nuttida Thongpramul; Pakorn Piromtong; Pilailuk Okada; Ratana Tacharoenmuang; Siripaporn Phuygun; Sittiporn Parnmen; Sunthareeya Waicharoen; Thanutsapa Thanadachakul; Warawan Wongboot; sirikanda wimol
EPI_ISL_2877034, EPI_ISL_2877035, EPI_ISL_2877036, EPI_ISL_2877037	Regional Medical Sciences Center 8 Udonthani	National Institute of Health, Department of Medical Sciences, Ministry of Public Health, Thailand	; Natchaya Khiadsang; Nuttida Thongpramul; Pakorn Piromtong; Pilailuk Okada; Ratana Tacharoenmuang; Siripaporn Phuygun; Sittiporn Parnmen; Sunthareeya Waicharoen; Thanutsapa Thanadachakul; Warawan Wongboot; sirikanda wimol
EPI_ISL_2812026, EPI_ISL_2812027	Rembau Health Clinic	Institute for Medical Research, Infectious Disease Research Centre, National Institutes of Health, Ministry of Health Malaysia	Azizan MA; Kamel K; Mohd Zawawi Z; Ramly N; Robert F; Suppiah J; Thayan R
EPI_ISL_2742191, EPI_ISL_2744951, EPI_ISL_2745419, EPI_ISL_2745736, EPI_ISL_2747215, EPI_ISL_2818034	Respiratory Virus Unit, Microbiology Services Colindale, Public Health England	COVID-19 Genomics UK (COG-UK) Consortium	PHE Covid Sequencing Team
EPI_ISL_2798352, EPI_ISL_2798988	Riga East University Hospital, National Microbiology Reference Laboratory	Riga East University Hospital, National Microbiology Reference Laboratory; Eurofins Genomics Europe Sequencing GmbH	Arzu Algulieva; Diāna Dušacka; Dārta Pūpola; Ilva Pole; Jevgenijs Bodrenko; Jūlija Čevere; Reinis Vangravs; Reinis Zeltmatis; Sergejs Ņikišins; Ģirts Šķenders
EPI_ISL_2321160, EPI_ISL_2839562, EPI_ISL_2839563	Royal Darwin Hospital Pathology	MDU-PHL	Caly L.; Druce J.; M.L.; Meumann, E.; N.L.; Sait; Seemann T.; Sherry
EPI_ISL_2250189, EPI_ISL_2250190, EPI_ISL_2250191, EPI_ISL_2250196, EPI_ISL_2250197, EPI_ISL_2250198, EPI_ISL_2250200, EPI_ISL_2250202, EPI_ISL_2250204, EPI_ISL_2250206, EPI_ISL_2250207, EPI_ISL_2250216, EPI_ISL_2250218, EPI_ISL_2250219, EPI_ISL_2250220			
see above	Royal Darwin Hospital Pathology	Microbiological Diagnostic Unit Public Health Laboratory (MDU-PHL)	Caly L.; Druce J.; M.L.; Meumann, E.; N.L.; Sait; Seemann T.; Sherry
EPI_ISL_1969246, EPI_ISL_1969247	Rumah Sakit Umum Daerah Palangkaraya	National Institute of Health Research and Development	Arie Ardiansyah Nugraha; Hana Apsari Pawestri; Hartanti Dian Ikawati; Kartika Dewi Puspa; Krisna Pangesti; Nelly Puspandari; Subangkit; Triyani Soekarso; Vivi Setiawaty
EPI_ISL_2840618	S.C. Laboratorio Analisi, ASL 3 Liguria	U.O. Igiene, Ospedale Policlinico San Martino	Bruzzone Bianca; Caligiuri Patrizia; De Pace Vanessa; Domnich Alexander; Icardi Giancarlo on behalf of SARS-CoV-2 ITALIAN RESEARCH ENTERPRISE-(SCIRE) Collaborative Group; Orsi Andrea; Ricucci Valentina; Spitaleri Antonino
EPI_ISL_2462342, EPI_ISL_2462343, EPI_ISL_2462344, EPI_ISL_2462345, EPI_ISL_2462346, EPI_ISL_2462348, EPI_ISL_2462359, EPI_ISL_2462364, EPI_ISL_2462367, EPI_ISL_2462368, EPI_ISL_2839979, EPI_ISL_2839980, EPI_ISL_2839982, EPI_ISL_2839989, EPI_ISL_2839993			
see above	SA Pathology	SA Pathology	Caitlin Selway; Chuan Kok Lim; Geoff Higgins; Ivan Bastian; Lex Leong; Mark Turra
EPI_ISL_1927305, EPI_ISL_1927400, EPI_ISL_1927401, EPI_ISL_1927403, EPI_ISL_1927404, EPI_ISL_1927405, EPI_ISL_1927407, EPI_ISL_1927408, EPI_ISL_1927410, EPI_ISL_1927411, EPI_ISL_1927413, EPI_ISL_1927414, EPI_ISL_1927416, EPI_ISL_1927417, EPI_ISL_1927419, EPI_ISL_1927420, EPI_ISL_1927422, EPI_ISL_1927423, EPI_ISL_1927424, EPI_ISL_1927426, EPI_ISL_2131671, EPI_ISL_2131672, EPI_ISL_2131673, EPI_ISL_2131676, EPI_ISL_2131677, EPI_ISL_2131691, EPI_ISL_2131692, EPI_ISL_2131693, EPI_ISL_2131694, EPI_ISL_2131695, EPI_ISL_2131696, EPI_ISL_2131697, EPI_ISL_2131698, EPI_ISL_2131701, EPI_ISL_2131702, EPI_ISL_2131703, EPI_ISL_2131704, EPI_ISL_2131705, EPI_ISL_2131706, EPI_ISL_2131707, EPI_ISL_2131708, EPI_ISL_2131709, EPI_ISL_2131712, EPI_ISL_2131714, EPI_ISL_2131716, EPI_ISL_2131717, EPI_ISL_2131719, EPI_ISL_2131724, EPI_ISL_2131725, EPI_ISL_2131728, EPI_ISL_2131730, EPI_ISL_2131731, EPI_ISL_2131732, EPI_ISL_2131733, EPI_ISL_2131734, EPI_ISL_2131736, EPI_ISL_2131738, EPI_ISL_2131744, EPI_ISL_2131748, EPI_ISL_2131764, EPI_ISL_2131769, EPI_ISL_2131770, EPI_ISL_2253111, EPI_ISL_2253113, EPI_ISL_2253121, EPI_ISL_2329929, EPI_ISL_2447617, EPI_ISL_2447667, EPI_ISL_2621755, EPI_ISL_2621756, EPI_ISL_2621759, EPI_ISL_2621760, EPI_ISL_2621761, EPI_ISL_2621770, EPI_ISL_2674810, EPI_ISL_2760890, EPI_ISL_2761075, EPI_ISL_2762358, EPI_ISL_2765503, EPI_ISL_2770204, EPI_ISL_2770210, EPI_ISL_2770211, EPI_ISL_2771592, EPI_ISL_2771593, EPI_ISL_2776922, EPI_ISL_2828255, EPI_ISL_2828259, EPI_ISL_2828260, EPI_ISL_2828261, EPI_ISL_2828264, EPI_ISL_2828267, EPI_ISL_2828270, EPI_ISL_2828271, EPI_ISL_2828272, EPI_ISL_2828274, EPI_ISL_2828275, EPI_ISL_2828276, EPI_ISL_2828279, EPI_ISL_2828280, EPI_ISL_2828281, EPI_ISL_2828289, EPI_ISL_2828293, EPI_ISL_2828301, EPI_ISL_2828307, EPI_ISL_2828308, EPI_ISL_2828309, EPI_ISL_2828310, EPI_ISL_2828311, EPI_ISL_2828314, EPI_ISL_2828316, EPI_ISL_2828317, EPI_ISL_2828318, EPI_ISL_2828319, EPI_ISL_2828325			
see above	SARS-CoV-2 testing team, National Institute of Infectious Diseases	Pathogen Genomics Center, National Institute of Infectious Diseases	Hazuka Y Furihata; Hiromizu Takahashi; Kentaro Itokawa; Makoto Kuroda; Masanori Hashino; Masumichi Saito; Naomi Nojiri; Nozomu Hanaoka; Rina Tanaka; Sana Uchikoba; Tsuguto Fujimoto; Tsuyoshi Sekizuka
EPI_ISL_1647029	SC (UCO) Igiene e Sanità Pubblica (funzione integrata con SC Microbiologia e Virologia)	ARGO Laboratorio Genomica ed Epigenomica	D'Agaro P; Dal Monego S; Degasperi M; Licastro D; Lombardo F
EPI_ISL_2828396, EPI_ISL_2828398	SC (UCO) Igiene e Sanità Pubblica, ASUGI, Trieste	ARGO Laboratorio Genomica ed Epigenomica	Barbone F; Breda C; Busetti M; D'Agaro P; Dal Monego S; Degasperi M; Fontana F; Licastro D; Marcello A; Piscianz E; Segat L
EPI_ISL_2015915	SELARL MIRIALIS CLUSES	CNR Virus des Infections Respiratoires - France SUD	Antonin Bal; Bruno Lina; Gregory Destras; Gwendolyne Burfin; Hadrien Regue; Laurence Josset; Martine Valette; Quentin Semanas
EPI_ISL_2686086	SELAS LABOSUD	Inovie AS GenBio	VERCRUYSSEN Florian LAURENT Pauline
EPI_ISL_2795862, EPI_ISL_2795878, EPI_ISL_2878250, EPI_ISL_2878295, EPI_ISL_2878417	SELAS MEDILYS	Department of Virology, Henri Mondor University Hospital, Assistance Publique Hôpitaux de Paris, Université Paris-Est Créteil, INSERM U955	Alexandre Soulier; Christophe Rodriguez; Elisabeth Trawinski; Guillaume Gricourt; Jean-Michel Pawlotsky; Melissa N'Debi; Slim Fourati; Vanessa Demontant
EPI_ISL_2695241, EPI_ISL_2695243, EPI_ISL_2695247	SESARAM	Instituto Nacional de Saude (INSA)	Borges et al
EPI_ISL_2536217, EPI_ISL_2828727	SISP Lecce	University of Bari Biomedical Sciences and Human Oncology	Maria Chironna
EPI_ISL_2587054, EPI_ISL_2587399, EPI_ISL_2889292, EPI_ISL_2889298, EPI_ISL_2889308, EPI_ISL_2889422, EPI_ISL_2889433, EPI_ISL_2889448, EPI_ISL_2889456, EPI_ISL_2889457, EPI_ISL_2889481, EPI_ISL_2889486, EPI_ISL_2889489, EPI_ISL_2889492, EPI_ISL_2889544, EPI_ISL_2889567, EPI_ISL_2889596, EPI_ISL_2889608, EPI_ISL_2889609, EPI_ISL_2889613, EPI_ISL_2889626, EPI_ISL_2889672, EPI_ISL_2889677, EPI_ISL_2889692, EPI_ISL_2889693, EPI_ISL_2889747, EPI_ISL_2889755, EPI_ISL_2889770, EPI_ISL_2889814			
see above	SK-Roy Romanow Provincial Laboratory	National Microbiology Laboratory (NML)	Alanna Senecal; Amanda Lang; Anna Majer; Anneliese Landgraff; CanCOGeN's metadata curation team; Darian Hole; Elsie Grudeski; Gary Van Domselaar; Grace Seo; Jennifer Tanner; Jessica Minion; Kara Loos; Keith MacKenzie; Kirsten Biggar; Madison Chapel; Meredith Faires; Morag Graham; Natalie Knox; Nathalie Bastien; Philip Mabon; Public Health Agency of Canada CanCOGeN team; Rachel DePaulo; Rhiannon Huzarewich; Russell Mandes; Ryan McDonald; Shari Tyson; Timothy Booth; Yan Li
EPI_ISL_1919655, EPI_ISL_2612837, EPI_ISL_2685852, EPI_ISL_2685873	SYNLAB	GIGA Medical Genomics	Bouchra Boujemla; Cécile Meex; Keith Durkin; Maria Artesi; Marie-Pierre Hayette; Nathalie Renotte; Pierrette Melin; Raphaël Boreux; Sébastien Bontems; Vincent Bours
EPI_ISL_2536117, EPI_ISL_2536119, EPI_ISL_2536120, EPI_ISL_2810490	SYNLAB	Instituto Nacional de Saude (INSA)	Borges et al
EPI_ISL_2123616, EPI_ISL_2123713	SYNLAB Labor MÃ¼nchen Zentrum LMZ	Robert Koch Institute	
EPI_ISL_2635435	SYNLAB MVZ Dachau	Robert Koch Institute	
EPI_ISL_2115085, EPI_ISL_2128794	SYNLAB MVZ Heidelberg	Robert Koch Institute	
EPI_ISL_2126453, EPI_ISL_2126465, EPI_ISL_2389726, EPI_ISL_2844928	SYNLAB MVZ Weiden	Robert Koch Institute	
EPI_ISL_2557178, EPI_ISL_2557188, EPI_ISL_2557189, EPI_ISL_2557190, EPI_ISL_2557191, EPI_ISL_2557192, EPI_ISL_2644992			
see above	SYNLAB Suomi	Expert Microbiology, National Institute for Health and Welfare	Carita Savolainen-Kopra; Erika Lindh; Haider al-Hello; Jani Halkilahti; Kirsi Liitsola; Niina Ikonen; Olli Vapalahti; Pekka Ellonen; Phuoc Truong; Päivi Laurila; Ravi Kant; Sari Hannula; Soile Blomqvist; Teemu Smura
EPI_ISL_2812145	Saitama Prefectural Institute of Public Health	Saitama Prefectural Institute of Public Health	Eri Aonuma; Hayato Ehara; Kentaro Itokawa; Makoto Kuroda; Masanori Hashino; Rina Tanaka; Satoshi Daisaki; Tsuyoshi Sekizuka
EPI_ISL_2349933, EPI_ISL_2444444, EPI_ISL_2444483, EPI_ISL_2550746, EPI_ISL_2603690, EPI_ISL_2603708, EPI_ISL_2692592, EPI_ISL_2692743, EPI_ISL_2692784, EPI_ISL_2692791, EPI_ISL_2810099, EPI_ISL_2810123, EPI_ISL_2810248, EPI_ISL_2810249, EPI_ISL_2810268, EPI_ISL_2894415			
see above	Salud Digna	Instituto Nacional de Medicina Genomica	Abraham Campos-Romero; Cedro-Tanda A; Escobar-Arrazola; Gonzalez-Barrera D; Herrera-Montalvo LA.; Hidalgo-Miranda A; Luna-Ruiz Marco; M; Mendoza-Vargas A; Moreno-Camacho José Luis; Munguia-Garza P; Ramirez-Vega O; Rangel-DeLeon D; Reyes-Grajeda JP; Rodriguez-Gallegos Jorge

EPI_ISL_2035737, EPI_ISL_2035739, EPI_ISL_2035744, EPI_ISL_2035746, EPI_ISL_2035747, EPI_ISL_2035748, EPI_ISL_2420473			
see above	San Gallicano Dermatological Institute I.F.O.	INMI Lazzaro Spallanzani IRCCS	A Di Caro; B Bartolini; CEM Gruber; E Giombini; E Trento; EG Di Domenico; F Ensoli; F Messina; F Pimpinelli; F Santini; G Bonfiglio; G D'Agosto; G Prignano; M Pontone; M Rueca; MR Capobianchi; O Butera
EPI_ISL_2691268	Santa Clara Valley Medical Center	Santa Clara County Public Health Laboratory	Santa Clara County Public Health Department
EPI_ISL_2140071	Servicio Virosis Respiratorias-Departamento Virología-INEI	Instituto Nacional Enfermedades Infecciosas C.G.Malbran	Avaro M.; Baumeister E.; Benedetti E.; Campos J.; Cisterna D.; Dattero ME; Lorenzo F.; Molina V.; Perandones C.; Poklepovich T.; Pontoriero A.; Russo M.; Tuduri E.
EPI_ISL_2426646, EPI_ISL_2426647, EPI_ISL_2558004, EPI_ISL_2558005, EPI_ISL_2558006, EPI_ISL_2558042, EPI_ISL_2689692, EPI_ISL_2689695, EPI_ISL_2689730, EPI_ISL_2689734, EPI_ISL_2758576, EPI_ISL_2861038			
see above	Servicio de Microbiologia Hospital Ramon y Cajal	Servicio de Microbiologia Hospital Ramon y Cajal	Galan JC; Martinez L. Abreu M; Ponce M; y Gonzalez-Alba JM
EPI_ISL_2510580, EPI_ISL_2516722	Servicio de Microbiología Clínica (Complejo Hospitalario de Navarra, Pamplona)	Centro de Secuenciación NASERTIC	Ana Miqueleiz; Ana Navascués; Carmen Ezpeleta Baquedano
EPI_ISL_2658260	Servicio de Microbiología. Consorcio Hospital General Universitario de Valencia	SeqCOVID-SPAIN consortium/IBV(CSIC)	Begoña Fuster Escrivá; Carme Salvador García; Concepción Gimeno Cardona and SeqCOVID-SPAIN consortium; María Dolores Ocete; Rafael Medina González
EPI_ISL_2600916	Servicio de Microbiología. Hospital Clínico Universitario de Valencia	SeqCOVID-SPAIN consortium/IBV(CSIC)	David Navarro Ortega; Eliseo Albert Vicent; Ignacio Torres and SeqCOVID-SPAIN consortium
EPI_ISL_2627516, EPI_ISL_2627521	Servicio de Microbiología. Hospital Universitario Doctor Peset	SeqCOVID-SPAIN consortium/IBV(CSIC)	José Miguel Nogueira Coito and SeqCOVID-SPAIN consortium; Juan Alberola Enguídanos; Juan José Camarena Miñana; Rosa González Pellicer
EPI_ISL_2360252, EPI_ISL_2360254	Shahid Ghodossi Laboratory	National Influenza Center	A Nejati; F Ajaminejad; F Ajaminejad and T Mokhtari Azad; J Yavarian; K Sadeghi; N Ghavvami; N Ghavvami and T Mokhtari Azad; NZ Shafiei Jandaghi; V Salimi
EPI_ISL_2545226, EPI_ISL_2545227, EPI_ISL_2545236, EPI_ISL_2545240, EPI_ISL_2545243, EPI_ISL_2545247, EPI_ISL_2545251, EPI_ISL_2545264, EPI_ISL_2545265, EPI_ISL_2545267, EPI_ISL_2545270, EPI_ISL_2545276, EPI_ISL_2545277, EPI_ISL_2545292, EPI_ISL_2545295, EPI_ISL_2545296, EPI_ISL_2545297, EPI_ISL_2545299, EPI_ISL_2545302, EPI_ISL_2545303, EPI_ISL_2567007, EPI_ISL_2567021, EPI_ISL_2567040, EPI_ISL_2567042, EPI_ISL_2567071, EPI_ISL_2811423, EPI_ISL_2811429, EPI_ISL_2811449, EPI_ISL_2811452, EPI_ISL_2811456, EPI_ISL_2811459, EPI_ISL_2811467, EPI_ISL_2811471, EPI_ISL_2811473, EPI_ISL_2811487, EPI_ISL_2811493, EPI_ISL_2811504, EPI_ISL_2811535, EPI_ISL_2811565, EPI_ISL_2811577, EPI_ISL_2811579, EPI_ISL_2811600, EPI_ISL_2811610, EPI_ISL_2811642, EPI_ISL_2811644, EPI_ISL_2811664, EPI_ISL_2811678, EPI_ISL_2811690, EPI_ISL_2811712, EPI_ISL_2834268, EPI_ISL_2834270, EPI_ISL_2834272, EPI_ISL_2834273, EPI_ISL_2834274, EPI_ISL_2834276, EPI_ISL_2834281, EPI_ISL_2834282, EPI_ISL_2834284, EPI_ISL_2834285, EPI_ISL_2834288, EPI_ISL_2834289, EPI_ISL_2834309, EPI_ISL_2834311, EPI_ISL_2834314, EPI_ISL_2834316, EPI_ISL_2834322, EPI_ISL_2834326, EPI_ISL_2834332, EPI_ISL_2834334, EPI_ISL_2834337, EPI_ISL_2834339, EPI_ISL_2834344, EPI_ISL_2834345, EPI_ISL_2834346, EPI_ISL_2834350, EPI_ISL_2834352, EPI_ISL_2834353, EPI_ISL_2834384, EPI_ISL_2834387, EPI_ISL_2834392, EPI_ISL_2834393, EPI_ISL_2834400, EPI_ISL_2834407, EPI_ISL_2834408, EPI_ISL_2834414, EPI_ISL_2834416, EPI_ISL_2834423, EPI_ISL_2834426, EPI_ISL_2834428, EPI_ISL_2834437, EPI_ISL_2834438, EPI_ISL_2834439, EPI_ISL_2834447, EPI_ISL_2834449, EPI_ISL_2834450, EPI_ISL_2834458, EPI_ISL_2834470, EPI_ISL_2834474, EPI_ISL_2834482, EPI_ISL_2834489, EPI_ISL_2834493, EPI_ISL_2834494, EPI_ISL_2834496, EPI_ISL_2834500, EPI_ISL_2834511, EPI_ISL_2834517, EPI_ISL_2834519, EPI_ISL_2834522, EPI_ISL_2834524			
see above	Shamir Medical Center (Asaf Harofe)	Shamir Medical Center (Asaf Harofe)	Abu Hamad Ramzia; Adina Bar Chaim; Anna Vishnevsky; Chen Weiner; Netta Zuckerman; Nir Rainy; Patricia Benveniste-Lekovitz; Reut Sorek Abramovich; Yevgeni Yegorov
EPI_ISL_2608422, EPI_ISL_2608423	Siem Reap Provinical Laboratory	Virology Unit, Institut Pasteur du Cambodge	Cecile Troupin; Chau Darapheak; Chin Savuth; Erik A Karlsson; Jurre Y Siegers; Kraing Sidonn; Leakhena Pum; Ly Sovann; Veasna Duong; Yi Sengdoeurn
EPI_ISL_2566445	Singapore General Hospital	Department of Microbiology	Chayaporn Suphavilai; James Sim Heng Chiak; Karrie Ko; Kenneth Xin Long Chan; Kern Rei Chng; Kian Sing Chan; Kun Lee Lim; Lynette Oon; Niranjan Nagarajan; Nurdyana Abdul Rahman; Sui Sin Goh
EPI_ISL_2816202, EPI_ISL_2816206, EPI_ISL_2816211	Sklifosovsky Research Institute of Emergency Medicine	WHO National Influenza Centre Russian Federation	Andrey Komissarov; Artem Fadeev; Daria Danilenko; Dmitry Lioznov; Elena Nabieva; Georgii Bazykin; Kirill Varchenko; Ksenia Safina; Kseniya Komissarova; Maria Pisareva; Mikhail Bakaev; Nikita Yolshin; Oula Mansour; Tamila Musaeva; Veronika Eder
EPI_ISL_2761446, EPI_ISL_2762059	Sonic - Labor Staber Nürnberg	Robert Koch Institute	
EPI_ISL_2260735, EPI_ISL_2260774, EPI_ISL_2388738, EPI_ISL_2762002	Sonic - MVZ Medizinisches Labor Bremen GmbH	Robert Koch Institute	
EPI_ISL_1904449, EPI_ISL_1904454, EPI_ISL_1904457, EPI_ISL_1904459, EPI_ISL_1904460, EPI_ISL_1904462, EPI_ISL_1904463, EPI_ISL_1904464, EPI_ISL_1904465, EPI_ISL_1904466, EPI_ISL_1904468, EPI_ISL_1904469, EPI_ISL_1904470, EPI_ISL_2405109, EPI_ISL_2405110, EPI_ISL_2405113, EPI_ISL_2405117, EPI_ISL_2462434, EPI_ISL_2650455, EPI_ISL_2650460, EPI_ISL_2828017			
see above	South Eastern Area Laboratory Services (SEALS)	NSW Health Pathology - Institute of Clinical Pathology and Medical Research; Westmead Hospital; University of Sydney	CIDM-PH et al.
EPI_ISL_2833681	Spital Limmattal	Institute of Medical Virology	Alexandra Trkola; Annette Audigé; Cyril Shah; Gabriela Ziltener; Guido Bloemberg; Jon Huder; Jürg Böni; Kevin Steiner; Maria Grünberg; Maryam Zaheri; Michael Huber; Riccarda Capaul; Stefan Schmutz; Verena Kufner
EPI_ISL_2833683, EPI_ISL_2833684, EPI_ISL_2858697	Spital Männedorf AG	Institute of Medical Virology	Alexandra Trkola; Annette Audigé; Cyril Shah; Gabriela Ziltener; Guido Bloemberg; Jon Huder; Jürg Böni; Kevin Steiner; Maria Grünberg; Maryam Zaheri; Michael Huber; Riccarda Capaul; Stefan Schmutz; Verena Kufner
EPI_ISL_2510859	Spital MŠnnedorf AG	Institute of Medical Virology	Alexandra Trkola; Annette AudigŽ; Catharine Aquino; Cyril Shah; Daniel Ehrsam; Gabriela Ziltener; Guido Bloemberg; Hubert Rehrauer; Isabel StŸrmer; Joel Wirz; Jon Huder; JŸrg Bšni; Kevin Steiner; Maria GrŸnberg; Maryam Zaheri; Michael Huber; Riccarda Capaul; Stefan Schmutz; Verena Kufner; Weihong Qi
EPI_ISL_2362528, EPI_ISL_2362530, EPI_ISL_2834141, EPI_ISL_2861376	St James's Hospital, Dublin 8, Ireland.	ELDA biotech	Brendan Crowley; Deirdre M. Broderick; Peter R. Flanagan
EPI_ISL_2876015, EPI_ISL_2876016, EPI_ISL_2876025, EPI_ISL_2876041	St Vincent's Pathology (SydPath)	NSW Health Pathology - Institute of Clinical Pathology and Medical Research; Westmead Hospital; University of Sydney	CIDM-PH et al.
EPI_ISL_2360241, EPI_ISL_2833694	Stadtspital Triemli	Institute of Medical Virology	Alexandra Trkola; Annette Audigé; Cyril Shah; Gabriela Ziltener; Guido Bloemberg; Jon Huder; Jürg Böni; Kevin Steiner; Maria Grünberg; Maryam Zaheri; Michael Huber; Riccarda Capaul; Stefan Schmutz; Verena Kufner
EPI_ISL_2377955, EPI_ISL_2800635	State Testing Facility	Altius Institute for Biomedical Research	Alex Isner; Alex Nguyen; Amanda Gale; Audra Johnson; Clem Green; Daniel Bates; Eric Thorland; Jacob Rodriguez; Jean Robinston; Jemma Nelson; Jessica Kunder; John Stamatoyannopoulos; Joshua Richards; Julia Wald; Kneshay Harper; Lauren Mitchell; Mark Frerker; Matt Hartman; Michael Buckley; Muhammad Halimun; Rebecca Bruders; Sadie Patraw; Sofia Olsson; Tobias Ragoczy
EPI_ISL_1914584	State Virus Research and Diagnostic Laboratory (VRDL), AIIMS Raipur	State Virus Research and Diagnostic Laboratory (VRDL), AIIMS Raipur	Anudita Bhargava; Kuldeep Sharma; Priyanka Singh; Pushpendra Singh; Sanjay Singh Negi; Somya Sharma
EPI_ISL_2684247, EPI_ISL_2684307, EPI_ISL_2684814, EPI_ISL_2684859, EPI_ISL_2685126, EPI_ISL_2685164, EPI_ISL_2685428, EPI_ISL_2685473, EPI_ISL_2685521, EPI_ISL_2685602, EPI_ISL_2685763			
see above	Statens Serum Institut Bioinformatics and Microbial Genomics	Statens Serum Institut Bioinformatics and Microbial Genomics	Danish Covid-19 Genome Consortium
EPI_ISL_2678466	Städtisches Klinikum Dresden Institut für Klinische Chemie und Laboratoriumsmedizin	Robert Koch Institute	
EPI_ISL_2812706	Sultan Haji Ahmad Shah Hospital	Institute for Medical Research, Infectious Disease Research Centre, National Institutes of Health, Ministry of Health Malaysia	Azizan MA; Kamel K; Mohd Zawawi Z; Ramly N; Robert F; Suppiah J; Thayan R
EPI_ISL_2091024	Sultanah Aminah Hospital, Johor Bahru	Institute for Medical Research, Infectious Disease Research Centre, National Institutes of Health, Ministry of Health Malaysia	Azizan MA; Kamel K; Mohd Zawawi Z; Ramly N; Robert F; Suppiah J; Thayan R
EPI_ISL_1972356, EPI_ISL_2091020	Sungai Buloh Hospital	Institute for Medical Research, Infectious Disease Research Centre, National Institutes of Health, Ministry of Health Malaysia	Azizan MA; Kamel K; Mohd Zawawi Z; Ramly N; Robert F; Suppiah J; Thayan R
EPI_ISL_2510687, EPI_ISL_2570808, EPI_ISL_2570809, EPI_ISL_2570811, EPI_ISL_2617435, EPI_ISL_2617436, EPI_ISL_2617437, EPI_ISL_2693670			
see above	Surabaya Health Office	Institute of Tropical Disease, Universitas Airlangga	Aldise M Nastri; Febria Rachmanita; Gatot Soegiarto; Herlin Ferliana; Jezzy R Dewantari; Kazufumi Shimizu; Krisnoadi Rahardjo; Laksmi Wulandari; Maria I Lusida; Resti Yudhawati; Rima R Prasetya; Soetjipto; Yasuko Mori
EPI_ISL_1808683, EPI_ISL_1808686, EPI_ISL_1899297, EPI_ISL_2032727, EPI_ISL_2033405, EPI_ISL_2033751, EPI_ISL_2034582, EPI_ISL_2034589, EPI_ISL_2205865, EPI_ISL_2209223, EPI_ISL_2257706, EPI_ISL_2414357, EPI_ISL_2414459, EPI_ISL_2414464, EPI_ISL_2414793, EPI_ISL_2415198, EPI_ISL_2415482, EPI_ISL_2415484, EPI_ISL_2415611, EPI_ISL_2416022, EPI_ISL_2416025, EPI_ISL_2418112, EPI_ISL_2418223, EPI_ISL_2418250, EPI_ISL_2418275, EPI_ISL_2418290, EPI_ISL_2418297, EPI_ISL_2418305, EPI_ISL_2418315, EPI_ISL_2418320, EPI_ISL_2418322, EPI_ISL_2418328, EPI_ISL_2418647, EPI_ISL_2419791, EPI_ISL_2584380, EPI_ISL_2584419, EPI_ISL_2584480, EPI_ISL_2613548, EPI_ISL_2613550, EPI_ISL_2613551, EPI_ISL_2615371, EPI_ISL_2618839, EPI_ISL_2618844, EPI_ISL_2618847, EPI_ISL_2618883, EPI_ISL_2618891, EPI_ISL_2618893, EPI_ISL_2618971, EPI_ISL_2618972, EPI_ISL_2618973, EPI_ISL_2618980, EPI_ISL_2618994, EPI_ISL_2619174, EPI_ISL_2619176, EPI_ISL_2619177, EPI_ISL_2619178, EPI_ISL_2619266, EPI_ISL_2619267, EPI_ISL_2619550, EPI_ISL_2619555, EPI_ISL_2619559, EPI_ISL_2619571, EPI_ISL_2619588, EPI_ISL_2619595, EPI_ISL_2619619, EPI_ISL_2619625, EPI_ISL_2619626, EPI_ISL_2619630, EPI_ISL_2619631, EPI_ISL_2619632, EPI_ISL_2619644, EPI_ISL_2619703, EPI_ISL_2792631, EPI_ISL_2792665, EPI_ISL_2792676, EPI_ISL_2792861, EPI_ISL_2792888, EPI_ISL_2793073, EPI_ISL_2793127, EPI_ISL_2793135, EPI_ISL_2793136, EPI_ISL_2793157, EPI_ISL_2793203, EPI_ISL_2793275, EPI_ISL_2793280, EPI_ISL_2793433, EPI_ISL_2793440, EPI_ISL_2793443, EPI_ISL_2793445, EPI_ISL_2793446, EPI_ISL_2793449, EPI_ISL_2793451, EPI_ISL_2793452, EPI_ISL_2793454, EPI_ISL_2793457, EPI_ISL_2793469, EPI_ISL_2793479, EPI_ISL_2793485, EPI_ISL_2793496, EPI_ISL_2793499, EPI_ISL_2793544, EPI_ISL_2793624, EPI_ISL_2793709, EPI_ISL_2793742, EPI_ISL_2793807, EPI_ISL_2793875, EPI_ISL_2793887, EPI_ISL_2793892, EPI_ISL_2793986, EPI_ISL_2793992, EPI_ISL_2794006, EPI_ISL_2794008, EPI_ISL_2794087, EPI_ISL_2794267, EPI_ISL_2794959, EPI_ISL_2795205, EPI_ISL_2795215, EPI_ISL_2795249, EPI_ISL_2795261, EPI_ISL_2795359, EPI_ISL_2795364, EPI_ISL_2795370, EPI_ISL_2795399, EPI_ISL_2795400, EPI_ISL_2795405, EPI_ISL_2795409, EPI_ISL_2795440, EPI_ISL_2795447, EPI_ISL_2795459, EPI_ISL_2795512, EPI_ISL_2795581, EPI_ISL_2795601, EPI_ISL_2820812, EPI_ISL_2888082, EPI_ISL_2888102, EPI_ISL_2888141, EPI_ISL_2888163, EPI_ISL_2888175, EPI_ISL_2888176, EPI_ISL_2888179, EPI_ISL_2888185, EPI_ISL_2888197, EPI_ISL_2888509, EPI_ISL_2888585, EPI_ISL_2888722, EPI_ISL_2888773, EPI_ISL_2888778			
see above	Swedish national genomic surveillance program of SARS-CoV-2	The Public Health Agency of Sweden	Alma Brolund; Maria Lind Karlberg; Maximilian Riess; Swedish national genomic surveillance program of SARS-CoV-2
EPI_ISL_1969243	Swissbel Hotel Airport	National Institute of Health Research and Development	Arie Ardiansyah Nugraha; Hana Apsari Pawestri; Hartanti Dian Ikawati; Kartika Dewi Puspa; Krisna Pangesti; Nelly Puspandari; Subangkit; Triyani Soekarso; Vivi Setiawaty
EPI_ISL_2876044	Sydney South West Pathology Service (SSWPS) - Liverpool Hospital - NSW Health Pathology	NSW Health Pathology - Institute of Clinical Pathology and Medical Research; Westmead Hospital; University of Sydney	CIDM-PH et al.
EPI_ISL_2405111, EPI_ISL_2405112, EPI_ISL_2462421, EPI_ISL_2650469	Sydney South West Pathology Service (SSWPS) - Royal Prince Alfred Hospital - NSW Health Pathology	NSW Health Pathology - Institute of Clinical Pathology and Medical Research; Westmead Hospital; University of Sydney	CIDM-PH et al.
EPI_ISL_2248985, EPI_ISL_2249005, EPI_ISL_2249015	Synlab	Instituto Nacional de Saude (INSA)	Borges et al
EPI_ISL_2267288	Synlab MVZ Augsburg	Robert Koch Institute	
EPI_ISL_2611028, EPI_ISL_2768092	Synlab Suisse SA	Clinical Bacteriology	Adrian Egli; Alfredo Mari; Fanny Wegner; Hans Hirsch; Helena MB Seth-Smith; Julia Bielicki; Karoline Leuzinger; Manuel Battegay; Tim Roloff

EPI_ISL_2423549, EPI_ISL_2443304	TXDSHS	TXDSHS	Anita Pokharel; Bonnie Oh; Chun Wang; Grace Kubin; Jenny Zhang; Karen Bobier; Lorraine Rodriguez; Maliha Rahman; Mayela Pedrueza; Myong Koag; Rachel Lee; Rashmi Tuladhar
EPI_ISL_2363996, EPI_ISL_2363998, EPI_ISL_2364003, EPI_ISL_2364004, EPI_ISL_2644978, EPI_ISL_2644988	TYKS, Kliininen mikrobiologia	Expert Microbiology, National Institute for Health and Welfare	Carita Savolainen-Kopra; Erika Lindh; Haider al-Hello; Jani Halkilahti; Kirsi Liitsola; Niina Ikonen; Olli Vapalahti; Pekka Ellonen; Phuoc Truong; Päivi Laurila; Ravi Kant; Sari Hannula; Soile Blomqvist; Teemu Smura
EPI_ISL_2081386, EPI_ISL_2800908, EPI_ISL_2800910, EPI_ISL_2800911, EPI_ISL_2800912, EPI_ISL_2833815, EPI_ISL_2833913			
see above	Tampa General Hospital Esoteric Lab	Tampa General Hospital Esoteric Research & Development Lab	Amorce Lima; Deanna Becker; Dominic Uy; Elaine Vendrone; Grant Vestal; Jon Faughn; Jorge Mecias-Frias; Suzane Silbert; Vicki Healer
EPI_ISL_2501034, EPI_ISL_2501035, EPI_ISL_2801904, EPI_ISL_2801905, EPI_ISL_2801906	Tboung Khmum Rapid Response Team	Virology Unit, Institut Pasteur du Cambodge	Cecile Troupin; Chau Darapheak; Chin Savuth; Erik A Karlsson; Jurre Y Siegers; Kraing Sidonn; Leakhena Pum; Ly Sovann; Veasna Duong; Yi Sengdoeurn
EPI_ISL_1838134	The National Centre for Cell Science	CSIR-Centre for Cellular and Molecular Biology-INSACOG	Ajay Pillai; Amareshwar Vodapalli; Ara Sreenivas; Archana Bharadwaj Siva; B Himasri; Blessy B John; Dhiraj Paul; Divya Tej Sowpati; INSACOG Consortium team; Karthik Bharadwaj Tallapaka; Lamuk Zaveri; Manoj Kumar Bhat; Mitali Inamdar; Mohak P Gujare; Onkar Kulkarni; Payel Mukherjee; Rakesh K Mishra; Sharath Chandra Thota; Shivang P. Bhanushali; Shreekant Verma; Sofia Banu; Sonal Manik Chavan; Tulasi Nagabandi; Valli Nagalakshmi Undamatla; Viswagithe S L; Yogesh Shouche
EPI_ISL_2551582	Tochigi Prefectural Institute of Environment and Health	Genome Analysis Center, Kamma Memorial Hospital	Fuminori Mizukoshi; Hanako Yazawa; Hiroshi Kamma; Jun Ishii; Kaori Watanabe; Michiaki Masuda; Satoko Soma; Takuya Yazawa; Yoshiko Hitomi
EPI_ISL_2562788	TriCore Reference Laboratories	Center for Global Health, University of New Mexico Health Sciences Center	Cecilia Thompson; Darrell Dinwiddie; Daryl Domman; Karissa Culbreath; Kendra Pesko; Kurt Schwalm; Valerie Morley
EPI_ISL_2649992	Tuanku Ampuan Najihah Hospital, Kuala Pilah	Institute for Medical Research, Infectious Disease Research Centre, National Institutes of Health, Ministry of Health Malaysia	Azizan MA; Kamel K; Mohd Zawawi Z; Ramly N; Robert F; Suppiah J; Thayan R
EPI_ISL_2878097, EPI_ISL_2878104	UAB Diagnostikos laboratorija	National Public Health Surveillance Laboratory	Ana Steponkiene; Danas Baksa; Jelena Razmuk; Lukas Vasionis; Lukas Zemaitis; Migle Gabrielaite; Svajune Muralyte
EPI_ISL_2694582, EPI_ISL_2694583, EPI_ISL_2694584	UAB InMedica	National Public Health Surveillance Laboratory	Ana Steponkiene; Danas Baksa; Jelena Razmuk; Lukas Vasionis; Lukas Zemaitis; Migle Gabrielaite; Svajune Muralyte
EPI_ISL_2644785, EPI_ISL_2878075	UAB Medicina practica laboratorija	Institute of Biotechnology, Life Sciences Center, Vilnius University	Albertas Timinskas; Alma Gedvilaite; Danguole Ziogiene; Emilija Vasiliunaite; Milda Norkiene
EPI_ISL_2448883	UCK	1. National Institute of Public Health - National Institute of Hygiene, Warsaw, Poland 2. Biobank Lab, University of Lodz 3. Laboratory of Respiratory Viruses, Teaching and Clinical Center of the Medical University of Lodz	Dominik Strapagiel; Izabela Dróżdż; Jakub Lach; Katarzyna Zacharczuk; Klaudyna Królikowska; Maciej Borowiec; Magdalena Nowakowska; Magdalena Traczyk-Borszyńska; Marcin Słomka; Marta Sobalska-Kwapis; Małgorzata Sadkowska-Todys; Tomasz Płoszaj; Tomasz Wołkowicz
EPI_ISL_2597819	UCK	1. Tricity SARS-CoV-2 sequencing consortium: University of Gdansk, Medical University of Gdansk, Vaxican Ltd., Invicta Ltd. 2. National Institute of Public Health - National Institute of Hygiene, Warsaw, Poland	Celina Cybulska; Karolina Gackowska; Katarzyna Groth; Katarzyna Zacharczuk; Krystyna Bienkowska Szewczyk; Lukasz Rabalski; Maciej Grzybek; Maciej Kosinski; Magdalena Nowakowska; Marcin Lubocki; Małgorzata Sadkowska-Todys; Tomasz Wołkowicz
EPI_ISL_2695124, EPI_ISL_2695125, EPI_ISL_2695130, EPI_ISL_2695132, EPI_ISL_2695134, EPI_ISL_2695136, EPI_ISL_2695144, EPI_ISL_2895164			
see above	ULS Castelo Branco	Instituto Nacional de Saude (INSA)	Borges et al
EPI_ISL_2340230	ULS Litoral Alentejano	Instituto Nacional de Saude (INSA)	Borges et al
EPI_ISL_2249202	ULS Litoral Alentejano	Instituto Nacional de Saude (INSA) and Centro de Investigaçao em Biodiversidade e Recursos Geneticos (CIBIO), Universidade do Porto	Borges et al
EPI_ISL_2249115, EPI_ISL_2695165, EPI_ISL_2695170, EPI_ISL_2695172, EPI_ISL_2695173, EPI_ISL_2695174, EPI_ISL_2695175, EPI_ISL_2695176, EPI_ISL_2695204, EPI_ISL_2695205, EPI_ISL_2695206, EPI_ISL_2695210, EPI_ISL_2695217, EPI_ISL_2695218			
see above	ULS Litoral Alentejano	Instituto Nacional de Saude (INSA) and Institute of Biomedicine (iBiMed), Universidade de Aveiro	Borges et al
EPI_ISL_2376292, EPI_ISL_2376294, EPI_ISL_2376405, EPI_ISL_2376406, EPI_ISL_2376408	ULSS 2 Marca Trevigiana	Istituto Zooprofilattico Sperimentale delle Venezie	Adelaide Milani; Alessia Schivo; Alice Fusaro; Ambra Pastori; Annalisa Salviato; Antonia Ricci; Calogero Terregino; Edoardo Giussani; Elisa Palumbo; Erika Giorgia Quaranta; Isabella Monne; Luca Tassoni
EPI_ISL_2376289, EPI_ISL_2376290, EPI_ISL_2376291, EPI_ISL_2376379, EPI_ISL_2376380, EPI_ISL_2392108	ULSS 3 Venezia	Istituto Zooprofilattico Sperimentale delle Venezie	Adelaide Milani; Alessia Schivo; Alice Fusaro; Ambra Pastori; Annalisa Salviato; Antonia Ricci; Calogero Terregino; Edoardo Giussani; Elisa Palumbo; Erika Giorgia Quaranta; Isabella Monne; Luca Tassoni
EPI_ISL_2376285, EPI_ISL_2376288	ULSS 4 Veneto Orientale	Istituto Zooprofilattico Sperimentale delle Venezie	Adelaide Milani; Alessia Schivo; Alice Fusaro; Ambra Pastori; Annalisa Salviato; Antonia Ricci; Calogero Terregino; Edoardo Giussani; Elisa Palumbo; Erika Giorgia Quaranta; Isabella Monne; Luca Tassoni
EPI_ISL_2448534	ULSS 7 Pedemontana - Distretto 2	Istituto Zooprofilattico Sperimentale delle Venezie	Adelaide Milani; Alessia Schivo; Alice Fusaro; Ambra Pastori; Annalisa Salviato; Antonia Ricci; Calogero Terregino; Edoardo Giussani; Elisa Palumbo; Erika Giorgia Quaranta; Isabella Monne; Luca Tassoni
EPI_ISL_2376296, EPI_ISL_2376297, EPI_ISL_2448430, EPI_ISL_2448618, EPI_ISL_2448619, EPI_ISL_2448620	ULSS 8 Berica	Istituto Zooprofilattico Sperimentale delle Venezie	Adelaide Milani; Alessia Schivo; Alice Fusaro; Ambra Pastori; Annalisa Salviato; Antonia Ricci; Calogero Terregino; Edoardo Giussani; Elisa Palumbo; Erika Giorgia Quaranta; Isabella Monne; Luca Tassoni
EPI_ISL_2543732	UM im. Karola Marcinkowskiego w Poznaniu Laboratorium UCA_Covid-19	1. National Institute of Public Health - National Institute of Hygiene, Warsaw, Poland 2. Biobank Lab, University of Lodz 3. Laboratory of Respiratory Viruses, Teaching and Clinical Center of the Medical University of Lodz	Dominik Strapagiel; Izabela Dróżdż; Jakub Lach; Katarzyna Zacharczuk; Klaudyna Królikowska; Maciej Borowiec; Magdalena Nowakowska; Magdalena Traczyk-Borszyńska; Marcin Słomka; Marta Sobalska-Kwapis; Małgorzata Sadkowska-Todys; Tomasz Płoszaj; Tomasz Wołkowicz
EPI_ISL_2726793	UM im. Karola Marcinkowskiego w Poznaniu Laboratorium UCA_Covid-19 Centrum Biologii Medycznej	1. National Institute of Public Health - National Institute of Hygiene, Warsaw, Poland 2. Biobank Lab, University of Lodz 3. Laboratory of Respiratory Viruses, Teaching and Clinical Center of the Medical University of Lodz	Dominik Strapagiel; Izabela Dróżdż; Jakub Lach; Katarzyna Zacharczuk; Klaudyna Królikowska; Maciej Borowiec; Magdalena Nowakowska; Magdalena Traczyk-Borszyńska; Marcin Słomka; Marta Sobalska-Kwapis; Małgorzata Sadkowska-Todys; Tomasz Płoszaj; Tomasz Wołkowicz
EPI_ISL_1993936, EPI_ISL_2099526, EPI_ISL_2132653	UMC Groningen, Clinical Virology, Department of Medical Microbiology and Infection Prevention	UMC Groningen, Clinical Virology, Department of Medical Microbiology and Infection Prevention	Alexander Friedrich; Coretta Van Leer-Buter; Erley Lizarazo-Forero; Hubert Niesters; Lilli Gard; Marjolein Knoester; Monika Fliss; Sigrid Rosema; Xuewei Zhou
EPI_ISL_2658352, EPI_ISL_2886117, EPI_ISL_2886121, EPI_ISL_2887964	UMC Utrecht	UMC Utrecht	Annemarie Wensing; Anniek Tanja; Pascal Mantiri; Rob Schuurman
EPI_ISL_2249037, EPI_ISL_2628687, EPI_ISL_2628850, EPI_ISL_2628923, EPI_ISL_2628927, EPI_ISL_2628964, EPI_ISL_2645947, EPI_ISL_2645961, EPI_ISL_2645965, EPI_ISL_2645979, EPI_ISL_2646008, EPI_ISL_2646041, EPI_ISL_2646057, EPI_ISL_2646096, EPI_ISL_2796353, EPI_ISL_2810493, EPI_ISL_2810644, EPI_ISL_2810648, EPI_ISL_2810658, EPI_ISL_2810682, EPI_ISL_2810744, EPI_ISL_2895501, EPI_ISL_2895619, EPI_ISL_2895623, EPI_ISL_2895628			
see above	UNILABS	Instituto Nacional de Saude (INSA)	Borges et al
EPI_ISL_2810382	UNILABS	Instituto Nacional de Saude (INSA) and BioSystems & Integrative Sciences Institute (BioISI) Genomics Unit, FCUL	Borges et al
EPI_ISL_2796012, EPI_ISL_2796021, EPI_ISL_2796032, EPI_ISL_2796038, EPI_ISL_2796154, EPI_ISL_2796174	UNILABS	Instituto Nacional de Saude (INSA) and Instituto Gulbenkian de Ciencia (IGC)	Borges et al
EPI_ISL_2854707	UPTD LABKESDA Kota Samarinda	National Institute of Health Research and Development	Arie Ardiansyah Nugraha; Hana Apsari Pawestri; Hartanti Dian Ikawati; Kartika Dewi Puspa; Krisna Pangesti; Nelly Puspandari; Subangkit; Triyani Soekarso; Vivi Setiawaty
EPI_ISL_2715293	US Air Force School of Aerospace Medicine	US Air Force School of Aerospace Medicine	Amanda Javorina; Anthony Fries; Carol Garrett; Clarise Starr; Elizabeth Macias; Jennifer Meyer; Sarah Purves; William Gruner
EPI_ISL_1620980, EPI_ISL_1715512, EPI_ISL_1826132, EPI_ISL_1850287, EPI_ISL_2002689, EPI_ISL_2031536, EPI_ISL_2082516, EPI_ISL_2618216, EPI_ISL_2618292, EPI_ISL_2802933, EPI_ISL_2803144, EPI_ISL_2803159			

see above	UW Virology Lab	UW Virology Lab	Alexander Greninger; Hong Xie; Keith R Jerome; Lasata Shrestha; Meei-Li Huang; Michelle Lin; Nathan Breit; Noah R. Baker; Patrick Mathias; Pavitra Roychoudhury; Ricardo Perez; Robert J. Livingston; Saraswathi Sathees; Sean Ellis; Shah Mohamed Bakhash; Tien V. Nguyen
EPI_ISL_2681130, EPI_ISL_2681218, EPI_ISL_2801578, EPI_ISL_2801595	Unidad de Investigación Médica de Yucatán (UIMY)	Instituto de Biotecnología de la UNAM	; Alejandra García-Gasca; Alejandra Hernández-Terán; Alejandro Sánchez-Flores; Alfredo Herrera-Estrella; Alicia Ocaña-Mondragón; Andreu Comas-García; Angel Gustavo Salas-Lais; Antonio Loza Román; Bernardo Martínez-Miguel; Blanca Taboada; Brenda Irasema Maldonado-Meza; Bruno Gómez-Gil; Carla Ivón Herrera-Najera; Carlos F. Arias; Celia Boukadida; Clara Esperanza Santacruz-Tinoco; Concepción Grajales-Muñiz; Consorcio Mexicano de Vigilancia Genómica (CoViGen-Mex). Authors (in alphabetical order): Julio Elias Alvarado-Yaah; Cristóbal Cháidez-Quiróz; Célida Duque Molina; Célida Martínez- Rodríguez; Daniel Fregoso-Rueda; Daniel Lira Morales; Eduardo Becerril-Vargas; Fernando Fontove-Herrera; Fidencio Mejía-Nepomuceno; Francisco Pulido; Gloria Elena Espinosa-Ayala; Gloria María Molina-Salinas; Gloria Vazquez; Hector Esteban Paz-Juárez; Hector Montoya-Fuentes; Helen Haydee Fernanda Ramírez-Plascencia; Irvin González-López; Jean Pierre González; Jesús Hernández; Joel Armando Vázquez-Pérez.; Jorge Salas-Hernández; José Antonio Enciso-Moreno; José Arturo Martínez-Orozco; José Esteban Muñoz-Medina; José de Jesús Nuñez-Contreras; Juan Bautista Chale-Dzul; Julissa Enciso-Ibarra; Luis Alberto Ochoa-Carrera; Margarita Matías-Florentino; Mario Mújica-Sánchez; Marissa Perez-Garcia; María Guadalupe Santiago-Mauricio; María Guadalupe de Jesús Mireles-Rivera; Nelly Sélem-Mojica; Pavel Isa; Ricardo Ciria Merce; Ricardo Grande; Rosa María Gutiérrez Rios; Santiago Ávila-Ríos; Selene Zárate; Susana Lopez; Verónica Mata-Haro; Victor Eduardo García-Arias; Víctor Hugo Borja-Aburto
EPI_ISL_2837266, EPI_ISL_2837299	Unidade de apoio ao diagnostico da COVID - UNADIG	Bioinformatics Laboratory / LNCC	Alessandra P Lamarca; Alexandra L Gerber; Amilcar Tanuri; Ana Paula de C Guimaraes; Ana Tereza R Vasconcelos; Andrea Cony Cavalcanti; Caio Luiz Pereira Ribeiro; Cassia Alves; Cintia Policarpo; Claudia Maria Braga de Mello; Cristiane Gomes da Silva; Diana Mariani; Douglas Terra Machado; Flavio Dias da Silva; Gleidson da Silva de Oliveira; Leandro Magalhaes de Souza; Liliane Cavalcante; Luiz G P de Almeida; Marcio Henrique de Oliveira Garcia; Mario Sergio Ribeiro; Ronaldo da Silva F Jr; Silvia Carvalho
EPI_ISL_2678452	Uniklinikum Carl Gustav Carus an der TU Dresden; Institut für Virologie	Robert Koch Institute	
EPI_ISL_2832815, EPI_ISL_2833593, EPI_ISL_2833594, EPI_ISL_2833598, EPI_ISL_2840167	Unilabs	Karolinska University Hospital	Annelie Bjerkner; Isak Sylvin; Jan Albert; Karolina Ininbergs; Lina Guerra Blomqvist; Lynda Eneh; Martin Ekman; Martina Wahlund; Robert Dyrdak; Sandra Broddesson; Tanja Normark; Tobias Allander; Valtteri Wirta; Zhibing Yun
EPI_ISL_2483123, EPI_ISL_2820867, EPI_ISL_2820880	Unilabs Laboratory Medicine	Norwegian Institute of Public Health, Department of Virology	Atiya R Ali; Debech Nadia; Engebretsen Serina Beate; Garcia Llorente Ignacio; Hilde Elshaug; Hilde Vollan; Jon Bråte; Kamilla Heddeland Instefjord; Karoline Bragstad; Kathrine Stene-Johansen; Line Victoria Moen; Marie Paulsen Madsen; Olav Hungnes; Pedersen Benedikte Nevjen; Rasmus Riis Kopperud
EPI_ISL_2622828, EPI_ISL_2816496	University College London, Great Ormond Street Hospital for Children NHS Foundation Trust, Imperial College Healthcare NHS Trust	COVID-19 Genomics UK (COG-UK) Consortium	Alison Holmes; Charlotte Williams; Helena Tutill; Jacqueline Findlay; James Price; Judith Breuer; Julianne Brown; Kathryn Harris; Leysa Forrest; Marius Cotic; Mark Kristiansen; Nadua Bayzid; Paola Niola; Paola Resende Silva; Patricia Dyal; Paul Randell; Rachel Williams; Samuel Weeks; Sergi Castellano; Sunando Roy; Tony Brooks; Yasmin Panchbhaya
EPI_ISL_2841707	University Hospital of Northern Norway, Department for Microbiology and Infectious Disease Control	Norwegian Institute of Public Health, Department of Virology	Atiya R Ali; Debech Nadia; Engebretsen Serina Beate; Garcia Llorente Ignacio; Hilde Elshaug; Hilde Vollan; Jon Bråte; Kamilla Heddeland Instefjord; Karoline Bragstad; Kathrine Stene-Johansen; Line Victoria Moen; Marie Paulsen Madsen; Olav Hungnes; Pedersen Benedikte Nevjen; Rasmus Riis Kopperud
EPI_ISL_1811202, EPI_ISL_1963298, EPI_ISL_1963299, EPI_ISL_1963300, EPI_ISL_2104479, EPI_ISL_2104494, EPI_ISL_2213568, EPI_ISL_2213571, EPI_ISL_2213579, EPI_ISL_2213582, EPI_ISL_2213584, EPI_ISL_2343033, EPI_ISL_2343068, EPI_ISL_2343125, EPI_ISL_2490656, EPI_ISL_2490657, EPI_ISL_2681456, EPI_ISL_2681461, EPI_ISL_2790082, EPI_ISL_2790084, EPI_ISL_2790088, EPI_ISL_2790092, EPI_ISL_2790094, EPI_ISL_2885473, EPI_ISL_2885480, EPI_ISL_2885497, EPI_ISL_2885498, EPI_ISL_2885502, EPI_ISL_2885504			
see above	University Hospitals of Geneva, Laboratory of Virology	HUG, Laboratory of Virology and the Health2030 Genome Center	Ana Rita Goncalves; Deborah Penet; Emmanouil Dermitzakis; Henri Pegeot; Ioannis Xenarios; Keith Harshman; Laurent Kaiser; Lorenzo Cerutti; Melyssa Elies; Samuel Cordey
EPI_ISL_1922125	University of Bari Biomedical Sciences and Human Oncology	University of Bari Biomedical Sciences and Human Oncology	Accogli M.; Chironna M.; Loconsole D.; Sallustio A.
EPI_ISL_2692117	University of Illinois at Chicago	RIPHL at Rush University Medical Center	Felix Araujo Perez; Julie Bentea; Kevin Kunstman; Laura Furtado; Marieta Hyde; Max Kolton; Omar Perez; Stefan Green
EPI_ISL_2003438, EPI_ISL_2500011	University of Liège COVID-19 testing center	GIGA Medical Genomics	Bouchra Boujemla; Cécile Meex; Fabrice Bureau; Keith Durkin; Laurent Gillet; Maria Artesi; Marie-Pierre Hayette; Nathalie Renotte; Pierrette Melin; Raphaël Boreux; Sébastien Bontems; Vincent Bours; Wouter Coppieters
EPI_ISL_2803661, EPI_ISL_2803662, EPI_ISL_2803663, EPI_ISL_2803665, EPI_ISL_2803666, EPI_ISL_2803668, EPI_ISL_2803669, EPI_ISL_2803670, EPI_ISL_2803671, EPI_ISL_2803672, EPI_ISL_2803673, EPI_ISL_2803674, EPI_ISL_2803675, EPI_ISL_2803676, EPI_ISL_2803678, EPI_ISL_2803679, EPI_ISL_2803680, EPI_ISL_2803681, EPI_ISL_2803682, EPI_ISL_2803684, EPI_ISL_2803685, EPI_ISL_2803686, EPI_ISL_2803687, EPI_ISL_2803688, EPI_ISL_2803689, EPI_ISL_2803690, EPI_ISL_2803691, EPI_ISL_2803692, EPI_ISL_2803693, EPI_ISL_2803694, EPI_ISL_2803695, EPI_ISL_2803696, EPI_ISL_2803697, EPI_ISL_2803698, EPI_ISL_2803699, EPI_ISL_2803700, EPI_ISL_2803701, EPI_ISL_2803702, EPI_ISL_2803703, EPI_ISL_2803704, EPI_ISL_2803705, EPI_ISL_2803706, EPI_ISL_2803707, EPI_ISL_2803708, EPI_ISL_2803709, EPI_ISL_2803710, EPI_ISL_2803711, EPI_ISL_2803712, EPI_ISL_2803713, EPI_ISL_2803714, EPI_ISL_2803715, EPI_ISL_2803716, EPI_ISL_2803717, EPI_ISL_2803718, EPI_ISL_2803719, EPI_ISL_2803720, EPI_ISL_2803721, EPI_ISL_2803722, EPI_ISL_2803723, EPI_ISL_2803725, EPI_ISL_2803726, EPI_ISL_2803727, EPI_ISL_2803728, EPI_ISL_2803729, EPI_ISL_2803730, EPI_ISL_2803731, EPI_ISL_2803732, EPI_ISL_2803733, EPI_ISL_2803734, EPI_ISL_2803735, EPI_ISL_2803736, EPI_ISL_2803738, EPI_ISL_2803739, EPI_ISL_2803740, EPI_ISL_2803741, EPI_ISL_2803742, EPI_ISL_2803743, EPI_ISL_2803744			
see above	University of Zambia, School of Veterinary Medicine	UNZAVET and PATH	Daniel Bridges; Mulenga Mwenda-Chimfwembe; Ngonda Saasa; ZNPHI and ZGSC
EPI_ISL_2558055	University of the Philippines National Institutes of Health (UP NIH)	Philippine Genome Center	Alethea R. de Guzman; Anna Ong-Lim; Arianne A. Zamora; Asia Louisa U. Chong; Benedict A. Maralit; Candice Francheska B. Tambaoan; Carlo M. Lapid; Celia Carlos; Devon Ray Pacial; Edsel Maurice Salvaña; El King D. Morado; Elcid Aaron R. Pangilinan; Eva Maria Cutiongco-de la Paz; Francis A. Tablizo; Irish Coleen A. Asin; Jaime C. Montoya; Jan Michael C. Yap; Jo-Hannah S. Llames; John Q. Wong; Joshua Gregor A. Dizon; Juan Antonio R. Magalang; Karol Sophia Agape R. Padilla; Kenneth M. Kim; Kris P. Punayan; Marc Edsel C. Ayes; Maria Rosario Singh-Vergeire and Cynthia P. Saloma; Maria Sofia L. Yangzon; Marissa Alejandria; Razel Nikka M. Hao; Renato Jacinto Q. Mantaring; Rianna Patricia S. Cruz; Sheila Mae M. Araiza
EPI_ISL_2600538, EPI_ISL_2686067	Università degli Studi di Perugia	Istituto Zooprofilattico Sperimentale dell'Abruzzo e Molise "G. Caporale"	Ancora M; Biagetti M; Calistri P; Camilloni B; Cammà C; Curini V; Delli Compagni E; Di Domenico M; Di Pasquale A; Giammarioli M; Lorusso A; Mangone I; Marcacci M; Mencacci A; Puglia I; Rinaldi A; Savini G; Scialabba S
EPI_ISL_2769117, EPI_ISL_2858698, EPI_ISL_2875836, EPI_ISL_2875938, EPI_ISL_2875941, EPI_ISL_2875942	UniversitätsSpital Zürich	Institute of Medical Virology	Alexandra Trkola; Annette Audigé; Catharine Aquino; Cyril Shah; Daniel Ehrsam; Gabriela Ziltener; Guido Bloemberg; Hubert Rehrauer; Isabel Stürmer; Joel Wirz; Jon Huder; Jürg Böni; Kevin Steiner; Maria Grünberg; Maryam Zaheri; Michael Huber; Riccarda Capaul; Stefan Schmutz; Verena Kufner; Weihong Qi
EPI_ISL_2360239	UniversitätsSpital Zürich 009	Institute of Medical Virology	Alexandra Trkola; Annette Audigé; Cyril Shah; Gabriela Ziltener; Guido Bloemberg; Jon Huder; Jürg Böni; Kevin Steiner; Maria Grünberg; Maryam Zaheri; Michael Huber; Riccarda Capaul; Stefan Schmutz; Verena Kufner
EPI_ISL_2761960	Universitätsklinikum Frankfurt - Institut für Medizinische Virologie	Robert Koch Institute	
EPI_ISL_2290622	Uniwersyteckie Centrum Kliniczne	1. National Institute of Public Health - National Institute of Hygiene, Warsaw, Poland 2. Biobank Lab, University of Lodz 3. Laboratory of Respiratory Viruses, Teaching and Clinical Center of the Medical University of Lodz	Dominik Strapagiel; Izabela Dróżdż; Jakub Lach; Katarzyna Zacharczuk; Klaudyna Królikowska; Maciej Borowiec; Magdalena Nowakowska; Magdalena Traczyk-Borszyńska; Marcin Słomka; Marta Sobalska-Kwapis; Małgorzata Sadkowska-Todys; Tomasz Płoszaj; Tomasz Wołkowicz
EPI_ISL_2840601, EPI_ISL_2840602, EPI_ISL_2840603, EPI_ISL_2840604	Urban Institute for Disease Prevention and Control	National Institute of Health, Department of Medical Sciences, Ministry of Public Health, Thailand	; Natchaya Khiadsang; Nuttida Thongpramul; Pakorn Piromtong; Pilailuk Okada; Ratana Tacharoenmuang; Siripaporn Phuygun; Sittiporn Parnmen; Sunthareeya Waicharoen; Thanutsapa Thanadachakul; Warawan Wongboot; sirikanda wimol
EPI_ISL_2020949	Urban Public Health Centre (UPHC), SECTOR-2, Gandhinagar	Gujarat Biotechnology Research Centre	Amit Kanani; Amrutlal Patel; Bhargav Waghela; Bhumika Prajapati; Chaitanya Joshi; Dinesh Kumar; Janvi Raval; Kalpesh Goswami; Madhvi Joshi; Nilen Patel; Nitesh Shah; Nitin Savaliya; Ramesh Pandit; Sonal Sharma; Tejendra Purohit; Twinkle Soni; Umang Mishra; Zarna Patel; Zuber Saiyed
EPI_ISL_2291743, EPI_ISL_2678942, EPI_ISL_2856341, EPI_ISL_2856542, EPI_ISL_2856648, EPI_ISL_2857081, EPI_ISL_2857269, EPI_ISL_2857287, EPI_ISL_2857891			
see above	Utah Public Health Laboratory	Utah Public Health Laboratory	Erin L. Young; Kelly F. Oakeson; Olinto Linares-Perdomo; Tara Gallagher
EPI_ISL_1824682, EPI_ISL_1824683, EPI_ISL_1824684, EPI_ISL_2285157, EPI_ISL_2285158, EPI_ISL_2285159, EPI_ISL_2285160			
see above	VITHAS Lab	Microbiology Department. Complexo Hospitalario Universitario de Vigo	Camacho AT; Perez S; Regueiro B
EPI_ISL_2001196, EPI_ISL_2017735	VRDL, Goverment Medical College (GMC), Surat	Gujarat Biotechnology Research Centre	Chaitanya Joshi; Dinesh Kumar; Janvi Raval; Madhvi Joshi; Neeta Khandelwal; Nitesh Shah; Nitin Savaliya; Ramesh Pandit; Sonal Sharma; Twinkle Soni; Umang Mishra; Zarna Patel; Zuber Saiyed
EPI_ISL_2379667, EPI_ISL_2420172, EPI_ISL_2420215, EPI_ISL_2420238, EPI_ISL_2521350, EPI_ISL_2521371, EPI_ISL_2622049, EPI_ISL_2622050, EPI_ISL_2622055, EPI_ISL_2622063, EPI_ISL_2622067, EPI_ISL_2622076, EPI_ISL_2731277, EPI_ISL_2731278			
see above	Valais Hospital, Central Institute	Valais Hospital, Central Institute	Alexis Dumoulin; Deborah Penet; Emmanouil Dermitzakis; Henri Pegeot; Ioannis Xenarios; Keith Harshman; Lorenzo Cerutti; Melyssa Elies
EPI_ISL_1662284	Veer Surendra Sai Institute of Medical Sciences and Research, Burla, Sambalpur	Institute of Life Sciences - INSACOG	Ajay Parida; Amol M. Kanampalliwar; Arup Ghosh; Atimukta Jha; INSACOG Consortium; Punit Prasad; Rajeeb Swain; Rupesh Dash; Safal Walia; Shifu Aggarwal; Sunil K. Raghav
EPI_ISL_2675078, EPI_ISL_2675080, EPI_ISL_2675093, EPI_ISL_2675094, EPI_ISL_2725077, EPI_ISL_2790020, EPI_ISL_2790022			
see above	Vestfold Hospital, Toensberg Department of Microbiology	Norwegian Institute of Public Health, Department of Virology	Atiya R Ali; Debech Nadia; Engebretsen Serina Beate; Garcia Llorente Ignacio; Hilde Elshaug; Hilde Vollan; Jon Bråte; Kamilla Heddeland Instefjord; Karoline Bragstad; Kathrine Stene-Johansen; Line Victoria Moen; Marie Paulsen Madsen; Olav Hungnes; Pedersen Benedikte Nevjen; Rasmus Riis Kopperud
EPI_ISL_2820895, EPI_ISL_2820932	Vestfold Hospital, Toensberg Department of Microbiology	Norwegian Institute of Public Health, Department of Virology	Atiya R Ali; Debech Nadia; Engebretsen Serina Beate; Garcia Llorente Ignacio; Hilde Elshaug; Hilde Vollan; Jon Bråte; Kamilla Heddeland Instefjord; Karoline Bragstad; Kathrine Stene-Johansen; Line Victoria Moen; Marie Paulsen Madsen; Olav Hungnes; Pedersen Benedikte Nevjen; Rasmus Riis Kopperud
EPI_ISL_2878070, EPI_ISL_2885922	Viešoji įstaiga Klaipėdos universitetinė ligoninė	Institute of Biotechnology, Life Sciences Center, Vilnius University	Albertas Timinskas; Alma Gedvilaite; Danguole Ziogiene; Emilija Vasiliunaite; Milda Norkiene
EPI_ISL_2885938, EPI_ISL_2885944, EPI_ISL_2885945, EPI_ISL_2885946	Viešoji įstaiga Vilniaus universiteto ligoninė Santaros klinikos	Institute of Biotechnology, Life Sciences Center, Vilnius University	Albertas Timinskas; Alma Gedvilaite; Danguole Ziogiene; Emilija Vasiliunaite; Milda Norkiene
EPI_ISL_1914236, EPI_ISL_1914237, EPI_ISL_1914238, EPI_ISL_1914239	Viollier AG	Department of Biosystems Science and Engineering, ETH Zurich	Andrea Patrignani; Andreia Cabral de Gouvea; Catharine Aquino; Chaoran Chen; Christian Beisel; Christiane Beckmann; Christoph Noppen; David Dreifuss; Deborah Penet; Doris Popovic; Elodie Burcklen; Emmanouil Dermitzakis; Griffin White; Henri Pegeot; Ina Nissen; Ioannis Xenarios; Ivan Topolsky; Jay Tracy; Katharina Jahn; Keith Harshman; Lara Fuhrmann; Laura Neff; Lennart Opitz; Lorenzo Cerutti; Maria Domenica Moccia; Maurice Redondo; Mirjam Feldkamp; Natascha Santacroce; Niko Beerenwinkel; Noemie Santamaria de Souza; Olivier Kobel; Philipp Jablonski; Ralph Schlapbach; Rebecca Denes; Sarah Nadeau; Simon Gruter; Sophie Seidel; Tanja Stadler; Timothy Sykes
EPI_ISL_1750690, EPI_ISL_2017208, EPI_ISL_2017223, EPI_ISL_2017224, EPI_ISL_2017225, EPI_ISL_2018995, EPI_ISL_2019465, EPI_ISL_2212778, EPI_ISL_2307592, EPI_ISL_2307932, EPI_ISL_2308031, EPI_ISL_2375015, EPI_ISL_2375080, EPI_ISL_2375122, EPI_ISL_2375177, EPI_ISL_2375211, EPI_ISL_2375282, EPI_ISL_2462656, EPI_ISL_2462658, EPI_ISL_2462835, EPI_ISL_2462851, EPI_ISL_2462855, EPI_ISL_2544326, EPI_ISL_2544358, EPI_ISL_2544374, EPI_ISL_2544422, EPI_ISL_2611051, EPI_ISL_2662307, EPI_ISL_2662356, EPI_ISL_2662390, EPI_ISL_2662428, EPI_ISL_2662433, EPI_ISL_2662435, EPI_ISL_2724385, EPI_ISL_2724389, EPI_ISL_2724446, EPI_ISL_2724449, EPI_ISL_2724454, EPI_ISL_2724455, EPI_ISL_2724456, EPI_ISL_2724509, EPI_ISL_2724510, EPI_ISL_2820562, EPI_ISL_2820583, EPI_ISL_2820585, EPI_ISL_2820600			
see above	Viollier AG	Department of Biosystems Science and Engineering, ETH Zürich	Andrea Patrignani; Andrea Patrizia Salzmann; Andreia Cabral de Gouvea; Catharine Aquino; Chaoran Chen; Christian Beisel; Christiane Beckmann; Christoph Noppen; Daniel Ehrsam; David Dreifuss; Deborah Penet; Doris Popovic; Elodie Burcklen; Emmanouil Dermitzakis; Griffin White; Henri Pegeot; Henriette Kurth; Ina Nissen; Ioannis Xenarios; Isabel Stürmer; Ivan Topolsky; Jay Tracy; Katharina Jahn; Keith Harshman; Kim Philipp Jablonski; Lara Fuhrmann; Laura Neff; Lennart Opitz; Lorenzo

			Cerutti; Maria Domenica Moccia; Maurice Redondo; Mirjam Feldkamp; Natascha Santacroce; Niko Beerenwinkel; Noemie Santamaria de Souza; Olivier Kobel; Philipp Jablonski; Ralph Schlapbach; Rebecca Denes; Sarah Nadeau; Simon Grüter; Sophie Seidel; Tanja Stadler; Timothy Sykes
EPI_ISL_2100241, EPI_ISL_2100242	Viral Respiratory Infections Laboratory, Cantacuzino National Military-Medical Institute	Cantacuzino Institute Virology	Carmen Cherciu; Mihaela Lazar; Mihaela Oprea; Sorin Dinu
EPI_ISL_2135844	Viral Respiratory Lab, National Institute for Biomedical Research (INRB)	Pathogen Sequencing Lab, National Institute for Biomedical Research (INRB)	Allison Black; Amuri Aziza; Andrew Rambaut; Catherine Pratt; Eddy Kinganda-Lusamaki; Edith Nkwembe; Emmanuel Lokilo Lofiko; Francisca Muyembe Mawete; Ian Goodfellow; James Hadfield; Jean Claude Makangara; Jean-Jacques Muyembe Tamfum; Josh Quick; Kristian Andersen; Matthias Pauthner; Michael Wiley; Nick Loman; Placide Mbala-Kingebeni; Steve Ahuka-Mundeke; Trevor Bedford
EPI_ISL_2549198, EPI_ISL_2549199	Virology Department, Central Health Laboratory	The Francis Crick Institute	Bahadoor BS; Crawford M; Daniels RS; Goldstone R; Harvey R; Manraj SS; Nicod J; Patel H; Ramuth M; Sonoo J
EPI_ISL_2354628, EPI_ISL_2354633	Virology Department, Royal Infirmary of Edinburgh, NHS Lothian / School of Biological Sciences, University of Edinburgh	COVID-19 Genomics UK (COG-UK) Consortium	Colquhoun R; Cotton S; Dewar R; Gallagher A; Hill V; Jackson B; Maloney D; McCrone JT; McHugh M; O'Toole Á; Rambaut A; Rooke S; Scher E; Templeton K; Yu X
EPI_ISL_2627360, EPI_ISL_2627362, EPI_ISL_2627363, EPI_ISL_2627365, EPI_ISL_2627367, EPI_ISL_2627368, EPI_ISL_2627369, EPI_ISL_2627370, EPI_ISL_2627372, EPI_ISL_2627373, EPI_ISL_2627374, EPI_ISL_2627375, EPI_ISL_2627376, EPI_ISL_2627377, EPI_ISL_2627378, EPI_ISL_2627379, EPI_ISL_2627380, EPI_ISL_2627381, EPI_ISL_2627382, EPI_ISL_2627383, EPI_ISL_2627384, EPI_ISL_2627385, EPI_ISL_2627386, EPI_ISL_2627387, EPI_ISL_2627390, EPI_ISL_2627391, EPI_ISL_2627392, EPI_ISL_2627393			
see above	Virology Laboratory, International Centre for Diarrhoeal Disease Research, Bangladesh (ICDDR,B)	Virology Laboratory, International Centre for Diarrhoeal Disease Research, Bangladesh (ICDDR,B)	Md. Mahfuzur Rahman; Mohammad Enayet Hossain; Mohammed Ziaur Rahman; Mojnu Miah; Mustafizur Rahman; Rashedul Hasan
EPI_ISL_1970571, EPI_ISL_1970572, EPI_ISL_2081229, EPI_ISL_2884494, EPI_ISL_2884627, EPI_ISL_2884629	Virology Laboratory, Scientific Department, Army Medical Center	Virology Laboratory, Scientific Department, Army Medical Center	Anella Monte; Anna Anselmo; Antonella Fortunato; Filippo Molinari; Florigio Lista; Francesco Giordani; Giancarlo Petralito; Giandomenico Cerreto; Giulia Campoli; Lucia Nicosia; Marzia Cavalli; Riccardo De Sanctis; Rossella Brandi; Silvia Fillo; Stella Lia; Vanessa Vera Fain
EPI_ISL_2501060, EPI_ISL_2608428, EPI_ISL_2608429, EPI_ISL_2608440, EPI_ISL_2693911, EPI_ISL_2693913, EPI_ISL_2693914, EPI_ISL_2693915, EPI_ISL_2693919, EPI_ISL_2693920, EPI_ISL_2693921, EPI_ISL_2693926, EPI_ISL_2693936, EPI_ISL_2693937, EPI_ISL_2693938, EPI_ISL_2693942, EPI_ISL_2801922, EPI_ISL_2801923			
see above	Virology Unit, Institut Pasteur du Cambodge	Virology Unit, Institut Pasteur du Cambodge	Cecile Troupin; Chau Darapheak; Chin Savuth; Erik A Karlsson; Jurre Y Siegers; Kraing Sidonn; Leakhena Pum; Ly Sovann; Veasna Duong; Yi Sengdoeurn
EPI_ISL_2363818, EPI_ISL_2363821, EPI_ISL_2508228, EPI_ISL_2645129	Vita Laboratoriot Oy	Expert Microbiology, National Institute for Health and Welfare	Carita Savolainen-Kopra; Erika Lindh; Haider al-Hello; Jani Halkilahti; Kirsi Liitsola; Niina Ikonen; Olli Vapalahti; Pekka Ellonen; Phuoc Truong; Päivi Laurila; Ravi Kant; Sari Hannula; Soile Blomqvist; Teemu Smura
EPI_ISL_2444966, EPI_ISL_2503925	Vitomed Sp. z o.o.	1. Academic Center for Pathomorphological and Genetic-Molecular Diagnostics ltd, Bialystok, Poland 2. National Institute of Public Health - National Institute of Hygiene, Warsaw, Poland	Anetta Sulewska; Jacek Nikliński; Janusz Dzięcioł; Joanna Kiśluk; Katarzyna Zacharczuk; Konrad Raczkowski; Magdalena Nowakowska; Małgorzata Sadkowska-Todys; Piotr Karabowicz; Piotr Majewski; Przemysław Biecek. Joanna Reszeć; Radosław Charkiewicz; Tomasz Wołkowicz
EPI_ISL_2836955	WACCBIP, University of Ghana, Accra, Ghana	WACCBIP, University of Ghana, Volta Road, Legon-Accra, Ghana	Collins M. Morang'a; Dominic S.Y. Amuzu; Evelyn B. Quansah; Evelyn Y. Bonney; Frederick Tei-Maya; Ivy A. Asante; Joe K. Mutungi; John K. Odoom; Joseph H.K. Bonney; Joyce M. Ngoi; Lawrence Ofori-Boadu; Lucas N. Amenga-Etego and Gordon A. Awandare; Mildred Adusei-Poku; Nicaise T. Ndam; Peter K Quashie; Philip M. Soglo; Samirah Saiid; Vincent Appiah; Violette V. M'cormack; William K. Ampofo; Yaw Bediako
EPI_ISL_1789541, EPI_ISL_1789542, EPI_ISL_2385077, EPI_ISL_2385081, EPI_ISL_2385085, EPI_ISL_2385090, EPI_ISL_2385091, EPI_ISL_2385095, EPI_ISL_2385096, EPI_ISL_2385101, EPI_ISL_2385113, EPI_ISL_2385114, EPI_ISL_2385116, EPI_ISL_2385119, EPI_ISL_2385121, EPI_ISL_2385123, EPI_ISL_2385125, EPI_ISL_2385128, EPI_ISL_2385129, EPI_ISL_2385138, EPI_ISL_2385142, EPI_ISL_2385143, EPI_ISL_2385144, EPI_ISL_2385145, EPI_ISL_2385146, EPI_ISL_2385147, EPI_ISL_2385148, EPI_ISL_2385149, EPI_ISL_2385152, EPI_ISL_2385158, EPI_ISL_2385159, EPI_ISL_2385162, EPI_ISL_2385163, EPI_ISL_2385165, EPI_ISL_2385167, EPI_ISL_2385169, EPI_ISL_2385170, EPI_ISL_2385172, EPI_ISL_2385173, EPI_ISL_2385174, EPI_ISL_2385175, EPI_ISL_2385176, EPI_ISL_2385178, EPI_ISL_2385179, EPI_ISL_2385184, EPI_ISL_2385185, EPI_ISL_2385187, EPI_ISL_2385188, EPI_ISL_2385193, EPI_ISL_2385195, EPI_ISL_2385204, EPI_ISL_2385206, EPI_ISL_2385311, EPI_ISL_2385319, EPI_ISL_2385323, EPI_ISL_2385324, EPI_ISL_2450462, EPI_ISL_2450465, EPI_ISL_2450466, EPI_ISL_2450470, EPI_ISL_2450479, EPI_ISL_2450489, EPI_ISL_2450490, EPI_ISL_2450499, EPI_ISL_2450503, EPI_ISL_2450508, EPI_ISL_2450510, EPI_ISL_2450521, EPI_ISL_2450525, EPI_ISL_2450526, EPI_ISL_2450527, EPI_ISL_2450530, EPI_ISL_2450535, EPI_ISL_2450536, EPI_ISL_2450538, EPI_ISL_2450540, EPI_ISL_2450542, EPI_ISL_2450597, EPI_ISL_2450600, EPI_ISL_2450604, EPI_ISL_2462879, EPI_ISL_2523389, EPI_ISL_2523406, EPI_ISL_2523444, EPI_ISL_2523469, EPI_ISL_2523480, EPI_ISL_2523503, EPI_ISL_2523516, EPI_ISL_2523522, EPI_ISL_2523549, EPI_ISL_2523559, EPI_ISL_2523568, EPI_ISL_2523587, EPI_ISL_2523678, EPI_ISL_2523690, EPI_ISL_2523738, EPI_ISL_2523747, EPI_ISL_2523762, EPI_ISL_2523768, EPI_ISL_2523783, EPI_ISL_2698461, EPI_ISL_2698473, EPI_ISL_2698487, EPI_ISL_2698552, EPI_ISL_2698564, EPI_ISL_2698593, EPI_ISL_2698631, EPI_ISL_2816150, EPI_ISL_2816152, EPI_ISL_2816169, EPI_ISL_2816172, EPI_ISL_2816190			
see above	WHO National Influenza Centre Russian Federation	WHO National Influenza Centre Russian Federation	Alexey Masharsky; Andrey Komissarov; Artem Fadeev; Daria Danilenko; Dmitry Lioznov; Elena Nabieva; Georgii Bazykin; Kirill Varchenko; Ksenia Safina; Kseniya Komissarova; Maria Baturova; Maria Pisareva; Maria Timofeeva; Mikhail Bakaev; Nikita Yolshin; Oula Mansour; Oula Masour; Tamila Musaeva; Veronika Eder
EPI_ISL_2566734	WSSE Gorzów Wlkp.	1. National Institute of Public Health - National Institute of Hygiene; 2. Eurofins Genomics Europe Sequencing GmbH	ECDC COVID-19 WGS support team; Eurofins Genomics Europe Sequencing Team; Gierczyński Rafał; Sadkowska-Todys Małgorzata; Wołkowicz Tomasz; Zacharczuk Katarzyna
EPI_ISL_1909092, EPI_ISL_1909093, EPI_ISL_1909094, EPI_ISL_1909095	WSSE Katowice	1. Academic Center for Pathomorphological and Genetic-Molecular Diagnostics ltd, Bialystok, Poland 2. National Institute of Public Health - National Institute of Hygiene, Warsaw, Poland	Anetta Sulewska; Jacek Nikliński; Janusz Dzięcioł; Joanna Kiśluk; Katarzyna Zacharczuk; Konrad Raczkowski; Magdalena Nowakowska; Małgorzata Sadkowska-Todys; Piotr Karabowicz; Piotr Majewski; Przemysław Biecek. Joanna Reszeć; Radosław Charkiewicz; Tomasz Wołkowicz
EPI_ISL_2377205, EPI_ISL_2377207	WSSE Katowice	1. National Institute of Public Health - National Institute of Hygiene; 2. Eurofins Genomics Europe Sequencing GmbH	ECDC COVID-19 WGS support team; Eurofins Genomics Europe Sequencing Team; Gierczyński Rafał; Sadkowska-Todys Małgorzata; Wołkowicz Tomasz; Zacharczuk Katarzyna
EPI_ISL_2131214	WSSE Warszawa	1. National Institute of Public Health - National Institute of Hygiene, Warsaw, Poland 2. Biobank Lab, University of Lodz 3. Laboratory of Respiratory Viruses, Teaching and Clinical Center of the Medical University of Lodz	Dominik Strapagiel; Izabela Dróżdż; Jakub Lach; Katarzyna Zacharczuk; Klaudyna Królikowska; Maciej Borowiec; Magdalena Nowakowska; Magdalena Traczyk-Borszyńska; Marcin Słomka; Marta Sobalska-Kwapis; Małgorzata Sadkowska-Todys; Tomasz Płoszaj; Tomasz Wołkowicz
EPI_ISL_2365681, EPI_ISL_2448918, EPI_ISL_2448927, EPI_ISL_2448928, EPI_ISL_2448929	WSSE w Gdańsku	1. National Institute of Public Health - National Institute of Hygiene, Warsaw, Poland 2. Biobank Lab, University of Lodz 3. Laboratory of Respiratory Viruses, Teaching and Clinical Center of the Medical University of Lodz	Dominik Strapagiel; Izabela Dróżdż; Jakub Lach; Katarzyna Zacharczuk; Klaudyna Królikowska; Maciej Borowiec; Magdalena Nowakowska; Magdalena Traczyk-Borszyńska; Marcin Słomka; Marta Sobalska-Kwapis; Małgorzata Sadkowska-Todys; Tomasz Płoszaj; Tomasz Wołkowicz
EPI_ISL_2090620, EPI_ISL_2090622, EPI_ISL_2090623, EPI_ISL_2094440	WSSE w Warszawie	National Institute of Public Health - National Institute of Hygiene	Gierczyńki Rafał; Sadkowska-Todys Małgorzata; Wołkowicz Tomasz; Zacharczuk Katarzyna
EPI_ISL_2031771	Weill Cornell Medicine	New York Genome Center	Andre Corvelo; Arryn Craney; Chris Mason; Dayna M. Oschwald; Hanna Rennert; Lars F Westblade; Margaret Elizabeth Ross; Melissa Cushing; Michael Zody; Olivier Elemento; Priya Velu; Samantha Fennessey; Tom Maniatis
EPI_ISL_2709409, EPI_ISL_2709410, EPI_ISL_2709411, EPI_ISL_2709412, EPI_ISL_2709413, EPI_ISL_2709414, EPI_ISL_2709415, EPI_ISL_2709418, EPI_ISL_2709422, EPI_ISL_2709423, EPI_ISL_2709424, EPI_ISL_2709426, EPI_ISL_2709427, EPI_ISL_2709436, EPI_ISL_2709443, EPI_ISL_2709445			
see above	West Java Health Laboratory	Eijkman Institute for Molecular Biology, National Research and Innovation Agency; West Java Health Laboratory	Amin Soebandrio; Azzania Fibriani; Cut Nur Cinthia Alamanda; Edison Johar; Ema Rahmawati; Frilasita A Yudhaputri; Hidayat Trimarsanto; Iskandar Adnan; Khin Saw Myint; Lidwina Priliani; Lydia V. Panggalo; Muhammad Rezki Rasyak; Rifky Waluyajati Rachman; Ryan Bayusantika Ristandi; Safarina G Malik; Sukma Oktavianthi; Willy Agustine
EPI_ISL_2885887	Wichita State University- Molecular Diagnostic Lab	Kansas Health and Environmental Lab	Ben Olsen; Jonathan Barnell; Katherine Wiggins; Mike Grose; and Phil Adam
EPI_ISL_2558139	Wisconsin State Laboratory of Hygiene Communicable Disease Division	Wisconsin State Laboratory of Hygiene Communicable Disease Division	Abigail C. Shockey; Alicia J. Mooney; Erika M. Hanson; Kelsey R. Florek; Richard Griesser; Sara Wagner; Tonya Danz
EPI_ISL_2854269	Wojewodzka Stacja Sanitarno-Epidemiologiczna w Lodzi, Oddzial Laboratoryjny Mikrobiologii i Parazytologii	Wojewodzka Stacja Sanitarno-Epidemiologiczna w Lodzi, Oddzial Laboratoryjny Mikrobiologii i Parazytologii	Agnieszka Karnowska; Aleksandra Krawczyk; Danuta Bartczak; Elian Terpo; Katarzyna Kosowska; Lucyna Kochanowska; Marta Gawronska; Martyna Matera; Szymon Konkolewski
EPI_ISL_2350908, EPI_ISL_2350914, EPI_ISL_2776150, EPI_ISL_2835690, EPI_ISL_2835695, EPI_ISL_2835696	Wojewodzka Stacja Sanitarno-Epidemiologiczna w Olsztynie, Laboratorium Badan Epidemiologiczno-Klinicznych	Wojewodzka Stacja Sanitarno-Epidemiologiczna w Olsztynie, Laboratorium Badan Epidemiologiczno-Klinicznych	Aleksandra Kobiatko; Barbara Dolinska; Emilia Tarabasz; Ewa Liszewska; Marta Lukian; Monika Czerminska; Patryk Bielecki; Paulina Rozycka; Sylwia Krzetowska; Tomasz Jakubczak
EPI_ISL_2646302, EPI_ISL_2893464	ZOL	Jessa	Berden et al. on behalf of Jessa_cmdLab; Severine Berden et al. on behalf of the Jessa_cmdLab
EPI_ISL_2543750	Zakład Mikrobiologii Klinicznej WSZ im dr R. Ostrzyckiego w Koninie	1. National Institute of Public Health - National Institute of Hygiene, Warsaw, Poland 2. Biobank Lab, University of Lodz 3. Laboratory of Respiratory Viruses, Teaching and Clinical Center of the Medical University of Lodz	Dominik Strapagiel; Izabela Dróżdż; Jakub Lach; Katarzyna Zacharczuk; Klaudyna Królikowska; Maciej Borowiec; Magdalena Nowakowska; Magdalena Traczyk-Borszyńska; Marcin Słomka; Marta Sobalska-Kwapis; Małgorzata Sadkowska-Todys; Tomasz Płoszaj; Tomasz Wołkowicz
EPI_ISL_2693481, EPI_ISL_2693485, EPI_ISL_2693487	Zavod za javno zdravstvo Međimurske Županije	Hrvatski zavod za javno zdravstvo	Irena Tabain; Ivana Ferenčak
EPI_ISL_2693526, EPI_ISL_2693531, EPI_ISL_2693532, EPI_ISL_2693533, EPI_ISL_2693535, EPI_ISL_2693536, EPI_ISL_2693537, EPI_ISL_2693539, EPI_ISL_2693540, EPI_ISL_2693541, EPI_ISL_2693542, EPI_ISL_2693543, EPI_ISL_2693544, EPI_ISL_2693545, EPI_ISL_2693577			
see above	Zavod za javno zdravstvo Šibensko- Kninske Županije	Hrvatski zavod za javno zdravstvo	Irena Tabain; Ivana Ferenčak
EPI_ISL_1911195, EPI_ISL_1911196, EPI_ISL_1911197, EPI_ISL_1911250	Zhejiang Provincial Center for Disease Control and Prevention Zhoushan Center for Disease Prevention and Contol	Zhejiang Province Center of Disease Control and prevention	Bing WU; Hongling Wang; Yanjun Zhang
EPI_ISL_2115877	amedes MVZ Zentrum fÃ¼r Pathologie und Zytodiagnostik GmbH (KÃ¶ln)	Robert Koch Institute	
EPI_ISL_2130761	amedes MVZ fÃ¼r Laboratoriumsmedizin Hamburg	Robert Koch Institute	

EPI_ISL_2561981, EPI_ISL_2561993, EPI_ISL_2561999, EPI_ISL_2562003, EPI_ISL_2562006, EPI_ISL_2562010, EPI_ISL_2562012			
see above	cerballiance-IDF	Cerba lab	Aude Lessenne; Bénédicte Roquebert; Emmanuel Lecorche; Kader Merah; Laura Verdurme; Patrice Herisson; Sabine Trombert-Paolantoni; Stéphanie Haïm-Boukobza; Thierry Collin
EPI_ISL_2845197	labopart - Medizinische Laboratorien Dresden	Robert Koch Institute	
EPI_ISL_2795753	laboratoire Belle Epine	Department of Virology, Henri Mondor University Hospital, Assistance Publique Hôpitaux de Paris, Université Paris-Est Créteil, INSERM U955	Alexandre Soulier; Christophe Rodriguez; Elisabeth Trawinski; Guillaume Gricourt; Jean-Michel Pawlotsky; Melissa N'Debi; Slim Fourati; Vanessa Demontant
EPI_ISL_2760488	nordlab - Partnerschaftspraxis für Laboratoriumsmedizin	Robert Koch Institute	
